# Frailty, Home Time, and Health Care Costs in Older Adults With Atrial Fibrillation Receiving Oral Anticoagulants

**DOI:** 10.1001/jamanetworkopen.2023.42264

**Published:** 2023-11-09

**Authors:** Kueiyu Joshua Lin, Daniel E. Singer, Darae Ko, Robert Glynn, Mehdi Najafzadeh, Su Been Lee, Lily Gui Bessette, Alexander Cervone, Elyse DiCesare, Dae Hyun Kim

**Affiliations:** 1 Division of Pharmacoepidemiology and Pharmacoeconomics, Department of Medicine, Brigham and Women’s Hospital, Harvard Medical School, Boston, Massachusetts; 2 Division of General Internal Medicine, Department of Medicine, Massachusetts General Hospital, Harvard Medical School, Boston; 3 Section of Cardiovascular Medicine, Boston Medical Center, Boston, Massachusetts; 4 Hinda and Arthur Marcus Institute for Aging Research, Hebrew SeniorLife, Harvard Medical School, Boston, Massachusetts; 5 Division of Gerontology, Department of Medicine, Beth Israel Deaconess Medical Center, Harvard Medical School, Boston, Massachusetts

## Abstract

**Importance:**

There are no data on patient-centered outcomes and health care costs by frailty in patients with atrial fibrillation (AF) taking oral anticoagulants (OACs).

**Objective:**

To compare home time, clinical events, and health care costs associated with OACs by frailty levels in older adults with AF.

**Design, Setting, and Participants:**

This community-based cohort study assessed Medicare fee-for-service beneficiaries 65 years or older with AF from January 1, 2013, to December 31, 2019. Data analysis was performed from January to December 2022.

**Exposures:**

Apixaban, rivaroxaban, and warfarin use were measured from prescription claims. Frailty was measured using a validated claims-based frailty index.

**Main outcomes and measures:**

Outcome measures were (1) home time (days alive out of the hospital and skilled nursing facility) loss greater than 14 days; (2) a composite end point of ischemic stroke, systemic embolism, major bleeding, or death; and (3) total cost per member per year after propensity score overlap weighting.

**Results:**

The weighted population comprised 136 551 beneficiaries, including 45 950 taking apixaban (mean [SD] age, 77.6 [7.3] years; 51.3% female), 45 320 taking rivaroxaban (mean [SD] age, 77.6 [7.3] years; 51.9% female), and 45 281 taking warfarin (mean [SD] age, 77.6 [7.3] years; 52.0% female). Compared with apixaban, rivaroxaban was associated with increased risk of home time lost greater than 14 days (risk difference per 100 persons, 1.8 [95% CI, 1.5-2.1]), composite end point (rate difference per 1000 person-years, 21.3 [95% CI, 16.4-26.2]), and total cost (mean difference, $890 [95% CI, $652-$1127]), with greater differences among the beneficiaries with frailty. Use of warfarin relative to apixaban was associated with increased home time lost (risk difference per 100 persons, 3.2 [95% CI, 2.9-3.5]) and composite end point (rate difference per 1000 person-years, 29.4 [95% CI, 24.5-34.3]), with greater differences among the beneficiaries with frailty. Compared with apixaban, warfarin was associated with lower total cost (mean difference, −$1166 [95% CI, −$1396 to −$937]) but higher cost when excluding OAC cost (mean difference, $1409 [95% CI, $1177 to $1642]) regardless of frailty levels.

**Conclusions and Relevance:**

In older adults with AF, apixaban was associated with increased home time and lower rates of clinical events than rivaroxaban and warfarin, especially for those with frailty. Apixaban was associated with lower total cost compared with rivaroxaban but higher cost compared with warfarin due to higher OAC cost. These findings suggest that apixaban may be preferred for older adults with AF, particularly those with frailty.

## Key Points

**Question:**

What is the association of frailty level with oral anticoagulant outcomes in terms of home time, clinical events, and total health care costs in patients with atrial fibrillation (AF)?

**Findings:**

In this cohort study of 136 551 Medicare beneficiaries with AF, apixaban was associated with increased home time and fewer clinical events than rivaroxaban and warfarin, with greater differences among those with frailty. Apixaban was associated with a lower cost than rivaroxaban but a higher cost than warfarin because of a higher anticoagulant cost.

**Meaning:**

These results suggest that apixaban may be preferred for older adults with AF, particularly for patients with frailty.

## Teaser

This cohort study of Medicare beneficiaries with arial fibrillation compares home time, health care cost, and clinical events associated with commonly prescribed oral anticoagulants by frailty level.

## Introduction

Atrial fibrillation (AF) is associated with significant morbidity and mortality in older adults.^1,2,3^ Despite the availability of direct oral anticoagulants (DOACs),^4,5,6,7^ the use of oral anticoagulants (OACs) remains suboptimal in older adults with AF.^3^ Frailty is among the main concerns that complicate prescribing decisions^8^ and is highly associated with the risk of bleeding,^9^ falls,^10^ and drug-related adverse events.^11^ Although the prevalence of frailty in older adults with AF ranges from 17% in the community to 62% in the hospital,^12^ patients with frailty are severely underrepresented in clinical trials, leading to limited data to inform OAC prescribing^8^ and suboptimal use in older adults with AF and with frailty.^13,14^ In a recent US nationwide trend analysis,^3^ frailty was associated with 26% lower odds of OAC prescribing among older adults. However, the evidence generated based on routine care on OACs in patients with frailty remains limited,^15^ and no prior studies have investigated the use of OACs with a focus on patient-centered outcomes and health care costs among patients with AF and with frailty.

Home time, defined as the number of days alive and spent out of the hospital or a skilled nursing facility (SNF), is a validated patient-centered outcome^16,17,18,19^ that is well correlated with multiple other patient-reported outcomes, including poor self-rated health, mobility impairment, depressed mood, limited social activity, and difficulty in self-care.^18^ It has been endorsed by patients and stakeholders.^20,21,22,23^ Home time was previously used in some cardiovascular trials^24,25^ and in warfarin-treated patients with AF as the outcome of interest,^19^ but it has not been investigated among patients with AF taking DOAC in the routine care setting. Another outcome that is understudied but highly relevant to patients and society is health care costs, which can represent the overall burden of AF, OAC treatment, frailty, and other multimorbidity on our health care systems. Home time and health care costs reflect the perspectives of patients and health care systems in a more holistic way, not limited to health care use resulting from ischemic stroke or major bleeding events.

In this study, we compared commonly prescribed OACs—apixaban, rivaroxaban, and warfarin (dabigatran constituted only approximately 0.2% and edoxaban <0.02% of all OAC users with AF in the US in 2020^3^)—with respect to home time, health care cost, and clinical events by frailty level in older adults with AF. We hypothesized that the association of different OAC treatments with home time and health care costs would differ by the patient’s frailty level and that the absolute difference in harms or benefits would be greater in patients with higher frailty.

## Methods

### Study Population and OAC Exposure

On the basis of Medicare fee-for-service claims data, using a target trial emulation framework,^26,27^ we performed a cohort study that emulated a hypothetical clinical trial to compare the patient-centered outcome, total costs, and clinical events among patients with AF initiating apixaban, rivaroxaban, and warfarin therapy between January 1, 2013, and December 31, 2019. The inclusion criteria were as follows: (1) 65 years or older; (2) dispensed apixaban, rivaroxaban, or warfarin without any OAC dispensed in the preceding year; (3) 1 or more diagnosis codes of AF in the preceding year; (4) elevated risk of ischemic stroke based on a CHA_2_DS_2_-VASc (congestive heart failure, hypertension, age ≥75 years [doubled], diabetes, stroke [doubled], vascular disease, age 65 to 74 years, and sex category [female]) score of 2 or higher for men and 3 or higher for women; and (5) continuous medical and pharmacy coverage without missing data on age, sex, race, or region (to ensure we had sufficient data to ascertain baseline characteristics). The cohort entry (index) date was the date of OAC dispensing, and the 365-day period before the index date was used as the baseline assessment period. Consistent with clinical trials, we applied the following additional exclusion criteria^4,5,6,7^: (1) received hospice care, (2) had other indications for OAC (venous thromboembolism or joint replacement), (3) had contraindications to either DOAC or warfarin (major bleeding within the previous 14 days, valvular diseases, or end-stage kidney disease), or (4) had an inpatient diagnosis of ischemic stroke in the previous 14 days. We chose apixaban as the referent group because it is currently the most commonly prescribed OAC in the US.^3,28^ This study was approved by the institutional review board of Brigham and Women’s Hospital. This study follows the Strengthening the Reporting of Observational Studies in Epidemiology (STROBE) reporting guideline for cohort studies. Detailed definitions of these conditions are provided in the eAppendix in Supplement 1.

### Measurement of Baseline Characteristics and Frailty

To control for potential confounding, we assessed age, sex, race, calendar time, geographic region, chronic conditions, prescription drug use, health care use, and costs from claims data in the baseline assessment period (eAppendix in Supplement 1). Race information was based on the self-reported demographic data recorded in the administrative insurance claims data, and we collected the race information to adjust for potential confounding of the treatment effect of OACs. We used the missing indicator method to handle missing information on race and region. We estimated frailty by using the Kim claims-based frailty index (CFI),^29,30,31,32^ which has been validated against physical function,^30^ frailty phenotype,^31^ deficit-accumulation frailty index,^31^ and severe disability.^31^ Beneficiaries were classified as nonfrail if the CFI was less than 0.15, prefrail if the CFI was 0.15 to less than 0.25, and frail if the CFI was 0.25 or greater.^33,34,35^ We also computed the CHA_2_DS_2_-VASc score,^36,37^ the modified HAS-BLED (hypertension, abnormal renal/liver function, stroke, bleeding history or predisposition, labile international normalized ratio, elderly, and drugs/alcohol concomitantly) score (excluding labile international normalized ratio),^37,38,39^ and the Gagne Combined Comorbidity Score (CCS).^40^

### Outcomes and Follow-Up

Beneficiaries were followed up from the day after OAC initiation until the earliest occurrence of the clinical end points, death, disenrollment from medical insurance, end of data availability, or 365 days, regardless of OAC adherence (intention-to-treat analysis). The follow-up was truncated at 365 days to minimize treatment misclassification because of declining adherence beyond that point. To allow the study cohort to have the 365-day follow-up, the last cohort entry date was December 31, 2019.

The first coprimary outcome was more than 14 days of home time lost during 365 days. The cutoff of 14 days was based on a prior validation study showing that a loss of home time greater than 14 days during 365 days was correlated with deterioration in mobility impairment, depression, and difficulty in self-care.^18^ Home time was calculated by subtracting the number of hospital days and SNF days from the total number of days alive during the follow-up period. This outcome could range from 0 days (ie, dying immediately after OAC initiation or spending the entire period in a hospital or SNF) to 365 days (ie, surviving 365 days without death, any hospitalizations, or any SNF stays). For those censored because of Medicare disenrollment or the end of data availability, we extrapolated the proportion of home time during the observed follow-up period to 365 days (ie, observed number of days at home / number of observed follow-up days × 365).

The second coprimary outcome was the total health care cost during 365 days identified by recorded Medicare payments. This outcome included costs of inpatient and outpatient encounters, pharmacy, SNF, home health, hospice, and durable medical equipment claims. We also assessed the total cost excluding the OAC costs. For beneficiaries whose follow-up was shorter than 365 days because of disenrollment from Medicare, we calculated the observed daily cost and multiplied it by 365 days. For those who died before 365 days, we used only the observed cost before death (ie, the cost after death was zero and the predeath cost was not extrapolated into the period after death).

To understand potential reasons underlying the observed home time and cost differences, we assessed a composite clinical end point of ischemic stroke or systemic embolism, major bleeding, and death and its individual components as secondary outcomes during the same follow-up period up to 365 days after cohort entry. The date of death was obtained through linkage to Social Security files or discharge status. Clinical events were defined using claims-based validated algorithms with positive predictive values of 85% to 90% for ischemic stroke^41,42^ and 86% to 96% for major bleeding^43^ (eAppendix in Supplement 1).

### Statistical Analysis

To reduce confounding and ensure comparability among beneficiaries who could potentially receive apixaban, rivaroxaban, or warfarin, we performed propensity score (PS)–based overlap weighting using the R package PSweight (R Foundation for Statistical Computing). This procedure estimates the mean treatment effect in a target population with the most overlap in covariates across the treatment groups by down-weighting individuals with an extreme PS.^44^ The PS was estimated as the probabilities of initiating each OAC using a multinomial logistic regression that included the abovementioned (a total of 91) baseline covariates (eTable 1 in Supplement 1 for the full list). We assessed covariate balance before and after overlap weighting using standardized mean differences. An absolute standardized mean difference less than 0.1 was considered an adequate balance. We estimated risk differences (per 100 persons) and 95% CIs of home time lost between an OAC and apixaban (reference) using weighted binomial regression. We estimated the mean cost differences and 95% CIs using a generalized linear model with γ-distribution and identity link to model a skewed distribution of cost.^45,46,47^ For clinical end points, we estimated the rate differences (per 1000 person-years) and 95% CIs using a generalized linear model with Poisson distribution and identity link, with follow-up time as the offset. For frailty subgroup analyses, we repeated PS estimation and outcome regression within each frailty subgroup and tested heterogeneity in effect estimates across the subgroups by the χ^2^ statistic from the Cochran *Q* test.^48^ Because PS weighting was performed within each subgroup, the weighted sample size in subgroups did not add up to the weighted sample size of the total population.

We conducted the following sensitivity analyses to test the robustness of our results. First, we tried to minimize the possibility of misclassifying recent strokes and major bleeding as incident events by excluding beneficiaries with those diagnosis codes in the 60 days before the index date. Second, because medication use during the SNF stay is unavailable in our databases, we excluded those with SNF stays during the baseline assessment period to ensure new OAC use. Third, to assess the potential influence of COVID-19 on the study findings, we repeated all the analyses after excluding data from 2020. Data analysis was performed from January to December 2022. Analyses were conducted in R software, version 4.1.1 and the Aetion Evidence Generation Platform, which has been validated by accurately replicating published studies.^49,50,51^ A 2-sided *P* < .05 was considered statistically significant.

## Results

### Characteristics of the Study Populations

The analytic cohort included 288 204 apixaban, 158 101 rivaroxaban, and 147 791 warfarin initiators (Figure). Before PS weighting (eTable 1 in Supplement 1), compared with apixaban users, rivaroxaban users were younger (apixaban vs rivaroxaban: mean [SD], 78.0 [7.5] vs 76.9 [7.1] years) and had a lower CHA_2_DS_2_-VASc score (4.3 [1.5] vs 4.1 [1.4]), had a lower CCS (3.3 [2.8] vs 3.0 [2.6]), lower prevalence of chronic kidney disease (20.2% vs 15.1%), and lower prevalence of frailty (23.1% vs 19.8%). Compared with apixaban users, warfarin users were similar with regard to age (apixaban vs warfarin: 78.0 [7.5] vs 78.0 [7.3] years), CHA_2_DS_2_-VASc score (4.3 [1.5] vs 4.4 [1.5]), CCS (3.3 [2.8] vs 3.5 [2.8]), prevalence of chronic kidney disease (20.2% vs 22.1%), and frailty (23.1% vs 25.5%). By frailty levels (eTables 2-4 in Supplement 1), beneficiaries with frailty were older (nonfrail vs frail: 75.4-77.0 vs 78.8-79.9 years), were more likely to be female (37.5%-40.3% vs 60.5%-64.3%) and Black or other race (6.2%-7.5% vs 10.5%-11.9%), had higher CHA_2_DS_2_-VASc scores (3.1-3.2 vs 5.3-5.5), and had higher CCSs (1.4-1.5 vs 5.5-5.9). This pattern was consistent in all OAC users.

**Figure.**
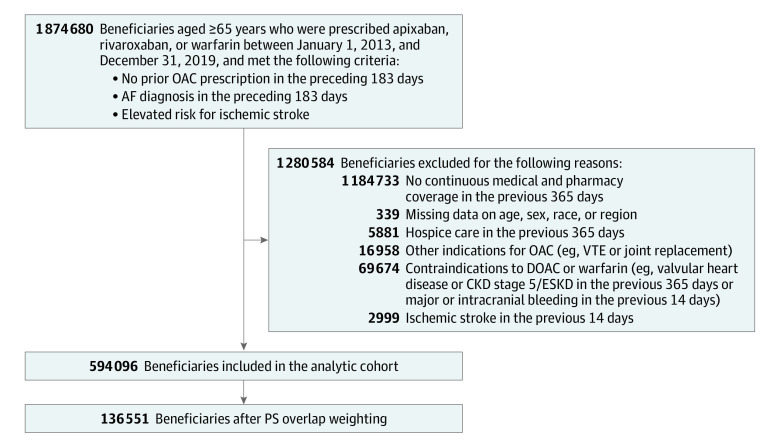
Cohort Selection Flow Diagram AF indicates atrial fibrillation; CKD, chronic kidney disease; DOAC, direct-acting oral anticoagulant; ESKD, end-stage kidney disease; OAC, oral anticoagulant; PS, propensity score; VTE, venous thromboembolism.

Propensity score overlap weighting produced a weighted population of 136 551 beneficiaries with balanced baseline characteristics (absolute standardized mean difference <0.1) across OAC groups (selected variables in Table 1; see eTable 1 in Supplement 1 for the entire list) as well as within each frailty level (eTables 2-4 in Supplement 1). Among the 45 950 patients taking apixaban, the mean (SD) age was 77.6 (7.3) years; 51.3% were female and 48.7% were male; and 4.1% were Black, 91.2% White, and 4.6% other or missing race. Among the 45 320 taking rivaroxaban, the mean (SD) age was 77.6 (7.3) years; 51.9% were female and 48.1% were male; and 4.3% were Black, 91.0% White, and 4.7% other or missing race. Among the 45 281 taking warfarin, the mean (SD) age was 77.6 (7.3) years; 52.0% were female and 48.0% were male; and 4.2% were Black, 91.2% White, and 4.6% other or missing race. For the apixaban, rivaroxaban, and warfarin groups, respectively, mean (SD) CHA_2_DS_2_-VASc scores were 4.2 (1.5), 4.3 (1.5), and 4.3 (1.5); mean CCSs were 3.2 (2.7), 3.3 (2.7), and 3.3 (2.7); and the percentages of patients with frailty were 22.2%, 23.5%, and 23.2%.

**Table 1.** Propensity Score Overlap–Weighted Populations of Medicare Fee-for-Service Beneficiaries With Atrial Fibrillation Initiating Apixaban, Rivaroxaban, or Warfarin Therapy

Characteristic	Medicare fee-for-service beneficiaries, No. (%)^a^		
	Apixaban (n = 45 950)	Rivaroxaban (n = 45 320)	Warfarin (n = 45 281)
Age, mean (SD), y	77.6 (7.3)	77.6 (7.3)	77.6 (7.3)
Sex			
Female	23 563.0 (51.3)	23 502.2 (51.9)	23 525.7 (52.0)
Male	22 386 (48.7)	21 817.8 (48.1)	21 754.8 (48.0)
Race			
Black	1886.0 (4.1)	1932.6 (4.3)	1897.2 (4.2)
White	41 927.8 (91.2)	41 241.1 (91.0)	41 302.2 (91.2)
Other or missing^b^	2135.8 (4.6)	2146.3 (4.7)	2081.0 (4.6)
Comorbidities			
CHA_2_DS_2_-VASc score	4.2 (1.5)	4.3 (1.5)	4.3 (1.5)
HAS-BLED score	2.3 (0.7)	2.3 (0.7)	2.3 (0.7)
Combined Comorbidity Score	3.2 (2.7)	3.3 (2.7)	3.3 (2.7)
Claims-based frailty index score	0.2 (0.1)	0.2 (0.1)	0.2 (0.1)
Acute kidney failure	6416.3 (14.0)	6611.5 (14.6)	6496.0 (14.3)
Anemia	12 773.8 (27.8)	12 840.7 (28.3)	12 891.7 (28.5)
CHF (inpatient)	9185.3 (20.0)	9480.6 (20.9)	9446.3 (20.9)
CHF (outpatient)	13 828.6 (30.1)	13 805.8 (30.5)	13 953.1 (30.8)
Chronic kidney disease	8765.2 (19.1)	8774.9 (19.4)	8771.5 (19.4)
COPD	12 643.6 (27.5)	12 867.7 (28.4)	12 821.6 (28.3)
Dementia	4032.5 (8.8)	4270.4 (9.4)	4159.0 (9.2)
Diabetes	17 725.7 (38.6)	17 595.6 (38.8)	17 599.8 (38.9)
Frailty	10 195.8 (22.2)	10 643.5 (23.5)	10 496.4 (23.2)
GI bleeding (inpatient)	1698.5 (3.7)	1740.5 (3.8)	1727.8 (3.8)
GI bleeding (outpatient)	2829.7 (6.2)	2808.9 (6.2)	2800.5 (6.2)
Hypertension	38 682.8 (84.2)	38 339.5 (84.6)	38 241.5 (84.5)
Ischemic heart disease	20 793.6 (45.3)	20 495.7 (45.2)	20 724.5 (45.8)
Malignant tumor	8408.2 (18.3)	8363.7 (18.5)	8347.3 (18.4)
Peripheral revascularization	8155.8 (17.7)	8187.1 (18.1)	8156.4 (18.0)
Stroke (inpatient)	3257.1 (7.1)	3430.2 (7.6)	3393.8 (7.5)
Stroke (outpatient)	9436.9 (20.5)	9482.4 (20.9)	9474.2 (20.9)
Transient ischemic attack	2796.0 (6.1)	2832.6 (6.3)	2866.2 (6.3)
Medications			
ACE inhibitors	13 065.4 (28.4)	12 980.0 (28.6)	13 000.6 (28.7)
Angiotensin II receptor blockers	12 427.5 (27.0)	12 147.9 (26.8)	12 183.6 (26.9)
Antiarrhythmic agents	9264.7 (20.2)	8834.2 (19.5)	9217.2 (20.4)
Antiplatelet agent	7078.6 (15.4)	6931.0 (15.3)	7079.1 (15.6)
Benzodiazepines	8190.1 (17.8)	8074.3 (17.8)	8176.4 (18.1)
β-Blockers	31 263.3 (68.0)	30 792.7 (67.9)	30 909.9 (68.3)
Bronchodilators	9159.9 (19.9)	9324.5 (20.6)	9251.7 (20.4)
Corticosteroids, inhaled	10 155.1 (22.1)	10 168.5 (22.4)	10 152.0 (22.4)
Corticosteroids, oral	15 040.9 (32.7)	14 961.6 (33.0)	14 969.8 (33.1)
Diuretics	25 567.7 (55.6)	25 300.5 (55.8)	25 387.8 (56.1)
Histamine_2_-blockers	3744.5 (8.1)	3783.1 (8.3)	3756.8 (8.3)
Insulin	3739.9 (8.1)	3830.8 (8.5)	3788.2 (8.4)
Metformin	7942.2 (17.3)	7896.6 (17.4)	7866.2 (17.4)
NSAIDs	7489.8 (16.3)	7449.7 (16.4)	7455.7 (16.5)
Opioids	17 712.6 (38.5)	17 649.3 (38.9)	17 768.1 (39.2)
Proton pump inhibitors	15 012.4 (32.7)	14 921.7 (32.9)	14 992.8 (33.1)
SSRI or SNRI	9833.5 (21.4)	9879.6 (21.8)	9886.2 (21.8)
Statins	28 711.7 (62.5)	28 211.0 (62.2)	28 247.5 (62.4)
Sulfonylurea	4619.1 (10.1)	4576.2 (10.1)	4607.9 (10.2)
Health care use, mean (SD)			
Hospitalizations	0.8 (1.1)	0.9 (1.1)	0.8 (1.0)
Skilled nursing facility	0.4 (1.2)	0.4 (1.3)	0.4 (1.2)
ED visits	0.7 (1.3)	0.7 (1.3)	0.7 (1.2)
Health care costs, mean (SD), $			
Inpatient	9339.3 (17 351.1)	9717.2 (17 435.0)	10 665.8 (18 931.8)
Outpatient	8941.6 (11 196.9)	9030.7 (11 325.6)	8943.8 (10 841.4)
Pharmacy	3290.9 (9521.3)	3351.9 (9882.9)	3064.7 (8692.2)
Skilled nursing facility	2781.9 (9986.4)	3104.2 (10 673.9)	2966.9 (10 382.6)
Home health	810.6 (2540.3)	852.6 (2634.0)	827.9 (2550.4)
Durable medical equipment	411.0 (2104.1)	415.9 (1965.1)	435.7 (2642.0)
Year of the cohort entry date			
2013	3356.7 (7.3)	3233.2 (7.1)	3591.3 (7.9)
2014	8012.2 (17.4)	7748.0 (17.1)	8404.4 (18.6)
2015	8356.2 (18.2)	8336.7 (18.4)	8222.9 (18.2)
2016	7636.8 (16.6)	7620.0 (16.8)	7449.5 (16.5)
2017	7411.5 (16.1)	7389.3 (16.3)	7142.7 (15.8)
2018	6148.0 (13.4)	6098.9 (13.5)	5788.7 (12.8)
2019^c^	5028.2 (10.9)	4893.9 (10.8)	4681.0 (10.3)
Geographic region			
Northeast	8939.7 (19.5)	8885.0 (19.6)	8736.2 (19.3)
Midwest	11 975.7 (26.1)	11 885.7 (26.2)	11 907.1 (26.3)
South	16 595.0 (36.1)	16 269.3 (35.9)	16 513.8 (36.5)
West	8393.6 (18.3)	8233.6 (18.2)	8070.6 (17.8)
Other or missing	45.6 (0.1)	46.4 (0.1)	52.8 (0.1)

Abbreviations: ACE, angiotensin-converting enzyme; CHA_2_DS_2_-VASc, congestive heart failure, hypertension, age 75 years or older (doubled), diabetes, stroke (doubled), vascular disease, age 65 to 74 years, and sex category (female); CHF, congestive heart failure; COPD, chronic obstructive pulmonary disease; ED, emergency department; GI, gastrointestinal; HAS-BLED, hypertension, abnormal renal/liver function, stroke, bleeding history or predisposition, labile international normalized ratio, elderly, and drugs/alcohol concomitantly; NSAID, nonsteroidal anti-inflammatory drug; SNRI, serotonin-norepinephrine reuptake inhibitor; SSRI, selective serotonin reuptake inhibitor.

^a^ Data are presented as number (percentage) of patients unless otherwise indicated.

^b^ Other races include Asian, North American Native, and other.

^c^ To allow the study cohort to have the 365-day follow-up, the last cohort entry date was December 31, 2019.

### Home Time Lost and Clinical Outcomes

In the weighted population (Table 2), the mean (SD) follow-up was 341 (75) days for apixaban users, 336 (82) days for rivaroxaban users, and 335 (83) days for warfarin users. Compared with apixaban users, rivaroxaban users had a higher risk of more than 14 days of home time lost during 365 days (apixaban vs rivaroxaban: 20.1% vs 21.9%; risk difference, 1.8 [95% CI, 1.5-2.1]) and a higher rate of the composite clinical end point (119.5 vs 140.8 events per 1000 person-years; rate difference, 21.3 [95% CI, 16.4-26.2]). Similarly, warfarin users had a higher risk of home time lost (apixaban vs warfarin: 20.1% vs 23.3%; risk difference, 3.2 [95% CI, 2.9-3.5]) and a higher rate of the composite end point (119.5 vs 148.9 events per 1000 person-years; rate difference, 29.4 [95% CI, 24.5-34.3]). The reductions in home time lost, the composite end point, and major bleeding with apixaban relative to rivaroxaban or warfarin were greater in the frail group than in the nonfrail group (see respective *P* values for heterogeneity in eFigure 1 in Supplement 1).

**Table 2.** Frailty and Association of Apixaban, Rivaroxaban, and Warfarin With Home Time Lost and Clinical Events in Medicare Fee-for-Service Beneficiaries With Atrial Fibrillation

Outcome	Apixaban		Rivaroxaban		Warfarin		Estimate (risk difference^a^ or rate difference^b^) (95% CI)	
	No. of patients	No. (%) of events	No. of patients	No. (%) of events	No. of patients	No. (%) of events	Rivaroxaban vs apixaban	Warfarin vs apixaban
Home time lost >14 d in 12 mo^a^								
Total population	45 950	9233 (20.1)	45 320	9923 (21.9)	45 280	10 546 (23.3)	1.8 (1.5-2.1)	3.2 (2.9-3.5)
Nonfrail	8612	521 (6.0)	8381	578 (6.9)	8441	654 (7.7)	0.8 (0.5-1.2)	1.7 (1.2-2.2)
Prefrail	26 383	4223 (16.0)	26 020	4448 (17.1)	25 979	4901 (18.9)	1.1 (0.7-1.4)	2.9 (2.5-3.2)
Frail	10 247	4444 (43.4)	10 193	4694 (46.0)	10 176	4777 (46.9)	2.7 (1.9-3.4)	3.6 (2.8-4.3)
*P* value for heterogeneity^c^	NA	NA	NA	NA	NA	NA	<.001	<.001
Composite end point^b^								
Total population	45 950	5130 (119.5)	45 320	5884 (140.8)	45 280	6192 (148.9)	21.3 (16.4-26.2)	29.4 (24.5-34.3)
Nonfrail	8612	312 (37.0)	8381	396 (48.7)	8441	427 (52.2)	11.6 (5.3-17.9)	15.1 (8.7-21.5)
Prefrail	26 383	2343 (93.7)	26 020	2729 (111.9)	25 979	2896 (119.3)	18.2 (12.5-23.8)	25.6 (19.8-31.3)
Frail	10 247	2443 (277.3)	10 193	2653 (308.9)	10 176	2749 (322.9)	31.6 (15.5-47.7)	45.5 (29.2-61.9)
*P* value for heterogeneity^c^	NA	NA	NA	NA	NA	NA	.050	<.001

Abbreviation: NA, not applicable.

^a^ Estimates are reported as the risk difference (per 100 persons) of home time lost greater than 14 days.

^b^ Estimates are reported as the rate difference (per 1000 person-years) of the composite end point of ischemic stroke, systemic embolism, major bleeding, or death in 12 months (365 days) after initiating an oral anticoagulant in the propensity score overlap–weighted populations.

^c^ *P* for heterogeneity tests whether the frailty level–specific estimates are different from each other.

### Health Care Costs

In the weighted population (Table 3), compared with apixaban users, rivaroxaban users had a higher mean (SD) 1-year total cost (apixaban vs rivaroxaban: $29 817 [$38 371] vs $30 706 [$37 247]; mean difference, $890 [95% CI, $652-$1127]). The higher total cost for rivaroxaban users was driven by the higher inpatient cost (eFigure 2 in Supplement 1). In comparison, warfarin users had a lower mean (SD) total cost (apixaban vs warfarin: $29 817 [$38 371] vs $28 650 [$36 789]; mean difference, −$1166 [95% CI, −$1396 to −$937]). The lower inpatient cost for apixaban compared with warfarin was offset by higher OAC cost. When the OAC cost was excluded, warfarin users had a higher total mean (SD) cost than apixaban users ($26 848 [$38 334] vs $28 257 [$36 721]; mean difference, $1409 [95% CI, $1177-$1642]). There was no statistically significant heterogeneity in the mean total cost difference between apixaban and either comparator by frailty levels.

**Table 3.** Frailty and Association of Apixaban, Rivaroxaban, and Warfarin With Health Care Cost in Medicare Fee-for-Service Beneficiaries With Atrial Fibrillation

Outcome	Mean (SD)			Mean difference (95% CI)	
	Apixaban	Rivaroxaban	Warfarin	Rivaroxaban vs apixaban	Warfarin vs apixaban
Total cost, $					
Total population	29 817 (38 371)	30 706 (37 247)	28 650 (36 789)	890 (652 to 1127)	−1166 (−1396 to −937)
Nonfrail	18 977 (26 107)	19 470 (25 495)	17 265 (27 619)	494 (108 to 880)	−1711 (−2075 to −1348)
Prefrail	27 901 (34 350)	28 443 (34 493)	26 608 (33 956)	541 (255 to 828)	−1294 (−1571 to −1016)
Frail	44 565 (51 149)	45 542 (46 390)	43 254 (44 636)	977 (350 to 1604)	−1311 (−1922 to −699)
*P* value for heterogeneity^a^	NA	NA	NA	.41	.18
Total cost excluding OAC cost, $					
Total population	26 848 (38 334)	27 915 (37 293)	28 257 (36 721)	1067 (836 to 1298)	1409 (1177 to 1642)
Nonfrail	15 773 (25 966)	16 420 (25 467)	16 927 (27 530)	647 (285 to 1010)	1154 (786 to 1522)
Prefrail	24 901 (34 267)	25 618 (34 480)	26 207 (33 885)	716 (439 to 994)	1306 (1025 to 1587)
Frail	41 898 (51 112)	43 059 (46 411)	42 833 (44 585)	1161 (543 to 1779)	935 (319 to 1551)
*P* value for heterogeneity^a^	NA	NA	NA	.36	.52
OAC cost, $					
Total population	2969 (2694)	2792 (2512)	394 (1021)	−177 (−207 to −147)	−2576 (−2598 to −2554)
Nonfrail	3205 (2812)	3051 (2144)	339 (946)	−154 (−229 to −78)	−2865 (−2921 to −2811)
Prefrail	3001 (2856)	2826 (2699)	402 (1040)	−175 (−215 to −135)	−2599 (−2629 to −2570)
Frail	2667 (2353)	2484 (2405)	422 (1028)	−184 (−237 to −130)	−2245 (−2285 to −2206)
*P* value for heterogeneity^a^	NA	NA	NA	.82	<.001

Abbreviations: NA, not applicable; OAC, oral anticoagulant.

^a^ *P* for heterogeneity tests whether the frailty level–specific estimates are different from each other. The table displays the mean cost in 12 months (365 days) after initiating an oral anticoagulant in the propensity score overlap–weighted populations.

### Sensitivity Analysis

We conducted sensitivity analyses excluding beneficiaries with stroke and major bleeding in the 60 days before the index date (eFigure 3 in Supplement 1 for home time lost and clinical events and eFigure 4 in Supplement 1 for health care costs), excluding those with SNF stay during the baseline assessment period (eFigures 5 and 6 in Supplement 1), and excluding data from 2020 (eFigures 7 and 8 in Supplement 1). These analyses showed similar findings to our main analysis.

## Discussion

We used US Medicare claims data to emulate a 3-group head-to-head target trial that compared warfarin, rivaroxaban, and apixaban in patients with AF. We found that apixaban was associated with lower home time lost and fewer clinical events than rivaroxaban and warfarin, with greater reductions among patients with frailty. The total health care cost difference varied by frailty level. Warfarin was associated with the lowest total cost, followed by apixaban and then rivaroxaban, regardless of frailty level. Excluding the OAC cost, apixaban was associated with the lowest cost compared with other OACs. Our results support apixaban as the preferred OAC for older adults with AF, particularly for older adults with frailty.

To our knowledge, this is the first study to investigate the association of frailty with home time, a patient-centered outcome, and with health care costs. We used a trial emulation framework with high scientific rigor,^26,27^ yet the evidence was generated based on a nationally representative population enriched with patients with frailty, who are severely underrepresented in traditional trials.^4,5,6,7^ Prior studies investigating frailty and clinical outcomes in patients with AF reported that apixaban was associated with lower rates of death, ischemic stroke, or major bleeding than warfarin across all frailty levels.^15,52^ The pairwise comparative design of a DOAC vs warfarin did not allow direct multigroup comparisons, a gap addressed by our 3-group head-to-head trial emulation.^44^ Moreover, our study provides evidence from more recent data to translate the associations between OACs and clinical outcomes into patient-centered outcomes and health care costs.

Home time is a highly valued state from the patient’s perspective^17,18,20,21,22,23^ that incorporates recurrent hospitalizations and SNF stays due to clinical outcomes, including stroke, bleeding, and mortality. In a hospital-based registry study of 12 552 patients with AF and ischemic stroke conducted in the pre-DOAC era (2009-2011), patients treated with warfarin at discharge had 47.6 more days at home during 2 years than untreated patients.^19^ We found that apixaban was associated with not only a reduction in clinical events but also a smaller risk of home time lost greater than 14 days during 365 days, a threshold that correlated with worsening mobility impairment, depression, and difficulty in self-care.^18^ The association favoring apixaban was stronger among beneficiaries with frailty, reflecting the higher background risks and disease burden in patients with frailty.^52,53^

Comparative analysis of health care costs associated with different treatments can provide community-based evidence to support coverage decisions as recommended by the 21st Century Cures Act and the Prescription Drug User Fee Act.^54,55^ To date, there is little community-based evidence comparing health care expenditure by OACs in the US. In a 2012-2015 US commercial claims database study, the use of any DOAC was associated with lower medical and total costs than warfarin, but no individual DOAC comparison was reported.^56^ Another study using a 2016-2018 US commercial claims database found lower 18-month medical costs among rivaroxaban users compared with apixaban users.^57^ In our analysis of Medicare beneficiaries, the 12-month total cost was lowest in warfarin users (mainly driven by lower OAC cost) and highest in rivaroxaban users (mainly driven by increased inpatient cost), regardless of the frailty level. When OAC cost was excluded, the total cost was lowest in apixaban users and highest in rivaroxaban users.

### Strengths and Limitations

The main strengths of our study include the generalizability of our results based on a nationally representative large database of older adults in the US; a rigorous trial emulation analytical framework^26,27^ with new-user, active comparator design, and prespecified eligibility criteria; PS overlap weighting that minimizes confounding and mimics a head-to-head clinical trial of 3 OACs; and sensitivity analyses to test the robustness of our findings. It is important to consider competing risk due to death in the elderly population with frailty. Both of our primary outcomes inherently accounted for the impact of death in their outcome ascertainment. Home time loss due to death is directly measured in our equation, and when calculating the cost outcomes, we used only the observed cost before death for those who died before 365 days. Therefore, the increased cost before death and zero cost after death are both reflected in our study findings.

Our study also has several limitations. First, clinical variables (eg, laboratory test results) and the use of over-the-counter aspirin or nonsteroidal anti-inflammatory drugs that could influence the effectiveness and safety of OACs were unavailable in administrative claims data. An imbalance in such variables by the treatment groups could lead to unmeasured confounding. Second, we truncated follow-up at 365 days for home time and health care cost assessment because adherence to OAC may decline beyond this period.^58^ This truncation may help reduce misclassification of the OAC exposure, but this study did not evaluate long-term home time, clinical events, and health care costs. Third, the CFI may not be readily available to clinicians at the point of care. Nonetheless, the CFI threshold of 0.25 approximates a specific score of the following clinical frailty assessments: Clinical Frailty Scale stage 5,^59^ FRAIL scale (fatigue, resistance, ambulation, illnesses, and loss of weight) score of 2 points,^60^ frailty phenotype of 2 points,^61^ and a comprehensive geriatric assessment–based frailty index of 0.25.^62,63^ Fourth, our outcome ascertainment period includes 2020 in which the COVID-19 pandemic may have impacted routine care for patients with AF.^64^ However, our sensitivity analysis, excluding the year of 2020, showed consistent results. Fifth, we truncated follow-up at 365 days to minimize OAC misclassification due to nonadherence. Therefore, we did not assess the long-term impact of OAC beyond 365 days after initiation. Sixth, our results are based on Medicare beneficiaries older than 65 years. The findings may not be generalizable to patients with no or other insurance coverage or younger populations. In addition, using PS overlap weighting, our inference was drawn based on OAC users with similar propensity to initiate any one of the study drugs (ie, with PS ranges that overlap with one another). The PS overlap population may represent the patients for whom the clinical equipoise is present, but the findings may not be generalizable to those with extreme PS values. Seventh, although we investigated health care cost as an outcome, a cost-effectiveness analysis is beyond the scope of this study. However, our findings may be useful for a future cost-effectiveness analysis study.

## Conclusions

This cohort study, based on a nationwide US claims database, provides evidence to guide OAC choice to maximize home time and reduce clinical events and health care spending in older adults with AF based on the level of frailty. Apixaban was associated with increased home time and lower rates of clinical events than rivaroxaban and warfarin, with greater benefit seen in those with frailty. Regardless of frailty levels, apixaban was associated with lower total cost compared with rivaroxaban but higher total cost compared with warfarin because of the OAC cost.

## Notes

Supplement 1. **eAppendix.** Study Protocol: Definition of the Study Variables **eTable 1.** Characteristics of the Overall Population of Medicare Fee-for-Service Beneficiaries With Atrial Fibrillation **eTable 2.** Characteristics of the Non-Frail Population of Medicare Fee-for-Service Beneficiaries With Atrial Fibrillation **eTable 3.** Characteristics of the Pre-Frail Population of Medicare Fee-for-Service Beneficiaries With Atrial Fibrillation **eTable 4.** Characteristics of the Frail Population of Medicare Fee-for-Service Beneficiaries With Atrial Fibrillation **eFigure 1.** Frailty and Association of Apixaban, Rivaroxaban, and Warfarin With Home Time Lost and Clinical Events in Medicare Fee-for-Service Beneficiaries With Atrial Fibrillation **eFigure 2.** Frailty and Association of Apixaban, Rivaroxaban, and Warfarin With Health Care Cost in Medicare Fee-for-Service Beneficiaries With Atrial Fibrillation **eFigure 3.** Frailty and Association of Apixaban, Rivaroxaban, and Warfarin With Home Time Loss and Clinical Events in Medicare Fee-for-Service Beneficiaries With Atrial Fibrillation – Sensitivity Analysis 1 to Exclude Ischemic Stroke and Major Bleeding in the 60 Days Prior to the Index Date **eFigure 4.** Frailty and Association of Apixaban, Rivaroxaban, and Warfarin With Health Care Cost in Medicare Fee-for-Service Beneficiaries With Atrial Fibrillation – Sensitivity Analysis 1 to Exclude Ischemic Stroke and Major Bleeding in the 60 Days Prior to the Index Date **eFigure 5.** Frailty and Association of Apixaban, Rivaroxaban, and Warfarin With Home Time Loss and Clinical Events in Medicare Fee-for-Service Beneficiaries With Atrial Fibrillation – Sensitivity Analysis 2 to Exclude Beneficiaries with Skilled Nursing Facility Stay in the 365 Days Prior to the Index Date **eFigure 6.** Frailty and Association of Apixaban, Rivaroxaban, and Warfarin With Health Care Cost in Medicare Fee-for-Service Beneficiaries With Atrial Fibrillation – Sensitivity Analysis 2 to Exclude Beneficiaries with Skilled Nursing Facility Stay in the 365 Days Prior to the Index Date **eFigure 7.** Frailty and Association of Apixaban, Rivaroxaban, and Warfarin With Home Time Loss and Clinical Events in Medicare Fee-for-Service Beneficiaries With Atrial Fibrillation – Sensitivity Analysis 2 to Exclude Data from 2020 **eFigure 8.** Frailty and Association of Apixaban, Rivaroxaban, and Warfarin With Health Care Cost in Medicare Fee-for-Service Beneficiaries With Atrial Fibrillation – Sensitivity Analysis 2 to Exclude Data from 2020

Supplement 2.
Data Sharing Statement
