# Supplementary material for: Frailty, Home Time, and Health Care Costs in Older Adults With Atrial Fibrillation Receiving Oral Anticoagulants
Source: JAMA Netw Open. 2023 Nov 9;6(11):e2342264. doi: 10.1001/jamanetworkopen.2023.42264 (PMC10636636; doi:10.1001/jamanetworkopen.2023.42264)
Supplement: Supplement 1. — eAppendix. Study Protocol: Definition of the Study Variables eTable 1. Characteristics of the Overall Population of Medicare Fee-for-Service Beneficiaries With Atrial Fibrillation eTable 2. Characteristics of the Non-Frail Population of Medicare Fee-for-Service Beneficiaries With Atrial Fibrillation eTable 3. Characteristics of the Pre-Frail Population of Medicare Fee-for-Service Beneficiaries With Atrial Fibrillation eTable 4. Characteristics of the Frail Population of Medicare Fee-for-Service Beneficiaries With Atrial Fibrillation eFigure 1. Frailty and Association of Apixaban, Rivaroxaban, and Warfarin With Home Time Lost and Clinical Events in Medicare Fee-for-Service Beneficiaries With Atrial Fibrillation eFigure 2. Frailty and Association of Apixaban, Rivaroxaban, and Warfarin With Health Care Cost in Medicare Fee-for-Service Beneficiaries With Atrial Fibrillation eFigure 3. Frailty and Association of Apixaban, Rivaroxaban, and Warfarin With Home Time Loss and Clinical Events in Medicare Fee-for-Service Beneficiaries With Atrial Fibrillation – Sensitivity Analysis 1 to Exclude Ischemic Stroke and Major Bleeding in the 60 Days Prior to the Index Date eFigure 4. Frailty and Association of Apixaban, Rivaroxaban, and Warfarin With Health Care Cost in Medicare Fee-for-Service Beneficiaries With Atrial Fibrillation – Sensitivity Analysis 1 to Exclude Ischemic Stroke and Major Bleeding in the 60 Days Prior to the Index Date eFigure 5. Frailty and Association of Apixaban, Rivaroxaban, and Warfarin With Home Time Loss and Clinical Events in Medicare Fee-for-Service Beneficiaries With Atrial Fibrillation – Sensitivity Analysis 2 to Exclude Beneficiaries with Skilled Nursing Facility Stay in the 365 Days Prior to the Index Date eFigure 6. Frailty and Association of Apixaban, Rivaroxaban, and Warfarin With Health Care Cost in Medicare Fee-for-Service Beneficiaries With Atrial Fibrillation – Sensitivity Analysis 2 to Exclude Beneficiaries with Skilled Nursing Facility Stay [file jamanetwopen-e2342264-s001.pdf]

Supplementary Online Content

Lin KJ, Singer DE, Ko D, et al. Frailty, home time, and health care costs in older adults with atrial fibrillation receiving oral anticoagulants. *JAMA Netw Open*. 2023;6(11):e2342264. doi:10.1001/jamanetworkopen.2023.42264

eAppendix. Study Protocol: Definition of the Study Variables
eTable 1. Characteristics of the Overall Population of Medicare Fee-for-Service Beneficiaries With Atrial Fibrillation
eTable 2. Characteristics of the Non-Frail Population of Medicare Fee-for-Service Beneficiaries With Atrial Fibrillation
eTable 3. Characteristics of the Pre-Frail Population of Medicare Fee-for-Service Beneficiaries With Atrial Fibrillation
eTable 4. Characteristics of the Frail Population of Medicare Fee-for-Service Beneficiaries With Atrial Fibrillation
eFigure 1. Frailty and Association of Apixaban, Rivaroxaban, and Warfarin With Home Time Lost and Clinical Events in Medicare Fee-for-Service Beneficiaries With Atrial Fibrillation
eFigure 2. Frailty and Association of Apixaban, Rivaroxaban, and Warfarin With Health Care Cost in Medicare Fee-for-Service Beneficiaries With Atrial Fibrillation
eFigure 3. Frailty and Association of Apixaban, Rivaroxaban, and Warfarin With Home Time Loss and Clinical Events in Medicare Fee-for-Service Beneficiaries With Atrial Fibrillation – Sensitivity Analysis 1 to Exclude Ischemic Stroke and Major Bleeding in the 60 Days Prior to the Index Date
eFigure 4. Frailty and Association of Apixaban, Rivaroxaban, and Warfarin With Health Care Cost in Medicare Fee-for-Service Beneficiaries With Atrial Fibrillation – Sensitivity Analysis 1 to Exclude Ischemic Stroke and Major Bleeding in the 60 Days Prior to the Index Date
eFigure 5. Frailty and Association of Apixaban, Rivaroxaban, and Warfarin With Home Time Loss and Clinical Events in Medicare Fee-for-Service Beneficiaries With Atrial Fibrillation – Sensitivity Analysis 2 to Exclude Beneficiaries with Skilled Nursing Facility Stay in the 365 Days Prior to the Index Date
eFigure 6. Frailty and Association of Apixaban, Rivaroxaban, and Warfarin With Health Care Cost in Medicare Fee-for-Service Beneficiaries With Atrial Fibrillation – Sensitivity Analysis 2 to Exclude Beneficiaries with Skilled Nursing Facility Stay in the 365 Days Prior to the Index Date
eFigure 7. Frailty and Association of Apixaban, Rivaroxaban, and Warfarin With Home Time Loss and Clinical Events in Medicare Fee-for-Service Beneficiaries With Atrial Fibrillation – Sensitivity Analysis 2 to Exclude Data from 2020
eFigure 8. Frailty and Association of Apixaban, Rivaroxaban, and Warfarin With Health Care Cost in Medicare Fee-for-Service Beneficiaries With Atrial Fibrillation – Sensitivity Analysis 2 to Exclude Data From 2020

This supplementary material has been provided by the authors to give readers additional information about their work.

© 2023 Lin KJ et al. *JAMA Network Open.*

eAppendix. Study Protocol: Definition of the Study Variables

Supplementary Methods:

We applied propensity score-based overlap weighting to achieve balance in baseline characteristics among patients who were treated with warfarin, rivaroxaban, and apixaban (R package PSweight was used). This procedure estimates the average treatment effect in a clinically relevant target population who could potentially receive any of the 3 oral anticoagulants (OACs) of interest (JAMA. 2020 Jun 16;323(23):2417-2418. PMID: 32369102). To estimate propensity scores, we fitted a multinomial logistic regression that modeled the receipt of each OAC as a function of the patient-level and hospital-level characteristics and calendar year to control for temporal trends, as listed in **eTable 1**. We calculated the predicted probabilities of receiving each of the 3 OACs for each patient.

Overlap weighting offers advantages over other propensity score methods. In propensity score matching, unmatched patients are excluded, which may result in a different target population and loss of precision. Moreover, matching 3 OAC groups is challenging. In inverse probability of treatment weighting, patients get the weight of the inverse of the probability of receiving the treatment that they received: for the treated, $w = 1/e(x)$, and for the untreated, $w = 1/1 - e(x)$, where $e(x)$ is the probability of receiving the treatment conditional on baseline characteristics (x). Patients in the extreme end of the propensity score distribution get very large weights. Therefore, the effect estimates are sensitive to large weights and have a wide confidence interval. In overlap weighting, patients get the weight of the probability of receiving the opposite treatment: for the group treated with drug A, $w = 1 - e(x)$, and for the group with drug B, $w = e(x)$. Patients who are almost always treated with drug A and those who are almost always treated with drug B get a weight close to zero, minimizing the influence of those in the extreme end of the propensity score distribution. As such, overlap weighting can achieve better covariate balance and offer greater precision of the treatment effect estimate. In the setting of multiple treatments, the overlap weight for the treatment group j $(j = 1, \ldots, J)$ is $w = \left\{\sum_{k=1}^{J} 1/e_k(x)\right\}^{-1}/e_j(x)$. Using overlap weighting, we were able to achieve adequate covariate balance, assessed by a standard mean difference <0.1. However, when we attempted inverse probability of treatment weighting, we were unable to achieve covariate balance.

Exposure Definitions

Exposure	NDC Generic Name
Apixaban	APIXABAN
Rivaroxaban	RIVAROXABAN
Warfarin	WARFARIN SODIUM

Outcome Definitions

Outcome	Codes/Definition
Major Bleeding	**Primary position, inpatient ICD-9 Diagnosis Code is any of**: 336.1, 363.61, 363.72, 376.32, 430, 432, 432.9, 568.81, 719.1, 719.10, 719.15, 719.18, 852, 852.0, 852.00, 852.04, 852.05, 852.10, 852.11, 852.12, 852.16, 852.20, 852.21, 852.22, 852.23, 852.25, 852.29, 852.3, 852.31, 852.32, 852.35, 852.36, 852.40, 852.43, 852.5, 852.51, 852.52, 852.53, 852.54, 852.59, 853.0, 853.00, 853.04, 853.05, 853.09, 853.10, 853.11, 853.13, 853.19, 866.02,

© 2023 Lin KJ et al. *JAMA Network Open.*

	866.11, 866.12, 363.62, 377.42, 379.23, 423.0, 431, 432.0, 432.1, 719.11, 719.12, 719.13, 719.14, 719.16, 719.17, 719.19, 729.92, 852.01, 852.02, 852.03, 852.06, 852.09, 852.1, 852.13, 852.14, 852.15, 852.19, 852.2, 852.24, 852.26, 852.30, 852.33, 852.34, 852.39, 852.4, 852.41, 852.42, 852.44, 852.45, 852.46, 852.49, 852.50, 852.55, 852.56, 853, 853.01, 853.02, 853.03, 853.06, 853.1, 853.12, 853.14, 853.15, 853.16, 866.01<br><br>**Primary position, inpatient ICD-10 Diagnosis Code is any of**: G95.19, H05.23, H05.231, H05.232, H05.239, H31.303, H31.311, H31.312, H31.319, H31.412, H31.419, H43.11, H43.12, H47.029, I31.2, I60.0, I60.01, I60.10, I60.11, I60.3, I60.31, I60.5, I60.51, I60.52, I60.6, I60.8, I61, I61.4, I61.8, I62.1, K66.1, M25.011, M25.012, M25.02, M25.022, M25.03, M25.032, M25.04, M25.041, M25.042, M25.049, M25.05, M25.059, M25.06, M25.061, M25.062, M25.069, M25.071, M25.074, M25.076, M25.08, S06.4X0D, S06.4X0S, S06.4X1, S06.4X1S, S06.4X2, S06.4X2D, S06.4X3, S06.4X3A, S06.4X4, S06.4X4A, S06.4X5S, S06.4X6, S06.4X6A, S06.4X6D, S06.4X6S, S06.4X7, S06.4X7A, S06.4X8A, S06.5X, S06.5X0, S06.5X0S, S06.5X1, S06.5X1A, S06.5X1S, S06.5X2, S06.5X2D, S06.5X2S, S06.5X3, S06.5X3A, S06.5X3D, S06.5X3S, S06.5X4, S06.5X4A, S06.5X4D, S06.5X5D, S06.5X6A, S06.5X6S, S06.5X8, S06.5X8A, S06.5X9A, S06.6, S06.6X0, S06.6X0D, S06.6X1, S06.6X1A, S06.6X1S, S06.6X2, S06.6X2D, S06.6X2S, S06.6X3, S06.6X3A, S06.6X3D, S06.6X4S, S06.6X5A, S06.6X5S, S06.6X6D, S06.6X7A, S06.6X8, S06.6X8A, S06.6X9S, S26.00, S26.00XS, S26.01, S26.01XS, S26.020D, S26.021, S26.021D, S26.021S, S26.022, S26.022A, S26.022S, S26.09XA, S26.09XD, S37.01, S37.011S, S37.012D, S37.021, S37.021A, S37.021D, S37.022, S37.029D, S37.029S, S37.03, S37.031, S37.031A, S37.031S, S37.032S, S37.039D, S37.041S, S37.042, S37.042A, S37.049A, S37.051A, S37.051D, S37.051S, S37.052D, S37.052S, S37.059, S37.059D, S37.06, S37.061, S37.061D, S37.062, S37.062A, S37.062D, S37.062S, S37.069, S37.069A, S37.069S, H05.233, H31.30, H31.301, H31.302, H31.309, H31.31, H31.313, H31.41, H31.411, H31.413, H43.1, H43.10, H43.13, H47.02, H47.021, H47.022, H47.023, I23.0, I60, I60.00, I60.02, I60.1, I60.12, I60.2, I60.30, I60.32, I60.4, I60.50, I60.7, I60.9, I61.0, I61.1, I61.2, I61.3, I61.5, I61.6, I61.9, I62, I62.0, I62.00, I62.01, I62.02, I62.03, I62.9, M25.0, M25.00, M25.01, M25.019, M25.021, M25.029, M25.031, M25.039, M25.051, M25.052, M25.07, M25.072, M25.073, M25.075, S06.4, S06.4X, S06.4X0, S06.4X0A, S06.4X1A, S06.4X1D, S06.4X2A, S06.4X2S, S06.4X3D, S06.4X3S, S06.4X4D, S06.4X4S, S06.4X5, S06.4X5A, S06.4X5D, S06.4X8, S06.4X9, S06.4X9A, S06.4X9D, S06.4X9S, S06.5, S06.5X0A, S06.5X0D, S06.5X1D, S06.5X2A, S06.5X4S, S06.5X5, S06.5X5A, S06.5X5S, S06.5X6, S06.5X6D, S06.5X7,

© 2023 Lin KJ et al. *JAMA Network Open.*

	S06.5X7A, S06.5X9, S06.5X9D, S06.5X9S, S06.6X, S06.6X0A, S06.6X0S, S06.6X1D, S06.6X2A, S06.6X3S, S06.6X4, S06.6X4A, S06.6X4D, S06.6X5, S06.6X5D, S06.6X6, S06.6X6A, S06.6X6S, S06.6X7, S06.6X9, S06.6X9A, S06.6X9D, S26.0, S26.00XA, S26.00XD, S26.01XA, S26.01XD, S26.02, S26.020, S26.020A, S26.020S, S26.021A, S26.022D, S26.09, S26.09XS, S37.011, S37.011A, S37.011D, S37.012, S37.012A, S37.012S, S37.019, S37.019A, S37.019D, S37.019S, S37.02, S37.021S, S37.022A, S37.022D, S37.022S, S37.029, S37.029A, S37.031D, S37.032, S37.032A, S37.032D, S37.039, S37.039A, S37.039S, S37.04, S37.041, S37.041A, S37.041D, S37.042D, S37.042S, S37.049, S37.049D, S37.049S, S37.05, S37.051, S37.052, S37.052A, S37.059A, S37.059S, S37.061A, S37.061S, S37.069D, M79.81<br><br>**ICD-9 Procedure Code (any position) is any of:** 99.04, 99.05, 99.03, 99.06 **with Primary ICD-9 Diagnosis Code is any of**: 285.1, 455.2, 455.5, 455.6, 455.7, 455.8, 456.0, 530.1, 530.11, 530.13, 530.19, 530.7, 531.3, 531.40, 531.5, 531.60, 531.61, 532.20, 532.3, 532.4, 532.41, 532.5, 532.6, 532.61, 532.9, 533.0, 533.00, 533.01, 533.20, 533.3, 533.40, 533.6, 533.60, 533.61, 533.7, 534.01, 534.2, 534.3, 534.4, 534.40, 534.41, 534.5, 534.6, 534.9, 535.00, 535.11, 535.20, 535.30, 535.41, 535.50, 535.61, 562.00, 562.02, 562.03, 562.10, 562.11, 562.12, 562.13, 569.3, 569.85, 578.1, 599.70, 599.71, 623.6, 623.8, 626.6, 626.8, 784.7, 784.8, 786.3, 786.30, 786.39, 280.0, 455.1, 455.4, 455.9, 456.20, 459.0, 530.10, 530.12, 530.82, 531.0, 531.00, 531.01, 531.1, 531.2, 531.20, 531.21, 531.4, 531.41, 531.6, 531.7, 531.9, 532.0, 532.00, 532.01, 532.1, 532.2, 532.21, 532.40, 532.60, 532.7, 533.1, 533.2, 533.21, 533.4, 533.41, 533.5, 533.9, 534.0, 534.00, 534.1, 534.20, 534.21, 534.60, 534.61, 534.7, 535.01, 535.10, 535.21, 535.31, 535.40, 535.51, 535.60, 537.83, 562.01, 578.0, 578.9, 593.81, 786.31<br><br>**HCPCS Procedure Code (any position) is any of**: P9010, P9016, P9017, P9019, P9022, P9032, P9036, P9038, P9039, P9051, P9052, P9054, P9055, P9056, P9058, P9059, P9060, P9011, P9020, P9021, P9023, P9031, P9033, P9034, P9035, P9037, P9040, P9044, P9053, P9057, P9070, P9071 **with Primary ICD-9 Diagnosis Code is any of:** 285.1, 455.2, 455.5, 455.6, 455.7, 455.8, 456.0, 530.1, 530.11, 530.13, 530.19, 530.7, 531.3, 531.40, 531.5, 531.60, 531.61, 532.20, 532.3, 532.4, 532.41, 532.5, 532.6, 532.61, 532.9, 533.0, 533.00, 533.01, 533.20, 533.3, 533.40, 533.6, 533.60, 533.61, 533.7, 534.01, 534.2, 534.3, 534.4, 534.40, 534.41, 534.5, 534.6, 534.9, 535.00, 535.11, 535.20, 535.30, 535.41, 535.50, 535.61, 562.00, 562.02, 562.03, 562.10, 562.11, 562.12, 562.13, 569.3, 569.85, 578.1, 599.70, 599.71, 623.6,

© 2023 Lin KJ et al. *JAMA Network Open.*

	623.8, 626.6, 626.8, 784.7, 784.8, 786.3, 786.30, 786.39, 280.0, 455.1, 455.4, 455.9, 456.20, 459.0, 530.10, 530.12, 530.82, 531.0, 531.00, 531.01, 531.1, 531.2, 531.20, 531.21, 531.4, 531.41, 531.6, 531.7, 531.9, 532.0, 532.00, 532.01, 532.1, 532.2, 532.21, 532.40, 532.60, 532.7, 533.1, 533.2, 533.21, 533.4, 533.41, 533.5, 533.9, 534.0, 534.00, 534.1, 534.20, 534.21, 534.60, 534.61, 534.7, 535.01, 535.10, 535.21, 535.31, 535.40, 535.51, 535.60, 537.83, 562.01, 578.0, 578.9, 593.81, 786.31<br><br>**Revenue Center Code is any of:** 0387, 0391, 0381, 0382, 0383, 0384 **with Primary ICD-9 Diagnosis Code is any of**: 285.1, 455.2, 455.5, 455.6, 455.7, 455.8, 456.0, 530.1, 530.11, 530.13, 530.19, 530.7, 531.3, 531.40, 531.5, 531.60, 531.61, 532.20, 532.3, 532.4, 532.41, 532.5, 532.6, 532.61, 532.9, 533.0, 533.00, 533.01, 533.20, 533.3, 533.40, 533.6, 533.60, 533.61, 533.7, 534.01, 534.2, 534.3, 534.4, 534.40, 534.41, 534.5, 534.6, 534.9, 535.00, 535.11, 535.20, 535.30, 535.41, 535.50, 535.61, 562.00, 562.02, 562.03, 562.10, 562.11, 562.12, 562.13, 569.3, 569.85, 578.1, 599.70, 599.71, 623.6, 623.8, 626.6, 626.8, 784.7, 784.8, 786.3, 786.30, 786.39, 280.0, 455.1, 455.4, 455.9, 456.20, 459.0, 530.10, 530.12, 530.82, 531.0, 531.00, 531.01, 531.1, 531.2, 531.20, 531.21, 531.4, 531.41, 531.6, 531.7, 531.9, 532.0, 532.00, 532.01, 532.1, 532.2, 532.21, 532.40, 532.60, 532.7, 533.1, 533.2, 533.21, 533.4, 533.41, 533.5, 533.9, 534.0, 534.00, 534.1, 534.20, 534.21, 534.60, 534.61, 534.7, 535.01, 535.10, 535.21, 535.31, 535.40, 535.51, 535.60, 537.83, 562.01, 578.0, 578.9, 593.81, 786.31<br><br>**ICD-10 Procedure Code (any position) is any of**: 30230P1, 30230T1, 30233H1, 30233M1, 30233R1, 30233W1, 30240K1, 30240W1, 30243H1, 30243M1, 30243N1, 30243R1, 30243T1, 30243V1, 30230H1, 30230K1, 30230L1, 30230M1, 30230N1, 30230R1, 30230V1, 30230W1, 30233K1, 30233L1, 30233N1, 30233P1, 30233T1, 30233V1, 30240H1, 30240L1, 30240M1, 30240N1, 30240P1, 30240R1, 30240T1, 30240V1, 30243K1, 30243L1, 30243P1, 30243W1 **with Primary ICD-10 Diagnosis Code is any of:** D50.0, D62, I85.11, K20, K20.8, K21.0, K22.11, K22.6, K25.0, K25.4, K25.6, K26.1, K26.4, K26.5, K26.6, K27.2, K27.5, K27.6, K28.0, K28.4, K28.5, K29.01, K29.21, K29.41, K31.811, K55.21, K57.01, K57.21, K57.51, K57.53, K57.93, K64.0, K64.5, K64.8, K64.9, K92.0, N89.7, N92.1, N93.9, R04.0, R04.1, R04.2, R31.0, R31.9, R58, I85.01, K20.0, K20.9, K25.1, K25.2, K25.5, K26.0, K26.2, K27.0, K27.1, K27.4, K28.1, K28.2, K28.6, K29.31, K29.51, K29.61, K29.71, K29.81, K29.91, K57.11, K57.31, K57.41, K57.81, K57.91, K62.5, K64.1, K64.2, K64.3, K64.4, K92.1, K92.2, N89.8, N93.8

© 2023 Lin KJ et al. *JAMA Network Open.*

	**HCPCS Procedure Code (any position) is any of**: P9010, P9016, P9017, P9019, P9022, P9032, P9036, P9038, P9039, P9051, P9052, P9054, P9055, P9056, P9058, P9059, P9060, P9011, P9020, P9021, P9023, P9031, P9033, P9034, P9035, P9037, P9040, P9044, P9053, P9057, P9070, P9071 **with Primary ICD-10 Diagnosis Code is any of:** D50.0, D62, I85.11, K20, K20.8, K21.0, K22.11, K22.6, K25.0, K25.4, K25.6, K26.1, K26.4, K26.5, K26.6, K27.2, K27.5, K27.6, K28.0, K28.4, K28.5, K29.01, K29.21, K29.41, K31.811, K55.21, K57.01, K57.21, K57.51, K57.53, K57.93, K64.0, K64.5, K64.8, K64.9, K92.0, N89.7, N92.1, N93.9, R04.0, R04.1, R04.2, R31.0, R31.9, R58, I85.01, K20.0, K20.9, K25.1, K25.2, K25.5, K26.0, K26.2, K27.0, K27.1, K27.4, K28.1, K28.2, K28.6, K29.31, K29.51, K29.61, K29.71, K29.81, K29.91, K57.11, K57.31, K57.41, K57.81, K57.91, K62.5, K64.1, K64.2, K64.3, K64.4, K92.1, K92.2, N89.8, N93.8<br><br>**Revenue Center Code is any of:** 0387, 0391, 0381, 0382, 0383, 0384 **with Primary ICD-10 Diagnosis Code is any of**: D50.0, D62, I85.11, K20, K20.8, K21.0, K22.11, K22.6, K25.0, K25.4, K25.6, K26.1, K26.4, K26.5, K26.6, K27.2, K27.5, K27.6, K28.0, K28.4, K28.5, K29.01, K29.21, K29.41, K31.811, K55.21, K57.01, K57.21, K57.51, K57.53, K57.93, K64.0, K64.5, K64.8, K64.9, K92.0, N89.7, N92.1, N93.9, R04.0, R04.1, R04.2, R31.0, R31.9, R58, I85.01, K20.0, K20.9, K25.1, K25.2, K25.5, K26.0, K26.2, K27.0, K27.1, K27.4, K28.1, K28.2, K28.6, K29.31, K29.51, K29.61, K29.71, K29.81, K29.91, K57.11, K57.31, K57.41, K57.81, K57.91, K62.5, K64.1, K64.2, K64.3, K64.4, K92.1, K92.2, N89.8, N93.8
Ischemic Stroke	**Primary position, inpatient** ICD-9 Diagnosis Code is any of: 433.01, 433.11, 433.21, 433.31, 434.01, 434.11, 362.31, 362.32, 362.33, 362.34, 433.81, 433.91, 436<br>Primary ICD-10 Diagnosis Code is any of: H34.0, H34.00, H34.01, H34.02, H34.03, H34.1, H34.10, H34.11, H34.13, H34.2, H34.21, H34.213, H34.23, H34.231, H34.239, I63.011, I63.012, I63.031, I63.112, I63.12, I63.132, I63.139, I63.19, I63.2, I63.21, I63.212, I63.213, I63.219, I63.22, I63.231, I63.232, I63.233, I63.239, I63.29, I63.30, I63.31, I63.311, I63.32, I63.322, I63.329, I63.33, I63.339, I63.34, I63.341, I63.342, I63.349, I63.39, I63.40, I63.411, I63.419, I63.422, I63.429, I63.431, I63.433, I63.49, I63.50, I63.51, I63.52, I63.523, I63.529, I63.531, I63.533, I63.541, I63.542, I63.543, I63.8, I63.9, H34.12, H34.211, H34.212, H34.219, H34.232, H34.233, I63, I63.0, I63.00, I63.01, I63.013, I63.019, I63.02, I63.03, I63.032, I63.033, I63.039, I63.09, I63.1, I63.10, I63.11, I63.111, I63.113, I63.119, I63.13, I63.131, I63.133, I63.20, I63.211, I63.23, I63.3, I63.312, I63.313, I63.319, I63.321, I63.323, I63.331, I63.332, I63.333, I63.343, I63.4, I63.41, I63.412, I63.413, I63.42,

© 2023 Lin KJ et al. *JAMA Network Open.*

	I63.421, I63.423, I63.43, I63.432, I63.439, I63.44, I63.441, I63.442, I63.443, I63.449, I63.5, I63.511, I63.512, I63.513, I63.519, I63.521, I63.522, I63.53, I63.532, I63.539, I63.54, I63.549, I63.59, I63.6
All-Cause Mortality	**Death** recorded by **Master Beneficiary Summary File** or **Inpatient** Discharge Status Code is any of: 20, 40, 41, 42
Major GI Bleeding	ICD-9 Procedure Code (any position) is any of: 99.04, 99.05, 99.03, 99.06 with Primary ICD-9 Diagnosis Code is any of: 455.2, 455.5, 455.6, 455.7, 455.8, 456.0, 530.1, 530.11, 530.13, 530.19, 530.7, 531.3, 531.40, 531.5, 531.60, 531.61, 532.20, 532.3, 532.4, 532.41, 532.5, 532.6, 532.61, 532.9, 533.0, 533.00, 533.01, 533.20, 533.3, 533.40, 533.6, 533.60, 533.61, 533.7, 534.01, 534.2, 534.3, 534.4, 534.40, 534.41, 534.5, 534.6, 534.9, 535.00, 535.11, 535.20, 535.30, 535.41, 535.50, 535.61, 562.00, 562.02, 562.03, 562.10, 562.11, 562.12, 562.13, 569.3, 569.85, 578.1, 455.1, 455.4, 455.9, 456.20, 530.10, 530.12, 530.82, 531.0, 531.00, 531.01, 531.1, 531.2, 531.20, 531.21, 531.4, 531.41, 531.6, 531.7, 531.9, 532.0, 532.00, 532.01, 532.1, 532.2, 532.21, 532.40, 532.60, 532.7, 533.1, 533.2, 533.21, 533.4, 533.41, 533.5, 533.9, 534.0, 534.00, 534.1, 534.20, 534.21, 534.60, 534.61, 534.7, 535.01, 535.10, 535.21, 535.31, 535.40, 535.51, 535.60, 537.83, 562.01, 578.0, 578.9<br><br>HCPCS Procedure Code (any position) is any of: P9010, P9016, P9017, P9019, P9022, P9032, P9036, P9038, P9039, P9051, P9052, P9054, P9055, P9056, P9058, P9059, P9060, P9011, P9020, P9021, P9023, P9031, P9033, P9034, P9035, P9037, P9040, P9044, P9053, P9057, P9070, P9071 with Primary ICD-9 Diagnosis Code is any of: 455.2, 455.5, 455.6, 455.7, 455.8, 456.0, 530.1, 530.11, 530.13, 530.19, 530.7, 531.3, 531.40, 531.5, 531.60, 531.61, 532.20, 532.3, 532.4, 532.41, 532.5, 532.6, 532.61, 532.9, 533.0, 533.00, 533.01, 533.20, 533.3, 533.40, 533.6, 533.60, 533.61, 533.7, 534.01, 534.2, 534.3, 534.4, 534.40, 534.41, 534.5, 534.6, 534.9, 535.00, 535.11, 535.20, 535.30, 535.41, 535.50, 535.61, 562.00, 562.02, 562.03, 562.10, 562.11, 562.12, 562.13, 569.3, 569.85, 578.1, 455.1, 455.4, 455.9, 456.20, 530.10, 530.12, 530.82, 531.0, 531.00, 531.01, 531.1, 531.2, 531.20, 531.21, 531.4, 531.41, 531.6, 531.7, 531.9, 532.0, 532.00, 532.01, 532.1, 532.2, 532.21, 532.40, 532.60, 532.7, 533.1, 533.2, 533.21, 533.4, 533.41, 533.5, 533.9, 534.0, 534.00, 534.1, 534.20, 534.21, 534.60, 534.61, 534.7, 535.01, 535.10, 535.21, 535.31, 535.40, 535.51, 535.60, 537.83, 562.01, 578.0, 578.9<br><br>Revenue Center Code is any of: 0387, 0391, 0381, 0382, 0383, 0384 with Primary ICD-9 Diagnosis Code is any of: 455.2, 455.5, 455.6, 455.7, 455.8, 456.0, 530.1, 530.11, 530.13, 530.19, 530.7, 531.3, 531.40, 531.5, 531.60, 531.61, 532.20, 532.3, 532.4, 532.41, 532.5, 532.6, 532.61, 532.9, 533.0,

© 2023 Lin KJ et al. *JAMA Network Open.*

	533.00, 533.01, 533.20, 533.3, 533.40, 533.6, 533.60, 533.61, 533.7, 534.01, 534.2, 534.3, 534.4, 534.40, 534.41, 534.5, 534.6, 534.9, 535.00, 535.11, 535.20, 535.30, 535.41, 535.50, 535.61, 562.00, 562.02, 562.03, 562.10, 562.11, 562.12, 562.13, 569.3, 569.85, 578.1, 455.1, 455.4, 455.9, 456.20, 530.10, 530.12, 530.82, 531.0, 531.00, 531.01, 531.1, 531.2, 531.20, 531.21, 531.4, 531.41, 531.6, 531.7, 531.9, 532.0, 532.00, 532.01, 532.1, 532.2, 532.21, 532.40, 532.60, 532.7, 533.1, 533.2, 533.21, 533.4, 533.41, 533.5, 533.9, 534.0, 534.00, 534.1, 534.20, 534.21, 534.60, 534.61, 534.7, 535.01, 535.10, 535.21, 535.31, 535.40, 535.51, 535.60, 537.83, 562.01, 578.0, 578.9<br><br>ICD-10 Procedure Code (any position) is any of: 30230P1, 30230T1, 30233H1, 30233M1, 30233R1, 30233W1, 30240K1, 30240W1, 30243H1, 30243M1, 30243N1, 30243R1, 30243T1, 30243V1, 30230H1, 30230K1, 30230L1, 30230M1, 30230N1, 30230R1, 30230V1, 30230W1, 30233K1, 30233L1, 30233N1, 30233P1, 30233T1, 30233V1, 30240H1, 30240L1, 30240M1, 30240N1, 30240P1, 30240R1, 30240T1, 30240V1, 30243K1, 30243L1, 30243P1, 30243W1 with Primary ICD-10 Diagnosis Code is any of: I85.11, K20, K20.8, K21.0, K22.11, K22.6, K25.0, K25.4, K25.6, K26.1, K26.4, K26.5, K26.6, K27.2, K27.5, K27.6, K28.0, K28.4, K28.5, K29.01, K29.21, K29.41, K31.811, K55.21, K57.01, K57.21, K57.51, K57.53, K57.93, K64.0, K64.5, K64.8, K64.9, K92.0, I85.01, K20.0, K20.9, K25.1, K25.2, K25.5, K26.0, K26.2, K27.0, K27.1, K27.4, K28.1, K28.2, K28.6, K29.31, K29.51, K29.61, K29.71, K29.81, K29.91, K57.11, K57.31, K57.41, K57.81, K57.91, K62.5, K64.1, K64.2, K64.3, K64.4, K92.1, K92.2<br><br>HCPCS Procedure Code (any position) is any of: P9010, P9016, P9017, P9019, P9022, P9032, P9036, P9038, P9039, P9051, P9052, P9054, P9055, P9056, P9058, P9059, P9060, P9011, P9020, P9021, P9023, P9031, P9033, P9034, P9035, P9037, P9040, P9044, P9053, P9057, P9070, P9071 with Primary ICD-10 Diagnosis Code is any of: I85.11, K20, K20.8, K21.0, K22.11, K22.6, K25.0, K25.4, K25.6, K26.1, K26.4, K26.5, K26.6, K27.2, K27.5, K27.6, K28.0, K28.4, K28.5, K29.01, K29.21, K29.41, K31.811, K55.21, K57.01, K57.21, K57.51, K57.53, K57.93, K64.0, K64.5, K64.8, K64.9, K92.0, I85.01, K20.0, K20.9, K25.1, K25.2, K25.5, K26.0, K26.2, K27.0, K27.1, K27.4, K28.1, K28.2, K28.6, K29.31, K29.51, K29.61, K29.71, K29.81, K29.91, K57.11, K57.31, K57.41, K57.81, K57.91, K62.5, K64.1, K64.2, K64.3, K64.4, K92.1, K92.2<br><br>Revenue Center Code is any of: 0387, 0391, 0381, 0382, 0383, 0384 with Primary ICD-10 Diagnosis Code is any of: I85.11, K20, K20.8, K21.0, K22.11,

© 2023 Lin KJ et al. *JAMA Network Open.*

	K22.6, K25.0, K25.4, K25.6, K26.1, K26.4, K26.5, K26.6, K27.2, K27.5, K27.6, K28.0, K28.4, K28.5, K29.01, K29.21, K29.41, K31.811, K55.21, K57.01, K57.21, K57.51, K57.53, K57.93, K64.0, K64.5, K64.8, K64.9, K92.0, I85.01, K20.0, K20.9, K25.1, K25.2, K25.5, K26.0, K26.2, K27.0, K27.1, K27.4, K28.1, K28.2, K28.6, K29.31, K29.51, K29.61, K29.71, K29.81, K29.91, K57.11, K57.31, K57.41, K57.81, K57.91, K62.5, K64.1, K64.2, K64.3, K64.4, K92.1, K92.2
Intracranial bleed	**Primary position, inpatient** ICD-9 Diagnosis Code is any of: 430, 432, 432.9, 852, 852.0, 852.00, 852.04, 852.05, 852.10, 852.11, 852.12, 852.16, 852.20, 852.21, 852.22, 852.23, 852.25, 852.29, 852.3, 852.31, 852.32, 852.35, 852.36, 852.40, 852.43, 852.5, 852.51, 852.52, 852.53, 852.54, 852.59, 853.0, 853.00, 853.04, 853.05, 853.09, 853.10, 853.11, 853.13, 853.19, 431, 432.0, 432.1, 852.01, 852.02, 852.03, 852.06, 852.09, 852.1, 852.13, 852.14, 852.15, 852.19, 852.2, 852.24, 852.26, 852.30, 852.33, 852.34, 852.39, 852.4, 852.41, 852.42, 852.44, 852.45, 852.46, 852.49, 852.50, 852.55, 852.56, 853, 853.01, 853.02, 853.03, 853.06, 853.1, 853.12, 853.14, 853.15, 853.16<br>**Primary position, inpatient** ICD-10 Diagnosis Code is any of: I60.0, I60.01, I60.10, I60.11, I60.3, I60.31, I60.5, I60.51, I60.52, I60.6, I60.8, I61, I61.4, I61.8, I62.1, S06.4X0D, S06.4X0S, S06.4X1, S06.4X1S, S06.4X2, S06.4X2D, S06.4X3, S06.4X3A, S06.4X4, S06.4X4A, S06.4X5S, S06.4X6, S06.4X6A, S06.4X6D, S06.4X6S, S06.4X7, S06.4X7A, S06.4X8A, S06.5X, S06.5X0, S06.5X0S, S06.5X1, S06.5X1A, S06.5X1S, S06.5X2, S06.5X2D, S06.5X2S, S06.5X3, S06.5X3A, S06.5X3D, S06.5X3S, S06.5X4, S06.5X4A, S06.5X4D, S06.5X5D, S06.5X6A, S06.5X6S, S06.5X8, S06.5X8A, S06.5X9A, S06.6, S06.6X0, S06.6X0D, S06.6X1, S06.6X1A, S06.6X1S, S06.6X2, S06.6X2D, S06.6X2S, S06.6X3, S06.6X3A, S06.6X3D, S06.6X4S, S06.6X5A, S06.6X5S, S06.6X6D, S06.6X7A, S06.6X8, S06.6X8A, S06.6X9S, I60, I60.00, I60.02, I60.1, I60.12, I60.2, I60.30, I60.32, I60.4, I60.50, I60.7, I60.9, I61.0, I61.1, I61.2, I61.3, I61.5, I61.6, I61.9, I62, I62.0, I62.00, I62.01, I62.02, I62.03, I62.9, S06.4, S06.4X, S06.4X0, S06.4X0A, S06.4X1A, S06.4X1D, S06.4X2A, S06.4X2S, S06.4X3D, S06.4X3S, S06.4X4D, S06.4X4S, S06.4X5, S06.4X5A, S06.4X5D, S06.4X8, S06.4X9, S06.4X9A, S06.4X9D, S06.4X9S, S06.5, S06.5X0A, S06.5X0D, S06.5X1D, S06.5X2A, S06.5X4S, S06.5X5, S06.5X5A, S06.5X5S, S06.5X6, S06.5X6D, S06.5X7, S06.5X7A, S06.5X9, S06.5X9D, S06.5X9S, S06.6X, S06.6X0A, S06.6X0S, S06.6X1D, S06.6X2A, S06.6X3S, S06.6X4, S06.6X4A, S06.6X4D, S06.6X5, S06.6X5D, S06.6X6, S06.6X6A, S06.6X6S, S06.6X7, S06.6X9, S06.6X9A, S06.6X9D

Inclusion/Exclusion Definitions

© 2023 Lin KJ et al. *JAMA Network Open.*

Inclusion/Exclusion	Codes/Definition
Atrial fibrillation and flutter	ICD-9 Diagnosis Code (any position) is any of: 427.3, 427.31, 427.32<br>ICD-10 Diagnosis Code (any position) is any of: I48.0, I48.3, I48.92, I48, I48.1, I48.2, I48.4, I48.9, I48.91
Hospice Stay	Hospice claim or Inpatient stay with Patient Discharge Status = 50 or 51
Joint replacement	ICD-9 Procedure Code (any position) is any of: 00.70, 00.71, 00.72, 00.73, 81.51, 81.52, 81.53, 00.80, 00.81, 00.82, 00.83, 00.84, 81.54, 81.55, 81.80, 81.81, 81.88, 81.5, 81.59, 81.97<br>ICD-10 Procedure Code (any position) is any of: 0RRE0KZ, 0RRJ07Z, 0RRK0J6, 0RRK0KZ, 0RWL3JZ, 0RWL4JZ, 0RWN3JZ, 0RWQ3JZ, 0RWR0JZ, 0RWU0JZ, 0RWW0JZ, 0SPA0JZ, 0SPS0JZ, 0SPW0JZ, 0SR906Z, 0SR90KZ, 0SRA039, 0SRA07Z, 0SRA0KZ, 0SRB02Z, 0SRB06Z, 0SRB07Z, 0SRB0J9, 0SRB0JZ, 0SRC0JZ, 0SRD069, 0SRE00Z, 0SRE01A, 0SRE0J9, 0SRE0JA, 0SRR03A, 0SRS01Z, 0SRS03A, 0SRS03Z, 0SRS0JA, 0SRT0KZ, 0SRU0JA, 0SRV0JA, 0SRW0JA, 0SRW0JZ, 0SW94JZ, 0SWC0JZ, 0SWC4JC, 0SWD3JC, 0SWD4JC, 0SWG0JZ, 0SWG3JZ, 0SWP4JZ, 0SWS3JZ, 0SWT4JZ, 0SWU0JZ, 0SWV0JZ, 0SWW0JZ, 0SWW4JZ, 0RRE0JZ, 0RRF07Z, 0RRF0KZ, 0RRJ0J6, 0RRJ0J7, 0RRK0J7, 0RRK0JZ, 0RWH4JZ, 0RWK0JZ, 0RWK3JZ, 0RWK4JZ, 0RWM3JZ, 0RWM4JZ, 0RWN4JZ, 0RWP4JZ, 0RWQ0JZ, 0RWT0JZ, 0RWU3JZ, 0RWV3JZ, 0SPC0JZ, 0SPU0JZ, 0SR9039, 0SR907Z, 0SRA00Z, 0SRA01A, 0SRA0JZ, 0SRB019, 0SRB01A, 0SRC069, 0SRC07Z, 0SRC0LZ, 0SRD0LA, 0SRD0LZ, 0SRE009, 0SRE0JZ, 0SRS019, 0SW93JZ, 0SWA3JZ, 0SWA4JZ, 0SWC0JC, 0SWC3JC, 0SWD3JZ, 0SWF0JZ, 0SWF3JZ, 0SWF4JZ, 0SWJ3JZ, 0SWK0JZ, 0SWK3JZ, 0SWL3JZ, 0SWM0JZ, 0SWM4JZ, 0SWN0JZ, 0SWR0JZ, 0SWW3JZ, 0RRF0JZ, 0RRG0KZ, 0RRH0JZ, 0RRJ00Z, 0RRK00Z, 0RRK07Z, 0RWG0JZ, 0RWG3JZ, 0RWH3JZ, 0RWJ0JZ, 0RWL0JZ, 0RWM0JZ, 0RWP0JZ, 0RWT4JZ, 0RWV4JZ, 0SPC48Z, 0SPC4JZ, 0SPD08Z, 0SPD0JZ, 0SPD4JZ, 0SPR0JZ, 0SPT0JZ, 0SR902A, 0SR902Z, 0SR903A, 0SR903Z, 0SR904Z, 0SR90JZ, 0SRA009, 0SRA0J9, 0SRB01Z, 0SRB03A, 0SRB04Z, 0SRB069, 0SRC06A, 0SRD06Z, 0SRD07Z, 0SRD0J9, 0SRE01Z, 0SRE039, 0SRE03A, 0SRR03Z, 0SRS0JZ, 0SRS0KZ, 0SRT0JZ, 0SRU07Z, 0SRU0J9, 0SRU0JZ, 0SRU0KZ, 0SRV07Z, 0SRV0J9, 0SRW07Z, 0SRW0J9, 0SRW0KZ, 0SWB3JZ, 0SWB4JZ, 0SWD0JZ, 0SWD4JZ, 0SWH0JZ, 0SWH4JZ, 0SWL0JZ, 0SWL4JZ, 0SWM3JZ, 0SWP0JZ, 0SWQ0JZ, 0SWQ3JZ, 0SWQ4JZ, 0SWR3JZ, 0SWR4JZ, 0SWV3JZ, 0SWV4JZ, 0RRE07Z, 0RRH0KZ, 0RRJ0JZ, 0RWJ3JZ, 0RWJ4JZ, 0RWS3JZ, 0RWW4JZ, 0RWX0JZ, 0SPD48Z, 0SPV0JZ, 0SR9019, 0SR901Z, 0SR904A, 0SR906A, 0SRA019, 0SRA03A, 0SRB029, 0SRB03Z, 0SRB049, 0SRB06A, 0SRB0JA, 0SRC0LA, 0SRD06A, 0SRD0JA, 0SRD0KZ, 0SRD0L9, 0SRE019, 0SRE03Z, 0SRR01A, 0SRR01Z, 0SRR0J9, 0SRR0JA, 0SRR0KZ, 0SRS01A, 0SRS039, 0SRT07Z, 0SRT0JA, 0SRV0KZ, 0SWB0JZ, 0SWC4JZ, 0SWE4JZ, 0SWG4JZ,

© 2023 Lin KJ et al. *JAMA Network Open.*

	0SWH3JZ, 0SWJ0JZ, 0SWK4JZ, 0SWN3JZ, 0SWP3JZ, 0SWS0JZ, 0SWT3JZ, 0SWU4JZ, 0RRG07Z, 0RRG0JZ, 0RRH07Z, 0RRJ0KZ, 0RWG4JZ, 0RWH0JZ, 0RWN0JZ, 0RWP3JZ, 0RWQ4JZ, 0RWR3JZ, 0RWR4JZ, 0RWS0JZ, 0RWS4JZ, 0RWT3JZ, 0RWU4JZ, 0RWV0JZ, 0RWW3JZ, 0RWX3JZ, 0RWX4JZ, 0SPC08Z, 0SPE0JZ, 0SR901A, 0SR9029, 0SR9049, 0SR9069, 0SR90J9, 0SR90JA, 0SRA00A, 0SRA01Z, 0SRA03Z, 0SRA0JA, 0SRB02A, 0SRB039, 0SRB04A, 0SRB0KZ, 0SRC06Z, 0SRC0J9, 0SRC0JA, 0SRC0KZ, 0SRC0L9, 0SRD0JZ, 0SRE00A, 0SRE07Z, 0SRE0KZ, 0SRR019, 0SRR039, 0SRR07Z, 0SRR0JZ, 0SRS07Z, 0SRS0J9, 0SRT0J9, 0SRV0JZ, 0SW90JZ, 0SWA0JZ, 0SWC3JZ, 0SWD0JC, 0SWE0JZ, 0SWE3JZ, 0SWJ4JZ, 0SWN4JZ, 0SWS4JZ, 0SWT0JZ, 0SWU3JZ
Pulmonary Embolism	ICD-9 Diagnosis Code (any position) is any of: 415.11, 415.12, 415.19, 415.1, 415.10, 415.199, 415.15, 415.16, 415.17, 415.13<br>ICD-10 Diagnosis Code (any position) is any of: I26.01, I26.09, I26.92, I26.0, I26.99, I26.02, I26.9, I26.90
Deep Vein Thrombosis	ICD-9 Diagnosis Code (any position) is any of: 451.0, 451.1, 451.11, 451.19, 451.2, 451.8, 451.81, 451.82, 451.83, 451.84, 451.89, 451.9, 453.0, 453.1, 453.2, 453.3, 453.4, 453.40, 453.41, 453.42, 453.5, 453.50, 453.51, 453.52, 453.6, 453.7, 453.71, 453.72, 453.73, 453.74, 453.75, 453.76, 453.77, 453.79, 453.8, 453.81, 453.82, 453.83, 453.84, 453.85, 453.86, 453.87, 453.89, 453.9<br>ICD-10 Diagnosis Code (any position) is any of: I80.222, I80.229, I80.232, I80.233, I80.29, I80.293, I82.21, I82.220, I82.29, I82.290, I82.402, I82.43, I82.432, I82.443, I82.491, I82.4Y3, I82.602, I82.611, I82.90, I82.A19, I82.B1, I80.21, I80.9, I82.401, I82.409, I82.411, I82.419, I82.42, I82.421, I82.422, I82.429, I82.439, I82.44, I82.441, I82.492, I82.4Z, I82.4Z3, I82.612, I82.619, I82.622, I82.B11, I82.C11, I82.C13, I82.C19, I80.201, I80.203, I80.211, I80.212, I80.213, I80.22, I80.223, I80.23, I80.231, I82.22, I82.4, I82.403, I82.412, I82.442, I82.493, I82.4Z2, I82.60, I82.601, I82.613, I82.623, I82.890, I82.A11, I82.A12, I82.A13, I80.2, I80.20, I80.219, I80.221, I80.291, I82.1, I82.2, I82.40, I82.413, I82.431, I82.433, I82.4Y1, I82.4Y9, I82.4Z9, I82.61, I82.62, I82.B13, I82.B19, I82.C12, I80.202, I80.209, I80.239, I80.292, I80.299, I80.3, I82.0, I82.210, I82.41, I82.423, I82.449, I82.49, I82.499, I82.4Y, I82.4Y2, I82.4Z1, I82.6, I82.603, I82.609, I82.621, I82.629, I82.A1, I82.B12, I82.C1<br><br>**ICD**-10 Diagnosis Code (any position) is any of: T81.718A, T80.0XXA, T81.72XA, T82.817A, T82.818A with ICD-10 Diagnosis Code (any position) is any of: I26.99, I26.90
Evidence of Valvular Disease	ICD-9 Diagnosis Code (any position) is any of: 394.0, 394.1, 394.2, 394.9, 395.0, 395.1, 395.2, 395.9, 396.0, 396.1, 396.2, 396.3, 396.8, 396.9, 397.0, 397.1, 397.9, 398.90, 398.91, 398.99, V42.2, V43.3

© 2023 Lin KJ et al. *JAMA Network Open.*

	ICD-10 Diagnosis Code (any position) is any of: I05.2, I06.9, I07.2, I08.0, I08.3, I05.1, I05.8, I06.0, I07.8, I08.8, I09.89, I05.9, I06.1, I06.2, I07.9, I09.9, I05.0, I07.0, I08.1, I08.2, I08.9, I09.81, Z95.2, Z95.3, I06.8, I07.1, I09.1, Z95.4<br>HCPCS Procedure Code (any position) is any of: 0257T, 0258T, 0259T, 0262T, 33400, 33401, 33403, 33420, 33422, 33425, 33426, 33427, 33430, 33460, 33463, 33464, 33465, 33468, 33475, 33496, 33660, 33665
Stage 5 Chronic Kidney Disease, End Stage Renal Disease, or Dialysis	ICD-9 Diagnosis Code (any position) is any of: 403.11, 403.91, V45.1, V56.1, V56.31, V56.8, 403.01, 585.5, 585.6, V45.11, V45.12, V56, V56.0, V56.2, V56.3, V56.32<br>ICD-10 Diagnosis Code (any position) is any of: I12.0, I13.11, Y84.1, Z49, Z49.0, Z49.02, Z49.32, I13.2, N18.5, Z49.01, Z49.3, Z49.31, Z91.15, Z99.2<br>ICD-9 Procedure Code (any position) is any of: 39.95, 54.98<br>ICD-10 Procedure Code (any position) is any of: 3E1M39Z, 5A1D70Z, 5A1D90Z, 5A1D80Z<br>HCPCS Procedure Code (any position) is any of: 90935, 90940, 90941, 90942, 90945, 90955, 90959, 90962, 90963, 90964, 90965, 90969, 90985, 99559, 90937, 90939, 90947, 90951, 90952, 90953, 90954, 90956, 90957, 90958, 90960, 90961, 90966, 90967, 90968, 90970, 90989, 90990, 90993, 99512
Liver Disease	ICD-9 Diagnosis Code (any position) is any of: 571.5, 571.6, 570, 571.2, 572.8 with ICD-9 Diagnosis Code (any position) is any of: 456.0, 456.21, 567.21, 567.89, 572.4, 789.59, 456.1, 456.20, 567.0, 567.29, 567.9, 572.2<br><br>ICD-10 Diagnosis Code (any position) is any of: K70.2, K70.31, K72.9, K72.90, K74.69, K70.30, K72.1, K74.0, K74.3, K74.4, K74.5, K74.60 with ICD-10 Diagnosis Code (any position) is any of: I85.11, I86.4, K65.8, K65.9, K70.41, K71.11, K72.01, K72.91, K76.7, R18.8, I85.00, I85.01, I85.10, K65.0, K67, K72.11<br><br>ICD-9 Diagnosis Code (any position) is any of: 570<br>ICD-10 Diagnosis Code (any position) is any of: K72, K72.0, K72.00, K72.01, K72.9, K72.90, K72.91, K76.2
Major Bleeding	Primary ICD-9 Diagnosis Code is any of: 336.1, 363.61, 363.63, 372.72, 376.32, 719.15, 719.18, 866.02, 866.11, 866.12, 363.62, 377.42, 379.23, 719.11, 719.12, 719.13, 719.14, 719.16, 719.17, 719.19, 729.92, 866.01, 432, 852.0, 852.00, 852.10, 852.20, 852.3, 852.40, 852.5, 853.0, 853.00, 853.10, 432.0, 852.1, 852.2, 852.30, 852.4, 852.50, 853.1, 430, 432.9, 852.04, 852.05, 852.11, 852.12, 852.16, 852.21, 852.22, 852.23, 852.25, 852.29, 852.31, 852.32, 852.35, 852.36, 852.43, 852.51, 852.52, 852.53, 852.54, 852.59, 853.04, 853.05, 853.09, 853.11, 853.13, 853.19, 431, 432.1, 852.01, 852.02, 852.03, 852.06, 852.09, 852.13, 852.14, 852.15, 852.19, 852.24, 852.26, 852.33, 852.34, 852.39, 852.41, 852.42, 852.44, 852.45, 852.46, 852.49, 852.55, 852.56, 853.01, 853.02, 853.03, 853.06, 853.12, 853.14, 853.15, 853.16

© 2023 Lin KJ et al. *JAMA Network Open.*

	Primary ICD-10 Diagnosis Code is any of: G95.19, H05.231, H05.232, H05.239, H11.30, H31.303, H31.311, H31.312, H31.319, H31.322, H31.323, H43.11, H43.12, H47.029, M25.011, M25.012, M25.022, M25.032, M25.041, M25.042, M25.049, M25.059, M25.061, M25.062, M25.069, M25.071, M25.074, M25.076, M25.08, M79.81, S31.001A, S37.031A, S37.042A, S37.049A, S37.051A, H05.233, H11.31, H11.32, H11.33, H31.301, H31.302, H31.309, H31.313, H31.321, H31.329, H43.10, H43.13, H47.021, H47.022, H47.023, M25.00, M25.019, M25.021, M25.029, M25.031, M25.039, M25.051, M25.052, M25.072, M25.073, M25.075, S37.032A, S37.039A, S37.041A, S37.052A, S37.059A, I60.01, I60.10, I60.11, I60.31, I60.51, I60.52, I60.6, I60.8, I61.4, I61.8, S06.340A, S06.341A, S06.344A, S06.345A, S06.346A, S06.347A, S06.349A, S06.351A, S06.353A, S06.354A, S06.357A, S06.358A, S06.361A, S06.364A, S06.365A, S06.366A, S06.368A, S06.369A, S06.4X3A, S06.4X4A, S06.4X6A, S06.4X7A, S06.4X8A, S06.5X1A, S06.5X3A, S06.5X4A, S06.5X6A, S06.5X8A, S06.5X9A, S06.6X1A, S06.6X3A, S06.6X5A, S06.6X7A, S06.6X8A, I60.00, I60.02, I60.12, I60.2, I60.30, I60.32, I60.4, I60.50, I60.7, I60.9, I61.0, I61.1, I61.2, I61.3, I61.5, I61.6, I61.9, I62.00, I62.01, I62.02, I62.03, I62.9, S06.342A, S06.343A, S06.348A, S06.350A, S06.352A, S06.355A, S06.356A, S06.359A, S06.360A, S06.362A, S06.363A, S06.367A, S06.4X0A, S06.4X1A, S06.4X2A, S06.4X5A, S06.4X9A, S06.5X0A, S06.5X2A, S06.5X5A, S06.5X7A, S06.6X0A, S06.6X2A, S06.6X4A, S06.6X6A, S06.6X9A<br><br>HCPCS Procedure Code (any position) is any of: P9010, P9011, P9016, P9017, P9019, P9020, P9021, P9022, P9023, P9044, P9051, P9052, P9053, P9054, P9055, P9056, P9057, P9058, P9059, P9060, P9031, P9032, P9033, P9034, P9035, P9036, P9037, P9038, P9039, P9040 with Primary ICD-9 Diagnosis Code is any of: 280.0, 285.1, 285.9, 423.0, 455.0, 455.1, 455.2, 455.3, 455.4, 455.5, 455.6, 455.7, 455.8, 455.9, 456.0, 456.20, 459.0, 530.1, 530.7, 530.82, 531.00, 531.01, 531.1, 531.20, 531.21, 531.3, 531.40, 531.41, 531.5, 531.60, 531.61, 531.7, 531.9, 532.00, 532.01, 532.1, 532.20, 532.21, 532.3, 532.40, 532.41, 532.5, 532.60, 532.61, 532.7, 532.9, 533.00, 533.01, 533.1, 533.20, 533.21, 533.3, 533.40, 533.41, 533.5, 533.60, 533.61, 533.7, 533.9, 534.00, 534.01, 534.1, 534.20, 534.21, 534.3, 534.40, 534.41, 534.5, 534.60, 534.61, 534.7, 534.9, 535.00, 535.01, 535.10, 535.11, 535.20, 535.21, 535.30, 535.31, 535.40, 535.41, 535.50, 535.51, 535.60, 535.61, 537.83, 562.00, 562.01, 562.02, 562.03, 562.10, 562.11, 562.12, 562.13, 568.81, 569.3, 569.85, 578.0, 578.1, 578.9, 599.7, 623.8, 626.2, 626.6, 784.7, 784.8, 786.3, 790.92<br><br>ICD-9 Procedure Code (any position) is any of: 99.03, 99.04, 99.05, 99.06 with Primary ICD-9 Diagnosis Code is any of: 280.0, 285.1, 285.9, 423.0, 455.0, 455.1, 455.2, 455.3, 455.4, 455.5, 455.6, 455.7, 455.8, 455.9, 456.0, 456.20, 459.0, 530.1,

© 2023 Lin KJ et al. *JAMA Network Open.*

	530.7, 530.82, 531.00, 531.01, 531.1, 531.20, 531.21, 531.3, 531.40, 531.41, 531.5, 531.60, 531.61, 531.7, 531.9, 532.00, 532.01, 532.1, 532.20, 532.21, 532.3, 532.40, 532.41, 532.5, 532.60, 532.61, 532.7, 532.9, 533.00, 533.01, 533.1, 533.20, 533.21, 533.3, 533.40, 533.41, 533.5, 533.60, 533.61, 533.7, 533.9, 534.00, 534.01, 534.1, 534.20, 534.21, 534.3, 534.40, 534.41, 534.5, 534.60, 534.61, 534.7, 534.9, 535.00, 535.01, 535.10, 535.11, 535.20, 535.21, 535.30, 535.31, 535.40, 535.41, 535.50, 535.51, 535.60, 535.61, 537.83, 562.00, 562.01, 562.02, 562.03, 562.10, 562.11, 562.12, 562.13, 568.81, 569.3, 569.85, 578.0, 578.1, 578.9, 599.7, 623.8, 626.2, 626.6, 784.7, 784.8, 786.3, 790.92<br><br>Revenue Center Code is any of: 0381, 0382, 0383, 0384, 0387 with Primary ICD-9 Diagnosis Code is any of: 280.0, 285.1, 285.9, 423.0, 455.2, 455.5, 455.8, 456.0, 456.20, 459.0, 530.1, 530.7, 530.82, 531.1, 531.3, 531.5, 531.7, 531.9, 532.1, 532.3, 532.5, 532.7, 532.9, 533.1, 533.3, 533.5, 533.7, 533.9, 534.1, 534.3, 534.5, 534.7, 534.9, 535.00, 535.01, 535.10, 535.11, 535.20, 535.21, 535.30, 535.31, 535.40, 535.41, 535.50, 535.51, 535.60, 535.61, 537.83, 562.00, 562.01, 562.02, 562.03, 562.10, 562.11, 562.12, 562.13, 568.81, 569.3, 569.85, 578.0, 578.1, 578.9, 599.7, 623.8, 626.2, 626.6, 784.7, 784.8, 786.3, 790.92, 455.0, 455.1, 455.3, 455.4, 455.6, 455.7, 455.9, 531.00, 531.01, 531.20, 531.21, 531.40, 531.41, 531.60, 531.61, 532.00, 532.01, 532.20, 532.21, 532.40, 532.41, 532.60, 532.61, 533.00, 533.01, 533.20, 533.21, 533.40, 533.41, 533.60, 533.61, 534.00, 534.01, 534.20, 534.21, 534.40, 534.41, 534.60, 534.61<br><br>ICD-10 Procedure Code (any position) is any of: 30230P1, 30230R1, 30233K1, 30233M1, 30233N1, 30233P1, 30233V1, 30233W1, 30240M1, 30240N1, 30240V1, 30240W1, 30243L1, 30243T1, 30243V1, 30243W1, 30250K1, 30250L1, 30250N1, 30250P1, 30250V1, 30250W1, 30253H1, 30253P1, 30253R1, 30253W1, 30260L1, 30260M1, 30260N1, 30260P1, 30260V1, 30260W1, 30263N1, 30263P1, 30263T1, 30230H1, 30230K1, 30230L1, 30230M1, 30230N1, 30230T1, 30230V1, 30230W1, 30233H1, 30233L1, 30233Q1, 30233R1, 30233T1, 30240H1, 30240K1, 30240L1, 30240P1, 30240R1, 30240T1, 30243H1, 30243K1, 30243M1, 30243N1, 30243P1, 30243Q1, 30243R1, 30250H1, 30250M1, 30250R1, 30250T1, 30253K1, 30253L1, 30253M1, 30253N1, 30253T1, 30253V1, 30260H1, 30260K1, 30260R1, 30260T1, 30263H1, 30263K1, 30263L1, 30263M1, 30263R1, 30263V1, 30263W1 with Primary ICD-10 Diagnosis Code is any of: D62, K22.6, K25.0, K25.4, K25.6, K26.4, K26.6, K27.2, K27.6, K28.0, K28.4, K29.01, K29.21, K29.41, K31.811, K55.21, K57.01, K57.12, K57.21, K57.33, K57.40, K57.51, K57.52, K57.53, K57.93, K64.0, K64.8, K64.9, K66.1, N92.0, N92.1, R04.0, R04.1, D64.9, K22.8, K25.2, K26.0, K26.2, K27.0, K27.4, K28.2, K28.6, K29.31, K29.51, K29.61, K29.71, K29.81, K29.91, K57.00,

© 2023 Lin KJ et al. *JAMA Network Open.*

	K57.11, K57.13, K57.20, K57.31, K57.32, K57.41, K57.80, K57.81, K57.91, K57.92, K62.5, K64.1, K64.2, K64.3, K64.4, K92.1, K92.2, R79.1<br><br>HCPCS Procedure Code (any position) is any of: P9016, P9017, P9019, P9020, P9039, P9052, P9053, P9057, P9059, P9060, P9010, P9031, P9032, P9035, P9037, P9038, P9055, P9021, P9033, P9036, P9056, P9011, P9023, P9034, P9054, P9058, P9022, P9040, P9044, P9051 with Primary ICD-10 Diagnosis Code is any of: D62, K22.6, K25.0, K25.4, K25.6, K26.4, K26.6, K27.2, K27.6, K28.0, K28.4, K29.01, K29.21, K29.41, K31.811, K55.21, K57.01, K57.12, K57.21, K57.33, K57.40, K57.51, K57.52, K57.53, K57.93, K64.0, K64.8, K64.9, K66.1, N92.0, N92.1, R04.0, R04.1, D64.9, K22.8, K25.2, K26.0, K26.2, K27.0, K27.4, K28.2, K28.6, K29.31, K29.51, K29.61, K29.71, K29.81, K29.91, K57.00, K57.11, K57.13, K57.20, K57.31, K57.32, K57.41, K57.80, K57.81, K57.91, K57.92, K62.5, K64.1, K64.2, K64.3, K64.4, K92.1, K92.2, R79.1<br><br>Revenue Center Code is any of: 0387, 0381, 0382, 0383, 0384 with Primary ICD-10 Diagnosis Code is any of: D62, K22.6, K25.0, K25.4, K25.6, K26.4, K26.6, K27.2, K27.6, K28.0, K28.4, K29.01, K29.21, K29.41, K31.811, K55.21, K57.01, K57.12, K57.21, K57.33, K57.40, K57.51, K57.52, K57.53, K57.93, K64.0, K64.8, K64.9, K66.1, N92.0, N92.1, R04.0, R04.1, D64.9, K22.8, K25.2, K26.0, K26.2, K27.0, K27.4, K28.2, K28.6, K29.31, K29.51, K29.61, K29.71, K29.81, K29.91, K57.00, K57.11, K57.13, K57.20, K57.31, K57.32, K57.41, K57.80, K57.81, K57.91, K57.92, K62.5, K64.1, K64.2, K64.3, K64.4, K92.1, K92.2, R79.1

Covariate Definitions

Covariate	Codes/Definition
Dual Status	Categorized as true if Dual Status Code is 03, 04, or 08
Acute renal failure	ICD-9 Diagnosis Code (any position) is any of: 584, 584.5, 584.6, 584.7, 584.8, 584.9<br>ICD-10 Diagnosis Code (any position) is any of: N17.8, N17.9, N17.2, N17.0, N19, N17.1
Acute Myocardial Infarction	ICD-9 Diagnosis Code (any position) is any of: 410, 410.0, 410.00, 410.01, 410.02, 410.1, 410.10, 410.11, 410.12, 410.2, 410.20, 410.21, 410.22, 410.3, 410.30, 410.31, 410.32, 410.4, 410.40, 410.41, 410.42, 410.5, 410.50, 410.51, 410.52, 410.6, 410.60, 410.61, 410.62, 410.7, 410.70, 410.71, 410.72, 410.8, 410.80, 410.81, 410.82, 410.9, 410.90, 410.91, 410.92<br>ICD-10 Diagnosis Code is any of: I21.09, I22.1, I22.8, I21.29, I21.01, I21.19, I21.02, I21.3, I22.0, I22.2, I21.11, I21.21, I21.4, I22.9

© 2023 Lin KJ et al. *JAMA Network Open.*

Alcohol Abuse or Dependence	ICD-9 Diagnosis Code (any position) is any of: 291.2, 291.5, 291.89, 305.03, 571.2, 571.3, 291.9, 303.02, 305.01, 571.1, 291.1, 291.8, 291.81, 305.02, 571.0, E860.0, 291.0, 291.4, 291.82, 303.0, 303.00, 303.03, 303.90, 303.92, 305.0, 305.00, 425.5, 291.3, 303.01, 303.9, 303.91, 303.93, 357.5, V11.3<br>ICD-10 Diagnosis Code (any position) is any of: F10.129, F10.150, F10.19, F10.20, F10.221, F10.230, F10.250, F10.921, F10.96, F10.982, F10.99, T51.0X1D, T51.0X1S, V11.3, F10.180, F10.21, F10.220, F10.229, F10.231, F10.232, F10.26, F10.121, F10.182, F10.188, F10.251, F10.259, F10.94, F10.959, F10.97, F10.981, K70.10, K70.41, F10.10, F10.120, F10.151, F10.239, F10.27, F10.280, F10.282, F10.920, F10.951, G62.1, I42.6, K70.0, K70.11, K70.2, K70.9, F10.14, F10.159, F10.181, F10.24, F10.281, F10.288, F10.29, F10.929, F10.950, F10.980, F10.988, K70.30, K70.31, K70.40, T51.0X1A
Anemia	ICD-9 Diagnosis Code (any position) is any of: 281.4, 282, 283.9, 284.0, 285.29, 280, 281.1, 281.2, 282.2, 282.8, 283, 283.1, 283.19, 284, 284.89, 285.1, 285.2, 285.9, 280.1, 281.3, 281.8, 282.3, 283.10, 280.0, 280.8, 280.9, 281.0, 281.9, 283.0, 284.09, 285, 285.21, 285.22, 285.3, 285.8, 281, 282.9, 284.8, 284.9, 285.0<br>ICD-10 Diagnosis Code (any position) is any of: D50, D50.0, D50.8, D51.2, D52, D52.0, D56.1, D57.0, D57.4, D57.411, D57.81, D58.2, D60.1, D61.1, D61.82, D62, D64, D64.1, D51.9, D52.9, D53.0, D53.8, D55.2, D55.9, D56.5, D56.9, D57.21, D57.219, D57.812, D57.819, D58.0, D58.1, D59.0, D59.4, D59.5, D60.0, D60.8, D61.81, D61.810, D61.811, D61.89, D64.2, D51.1, D51.3, D52.1, D52.8, D55.3, D56, D57.20, D57.211, D57.212, D57.412, D58.8, D59.1, D59.8, D59.9, D61.2, D64.0, D64.3, D64.81, D50.1, D50.9, D51, D55, D55.8, D56.2, D56.3, D57.00, D57.01, D57.02, D57.2, D57.41, D57.419, D57.8, D57.80, D57.811, D58.9, D59, D60, D61, D61.0, D61.01, D61.3, D61.8, D61.818, D61.9, D51.0, D51.8, D53, D53.1, D53.2, D53.9, D55.0, D55.1, D56.0, D56.4, D56.8, D57, D57.1, D57.3, D57.40, D58, D59.2, D59.3, D59.6, D60.9, D61.09, D64.4, D64.8, D64.89, D64.9
Cardio-ablation	ICD-9 Procedure Code (any position) is any of: 37.34<br>ICD-10 Procedure Code (any position) is any of: 02593ZZ, 025J3ZZ, 025K3ZZ, 025M3ZZ, 02B83ZZ, 02T83ZZ, 025H3ZZ, 02B73ZZ, 02BG3ZZ, 025L3ZZ, 02BK3ZZ, 02BL3ZZ, 02563ZZ, 02573ZZ, 025F3ZZ, 025G3ZZ, 02B93ZZ, 02BH3ZZ, 02BJ3ZZ, 02553ZZ, 02583ZZ, 02B53ZZ, 02B63ZZ, 02BF3ZZ, 02BM3ZZ
Cardioversion	ICD-9 Procedure Code (any position) is any of: 99.61<br>ICD-10 Procedure Code (any position) is any of: 5A2204Z<br>HCPCS Procedure Code (any position) is any of: 92960, 92961

© 2023 Lin KJ et al. *JAMA Network Open.*

Chronic Kidney Disease	ICD-9 Diagnosis Code (any position) is any of: 585.3, 585.4, 585.9<br>ICD-10 Diagnosis Code (any position) is any of: N18.3, N18.9, N18.4
Congestive heart failure	ICD-9 Diagnosis Code (any position) is any of: 398.91, 402.01, 402.11, 402.91, 404.01, 404.03, 404.11, 404.13, 404.91, 404.93, 428.0, 428.1, 428.2, 428.20, 428.21, 428.22, 428.23, 428.3, 428.30, 428.31, 428.32, 428.33, 428.4, 428.40, 428.41, 428.42, 428.43, 428.9, 425.0, 425.9, 429.3, 425.18, 428, 425.1, 425.3, 425.5, 425.7, 425, 425.11, 425.2, 425.4, 425.8<br>ICD-10 Diagnosis Code (any position) is any of: I42.0, I42.4, I43, I50.1, I50.4, I50.42, I42, I42.2, I42.3, I42.6, I42.7, I42.8, I50.32, I11.0, I42.5, I42.9, I50.2, I50.20, I50.21, I50.43, A18.84, I50, I50.23, I50.3, I50.30, I50.40, I50.41, I50.9, I42.1, I50.22, I50.31, I50.33
Coronary revascularization (PTCA, stenting, CABG)	ICD-9 Procedure Code (any position) is any of: 00.66, 36.03, 36.11, 36.12, 36.32, 36.10, 36.17, 36.34, 36.06, 36.07, 36.09, 36.14, 36.15, 36.16, 36.19, 36.2, 36.33, 36.1, 36.13, 36.31<br>ICD-10 Procedure Code (any position) is any of: 021L0Z5, 0270056, 0270066, 0270076, 027007Z, 02700DZ, 02700GZ, 02700T6, 02700Z6, 027034Z, 0270376, 02703FZ, 02703Z6, 027045Z, 0270466, 02704F6, 02704T6, 0271066, 0271076, 027137Z, 02713F6, 02713GZ, 02713T6, 02713TZ, 02713ZZ, 0271446, 027144Z, 027145Z, 02714G6, 02714TZ, 02714Z6, 027206Z, 02720GZ, 0272356, 027235Z, 027237Z, 02723FZ, 02723T6, 027244Z, 02724E6, 02724T6, 0273046, 0273056, 02730E6, 02730G6, 02730ZZ, 0273376, 02733DZ, 0273446, 02734D6, 02C03ZZ, 02C23Z6, 02C30Z6, 02C34Z6, 021K0Z5, 027006Z, 02700E6, 02700FZ, 02700ZZ, 0270366, 02703D6, 02703E6, 02703ZZ, 0270446, 0270476, 02704GZ, 02704Z6, 02710E6, 02710FZ, 02710T6, 02710Z6, 0271346, 02713E6, 0271456, 0271476, 027147Z, 02714DZ, 02714F6, 027204Z, 0272056, 0272066, 02720E6, 02720EZ, 02720FZ, 02720G6, 02720T6, 02720ZZ, 0272366, 02723ZZ, 0272446, 0272466, 0272476, 02724FZ, 027304Z, 0273076, 027307Z, 02730DZ, 02730T6, 02733TZ, 027344Z, 0273456, 0273466, 02734E6, 02734EZ, 02734G6, 02734TZ, 02C03Z6, 02QA3ZZ, 0270046, 0270356, 027035Z, 027036Z, 02703F6, 02703G6, 02703TZ, 027044Z, 0270456, 02704E6, 02704G6, 0271046, 027105Z, 02710G6, 02710GZ, 02710TZ, 0271356, 02713D6, 02713DZ, 02713EZ, 0271466, 02714D6, 02714E6, 02714FZ, 027207Z, 02720F6, 02720Z6, 027234Z, 0272376, 02723D6, 02723E6, 02723EZ, 02723F6, 02723G6, 02723GZ, 02723Z6, 027246Z, 027247Z, 02724G6, 02724TZ, 02724ZZ, 027305Z, 02730F6, 02730FZ, 027334Z, 027337Z, 02733EZ, 02733G6, 02733GZ, 02733ZZ, 027347Z, 02734DZ, 02C13Z6, 02C14Z6, 02C23ZZ, 02C33ZZ, 02QB3ZZ, 02QC3ZZ, 02700EZ, 02700F6, 02700G6, 02700TZ, 027037Z, 027047Z, 02704ZZ, 027106Z, 02710DZ, 02710EZ, 027135Z, 02713FZ,

© 2023 Lin KJ et al. *JAMA Network Open.*

	02713G6, 02714EZ, 02714T6, 027205Z, 0272076, 02720D6, 027236Z, 02724D6, 02724F6, 02724GZ, 0273066, 02730Z6, 0273346, 0273356, 027335Z, 0273366, 02733E6, 02733F6, 02733FZ, 02733T6, 02733Z6, 02C00Z6, 02C10Z6, 021K4Z5, 021L4Z5, 027004Z, 027005Z, 02700D6, 0270346, 02703DZ, 02703EZ, 02703GZ, 02703T6, 027046Z, 02704D6, 02704DZ, 02704EZ, 02704FZ, 02704TZ, 027104Z, 0271056, 027107Z, 02710D6, 02710F6, 02710ZZ, 027134Z, 0271366, 027136Z, 0271376, 02713Z6, 027146Z, 02714GZ, 02714ZZ, 0272046, 02720DZ, 02720TZ, 0272346, 02723DZ, 02723TZ, 0272456, 027245Z, 02724DZ, 02724EZ, 02724Z6, 027306Z, 02730D6, 02730EZ, 02730GZ, 02730TZ, 027336Z, 02733D6, 027345Z, 027346Z, 0273476, 02734F6, 02734FZ, 02734GZ, 02734T6, 02734Z6, 02734ZZ, 02C04Z6, 02C13ZZ, 02C20Z6, 02C24Z6, 02C33Z6, 02QA4ZZ, 02QB4ZZ, 02QC4ZZ<br>HCPCS Procedure Code (any position) is any of: 33510, 33511, 33517, 92921, 92924, 92938, 92941, 92973, 92995, 33516, 33518, 33519, 33521, 33534, 33572, 92944, 92984, 33513, 33535, 92982, 92996, 33140, 33141, 33512, 33514, 33523, 33545, 92920, 92937, 92943, 33522, 33530, 33533, 33536, 92925
COPD	ICD-9 Diagnosis Code (any position) is any of: 491.0, 491.9, 491.8, 492.0, 492.8, 491.1, 491.2, 496, 491.20, 491.21, 491.22<br>ICD-10 Diagnosis Code (any position) is any of: J44.9, J43.0, J41.8, J43.8, J43.9, J41.0, J42, J41.1, J43.1, J43.2, J44.0, J44.1
Diabetes	ICD-9 Diagnosis Code (any position) is any of: 249.00, 249.10, 249.11, 249.40, 249.41, 249.50, 249.51, 249.60, 249.61, 249.81, 249.91, 250.01, 250.02, 250.10, 250.11, 250.13, 250.20, 250.32, 250.42, 250.51, 250.61, 250.63, 250.70, 250.72, 250.73, 250.83, 249.01, 249.20, 249.21, 249.30, 249.31, 249.70, 249.71, 249.80, 249.90, 250.00, 250.03, 250.12, 250.21, 250.22, 250.23, 250.30, 250.31, 250.33, 250.40, 250.41, 250.43, 250.50, 250.52, 250.53, 250.60, 250.62, 250.71, 250.80, 250.81, 250.82, 250.90, 362.01, 362.02, 362.03, 362.06, 250.91, 250.92, 250.93, 357.2, 362.04, 362.05, 366.41<br>ICD-10 Diagnosis Code (any position) is any of: E08.00, E08.10, E08.11, E08.21, E08.29, E08.311, E08.319, E08.321, E08.3211, E08.3212, E08.329, E08.3291, E08.3292, E08.331, E08.339, E08.3392, E08.3393, E08.3413, E08.3419, E08.3491, E08.3493, E08.3499, E08.351, E08.3512, E08.3513, E08.3522, E08.3523, E08.3529, E08.3533, E08.3539, E08.3543, E08.3549, E08.3551, E08.3552, E08.36, E08.37X3, E08.39, E08.42, E08.52, E08.610, E08.620, E08.621, E08.622, E08.628, E08.630, E08.641, E08.8, E09.01, E09.29, E09.321, E09.3212, E09.3213, E09.3219, E09.3291, E09.3292, E09.3293, E09.331, E09.3313, E09.339, E09.3419, E09.3493, E09.3499, E09.351, E09.3511, E09.3512, E09.3513, E09.3519, E09.3522, E09.3523,

© 2023 Lin KJ et al. *JAMA Network Open.*

	E09.3532, E09.3542, E09.3552, E09.3553, E09.359, E09.3593, E09.36, E09.37X1, E09.39, E09.42, E09.43, E09.51, E09.52, E09.59, E09.610, E09.620, E09.622, E09.630, E09.649, E09.69, E09.8, E10.22, E10.29, E10.321, E10.3211, E10.3212, E10.329, E10.3291, E10.3299, E10.3311, E10.3312, E10.3319, E10.339, E10.3391, E10.3392, E10.3399, E10.3419, E10.349, E10.3491, E10.3492, E10.351, E10.3511, E10.3512, E10.3519, E10.37X1, E10.37X3, E10.37X9, E10.39, E10.40, E10.43, E10.49, E10.51, E10.52, E10.59, E10.610, E10.620, E10.622, E10.628, E08.01, E08.22, E08.3213, E08.3219, E08.3293, E08.3299, E08.3311, E08.3312, E08.3313, E08.3319, E08.3391, E08.3399, E08.341, E08.3411, E08.3412, E08.349, E08.3492, E08.3511, E08.3519, E08.3521, E08.3531, E08.3532, E08.3541, E08.3542, E08.3553, E08.3559, E08.359, E08.3591, E08.3592, E08.3593, E08.3599, E08.37X1, E08.37X2, E08.37X9, E08.40, E08.41, E08.43, E08.44, E08.49, E08.51, E08.59, E08.618, E08.638, E08.649, E08.65, E08.69, E08.9, E09.00, E09.10, E09.11, E09.21, E09.22, E09.311, E09.319, E09.3211, E09.329, E09.3299, E09.3311, E09.3312, E09.3319, E09.3391, E09.3393, E09.3399, E09.341, E09.3412, E09.3413, E09.349, E09.3491, E09.3533, E09.3551, E09.3591, E09.3592, E09.3599, E09.37X9, E09.40, E09.41, E09.44, E09.49, E09.618, E09.621, E09.628, E09.638, E09.641, E09.65, E09.9, E10.10, E10.11, E10.21, E10.311, E10.319, E10.3213, E10.3219, E10.3292, E10.3293, E10.331, E10.3313, E10.3393, E10.341, E10.3411, E10.3412, E10.3413, E10.3493, E10.3499, E10.3513, E10.359, E10.36, E10.37X2, E10.41, E10.42, E10.44, E10.618, E10.621, E10.630, E10.649, E10.69, E10.8, E10.9, E11.00, E11.10, E11.11, E11.22, E11.319, E11.321, E11.3212, E11.329, E11.3293, E11.331, E11.3312, E11.3313, E11.339, E11.3391, E11.3393, E11.3399, E11.3411, E11.3413, E11.3419, E11.349, E11.3491, E11.3492, E11.3511, E11.3512, E11.3521, E11.3522, E11.3523, E11.3529, E11.3531, E11.3532, E11.3533, E11.3551, E11.3552, E11.3553, E11.3593, E11.37X1, E11.37X2, E11.37X3, E11.37X9, E11.40, E11.43, E11.44, E11.49, E11.52, E11.59, E11.618, E11.622, E11.628, E11.630, E11.641, E11.649, E11.9, E13.00, E13.21, E13.22, E13.29, E13.311, E13.319, E13.3211, E13.3291, E13.3292, E13.331, E13.3312, E13.3319, E13.339, E13.3391, E13.3392, E13.3399, E13.3411, E13.3412, E13.3493, E13.3511, E13.3521, E13.3522, E13.3523, E13.3531, E13.3533, E13.3539, E13.3542, E13.3549, E13.3551, E13.3552, E13.3553, E13.3559, E13.36, E13.40, E13.51, E13.52, E13.59, E13.610, E13.618, E13.621, E13.622, E13.628, E13.630, E13.641, E13.649, E13.65, E10.638, E10.641, E10.65, E11.01, E11.21, E11.29, E11.311, E11.3211, E11.3213, E11.3219, E11.3291, E11.3292, E11.3299, E11.3311, E11.3319, E11.3392, E11.341, E11.3412, E11.3493, E11.3499, E11.351, E11.3513, E11.3519, E11.3539, E11.3541, E11.3542, E11.3543,

© 2023 Lin KJ et al. *JAMA Network Open.*

	E11.3549, E11.3559, E11.359, E11.3591, E11.3592, E11.3599, E11.36, E11.39, E11.41, E11.42, E11.51, E11.610, E11.620, E11.621, E11.638, E11.65, E11.69, E11.8, E13.01, E13.10, E13.11, E13.321, E13.3212, E13.3213, E13.3219, E13.329, E13.3293, E13.3299, E13.3311, E13.3313, E13.3393, E13.341, E13.3413, E13.3419, E13.349, E13.3491, E13.3492, E13.3499, E13.351, E13.3512, E13.3513, E13.3519, E13.3529, E13.3532, E13.3541, E13.3543, E13.359, E13.39, E13.41, E13.42, E13.43, E13.44, E13.49, E13.620, E13.638, E13.69, E13.8, E13.9
Falls	ICD-9 Diagnosis Code (any position) is any of: E880.9, E883.2, E883.9, E888.0, E880, E880.1, E884, E884.5, E884.9, E885.2, E886, E886.9, E881.0, E883, E884.0, E885.1, E888.1, V15.88, E881, E882, E884.1, E884.2, E884.3, E884.4, E884.6, E885, E885.9, E887, E888, E888.9, E880.0, E881.1, E883.0, E883.1, E885.0, E885.3, E885.4, E886.0, E888.8<br>ICD-10 Diagnosis Code (any position) is any of: V00.118D, V00.122D, V00.131D, V00.141D, V00.142A, V00.148D, V00.151A, V00.182A, V00.188A, V00.212D, V00.221D, V00.228A, V00.228D, V00.288A, V00.312D, V00.318A, V00.321A, V00.381D, V00.382D, V00.388D, V00.812A, V00.821A, V00.821D, V00.822D, V00.828A, V00.831A, V00.832A, V00.892D, W00.1XXA, W00.2XXA, W00.9XXD, W01.10XD, W01.110A, W01.118D, W01.119D, W01.198D, W05.2XXA, W06.XXXD, W09.0XXA, W09.2XXA, W09.8XXA, W09.8XXD, W10.0XXD, W10.1XXA, W10.2XXD, W12.XXXD, W13.1XXA, W13.2XXA, W13.3XXA, W13.8XXA, W13.9XXD, W16.021D, W16.022D, W16.222A, W16.311D, W16.312D, W16.322D, W16.331A, W16.332A, W16.42XD, W16.522D, W16.531A, W16.621A, W16.712A, W16.712D, W16.722A, W16.822A, W16.822D, W16.831D, W16.92XD, W17.1XXD, W17.3XXA, W17.81XD, W17.89XA, W18.02XA, W18.12XD, W18.40XA, W18.49XA, V00.112A, V00.112D, V00.142D, V00.152D, V00.158D, V00.211D, V00.212A, V00.218A, V00.282D, V00.311D, V00.328A, V00.822A, V00.832D, V00.898A, W00.0XXA, W00.1XXD, W08.XXXD, W09.2XXD, W10.9XXA, W13.0XXD, W16.011A, W16.021A, W16.032D, W16.121D, W16.211A, W16.212D, W16.222D, W16.42XA, W16.522A, W16.611D, W16.621D, W16.722D, W16.811A, W16.832A, W16.91XD, W17.1XXA, W17.82XD, W18.01XA, W18.09XD, W18.12XA, W18.31XA, W18.39XD, W18.42XA, W18.42XD, W18.43XD, W19.XXXA, V00.118A, V00.122A, V00.131A, V00.148A, V00.152A, V00.158A, V00.181A, V00.181D, V00.182D, V00.188D, V00.218D, V00.222A, V00.222D, V00.281D, V00.311A, V00.312A, V00.321D, V00.328D, V00.811D, V00.831D, V00.891D, W00.9XXA, W01.111A, W01.111D, W01.118A, W01.198A, W05.1XXA, W06.XXXA, W07.XXXA, W09.1XXA, W10.8XXA, W10.8XXD, W10.9XXD, W11.XXXA, W13.0XXA, W13.3XXD, W16.022A, W16.112A, W16.112D, W16.131A, W16.132A,

© 2023 Lin KJ et al. *JAMA Network Open.*

	W16.132D, W16.211D, W16.322A, W16.521A, W16.711D, W16.811D, W16.831A, W16.832D, W16.91XA, W18.00XD, W18.2XXD, W18.30XD, W18.40XD, V00.111A, V00.128D, V00.132A, V00.138A, V00.151D, V00.211A, V00.221A, V00.281A, V00.282A, V00.322A, V00.322D, V00.381A, V00.811A, V00.818A, V00.838A, V00.891A, V00.892A, W00.0XXD, W00.2XXD, W01.0XXA, W01.119A, W01.190A, W03.XXXD, W05.2XXD, W07.XXXD, W08.XXXA, W09.0XXD, W11.XXXD, W12.XXXA, W13.1XXD, W13.2XXD, W13.4XXD, W14.XXXD, W16.011D, W16.012D, W16.031A, W16.032A, W16.111A, W16.122D, W16.221A, W16.221D, W16.311A, W16.312A, W16.331D, W16.332D, W16.41XA, W16.41XD, W16.511D, W16.512A, W16.532A, W16.532D, W16.721A, W16.721D, W16.812A, W16.812D, W16.821A, W16.92XA, W17.0XXA, W17.2XXA, W17.2XXD, W17.89XD, W18.00XA, W18.01XD, W18.02XD, W18.11XD, W18.30XA, W18.31XD, W18.41XA, V00.111D, V00.121A, V00.121D, V00.128A, V00.132D, V00.138D, V00.141A, V00.288D, V00.318D, V00.382A, V00.388A, V00.812D, V00.818D, V00.828D, V00.838D, V00.898D, W01.0XXD, W01.10XA, W01.110D, W01.190D, W03.XXXA, W04.XXXA, W04.XXXD, W05.0XXA, W05.0XXD, W05.1XXD, W09.1XXD, W10.0XXA, W10.1XXD, W10.2XXA, W13.4XXA, W13.8XXD, W13.9XXA, W14.XXXA, W15.XXXA, W15.XXXD, W16.012A, W16.031D, W16.111D, W16.121A, W16.122A, W16.131D, W16.212A, W16.321A, W16.321D, W16.511A, W16.512D, W16.521D, W16.531D, W16.611A, W16.612A, W16.612D, W16.622A, W16.622D, W16.711A, W16.821D, W17.0XXD, W17.3XXD, W17.4XXA, W17.4XXD, W17.81XA, W17.82XA, W18.11XA, W18.2XXA, W18.39XA, W18.41XD, W18.43XA, W18.49XD, W19.XXXD, Z91.81
Fractures	ICD-9 Diagnosis Code (any position) is any of: 733.13, 733.15, 733.19, 800.03, 800.05, 800.22, 800.26, 800.46, 800.53, 800.55, 800.59, 800.60, 800.64, 800.66, 800.7, 800.74, 800.79, 800.8, 800.80, 800.91, 800.95, 801.02, 801.04, 801.15, 801.16, 801.2, 801.21, 801.26, 801.29, 801.49, 801.51, 801.54, 801.82, 801.86, 801.93, 802, 802.21, 802.25, 802.30, 802.31, 802.39, 802.6, 803, 803.00, 803.03, 803.09, 803.10, 803.13, 803.14, 803.2, 803.26, 803.36, 803.40, 803.42, 803.45, 803.46, 803.50, 803.53, 803.60, 803.64, 803.7, 803.8, 803.83, 803.89, 804.0, 804.01, 804.09, 804.11, 804.12, 804.19, 804.26, 804.30, 804.41, 804.42, 804.45, 804.70, 804.74, 804.76, 804.81, 804.85, 804.89, 804.9, 805.01, 805.06, 805.07, 805.12, 805.16, 805.3, 805.6, 805.8, 806.0, 806.13, 806.19, 806.24, 806.25, 806.30, 806.37, 806.4, 806.69, 806.71, 806.9, 807.02, 807.06, 807.07, 807.14, 807.15, 807.16, 807.19, 807.2, 808, 808.4, 808.43, 808.44, 808.5, 808.59, 810.0, 810.02, 811.0, 811.00, 811.19, 812.30, 812.31, 812.41, 812.50, 813.0, 813.08, 813.11, 813.2, 813.83, 813.9, 815.0, 815.12, 820, 820.01, 820.03, 820.13, 820.3, 821.0, 821.10, 821.29, 821.31, 821.33, 822.0,

© 2023 Lin KJ et al. *JAMA Network Open.*

	822.1, 823, 823.01, 823.32, 823.82, 823.91, 824.2, 824.7, 825.1, 825.34, 828.0, 828.1, 733.1, 733.16, 800.04, 800.1, 800.10, 800.11, 800.12, 800.13, 800.16, 800.21, 800.23, 800.29, 800.30, 800.31, 800.4, 800.43, 800.45, 800.50, 800.54, 800.71, 800.81, 800.84, 800.89, 800.9, 800.92, 800.94, 801.00, 801.4, 801.41, 801.43, 801.63, 801.73, 801.75, 801.76, 801.8, 801.80, 801.83, 801.84, 801.91, 802.20, 802.26, 803.0, 803.05, 803.12, 803.24, 803.3, 803.34, 803.39, 803.4, 803.49, 803.55, 803.56, 803.6, 803.65, 803.71, 803.73, 803.74, 803.91, 803.93, 803.95, 804.05, 804.10, 804.13, 804.20, 804.32, 804.34, 804.39, 804.40, 804.63, 804.65, 804.7, 804.71, 804.75, 804.79, 804.8, 804.80, 804.82, 804.83, 804.84, 804.92, 804.99, 805.02, 805.10, 805.13, 805.15, 805.17, 805.4, 805.5, 806.00, 806.03, 806.05, 806.11, 806.12, 806.22, 806.26, 806.3, 806.31, 806.34, 806.36, 806.61, 806.7, 807.3, 808.0, 808.2, 808.49, 808.9, 810.03, 811.02, 812, 812.2, 812.20, 812.21, 812.43, 813.06, 813.12, 813.20, 813.30, 813.32, 813.41, 813.50, 813.53, 813.8, 813.81, 813.91, 814.0, 814.10, 814.13, 814.18, 815, 815.03, 815.10, 815.11, 815.19, 819.1, 820.09, 820.20, 820.22, 820.31, 821.01, 821.11, 821.22, 821.32, 823.11, 823.20, 823.80, 823.90, 823.92, 824.8, 825, 825.2, 825.31, 825.32, 827, 827.0, 829.0, 733.11, 733.14, 800, 800.06, 800.09, 800.14, 800.3, 800.32, 800.42, 800.5, 800.51, 800.52, 800.56, 800.61, 800.69, 800.73, 800.82, 800.86, 800.90, 800.93, 801, 801.0, 801.06, 801.12, 801.19, 801.23, 801.24, 801.33, 801.35, 801.45, 801.55, 801.56, 801.6, 801.61, 801.62, 801.64, 801.66, 801.69, 801.74, 801.90, 801.94, 801.99, 802.1, 802.2, 802.22, 802.24, 802.36, 802.37, 802.5, 802.7, 802.8, 803.01, 803.06, 803.16, 803.25, 803.33, 803.43, 803.44, 803.5, 803.54, 803.59, 803.72, 803.75, 803.79, 803.80, 803.82, 803.85, 803.9, 803.94, 803.99, 804.02, 804.03, 804.16, 804.22, 804.23, 804.3, 804.35, 804.36, 804.44, 804.46, 804.52, 804.54, 804.61, 804.62, 804.69, 804.72, 804.90, 804.94, 805, 805.04, 805.05, 805.14, 805.18, 806.07, 806.08, 806.15, 806.16, 806.2, 806.20, 806.27, 806.28, 806.32, 806.35, 806.5, 806.72, 806.8, 807.01, 807.04, 807.11, 807.4, 807.5, 808.53, 808.8, 809.0, 810, 810.10, 810.12, 811.01, 811.03, 811.10, 811.12, 812.02, 812.09, 812.1, 812.11, 812.40, 812.5, 812.53, 813, 813.01, 813.02, 813.04, 813.05, 813.1, 813.15, 813.16, 813.43, 813.47, 814, 814.02, 814.03, 814.05, 814.06, 814.08, 814.17, 814.19, 815.00, 815.04, 816.03, 816.11, 816.12, 816.13, 817, 818.0, 820.0, 820.2, 820.32, 820.9, 821.20, 821.23, 821.3, 822, 823.00, 823.10, 823.12, 823.2, 823.21, 823.3, 823.42, 823.9, 824.0, 824.3, 825.22, 825.23, 825.24, 825.29, 825.33, 825.39, 826.0, 800.0, 800.00, 800.20, 800.33, 800.35, 800.40, 800.49, 800.70, 800.76, 800.83, 800.85, 800.96, 800.99, 801.03, 801.05, 801.1, 801.11, 801.13, 801.20, 801.3, 801.39, 801.42, 801.5, 801.50, 801.53, 801.71, 801.72, 801.79, 801.95, 802.0, 802.23, 802.28, 802.29, 802.33, 802.38, 802.9, 803.02, 803.19, 803.21, 803.22, 803.30, 803.31, 803.32, 803.41, 803.51, 803.52, 803.62, 803.63, 803.76, 803.81, 803.84, 803.86, 803.90, 803.92, 803.96, 804,

© 2023 Lin KJ et al. *JAMA Network Open.*

	804.04, 804.06, 804.14, 804.21, 804.25, 804.29, 804.31, 804.33, 804.4, 804.43, 804.49, 804.51, 804.55, 804.56, 804.6, 804.60, 804.66, 804.73, 804.93, 804.95, 804.96, 805.00, 805.7, 805.9, 806.01, 806.02, 806.09, 806.1, 806.14, 806.18, 806.21, 806.29, 806.33, 806.38, 806.6, 806.60, 806.62, 806.70, 806.79, 807.03, 807.12, 807.6, 808.42, 809, 809.1, 810.00, 810.01, 811, 811.11, 811.13, 812.00, 812.19, 812.51, 812.54, 813.00, 813.03, 813.07, 813.13, 813.18, 813.23, 813.3, 813.4, 813.40, 813.44, 813.45, 813.52, 813.54, 813.80, 813.82, 813.92, 813.93, 814.04, 814.07, 814.1, 814.11, 814.14, 814.16, 815.01, 815.09, 815.1, 816, 816.00, 816.01, 816.02, 816.1, 816.10, 817.1, 818.1, 819.0, 820.02, 820.10, 820.12, 820.8, 821, 821.1, 821.2, 821.39, 823.02, 823.22, 823.30, 823.31, 823.41, 823.8, 824, 824.1, 824.4, 824.5, 824.6, 825.20, 825.25, 825.3, 825.30, 825.35, 829.1, 733.10, 733.12, 800.01, 800.02, 800.15, 800.19, 800.2, 800.24, 800.25, 800.34, 800.36, 800.39, 800.41, 800.44, 800.6, 800.62, 800.63, 800.65, 800.72, 800.75, 801.01, 801.09, 801.10, 801.14, 801.22, 801.25, 801.30, 801.31, 801.32, 801.34, 801.36, 801.40, 801.44, 801.46, 801.52, 801.59, 801.60, 801.65, 801.7, 801.70, 801.81, 801.85, 801.89, 801.9, 801.92, 801.96, 802.27, 802.3, 802.32, 802.34, 802.35, 802.4, 803.04, 803.1, 803.11, 803.15, 803.20, 803.23, 803.29, 803.35, 803.61, 803.66, 803.69, 803.70, 804.00, 804.1, 804.15, 804.2, 804.24, 804.5, 804.50, 804.53, 804.59, 804.64, 804.86, 804.91, 805.0, 805.03, 805.08, 805.1, 805.11, 805.2, 806, 806.04, 806.06, 806.10, 806.17, 806.23, 806.39, 807, 807.0, 807.00, 807.05, 807.08, 807.09, 807.1, 807.10, 807.13, 807.17, 807.18, 808.1, 808.3, 808.41, 808.51, 808.52, 808.54, 810.1, 810.11, 810.13, 811.09, 811.1, 812.0, 812.01, 812.03, 812.10, 812.12, 812.13, 812.3, 812.4, 812.42, 812.44, 812.49, 812.52, 812.59, 813.10, 813.14, 813.17, 813.21, 813.22, 813.31, 813.33, 813.42, 813.46, 813.5, 813.51, 813.90, 814.00, 814.01, 814.09, 814.12, 814.15, 815.02, 815.13, 815.14, 816.0, 817.0, 818, 819, 820.00, 820.1, 820.11, 820.19, 820.21, 820.30, 821.00, 821.21, 821.30, 823.0, 823.1, 823.4, 823.40, 823.81, 824.9, 825.0, 825.21, 826, 826.1, 827.1, 828, 829<br>ICD-10 Diagnosis Code (any position) is any of: M48.51XA, M48.57XA, M80.011A, M80.019A, M80.051A, M80.052A, M80.079A, M80.811A, M80.839A, M80.849A, M80.852A, M80.871A, M80.872A, M80.88XA, M84.433A, M84.463A, M84.469A, M84.472A, M84.473A, M84.478A, M84.519A, M84.542A, M84.552A, M84.569A, M84.575A, M84.58XA, M84.621A, M84.631A, M84.649A, M84.652A, M84.68XA, S02.110B, S02.119A, S02.2XXB, S02.411A, S06.335A, S06.361A, S06.4X4A, S06.5X3A, S06.5X4A, S06.6X1A, S06.6X4A, S06.6X8A, S06.892A, S06.899A, S12.041A, S12.101A, S12.101B, S12.111A, S12.112A, S12.120B, S12.131A, S12.150B, S12.190A, S12.190B, S12.191A, S12.201A, S12.231B, S12.251B, S12.290B, S12.300A, S12.301A, S12.350A, S12.351B, S12.400B, S12.450A, S12.491B, S12.500B,

© 2023 Lin KJ et al. *JAMA Network Open.*

	S12.501B, S12.550B, S14.109A, S14.113A, S14.117A, S14.124A, S14.135A, S14.151A, S14.155A, S22.011A, S22.012B, S22.018B, S22.019A, S22.022B, S22.028A, S22.031A, S22.038A, S22.039A, S22.042A, S22.049A, S22.049B, S22.051A, S22.051B, S22.052A, S22.060A, S22.061A, S22.069A, S22.071A, S22.072B, S22.078A, S22.080B, S22.081B, S22.088B, S22.20XA, S22.24XB, S22.32XB, S22.43XB, S22.49XA, S22.9XXA, S22.9XXB, S24.102A, S24.104A, S24.134A, S32.002A, S32.030A, S32.031B, S32.032B, S32.040B, S32.041B, S32.052A, S32.052B, S32.112B, S32.119A, S32.122A, S32.130B, S32.15XA, S32.15XB, S32.19XA, S32.314B, S32.315A, S32.392B, S32.399A, S32.401A, S32.402A, S32.409A, S32.412A, S32.414B, S32.422B, S32.426A, S32.436A, S32.444A, S32.445A, S32.446B, S32.451B, S32.454B, S32.456A, S32.471B, S32.473B, S32.475A, S32.484A, S32.485A, S32.509B, S32.511A, S32.612B, S32.615A, S32.615B, S32.691A, S32.691B, S32.692A, S32.810B, S32.811A, S32.89XA, S32.9XXB, S34.101A, S34.111A, S34.119A, S34.122A, S34.129A, S34.131A, S34.139A, S42.015B, S42.016B, S42.018A, S42.019B, S42.031A, S42.034A, S42.102A, S42.109A, S42.111A, S42.112A, S42.114B, S42.116A, S42.121A, S42.122B, S42.133A, S42.134B, S42.142A, S42.143A, S42.152A, S42.153A, S42.155A, S42.192B, S42.199B, S42.211B, S42.212A, S42.212B, S42.214B, S42.223B, S42.225A, S42.239A, S42.242B, S42.255A, S42.261A, S42.263B, S42.266A, S42.266B, S42.292A, S42.301A, S42.309A, S42.311A, S42.321B, S42.323A, S42.324B, S42.332A, S42.335A, S42.336B, S42.342A, S42.343A, S42.351B, S42.353B, S42.366A, S42.399A, S42.401A, S42.412B, S42.415A, S42.416A, S42.423A, S42.424A, S42.425B, S42.432A, S42.443A, S42.445A, S42.447A, S42.453B, S42.456A, S42.466A, S42.466B, S42.472B, S42.474B, S42.475B, S42.489A, S42.491B, S42.492A, S42.493A, S42.493B, S42.494A, S42.495A, S42.496A, S42.496B, S42.90XA, S49.009A, S49.041A, S49.092A, S52.001A, S52.001B, S52.001C, S52.009A, S52.009C, S52.019A, S52.022A, S52.024C, S52.025C, S52.026C, S52.032B, S52.045A, S52.046B, S52.046C, S52.091B, S52.092C, S52.099C, S52.122C, S52.124C, S52.125A, S52.131A, S52.132A, S52.133B, S52.134C, S52.135C, S52.136A, S52.221C, S52.223C, S52.225B, S52.231A, S52.231C, S52.232A, S52.233C, S52.235B, S52.242A, S52.243C, S52.246B, S52.252A, S52.253A, S52.262B, S52.263B, S52.266A, S52.272A, S52.272B, S52.279B, S52.279C, S52.281A, S52.283C, S52.302C, S52.321A, S52.321C, S52.322B, S52.325A, S52.325B, S52.326A, S52.331B, S52.332A, S52.334A, S52.336A, S52.341A, S52.341B, S52.342A, S52.345A, S52.351A, S52.352C, S52.354A, S52.355C, S52.356B, S52.362A, S52.362B, S52.363A, S52.365C, S52.366C, S52.372A, S52.372B, S52.379B, S52.391A, S52.391B, S52.399A, S52.501A, S52.501C, S52.509C, S52.511C, S52.513A, S52.514A, S52.515A, S52.529A, S52.531C, S52.532A, S52.532C, S52.541A, S52.551A, S52.551C, S52.552A, S52.552B, S52.561A, S52.561B,

© 2023 Lin KJ et al. *JAMA Network Open.*

	S52.562C, S52.569C, S52.571A, S52.599C, S52.609C, S52.611C, S52.613A, S52.691B, S52.92XB, S59.029A, S59.032A, S59.042A, S59.102A, S59.111A, S59.121A, S59.191A, S59.199A, S59.201A, S59.209A, S59.219A, S59.221A, S62.013A, S62.013B, S62.021A, S62.021B, S62.024B, S62.031A, S62.031B, S62.033B, S62.035B, S62.036A, S62.109A, S62.113B, S62.114A, S62.116B, S62.136B, S62.141B, S62.153B, S62.155B, S62.161A, S62.162B, S62.164A, S62.164B, S62.166B, S62.172A, S62.174A, S62.175A, S62.183A, S62.185A, S62.186B, S62.201B, S62.202A, S62.209A, S62.212A, S62.213A, S62.221B, S62.224B, S62.225A, S62.232B, S62.236A, S62.236B, S62.242A, S62.242B, S62.244A, S62.246A, S62.251A, S62.251B, S62.252A, S62.254A, S62.255A, S62.292B, S62.303A, S62.304B, S62.305A, S62.306A, S62.307B, S62.316B, S62.321A, S62.322B, S62.323A, S62.324A, S62.325B, S62.327A, S62.329B, S62.334A, S62.334B, S62.335A, S62.335B, S62.337A, S62.337B, S62.344A, S62.344B, S62.346A, S62.347A, S62.349B, S62.350B, S62.351A, S62.351B, S62.357B, S62.358B, S62.359A, S62.359B, S62.369B, S62.394B, S62.395A, S62.397B, S62.501B, S62.511B, S62.512A, S62.516B, S62.600B, S62.601B, S62.606B, S62.607B, S62.619A, S62.625B, S62.629B, S62.632B, S62.638B, S62.641A, S62.644A, S62.644B, S62.653B, S62.655A, S62.662A, S62.666B, S62.668A, S62.669B, S62.92XA, S62.92XB, S72.001A, S72.002B, S72.019A, S72.019B, S72.022A, S72.035A, S72.036A, S72.042C, S72.043C, S72.044A, S72.045B, S72.046B, S72.059C, S72.062B, S72.062C, S72.063B, S72.063C, S72.065A, S72.092A, S72.114C, S72.121A, S72.122B, S72.124C, S72.125C, S72.126B, S72.126C, S72.131B, S72.132A, S72.133A, S72.136A, S72.136B, S72.142A, S72.142C, S72.146C, S72.22XA, S72.26XB, S72.26XC, S72.301C, S72.302A, S72.324A, S72.325B, S72.325C, S72.331B, S72.332B, S72.333A, S72.333B, S72.333C, S72.334B, S72.335C, S72.336A, S72.341B, S72.343B, S72.346B, S72.351A, S72.351B, S72.352C, S72.354A, S72.362A, S72.392B, S72.392C, S72.401C, S72.402A, S72.413C, S72.421C, S72.422B, S72.432A, S72.433B, S72.436A, S72.436C, S72.441B, S72.445B, S72.445C, S72.446C, S72.453A, S72.463B, S72.499B, S72.8X9A, S72.90XE, S72.91XE, S72.92XA, S79.011A, S79.091A, S79.099A, S79.131A, S79.139A, S82.012C, S82.013B, S82.015A, S82.016B, S82.021A, S82.021B, S82.022A, S82.023C, S82.025A, S82.026C, S82.031B, S82.032A, S82.032C, S82.033A, S82.042A, S82.042B, S82.043B, S82.045A, S82.092A, S82.092B, S82.099A, S82.099C, S82.101C, S82.102B, S82.111B, S82.112C, S82.114C, S82.115B, S82.122B, S82.124B, S82.132B, S82.133C, S82.134B, S82.135B, S82.136A, S82.141A, S82.143A, S82.145A, S82.146B, S82.161A, S82.202A, S82.209C, S82.222A, S82.223A, S82.224A, S82.232B, S82.243A, S82.245A, S82.251A, S82.254A, S82.256B, S82.256C, S82.291B, S82.292A, S82.299A, S82.391C, S82.392C, S82.421A, S82.425A, S82.431C, S82.432B, S82.434C, S82.436A, S82.436B, S82.443B,

© 2023 Lin KJ et al. *JAMA Network Open.*

	S82.444A, S82.452A, S82.452B, S82.453A, S82.454A, S82.455B, S82.456A, S82.456B, S82.461B, S82.464A, S82.464B, S82.466C, S82.499A, S82.53XA, S82.54XB, S82.54XC, S82.56XC, S82.61XA, S82.62XA, S82.65XB, S82.66XA, S82.66XC, S82.832B, S82.843A, S82.843B, S82.845B, S82.851C, S82.852A, S82.852B, S82.853A, S82.853B, S82.854B, S82.856A, S82.863B, S82.864B, S82.871A, S82.871B, S82.871C, S82.875B, S82.876A, S82.899A, S82.899C, S82.90XB, S82.91XC, S82.92XA, S82.92XB, S89.039A, S89.092A, S89.099A, S89.112A, S89.139A, S89.199A, S89.221A, S89.291A, S89.301A, S92.021A, S92.022A, S92.023A, S92.025A, S92.032B, S92.033A, S92.034B, S92.036A, S92.041B, S92.043B, S92.044A, S92.046A, S92.055A, S92.063A, S92.065B, S92.109B, S92.116A, S92.123B, S92.124A, S92.125B, S92.133B, S92.135A, S92.136A, S92.136B, S92.142A, S92.142B, S92.144A, S92.144B, S92.145A, S92.153A, S92.153B, S92.155A, S92.156A, S92.202A, S92.202B, S92.212A, S92.213A, S92.215A, S92.224A, S92.225A, S92.225B, S92.226A, S92.231B, S92.233A, S92.233B, S92.235B, S92.243B, S92.244B, S92.252B, S92.253A, S92.256A, S92.312B, S92.313B, S92.316A, S92.323A, S92.331B, S92.336A, S92.341B, S92.342A, S92.344B, S92.346A, S92.352A, S92.354A, S92.355B, S92.403A, S92.403B, S92.404B, S92.412B, S92.413A, S92.415B, S92.424B, S92.491B, S92.492B, S92.503A, S92.512A, S92.512B, S92.531A, S92.901A, S92.911B, T14.8, M48.52XA, M80.022A, M80.032A, M80.039A, M80.042A, M80.049A, M80.059A, M80.061A, M80.062A, M80.069A, M80.071A, M80.072A, M80.821A, M80.829A, M80.831A, M80.842A, M80.861A, M80.869A, M80.879A, M84.412A, M84.419A, M84.429A, M84.432A, M84.442A, M84.444A, M84.451A, M84.459A, M84.476A, M84.50XA, M84.529A, M84.551A, M84.559A, M84.561A, M84.632A, M84.634A, M84.639A, M84.642A, M84.653A, M84.671A, S02.0XXB, S02.119B, S02.19XA, S02.400A, S02.400B, S02.402A, S02.412A, S02.413A, S02.92XA, S02.92XB, S06.337A, S06.339A, S06.4X0A, S06.4X5A, S06.6X3A, S06.6X7A, S06.896A, S06.9X1A, S06.9X5A, S06.9X9A, S12.000A, S12.02XA, S12.02XB, S12.030A, S12.090B, S12.091A, S12.091B, S12.100B, S12.110B, S12.131B, S12.14XB, S12.200B, S12.201B, S12.231A, S12.24XA, S12.300B, S12.331A, S12.390B, S12.431A, S12.44XA, S12.451A, S12.490A, S12.490B, S12.491A, S12.531A, S12.601A, S12.630A, S12.64XA, S12.64XB, S12.651B, S14.104A, S14.107A, S14.114A, S14.133A, S14.152A, S22.001A, S22.002A, S22.008A, S22.012A, S22.021B, S22.030A, S22.031B, S22.032A, S22.032B, S22.039B, S22.048A, S22.060B, S22.062B, S22.068A, S22.069B, S22.082B, S22.089A, S22.20XB, S22.21XB, S22.39XB, S24.112A, S24.154A, S32.000A, S32.001B, S32.022A, S32.028A, S32.049A, S32.050B, S32.059B, S32.10XB, S32.110A, S32.111B, S32.119B, S32.132A, S32.14XB, S32.17XB, S32.2XXB, S32.301A, S32.301B, S32.311A, S32.312B, S32.313A, S32.391A, S32.391B, S32.409B,

© 2023 Lin KJ et al. *JAMA Network Open.*

	S32.412B, S32.413A, S32.415B, S32.416A, S32.421A, S32.421B, S32.432A, S32.433B, S32.434B, S32.435B, S32.436B, S32.441A, S32.441B, S32.444B, S32.445B, S32.452B, S32.455A, S32.455B, S32.464B, S32.465B, S32.472A, S32.472B, S32.473A, S32.476B, S32.482B, S32.483A, S32.485B, S32.486A, S32.491B, S32.492A, S32.511B, S32.512A, S32.591A, S32.599A, S32.599B, S32.612A, S32.810A, S32.811B, S32.82XB, S32.89XB, S34.104A, S34.109A, S34.125A, S42.001A, S42.002A, S42.002B, S42.009A, S42.015A, S42.021A, S42.021B, S42.022A, S42.023A, S42.024A, S42.033A, S42.101A, S42.109B, S42.112B, S42.115B, S42.134A, S42.136A, S42.141B, S42.151A, S42.191A, S42.191B, S42.201B, S42.202A, S42.202B, S42.213B, S42.214A, S42.216A, S42.222A, S42.223A, S42.224A, S42.226B, S42.239B, S42.251A, S42.251B, S42.254B, S42.256A, S42.262A, S42.264B, S42.291B, S42.293B, S42.296A, S42.309B, S42.325A, S42.325B, S42.326B, S42.343B, S42.346B, S42.354A, S42.355B, S42.362A, S42.364A, S42.364B, S42.366B, S42.391A, S42.392A, S42.411A, S42.412A, S42.413B, S42.414A, S42.414B, S42.415B, S42.426A, S42.431B, S42.434A, S42.435B, S42.442A, S42.448B, S42.449A, S42.452A, S42.453A, S42.455A, S42.463B, S42.464A, S42.475A, S42.481A, S49.021A, S49.102A, S49.109A, S49.111A, S49.112A, S49.131A, S52.002C, S52.022B, S52.022C, S52.025B, S52.026B, S52.032A, S52.033B, S52.035B, S52.036A, S52.042C, S52.045C, S52.102B, S52.112A, S52.119A, S52.121A, S52.123B, S52.125B, S52.131B, S52.131C, S52.134A, S52.136C, S52.181B, S52.182A, S52.189A, S52.201C, S52.209C, S52.211A, S52.225C, S52.235C, S52.236A, S52.241C, S52.242B, S52.245A, S52.246C, S52.251C, S52.252B, S52.254A, S52.255C, S52.262C, S52.265A, S52.265C, S52.271A, S52.281C, S52.282A, S52.282B, S52.292C, S52.299B, S52.299C, S52.301A, S52.301B, S52.301C, S52.309A, S52.312A, S52.319A, S52.325C, S52.326C, S52.332C, S52.333A, S52.335B, S52.335C, S52.336C, S52.344C, S52.345C, S52.354B, S52.354C, S52.355A, S52.361A, S52.363B, S52.365A, S52.382A, S52.382C, S52.389B, S52.391C, S52.392A, S52.392B, S52.399C, S52.509A, S52.512A, S52.513C, S52.514B, S52.515C, S52.516C, S52.532B, S52.539B, S52.541C, S52.561C, S52.569A, S52.569B, S52.571C, S52.572C, S52.579B, S52.591B, S52.592C, S52.609A, S52.611B, S52.613C, S52.614B, S52.616A, S52.621A, S52.622A, S52.629A, S52.692A, S52.692C, S52.91XA, S52.92XA, S59.002A, S59.011A, S59.021A, S59.022A, S59.091A, S59.122A, S59.131A, S59.231A, S62.009B, S62.011B, S62.012A, S62.012B, S62.015A, S62.016B, S62.022B, S62.023B, S62.032A, S62.034A, S62.109B, S62.111A, S62.115A, S62.123B, S62.124A, S62.125B, S62.126B, S62.132B, S62.144A, S62.145B, S62.151B, S62.152B, S62.154B, S62.155A, S62.181B, S62.182B, S62.186A, S62.202B, S62.223B, S62.224A, S62.231B, S62.234B, S62.235A, S62.235B, S62.243A, S62.243B, S62.254B, S62.292A, S62.299A, S62.299B, S62.300B, S62.301B, S62.305B,

© 2023 Lin KJ et al. *JAMA Network Open.*

	S62.307A, S62.311A, S62.314A, S62.315A, S62.320A, S62.320B, S62.324B, S62.326B, S62.327B, S62.328A, S62.329A, S62.333A, S62.338A, S62.341A, S62.345B, S62.349A, S62.352B, S62.353A, S62.353B, S62.354A, S62.355A, S62.360A, S62.361A, S62.362A, S62.363A, S62.364A, S62.368B, S62.369A, S62.391A, S62.394A, S62.395B, S62.396B, S62.399B, S62.501A, S62.502B, S62.509A, S62.521A, S62.523A, S62.523B, S62.524A, S62.600A, S62.602A, S62.604A, S62.604B, S62.609B, S62.613A, S62.613B, S62.614A, S62.618A, S62.618B, S62.622B, S62.632A, S62.636A, S62.636B, S62.639B, S62.643A, S62.645A, S62.646A, S62.647B, S62.648B, S62.650A, S62.651A, S62.654B, S62.658B, S62.661A, S62.661B, S62.663A, S62.664A, S62.665B, S62.90XA, S72.001C, S72.021C, S72.022B, S72.025A, S72.025C, S72.031B, S72.031C, S72.032A, S72.033C, S72.034B, S72.041C, S72.042A, S72.043A, S72.044B, S72.044C, S72.052B, S72.061B, S72.063A, S72.064C, S72.066B, S72.109B, S72.109C, S72.112B, S72.113B, S72.114B, S72.121C, S72.122A, S72.124A, S72.124B, S72.126A, S72.132B, S72.134A, S72.135A, S72.141A, S72.143A, S72.144A, S72.145A, S72.145B, S72.146A, S72.22XC, S72.24XC, S72.25XA, S72.302C, S72.309C, S72.321B, S72.325A, S72.331A, S72.332A, S72.332C, S72.341C, S72.342A, S72.342B, S72.343C, S72.352B, S72.355C, S72.356A, S72.356C, S72.363A, S72.364A, S72.364B, S72.364C, S72.365B, S72.366A, S72.392A, S72.399A, S72.409C, S72.412C, S72.413A, S72.414B, S72.421A, S72.422A, S72.426B, S72.426C, S72.431A, S72.434A, S72.434B, S72.434C, S72.441A, S72.446B, S72.451A, S72.452C, S72.453B, S72.453C, S72.454C, S72.455B, S72.456A, S72.462A, S72.463C, S72.464B, S72.465A, S72.465C, S72.466A, S72.466B, S72.466C, S72.492B, S72.8X2B, S72.8X9C, S72.90XC, S72.91XB, S72.92XE, S79.009A, S79.092A, S79.119A, S79.132A, S79.142A, S79.199A, S82.009A, S82.011B, S82.012B, S82.014A, S82.021C, S82.022B, S82.022C, S82.026B, S82.035C, S82.036B, S82.045B, S82.046A, S82.046B, S82.091B, S82.092C, S82.099B, S82.101A, S82.109A, S82.112B, S82.113A, S82.114A, S82.115C, S82.121C, S82.123A, S82.123C, S82.125A, S82.125B, S82.126B, S82.126C, S82.131B, S82.136B, S82.144C, S82.146A, S82.152C, S82.154A, S82.191B, S82.199A, S82.199B, S82.201C, S82.209A, S82.225A, S82.226C, S82.241A, S82.241B, S82.242B, S82.252B, S82.252C, S82.253A, S82.254B, S82.254C, S82.255A, S82.255C, S82.262A, S82.264A, S82.264C, S82.266B, S82.292B, S82.292C, S82.302B, S82.311A, S82.391A, S82.422B, S82.422C, S82.423A, S82.423C, S82.424C, S82.425C, S82.432A, S82.442C, S82.443C, S82.451A, S82.451B, S82.452C, S82.453B, S82.462A, S82.462B, S82.462C, S82.463C, S82.465C, S82.491A, S82.491B, S82.499C, S82.53XC, S82.55XC, S82.64XB, S82.64XC, S82.839B, S82.841C, S82.844A, S82.852C, S82.855A, S82.855C, S82.856B, S82.861C, S82.862A, S82.863A, S82.863C, S82.866A, S82.866B, S82.874C, S82.875A, S82.876B, S82.892A, S82.899B,

© 2023 Lin KJ et al. *JAMA Network Open.*

	S82.91XA, S82.91XB, S89.009A, S89.019A, S89.021A, S89.111A, S89.119A, S89.192A, S89.212A, S89.222A, S89.229A, S89.312A, S89.322A, S89.329A, S89.391A, S89.399A, S92.009A, S92.011A, S92.012A, S92.012B, S92.013A, S92.014A, S92.014B, S92.015A, S92.023B, S92.024B, S92.031A, S92.033B, S92.035A, S92.041A, S92.045A, S92.052A, S92.052B, S92.053B, S92.054A, S92.056A, S92.065A, S92.066B, S92.101A, S92.111A, S92.113B, S92.122B, S92.123A, S92.124B, S92.132B, S92.134A, S92.134B, S92.135B, S92.141A, S92.143A, S92.143B, S92.152B, S92.191A, S92.191B, S92.192A, S92.192B, S92.199A, S92.199B, S92.215B, S92.216B, S92.226B, S92.244A, S92.255A, S92.302B, S92.315B, S92.322A, S92.332A, S92.332B, S92.344A, S92.351B, S92.356A, S92.356B, S92.412A, S92.421A, S92.425A, S92.426B, S92.506B, S92.511A, S92.513A, S92.513B, S92.515B, S92.516B, S92.522A, S92.523A, S92.524B, S92.531B, S92.533B, S92.902B, S92.912A, S92.919A, M48.54XA, M48.56XA, M80.00XA, M80.819A, M80.832A, M80.862A, M84.411A, M84.434A, M84.443A, M84.452A, M84.475A, M84.511A, M84.532A, M84.533A, M84.534A, M84.539A, M84.541A, M84.550A, M84.60XA, M84.612A, M84.629A, M84.641A, M84.659A, M84.662A, M84.663A, M84.669A, M84.674A, S02.110A, S02.112B, S02.401A, S02.401B, S02.411B, S02.412B, S02.413B, S02.42XA, S02.609A, S02.66XA, S02.66XB, S02.69XA, S06.330A, S06.331A, S06.362A, S06.363A, S06.367A, S06.4X2A, S06.4X9A, S06.6X2A, S06.6X9A, S06.894A, S06.898A, S06.9X2A, S06.9X8A, S12.01XA, S12.040B, S12.041B, S12.090A, S12.110A, S12.111B, S12.120A, S12.121A, S12.14XA, S12.150A, S12.151A, S12.191B, S12.250B, S12.301B, S12.34XB, S12.391A, S12.401A, S12.401B, S12.44XB, S12.500A, S12.501A, S12.54XA, S12.550A, S12.600A, S12.600B, S12.631B, S12.650A, S12.651A, S12.690B, S12.9XXA, S14.105A, S14.106A, S14.112A, S14.121A, S14.122A, S14.131A, S14.137A, S14.153A, S14.157A, S22.000A, S22.009A, S22.009B, S22.011B, S22.020A, S22.020B, S22.021A, S22.029B, S22.030B, S22.038B, S22.041A, S22.042B, S22.048B, S22.050A, S22.052B, S22.058A, S22.058B, S22.059A, S22.062A, S22.068B, S22.070A, S22.070B, S22.079B, S22.21XA, S22.23XA, S22.32XA, S22.41XA, S22.42XA, S24.109A, S24.113A, S24.153A, S32.000B, S32.001A, S32.002B, S32.008A, S32.008B, S32.009A, S32.011A, S32.018A, S32.028B, S32.030B, S32.032A, S32.039A, S32.042B, S32.051A, S32.051B, S32.058A, S32.058B, S32.10XA, S32.110B, S32.120B, S32.121B, S32.130A, S32.16XB, S32.19XB, S32.309B, S32.316B, S32.399B, S32.411B, S32.413B, S32.414A, S32.415A, S32.423A, S32.435A, S32.443A, S32.451A, S32.461A, S32.462A, S32.463A, S32.465A, S32.466B, S32.471A, S32.474B, S32.476A, S32.481A, S32.482A, S32.486B, S32.501A, S32.502A, S32.601A, S32.609A, S32.614A, S32.699A, S32.699B, S34.105A, S34.113A, S34.114A, S34.115A, S42.009B, S42.011A, S42.011B, S42.012B, S42.013B, S42.017B, S42.019A,

© 2023 Lin KJ et al. *JAMA Network Open.*

	S42.022B, S42.023B, S42.025B, S42.026B, S42.035A, S42.036A, S42.036B, S42.113A, S42.114A, S42.116B, S42.123B, S42.124A, S42.124B, S42.125A, S42.126A, S42.131A, S42.132A, S42.132B, S42.144B, S42.146A, S42.151B, S42.152B, S42.154B, S42.156A, S42.192A, S42.201A, S42.209A, S42.215A, S42.221B, S42.241B, S42.249A, S42.253B, S42.255B, S42.262B, S42.263A, S42.264A, S42.265A, S42.265B, S42.294A, S42.321A, S42.322A, S42.323B, S42.324A, S42.331A, S42.331B, S42.334A, S42.341B, S42.342B, S42.344A, S42.345B, S42.355A, S42.356B, S42.361B, S42.365A, S42.409B, S42.413A, S42.421B, S42.422B, S42.423B, S42.425A, S42.431A, S42.435A, S42.441A, S42.443B, S42.446B, S42.448A, S42.452B, S42.454A, S42.464B, S42.465A, S42.472A, S42.473B, S42.482A, S42.494B, S49.029A, S49.039A, S49.049A, S49.119A, S49.121A, S49.122A, S49.129A, S49.139A, S49.141A, S49.142A, S49.192A, S52.002B, S52.011A, S52.021B, S52.021C, S52.023A, S52.024A, S52.025A, S52.026A, S52.033A, S52.033C, S52.034C, S52.043A, S52.043B, S52.043C, S52.044C, S52.045B, S52.091A, S52.091C, S52.092A, S52.099A, S52.102A, S52.111A, S52.121C, S52.122A, S52.123A, S52.125C, S52.126B, S52.126C, S52.133A, S52.133C, S52.134B, S52.136B, S52.189C, S52.201A, S52.212A, S52.222B, S52.223A, S52.224A, S52.224C, S52.225A, S52.226A, S52.226B, S52.231B, S52.232C, S52.234B, S52.234C, S52.236B, S52.241A, S52.242C, S52.244B, S52.245C, S52.246A, S52.253C, S52.255A, S52.256B, S52.256C, S52.261A, S52.261C, S52.265B, S52.266C, S52.271B, S52.271C, S52.272C, S52.292B, S52.324A, S52.333C, S52.343C, S52.344B, S52.346B, S52.351B, S52.351C, S52.352A, S52.352B, S52.353A, S52.356A, S52.361B, S52.364A, S52.365B, S52.366A, S52.371C, S52.379C, S52.381A, S52.389C, S52.501B, S52.512C, S52.514C, S52.521A, S52.531A, S52.542A, S52.542B, S52.552C, S52.562A, S52.572A, S52.572B, S52.592B, S52.601C, S52.602A, S52.609B, S52.612A, S52.612C, S52.615C, S52.616B, S52.691A, S52.699C, S52.90XA, S52.90XB, S52.90XC, S52.92XC, S59.001A, S59.039A, S59.049A, S59.099A, S59.109A, S59.132A, S59.142A, S59.212A, S59.249A, S62.001B, S62.011A, S62.014A, S62.015B, S62.016A, S62.026B, S62.101A, S62.101B, S62.102A, S62.123A, S62.124B, S62.131A, S62.132A, S62.133A, S62.133B, S62.144B, S62.146B, S62.151A, S62.153A, S62.156A, S62.161B, S62.162A, S62.163A, S62.165B, S62.171B, S62.175B, S62.181A, S62.184A, S62.211B, S62.213B, S62.233A, S62.233B, S62.241A, S62.241B, S62.252B, S62.291A, S62.291B, S62.300A, S62.301A, S62.302A, S62.309B, S62.312B, S62.314B, S62.317A, S62.318B, S62.319A, S62.326A, S62.328B, S62.331A, S62.331B, S62.332B, S62.333B, S62.339B, S62.340A, S62.342A, S62.343A, S62.343B, S62.345A, S62.352A, S62.360B, S62.363B, S62.366A, S62.366B, S62.367B, S62.390A, S62.393B, S62.502A, S62.509B, S62.514A, S62.514B, S62.515B, S62.524B, S62.525B, S62.526A, S62.603B, S62.612A, S62.615B, S62.616A,

© 2023 Lin KJ et al. *JAMA Network Open.*

	S62.623A, S62.623B, S62.624B, S62.625A, S62.626A, S62.627A, S62.628A, S62.629A, S62.634A, S62.634B, S62.635A, S62.637B, S62.640B, S62.642A, S62.646B, S62.648A, S62.649A, S62.649B, S62.652B, S62.653A, S62.655B, S62.656B, S62.658A, S62.659A, S62.660B, S62.667A, S62.91XB, S72.001B, S72.002C, S72.011B, S72.012B, S72.022C, S72.024A, S72.024B, S72.025B, S72.032B, S72.033A, S72.035C, S72.036C, S72.043B, S72.045A, S72.046A, S72.061C, S72.066A, S72.091A, S72.092B, S72.111A, S72.112A, S72.112C, S72.114A, S72.115A, S72.116B, S72.121B, S72.123B, S72.134C, S72.135B, S72.135C, S72.141C, S72.146B, S72.301B, S72.302B, S72.321A, S72.322B, S72.322C, S72.335A, S72.336C, S72.343A, S72.344C, S72.345B, S72.345C, S72.346C, S72.353B, S72.354B, S72.354C, S72.355B, S72.356B, S72.362B, S72.363B, S72.363C, S72.391B, S72.402B, S72.411A, S72.411B, S72.411C, S72.412A, S72.414C, S72.415A, S72.415C, S72.416B, S72.422C, S72.423A, S72.423B, S72.425B, S72.431B, S72.432B, S72.432C, S72.435B, S72.435C, S72.442C, S72.444A, S72.444B, S72.445A, S72.446A, S72.455A, S72.455C, S72.461B, S72.491A, S72.491C, S72.492A, S72.8X1A, S72.8X1C, S72.8X2A, S72.8X9B, S72.90XA, S72.91XA, S72.92XC, S79.111A, S79.122A, S79.141A, S79.191A, S79.192A, S82.002C, S82.009B, S82.012A, S82.016C, S82.023A, S82.023B, S82.025B, S82.032B, S82.034C, S82.044B, S82.101B, S82.102C, S82.109B, S82.109C, S82.114B, S82.115A, S82.121A, S82.121B, S82.124A, S82.124C, S82.132C, S82.133A, S82.134A, S82.143B, S82.144A, S82.152A, S82.152B, S82.153B, S82.154C, S82.155C, S82.191C, S82.192B, S82.201A, S82.201B, S82.221B, S82.222B, S82.223C, S82.226B, S82.231B, S82.232C, S82.234A, S82.236C, S82.241C, S82.242C, S82.243C, S82.246A, S82.246B, S82.251C, S82.255B, S82.261C, S82.264B, S82.299B, S82.301A, S82.302A, S82.309A, S82.309B, S82.309C, S82.312A, S82.319A, S82.401A, S82.421B, S82.423B, S82.426A, S82.433A, S82.433C, S82.434B, S82.435B, S82.436C, S82.441C, S82.455A, S82.456C, S82.461C, S82.463B, S82.465A, S82.465B, S82.492B, S82.492C, S82.51XA, S82.52XA, S82.53XB, S82.55XA, S82.55XB, S82.56XA, S82.56XB, S82.61XB, S82.63XC, S82.819A, S82.829A, S82.839C, S82.841B, S82.842B, S82.844B, S82.853C, S82.856C, S82.862B, S82.864C, S82.866C, S82.873B, S82.891A, S82.891B, S82.891C, S82.892B, S82.90XC, S82.92XC, S89.002A, S89.012A, S89.032A, S89.041A, S89.049A, S89.102A, S89.109A, S89.122A, S89.149A, S89.201A, S89.209A, S89.219A, S89.309A, S89.392A, S92.002B, S92.009B, S92.015B, S92.016B, S92.031B, S92.035B, S92.036B, S92.042A, S92.043A, S92.044B, S92.045B, S92.056B, S92.061A, S92.102B, S92.109A, S92.111B, S92.114B, S92.116B, S92.121B, S92.146A, S92.151B, S92.155B, S92.201A, S92.211A, S92.211B, S92.216A, S92.222A, S92.222B, S92.223B, S92.241B, S92.246B, S92.253B, S92.254A, S92.254B, S92.255B, S92.301B, S92.302A, S92.312A, S92.314A, S92.321A, S92.322B,

© 2023 Lin KJ et al. *JAMA Network Open.*

	S92.323B, S92.324A, S92.324B, S92.325B, S92.333A, S92.336B, S92.342B, S92.343B, S92.345A, S92.345B, S92.346B, S92.353A, S92.355A, S92.401A, S92.402B, S92.405B, S92.414A, S92.414B, S92.415A, S92.416B, S92.422A, S92.504A, S92.505A, S92.514B, S92.515A, S92.521B, S92.523B, S92.525B, S92.526A, S92.534B, S92.536A, S92.592A, S92.599A, S92.909A, S92.909B, S92.912B, M48.55XA, M48.58XA, M80.021A, M80.031A, M80.041A, M80.08XA, M80.812A, M80.851A, M84.40XA, M84.422A, M84.431A, M84.439A, M84.441A, M84.445A, M84.446A, M84.453A, M84.454A, M84.461A, M84.462A, M84.464A, M84.471A, M84.479A, M84.48XA, M84.521A, M84.531A, M84.553A, M84.563A, M84.564A, M84.573A, M84.619A, M84.633A, M84.651A, M84.661A, M84.673A, M84.675A, M84.676A, S02.0XXA, S02.111A, S02.111B, S02.112A, S02.113B, S02.19XB, S02.402B, S02.600B, S06.332A, S06.336A, S06.338A, S06.4X3A, S06.4X6A, S06.4X7A, S06.5X1A, S06.5X6A, S06.5X8A, S06.6X0A, S06.6X5A, S06.6X6A, S06.890A, S06.893A, S06.9X3A, S06.9X6A, S12.000B, S12.001A, S12.031B, S12.112B, S12.121B, S12.130A, S12.151B, S12.230A, S12.230B, S12.24XB, S12.250A, S12.331B, S12.34XA, S12.350B, S12.390A, S12.391B, S12.450B, S12.451B, S12.54XB, S12.551A, S12.551B, S12.590A, S12.590B, S12.601B, S12.631A, S12.650B, S12.691B, S12.8XXA, S14.116A, S14.123A, S14.125A, S14.126A, S14.134A, S14.154A, S22.001B, S22.010A, S22.010B, S22.022A, S22.040B, S22.041B, S22.050B, S22.059B, S22.061B, S22.071B, S22.072A, S22.078B, S22.081A, S22.22XA, S22.23XB, S22.24XA, S22.39XA, S22.41XB, S22.43XA, S22.49XB, S24.101A, S24.111A, S24.131A, S32.009B, S32.010A, S32.012A, S32.018B, S32.019B, S32.021A, S32.029A, S32.029B, S32.038A, S32.042A, S32.048A, S32.048B, S32.050A, S32.112A, S32.120A, S32.121A, S32.122B, S32.129B, S32.131A, S32.16XA, S32.302A, S32.302B, S32.392A, S32.401B, S32.402B, S32.416B, S32.422A, S32.424A, S32.425A, S32.426B, S32.431A, S32.431B, S32.433A, S32.442B, S32.443B, S32.446A, S32.453B, S32.454A, S32.456B, S32.464A, S32.474A, S32.483B, S32.484B, S32.499B, S32.501B, S32.502B, S32.509A, S32.519A, S32.602B, S32.613B, S32.614B, S32.616A, S32.82XA, S34.112A, S34.123A, S42.013A, S42.014A, S42.016A, S42.024B, S42.025A, S42.026A, S42.032B, S42.033B, S42.034B, S42.035B, S42.115A, S42.121B, S42.122A, S42.123A, S42.125B, S42.126B, S42.135A, S42.135B, S42.141A, S42.143B, S42.145B, S42.146B, S42.153B, S42.213A, S42.222B, S42.231A, S42.231B, S42.232B, S42.242A, S42.271A, S42.291A, S42.293A, S42.294B, S42.295A, S42.295B, S42.301B, S42.312A, S42.326A, S42.333B, S42.334B, S42.335B, S42.336A, S42.341A, S42.345A, S42.346A, S42.351A, S42.352A, S42.352B, S42.353A, S42.354B, S42.356A, S42.362B, S42.365B, S42.399B, S42.401B, S42.411B, S42.416B, S42.433A, S42.436A, S42.436B, S42.441B, S42.442B, S42.444A, S42.444B, S42.449B, S42.451A,

© 2023 Lin KJ et al. *JAMA Network Open.*

	S42.454B, S42.456B, S42.462A, S42.463A, S42.474A, S42.476B, S42.491A, S42.492B, S42.495B, S42.91XA, S42.92XA, S42.92XB, S49.002A, S49.019A, S49.032A, S49.091A, S49.101A, S49.132A, S52.002A, S52.009B, S52.024B, S52.031B, S52.031C, S52.035A, S52.036C, S52.041A, S52.041C, S52.042A, S52.042B, S52.044B, S52.046A, S52.101C, S52.109A, S52.121B, S52.122B, S52.123C, S52.124A, S52.126A, S52.132B, S52.132C, S52.135A, S52.135B, S52.181A, S52.181C, S52.182B, S52.202A, S52.209B, S52.221A, S52.221B, S52.222C, S52.223B, S52.232B, S52.233B, S52.235A, S52.241B, S52.243A, S52.243B, S52.251B, S52.253B, S52.254B, S52.255B, S52.256A, S52.262A, S52.263A, S52.264A, S52.264C, S52.266B, S52.282C, S52.283A, S52.283B, S52.291C, S52.299A, S52.302A, S52.309B, S52.311A, S52.323B, S52.324C, S52.334B, S52.336B, S52.341C, S52.344A, S52.346A, S52.346C, S52.353B, S52.353C, S52.355B, S52.363C, S52.371A, S52.371B, S52.381B, S52.381C, S52.382B, S52.389A, S52.399B, S52.502B, S52.502C, S52.511A, S52.511B, S52.512B, S52.515B, S52.516A, S52.516B, S52.522A, S52.531B, S52.541B, S52.542C, S52.559C, S52.562B, S52.591A, S52.599A, S52.601A, S52.602B, S52.611A, S52.615A, S52.615B, S52.691C, S52.692B, S52.699B, S52.91XB, S59.019A, S59.031A, S59.092A, S59.119A, S59.139A, S59.141A, S59.192A, S59.202A, S59.222A, S59.232A, S59.241A, S59.242A, S59.292A, S62.002A, S62.002B, S62.022A, S62.024A, S62.025A, S62.033A, S62.034B, S62.111B, S62.112A, S62.114B, S62.116A, S62.125A, S62.134A, S62.135A, S62.141A, S62.142B, S62.143A, S62.143B, S62.145A, S62.146A, S62.165A, S62.166A, S62.171A, S62.172B, S62.173A, S62.174B, S62.176A, S62.176B, S62.184B, S62.201A, S62.211A, S62.212B, S62.221A, S62.222A, S62.223A, S62.226A, S62.226B, S62.234A, S62.245A, S62.245B, S62.253A, S62.253B, S62.256B, S62.309A, S62.310A, S62.310B, S62.312A, S62.313A, S62.313B, S62.315B, S62.316A, S62.318A, S62.319B, S62.322A, S62.323B, S62.330A, S62.330B, S62.338B, S62.339A, S62.341B, S62.350A, S62.354B, S62.358A, S62.364B, S62.365A, S62.368A, S62.390B, S62.392A, S62.392B, S62.393A, S62.396A, S62.397A, S62.398B, S62.513A, S62.516A, S62.522B, S62.525A, S62.526B, S62.606A, S62.608A, S62.610A, S62.611A, S62.616B, S62.617A, S62.617B, S62.619B, S62.621B, S62.622A, S62.626B, S62.627B, S62.630A, S62.631A, S62.633A, S62.633B, S62.635B, S62.638A, S62.641B, S62.650B, S62.651B, S62.654A, S62.656A, S62.657B, S62.663B, S62.664B, S62.666A, S62.668B, S62.90XB, S72.002A, S72.009C, S72.011A, S72.011C, S72.021A, S72.023C, S72.033B, S72.034A, S72.041A, S72.041B, S72.045C, S72.046C, S72.051A, S72.051B, S72.052A, S72.052C, S72.059A, S72.062A, S72.064B, S72.066C, S72.091C, S72.092C, S72.099A, S72.099B, S72.101C, S72.102B, S72.111B, S72.111C, S72.113C, S72.115C, S72.123A, S72.123C, S72.125B, S72.131A, S72.133B, S72.134B, S72.141B, S72.144B, S72.21XA, S72.21XB, S72.21XC,

© 2023 Lin KJ et al. *JAMA Network Open.*

	S72.22XB, S72.23XA, S72.23XC, S72.24XA, S72.301A, S72.321C, S72.322A, S72.323A, S72.323C, S72.331C, S72.334A, S72.334C, S72.335B, S72.342C, S72.344A, S72.344B, S72.345A, S72.351C, S72.352A, S72.361A, S72.361C, S72.362C, S72.366B, S72.366C, S72.391A, S72.399B, S72.401B, S72.402C, S72.409B, S72.413B, S72.416A, S72.416C, S72.421B, S72.425A, S72.425C, S72.426A, S72.433A, S72.433C, S72.436B, S72.441C, S72.442B, S72.443A, S72.461A, S72.462C, S72.463A, S72.464C, S72.465B, S72.472A, S72.479A, S72.492C, S72.8X2C, S72.90XB, S79.002A, S79.102A, S79.109A, S79.129A, S79.149A, S82.001B, S82.002A, S82.011A, S82.011C, S82.013C, S82.016A, S82.024B, S82.024C, S82.031A, S82.031C, S82.033B, S82.033C, S82.034B, S82.035A, S82.036C, S82.041A, S82.041C, S82.042C, S82.043A, S82.044A, S82.044C, S82.046C, S82.091A, S82.091C, S82.113B, S82.113C, S82.116A, S82.131A, S82.132A, S82.134C, S82.135A, S82.142B, S82.142C, S82.144B, S82.146C, S82.153A, S82.153C, S82.154B, S82.155B, S82.156A, S82.156C, S82.169A, S82.192A, S82.199C, S82.202B, S82.209B, S82.221A, S82.223B, S82.224B, S82.225B, S82.225C, S82.231A, S82.233C, S82.234B, S82.235A, S82.235B, S82.235C, S82.236A, S82.236B, S82.242A, S82.243B, S82.244A, S82.244B, S82.245B, S82.245C, S82.246C, S82.251B, S82.252A, S82.253C, S82.261A, S82.261B, S82.263B, S82.265A, S82.265B, S82.265C, S82.291C, S82.299C, S82.301B, S82.301C, S82.392B, S82.399B, S82.401B, S82.402B, S82.409B, S82.409C, S82.421C, S82.422A, S82.424A, S82.426C, S82.432C, S82.442A, S82.445B, S82.446A, S82.446B, S82.451C, S82.453C, S82.454B, S82.454C, S82.455C, S82.461A, S82.464C, S82.466B, S82.492A, S82.51XB, S82.51XC, S82.63XA, S82.63XB, S82.65XA, S82.65XC, S82.66XB, S82.821A, S82.831A, S82.831C, S82.832A, S82.832C, S82.841A, S82.842A, S82.843C, S82.844C, S82.845A, S82.845C, S82.846C, S82.851A, S82.851B, S82.855B, S82.864A, S82.865A, S82.872C, S82.874B, S89.011A, S89.042A, S89.101A, S89.142A, S89.202A, S89.311A, S89.321A, S92.001A, S92.011B, S92.013B, S92.016A, S92.022B, S92.025B, S92.051B, S92.053A, S92.054B, S92.055B, S92.061B, S92.062A, S92.064A, S92.064B, S92.101B, S92.102A, S92.113A, S92.115A, S92.115B, S92.125A, S92.126B, S92.131A, S92.131B, S92.132A, S92.133A, S92.141B, S92.145B, S92.152A, S92.154A, S92.154B, S92.156B, S92.201B, S92.212B, S92.214B, S92.221A, S92.223A, S92.232B, S92.234B, S92.235A, S92.242A, S92.242B, S92.245B, S92.246A, S92.251B, S92.256B, S92.311B, S92.313A, S92.314B, S92.321B, S92.326A, S92.331A, S92.335B, S92.341A, S92.343A, S92.353B, S92.401B, S92.402A, S92.404A, S92.405A, S92.406B, S92.411A, S92.413B, S92.422B, S92.423A, S92.424A, S92.425B, S92.491A, S92.492A, S92.499A, S92.501A, S92.501B, S92.502A, S92.504B, S92.506A, S92.514A, S92.516A, S92.521A, S92.524A, S92.532A, S92.533A, S92.591A, S92.592B, S92.599B, S92.901B, S92.902A, S92.919B, M48.53XA,

© 2023 Lin KJ et al. *JAMA Network Open.*

	M80.012A, M80.029A, M80.80XA, M80.822A, M80.841A, M80.859A, M84.421A, M84.474A, M84.477A, M84.512A, M84.522A, M84.549A, M84.562A, M84.571A, M84.572A, M84.574A, M84.576A, M84.611A, M84.622A, M84.650A, M84.664A, M84.672A, S02.113A, S02.118A, S02.118B, S02.2XXA, S02.42XB, S02.600A, S02.609B, S02.69XB, S02.91XA, S02.91XB, S06.333A, S06.334A, S06.360A, S06.364A, S06.365A, S06.366A, S06.368A, S06.369A, S06.4X1A, S06.4X8A, S06.5X0A, S06.5X2A, S06.5X5A, S06.5X7A, S06.5X9A, S06.891A, S06.895A, S06.897A, S06.9X0A, S06.9X4A, S06.9X7A, S12.001B, S12.01XB, S12.030B, S12.031A, S12.040A, S12.100A, S12.130B, S12.200A, S12.251A, S12.290A, S12.291A, S12.291B, S12.330A, S12.330B, S12.351A, S12.400A, S12.430A, S12.430B, S12.431B, S12.530A, S12.530B, S12.531B, S12.591A, S12.591B, S12.630B, S12.690A, S12.691A, S14.101A, S14.102A, S14.103A, S14.111A, S14.115A, S14.127A, S14.132A, S14.136A, S14.156A, S22.000B, S22.002B, S22.008B, S22.018A, S22.019B, S22.028B, S22.029A, S22.040A, S22.079A, S22.080A, S22.082A, S22.088A, S22.089B, S22.22XB, S22.31XA, S22.31XB, S22.42XB, S22.5XXA, S22.5XXB, S24.103A, S24.114A, S24.132A, S24.133A, S24.151A, S24.152A, S32.010B, S32.011B, S32.012B, S32.019A, S32.020A, S32.020B, S32.021B, S32.022B, S32.031A, S32.038B, S32.039B, S32.040A, S32.041A, S32.049B, S32.059A, S32.111A, S32.129A, S32.131B, S32.132B, S32.139A, S32.139B, S32.14XA, S32.17XA, S32.2XXA, S32.309A, S32.311B, S32.312A, S32.313B, S32.314A, S32.315B, S32.316A, S32.411A, S32.423B, S32.424B, S32.425B, S32.432B, S32.434A, S32.442A, S32.452A, S32.453A, S32.461B, S32.462B, S32.463B, S32.466A, S32.475B, S32.481B, S32.491A, S32.492B, S32.499A, S32.512B, S32.519B, S32.591B, S32.592A, S32.592B, S32.601B, S32.602A, S32.609B, S32.611A, S32.611B, S32.613A, S32.616B, S32.692B, S32.9XXA, S34.102A, S34.103A, S34.121A, S34.124A, S34.132A, S34.3XXA, S42.001B, S42.012A, S42.014B, S42.017A, S42.018B, S42.031B, S42.032A, S42.101B, S42.102B, S42.111B, S42.113B, S42.131B, S42.133B, S42.136B, S42.142B, S42.144A, S42.145A, S42.154A, S42.155B, S42.156B, S42.199A, S42.209B, S42.211A, S42.215B, S42.216B, S42.221A, S42.224B, S42.225B, S42.226A, S42.232A, S42.241A, S42.249B, S42.252A, S42.252B, S42.253A, S42.254A, S42.256B, S42.261B, S42.272A, S42.279A, S42.292B, S42.296B, S42.302A, S42.302B, S42.319A, S42.322B, S42.332B, S42.333A, S42.344B, S42.361A, S42.363A, S42.363B, S42.391B, S42.392B, S42.402A, S42.402B, S42.409A, S42.421A, S42.422A, S42.424B, S42.426B, S42.432B, S42.433B, S42.434B, S42.445B, S42.446A, S42.447B, S42.451B, S42.455B, S42.461A, S42.461B, S42.462B, S42.465B, S42.471A, S42.471B, S42.473A, S42.476A, S42.90XB, S42.91XB, S49.001A, S49.011A, S49.012A, S49.022A, S49.031A, S49.042A, S49.099A, S49.149A, S49.191A, S49.199A, S52.012A, S52.021A, S52.023B, S52.023C, S52.031A,

© 2023 Lin KJ et al. *JAMA Network Open.*

	S52.032C, S52.034A, S52.034B, S52.035C, S52.036B, S52.041B, S52.044A, S52.092B, S52.099B, S52.101A, S52.101B, S52.102C, S52.109B, S52.109C, S52.124B, S52.182C, S52.189B, S52.201B, S52.202B, S52.202C, S52.209A, S52.219A, S52.222A, S52.224B, S52.226C, S52.233A, S52.234A, S52.236C, S52.244A, S52.244C, S52.245B, S52.251A, S52.252C, S52.254C, S52.261B, S52.263C, S52.264B, S52.279A, S52.281B, S52.291A, S52.291B, S52.292A, S52.302B, S52.309C, S52.321B, S52.322A, S52.322C, S52.323A, S52.323C, S52.324B, S52.326B, S52.331A, S52.331C, S52.332B, S52.333B, S52.334C, S52.335A, S52.342B, S52.342C, S52.343A, S52.343B, S52.345B, S52.356C, S52.361C, S52.362C, S52.364B, S52.364C, S52.366B, S52.372C, S52.379A, S52.392C, S52.502A, S52.509B, S52.513B, S52.539A, S52.539C, S52.549A, S52.549B, S52.549C, S52.551B, S52.559A, S52.559B, S52.571B, S52.579A, S52.579C, S52.591C, S52.592A, S52.599B, S52.601B, S52.602C, S52.612B, S52.613B, S52.614A, S52.614C, S52.616C, S52.699A, S52.91XC, S59.009A, S59.012A, S59.041A, S59.101A, S59.112A, S59.129A, S59.149A, S59.211A, S59.229A, S59.239A, S59.291A, S59.299A, S62.001A, S62.009A, S62.014B, S62.023A, S62.025B, S62.026A, S62.032B, S62.035A, S62.036B, S62.102B, S62.112B, S62.113A, S62.115B, S62.121A, S62.121B, S62.122A, S62.122B, S62.126A, S62.131B, S62.134B, S62.135B, S62.136A, S62.142A, S62.152A, S62.154A, S62.156B, S62.163B, S62.173B, S62.182A, S62.183B, S62.185B, S62.209B, S62.222B, S62.225B, S62.231A, S62.232A, S62.244B, S62.246B, S62.255B, S62.256A, S62.302B, S62.303B, S62.304A, S62.306B, S62.308A, S62.308B, S62.311B, S62.317B, S62.321B, S62.325A, S62.332A, S62.336A, S62.336B, S62.340B, S62.342B, S62.346B, S62.347B, S62.348A, S62.348B, S62.355B, S62.356A, S62.356B, S62.357A, S62.361B, S62.362B, S62.365B, S62.367A, S62.391B, S62.398A, S62.399A, S62.511A, S62.512B, S62.513B, S62.515A, S62.521B, S62.522A, S62.601A, S62.602B, S62.603A, S62.605A, S62.605B, S62.607A, S62.608B, S62.609A, S62.610B, S62.611B, S62.612B, S62.614B, S62.615A, S62.620A, S62.620B, S62.621A, S62.624A, S62.628B, S62.630B, S62.631B, S62.637A, S62.639A, S62.640A, S62.642B, S62.643B, S62.645B, S62.647A, S62.652A, S62.657A, S62.659B, S62.660A, S62.662B, S62.665A, S62.667B, S62.669A, S62.91XA, S72.009A, S72.009B, S72.012A, S72.012C, S72.019C, S72.021B, S72.023A, S72.023B, S72.024C, S72.026A, S72.026B, S72.026C, S72.031A, S72.032C, S72.034C, S72.035B, S72.036B, S72.042B, S72.051C, S72.059B, S72.061A, S72.064A, S72.065B, S72.065C, S72.091B, S72.099C, S72.101A, S72.101B, S72.102A, S72.102C, S72.109A, S72.113A, S72.115B, S72.116A, S72.116C, S72.122C, S72.125A, S72.131C, S72.132C, S72.133C, S72.136C, S72.142B, S72.143B, S72.143C, S72.144C, S72.145C, S72.23XB, S72.24XB, S72.25XB, S72.25XC, S72.26XA, S72.309A, S72.309B, S72.323B, S72.324B, S72.324C, S72.326A, S72.326B, S72.326C,

© 2023 Lin KJ et al. *JAMA Network Open.*

	S72.336B, S72.341A, S72.346A, S72.353A, S72.353C, S72.355A, S72.361B, S72.365A, S72.365C, S72.391C, S72.399C, S72.401A, S72.409A, S72.412B, S72.414A, S72.415B, S72.423C, S72.424A, S72.424B, S72.424C, S72.431C, S72.435A, S72.442A, S72.443B, S72.443C, S72.444C, S72.451B, S72.451C, S72.452A, S72.452B, S72.454A, S72.454B, S72.456B, S72.456C, S72.461C, S72.462B, S72.464A, S72.471A, S72.491B, S72.499A, S72.499C, S72.8X1B, S72.91XC, S72.92XB, S79.001A, S79.012A, S79.019A, S79.101A, S79.112A, S79.121A, S82.001A, S82.001C, S82.002B, S82.009C, S82.013A, S82.014B, S82.014C, S82.015B, S82.015C, S82.024A, S82.025C, S82.026A, S82.034A, S82.035B, S82.036A, S82.041B, S82.043C, S82.045C, S82.102A, S82.111A, S82.111C, S82.112A, S82.116B, S82.116C, S82.122A, S82.122C, S82.123B, S82.125C, S82.126A, S82.131C, S82.133B, S82.135C, S82.136C, S82.141B, S82.141C, S82.142A, S82.143C, S82.145B, S82.145C, S82.151A, S82.151B, S82.151C, S82.155A, S82.156B, S82.162A, S82.191A, S82.192C, S82.202C, S82.221C, S82.222C, S82.224C, S82.226A, S82.231C, S82.232A, S82.233A, S82.233B, S82.234C, S82.244C, S82.253B, S82.256A, S82.262B, S82.262C, S82.263A, S82.263C, S82.266A, S82.266C, S82.291A, S82.302C, S82.391B, S82.392A, S82.399A, S82.399C, S82.401C, S82.402A, S82.402C, S82.409A, S82.424B, S82.425B, S82.426B, S82.431A, S82.431B, S82.433B, S82.434A, S82.435A, S82.435C, S82.441A, S82.441B, S82.442B, S82.443A, S82.444B, S82.444C, S82.445A, S82.445C, S82.446C, S82.463A, S82.466A, S82.491C, S82.499B, S82.52XB, S82.52XC, S82.54XA, S82.61XC, S82.62XB, S82.62XC, S82.64XA, S82.811A, S82.812A, S82.822A, S82.831B, S82.839A, S82.842C, S82.846A, S82.846B, S82.854A, S82.854C, S82.861A, S82.861B, S82.862C, S82.865B, S82.865C, S82.872A, S82.872B, S82.873A, S82.873C, S82.874A, S82.875C, S82.876C, S82.892C, S82.90XA, S89.001A, S89.022A, S89.029A, S89.031A, S89.091A, S89.121A, S89.129A, S89.131A, S89.132A, S89.141A, S89.191A, S89.211A, S89.292A, S89.299A, S89.302A, S89.319A, S92.001B, S92.002A, S92.021B, S92.024A, S92.026A, S92.026B, S92.032A, S92.034A, S92.042B, S92.046B, S92.051A, S92.062B, S92.063B, S92.066A, S92.112A, S92.112B, S92.114A, S92.121A, S92.122A, S92.126A, S92.146B, S92.151A, S92.209A, S92.209B, S92.213B, S92.214A, S92.221B, S92.224B, S92.231A, S92.232A, S92.234A, S92.236A, S92.236B, S92.241A, S92.243A, S92.245A, S92.251A, S92.252A, S92.301A, S92.309A, S92.309B, S92.311A, S92.315A, S92.316B, S92.325A, S92.326B, S92.333B, S92.334A, S92.334B, S92.335A, S92.351A, S92.352B, S92.354B, S92.406A, S92.411B, S92.416A, S92.421B, S92.423B, S92.426A, S92.499B, S92.502B, S92.503B, S92.505B, S92.511B, S92.522B, S92.525A, S92.526B, S92.532B, S92.534A, S92.535A, S92.535B, S92.536B, S92.591B, S92.911A

© 2023 Lin KJ et al. *JAMA Network Open.*

Major GI Bleeding	ICD-9 Diagnosis Code (any position) is any of: 430, 432.9, 568.81, 569.83, 569.86, 599.70, 599.71, 719.1, 719.10, 719.15, 719.18, 784.7, 784.8, 786.3, 786.30, 786.39, 423.0, 431, 432.0, 432.1, 459.0, 599.7, 599.72, 719.11, 719.12, 719.13, 719.14, 719.16, 719.17, 719.19, 786.31, 455.2, 455.5, 455.6, 455.7, 455.8, 456.0, 530.1, 530.11, 530.13, 530.19, 530.7, 531.3, 531.40, 531.5, 531.60, 531.61, 532.20, 532.3, 532.4, 532.41, 532.5, 532.6, 532.61, 532.9, 533.0, 533.00, 533.01, 533.20, 533.3, 533.40, 533.6, 533.60, 533.61, 533.7, 534.01, 534.2, 534.3, 534.4, 534.40, 534.41, 534.5, 534.6, 534.9, 535.00, 535.11, 535.20, 535.30, 535.41, 535.50, 535.61, 562.00, 562.02, 562.03, 562.10, 562.11, 562.12, 562.13, 569.3, 569.85, 578.1, 455.1, 455.4, 455.9, 456.20, 530.10, 530.12, 530.82, 531.0, 531.00, 531.01, 531.1, 531.2, 531.20, 531.21, 531.4, 531.41, 531.6, 531.7, 531.9, 532.0, 532.00, 532.01, 532.1, 532.2, 532.21, 532.40, 532.60, 532.7, 533.1, 533.2, 533.21, 533.4, 533.41, 533.5, 533.9, 534.0, 534.00, 534.1, 534.20, 534.21, 534.60, 534.61, 534.7, 535.01, 535.10, 535.21, 535.31, 535.40, 535.51, 535.60, 537.83, 562.01, 578.0, 578.9<br>ICD-10 Diagnosis Code (any position) is any of: I31.2, I60.01, I60.10, I60.11, I60.31, I60.51, I60.52, I60.6, I60.8, I61.4, I61.8, I62.1, K56.60, K57.33, K63.1, K66.1, M25.011, M25.012, M25.022, M25.032, M25.041, M25.042, M25.049, M25.059, M25.061, M25.062, M25.069, M25.071, M25.074, M25.076, M25.08, R04, R04.0, R04.1, R04.2, R04.8, R04.9, R31.0, R31.1, R31.2, R31.21, R31.9, R58, I60.00, I60.02, I60.12, I60.2, I60.30, I60.32, I60.4, I60.50, I60.7, I60.9, I61.0, I61.1, I61.2, I61.3, I61.5, I61.6, I61.9, I62.00, I62.01, I62.02, I62.03, I62.9, K57.13, K63.81, M25.00, M25.019, M25.021, M25.029, M25.031, M25.039, M25.051, M25.052, M25.072, M25.073, M25.075, R04.81, R04.89, R31, R31.29, I85.11, K20, K20.8, K21.0, K22.11, K22.6, K25.0, K25.4, K25.6, K26.1, K26.4, K26.5, K26.6, K27.2, K27.5, K27.6, K28.0, K28.4, K28.5, K29.01, K29.21, K29.41, K31.811, K55.21, K57.01, K57.21, K57.51, K57.53, K57.93, K64.0, K64.5, K64.8, K64.9, K92.0, I85.01, K20.0, K20.9, K25.1, K25.2, K25.5, K26.0, K26.2, K27.0, K27.1, K27.4, K28.1, K28.2, K28.6, K29.31, K29.51, K29.61, K29.71, K29.81, K29.91, K57.11, K57.31, K57.41, K57.81, K57.91, K62.5, K64.1, K64.2, K64.3, K64.4, K92.1, K92.2<br>ICD-9 Procedure Code (any position) is any of: 44.43<br>ICD-10 Procedure Code (any position) is any of: 0W3P8ZZ<br>HCPCS Procedure Code (any position) is any of: 43255
Home Oxygen Use	ICD-9 Diagnosis Code (any position) is any of: V46.2<br>ICD-10 Diagnosis Code (any position) is any of: Z99.81
Hypertension	ICD-9 Diagnosis Code (any position) is any of: 401.0, 401.1, 401.9, 402.0, 402.00, 402.01, 402.1, 402.10, 402.11, 402.9, 402.90, 402.91, 403.0, 403.00, 403.01, 403.1, 403.10, 403.11, 403.9, 403.90, 403.91, 404.0, 404.00, 404.01,

© 2023 Lin KJ et al. *JAMA Network Open.*

	404.02, 404.03, 404.1, 404.10, 404.11, 404.12, 404.13, 404.9, 404.90, 404.91, 404.92, 404.93, 405.0, 405.01, 405.09, 405.1, 405.11, 405.19, 405.9, 405.91, 405.99<br>ICD-10 Diagnosis Code (any position) is any of: I10, I13.2, I15.0, I15.8, I13.11, I13.10, I12.0, I13.0, I11.9, I12.9
Malignancy	ICD-9 Diagnosis Code (any position) is any of: 140.0, 140.1, 140.3, 140.4, 140.5, 140.6, 140.8, 140.9, 141.0, 141.1, 141.2, 141.3, 141.4, 141.5, 141.6, 141.8, 141.9, 142.0, 142.1, 142.2, 142.8, 142.9, 143.0, 143.1, 143.8, 143.9, 144.0, 144.1, 144.8, 144.9, 145.0, 145.1, 145.2, 145.3, 145.4, 145.5, 145.6, 145.8, 145.9, 146.0, 146.1, 146.2, 146.3, 146.4, 146.5, 146.6, 146.7, 146.8, 146.9, 147.0, 147.1, 147.2, 147.3, 147.8, 147.9, 148.0, 148.1, 148.2, 148.3, 148.8, 148.9, 149.0, 149.1, 149.8, 149.9, 150.0, 150.1, 150.2, 150.3, 150.4, 150.5, 150.8, 150.9, 151.0, 151.1, 151.2, 151.3, 151.4, 151.5, 151.6, 151.8, 151.9, 152.0, 152.1, 152.2, 152.3, 152.8, 152.9, 153.0, 153.1, 153.2, 153.3, 153.4, 153.5, 153.6, 153.7, 153.8, 153.9, 154.0, 154.1, 154.2, 154.3, 154.8, 155.0, 155.1, 155.2, 156.0, 156.1, 156.2, 156.8, 156.9, 157.0, 157.1, 157.2, 157.3, 157.4, 157.8, 157.9, 158.0, 158.8, 158.9, 159.0, 159.1, 159.8, 159.9, 160.0, 160.1, 160.2, 160.3, 160.4, 160.5, 160.8, 160.9, 161.0, 161.1, 161.2, 161.3, 161.8, 161.9, 162.0, 162.2, 162.3, 162.4, 162.5, 162.8, 162.9, 163.0, 163.1, 163.8, 163.9, 164.0, 164.1, 164.2, 164.3, 164.8, 164.9, 165.0, 165.8, 165.9, 170.0, 170.1, 170.2, 170.3, 170.4, 170.5, 170.6, 170.7, 170.8, 170.9, 171.0, 171.2, 171.3, 171.4, 171.5, 171.6, 171.7, 171.8, 171.9, 172.0, 172.1, 172.2, 172.3, 172.4, 172.5, 172.6, 172.7, 172.8, 172.9, 173.0, 173.00, 173.01, 173.02, 173.09, 173.1, 173.10, 173.11, 173.12, 173.19, 173.2, 173.20, 173.21, 173.22, 173.29, 173.3, 173.30, 173.31, 173.32, 173.39, 173.4, 173.40, 173.41, 173.42, 173.49, 173.5, 173.50, 173.51, 173.52, 173.59, 173.6, 173.60, 173.61, 173.62, 173.69, 173.7, 173.70, 173.71, 173.72, 173.79, 173.8, 173.80, 173.81, 173.82, 173.89, 173.9, 173.90, 173.91, 173.92, 173.99, 174.0, 174.1, 174.2, 174.3, 174.4, 174.5, 174.6, 174.8, 174.9, 175.0, 175.9, 176.0, 176.1, 176.2, 176.3, 176.4, 176.5, 176.8, 176.9, 180.0, 180.1, 180.8, 180.9, 182.0, 182.1, 182.8, 183.0, 183.2, 183.3, 183.4, 183.5, 183.8, 183.9, 184.0, 184.1, 184.2, 184.3, 184.4, 184.8, 184.9, 186.0, 186.9, 187.1, 187.2, 187.3, 187.4, 187.5, 187.6, 187.7, 187.8, 187.9, 188.0, 188.1, 188.2, 188.3, 188.4, 188.5, 188.6, 188.7, 188.8, 188.9, 189.0, 189.1, 189.2, 189.3, 189.4, 189.8, 189.9, 190.0, 190.1, 190.2, 190.3, 190.4, 190.5, 190.6, 190.7, 190.8, 190.9, 191.0, 191.1, 191.2, 191.3, 191.4, 191.5, 191.6, 191.7, 191.8, 191.9, 192.0, 192.1, 192.2, 192.3, 192.8, 192.9, 194.0, 194.1, 194.3, 194.4, 194.5, 194.6, 194.8, 194.9, 195.0, 195.1, 195.2, 195.3, 195.4, 195.5, 195.8, 196.0, 196.1, 196.2, 196.3, 196.5, 196.6, 196.8, 196.9, 197.0, 197.1, 197.2, 197.3, 197.4, 197.5, 197.6, 197.7, 197.8, 198.0, 198.1, 198.2, 198.3, 198.4, 198.5, 198.6, 198.7, 198.8,

© 2023 Lin KJ et al. *JAMA Network Open.*

	198.81, 198.82, 198.89, 199.0, 199.1, 199.2, 200.0, 200.00, 200.01, 200.02, 200.03, 200.04, 200.05, 200.06, 200.07, 200.08, 200.1, 200.10, 200.11, 200.12, 200.13, 200.14, 200.15, 200.16, 200.17, 200.18, 200.2, 200.20, 200.21, 200.22, 200.23, 200.24, 200.25, 200.26, 200.27, 200.28, 200.3, 200.30, 200.31, 200.32, 200.33, 200.34, 200.35, 200.36, 200.37, 200.38, 200.4, 200.40, 200.41, 200.42, 200.43, 200.44, 200.45, 200.46, 200.47, 200.48, 200.5, 200.50, 200.51, 200.52, 200.53, 200.54, 200.55, 200.56, 200.57, 200.58, 200.6, 200.60, 200.61, 200.62, 200.63, 200.64, 200.65, 200.66, 200.67, 200.68, 200.7, 200.70, 200.71, 200.72, 200.73, 200.74, 200.75, 200.76, 200.77, 200.78, 200.8, 200.80, 200.81, 200.82, 200.83, 200.84, 200.85, 200.86, 200.87, 200.88, 201.0, 201.00, 201.01, 201.02, 201.03, 201.04, 201.05, 201.06, 201.07, 201.08, 201.1, 201.10, 201.11, 201.12, 201.13, 201.14, 201.15, 201.16, 201.17, 201.18, 201.2, 201.20, 201.21, 201.22, 201.23, 201.24, 201.25, 201.26, 201.27, 201.28, 201.4, 201.40, 201.41, 201.42, 201.43, 201.44, 201.45, 201.46, 201.47, 201.48, 201.5, 201.50, 201.51, 201.52, 201.53, 201.54, 201.55, 201.56, 201.57, 201.58, 201.6, 201.60, 201.61, 201.62, 201.63, 201.64, 201.65, 201.66, 201.67, 201.68, 201.7, 201.70, 201.71, 201.72, 201.73, 201.74, 201.75, 201.76, 201.77, 201.78, 201.9, 201.90, 201.91, 201.92, 201.93, 201.94, 201.95, 201.96, 201.97, 201.98, 202.0, 202.00, 202.01, 202.02, 202.03, 202.04, 202.05, 202.06, 202.07, 202.08, 202.1, 202.10, 202.11, 202.12, 202.13, 202.14, 202.15, 202.16, 202.17, 202.18, 202.2, 202.20, 202.21, 202.22, 202.23, 202.24, 202.25, 202.26, 202.27, 202.28, 202.3, 202.30, 202.31, 202.32, 202.33, 202.34, 202.35, 202.36, 202.37, 202.38, 202.4, 202.40, 202.41, 202.42, 202.43, 202.44, 202.45, 202.46, 202.47, 202.48, 202.5, 202.50, 202.51, 202.52, 202.53, 202.54, 202.55, 202.56, 202.57, 202.58, 202.6, 202.60, 202.61, 202.62, 202.63, 202.64, 202.65, 202.66, 202.67, 202.68, 202.7, 202.70, 202.71, 202.72, 202.73, 202.74, 202.75, 202.76, 202.77, 202.78, 202.8, 202.80, 202.81, 202.82, 202.83, 202.84, 202.85, 202.86, 202.87, 202.88, 202.9, 202.90, 202.91, 202.92, 202.93, 202.94, 202.95, 202.96, 202.97, 202.98, 203.0, 203.00, 203.01, 203.02, 203.1, 203.10, 203.11, 203.12, 203.8, 203.80, 203.81, 203.82, 204.0, 204.00, 204.01, 204.02, 204.1, 204.10, 204.11, 204.12, 204.2, 204.20, 204.21, 204.22, 204.8, 204.80, 204.81, 204.82, 204.9, 204.90, 204.91, 204.92, 205.0, 205.00, 205.01, 205.02, 205.1, 205.10, 205.11, 205.12, 205.2, 205.20, 205.21, 205.22, 205.3, 205.30, 205.31, 205.32, 205.8, 205.80, 205.81, 205.82, 205.9, 205.90, 205.91, 205.92, 206.0, 206.00, 206.01, 206.02, 206.1, 206.10, 206.11, 206.12, 206.2, 206.20, 206.21, 206.22, 206.8, 206.80, 206.81, 206.82, 206.9, 206.90, 206.91, 206.92, 207.0, 207.00, 207.01, 207.02, 207.1, 207.10, 207.11, 207.12, 207.2, 207.20, 207.21, 207.22, 207.8, 207.80, 207.81, 207.82, 208.0, 208.00, 208.01, 208.02, 208.1, 208.10, 208.11, 208.12, 208.2, 208.20, 208.21, 208.22, 208.8, 208.80, 208.81, 208.82, 208.9, 208.90, 208.91, 208.92

© 2023 Lin KJ et al. *JAMA Network Open.*

	ICD-10 Diagnosis Code (any position) is any of: C00.1, C00.6, C02.0, C02.1, C02.3, C04.9, C05, C05.0, C05.1, C06, C07, C09.0, C09.9, C10, C10.0, C10.2, C10.3, C10.4, C11.8, C13.0, C15.3, C16, C16.0, C16.4, C16.5, C17.9, C26, C26.9, C32, C32.9, C34.0, C34.00, C34.11, C34.12, C34.2, C34.3, C34.80, C34.82, C34.91, C38.1, C38.3, C38.8, C40.02, C40.10, C40.3, C40.91, C41.2, C41.3, C43.11, C43.21, C43.30, C43.4, C43.60, C43.62, C45.7, C46.4, C46.5, C46.52, C47, C47.20, C47.6, C48.0, C48.1, C49.1, C49.10, C49.11, C49.12, C49.20, C50, C50.012, C50.02, C50.112, C50.12, C50.121, C50.129, C50.229, C50.31, C50.319, C50.41, C50.411, C50.422, C50.611, C50.91, C50.912, C50.919, C50.921, C51.9, C54.8, C56, C56.9, C57.00, C57.01, C57.1, C57.11, C57.2, C57.22, C57.3, C57.4, C57.9, C60.8, C61, C62.00, C62.01, C62.02, C62.10, C63.7, C66.9, C67, C67.9, C69.00, C69.11, C69.31, C69.32, C69.4, C69.40, C69.5, C69.51, C69.62, C69.9, C69.90, C70.9, C71.6, C71.9, C72.0, C72.22, C72.3, C74.0, C74.91, C75, C76, C76.2, C76.3, C76.50, C81.0, C81.05, C81.10, C81.12, C81.20, C81.21, C81.28, C81.93, C82.00, C82.01, C82.02, C82.07, C82.08, C82.21, C82.29, C82.3, C82.31, C82.32, C82.41, C82.44, C82.45, C82.47, C82.48, C82.60, C82.80, C82.84, C82.88, C82.95, C82.97, C83.04, C83.13, C83.16, C83.18, C83.19, C83.30, C83.34, C83.37, C83.50, C83.51, C83.56, C83.57, C83.58, C83.59, C83.8, C83.80, C83.92, C84.0, C84.08, C84.09, C84.42, C84.46, C84.74, C84.75, C84.76, C84.92, C84.96, C85.20, C85.27, C85.80, C85.83, C85.86, C85.89, C85.90, C85.97, C90.2, C95, C95.0, C95.02, C96.2, C96.9, C96.Z, D45, C00.2, C00.4, C00.5, C02.2, C02.9, C03, C04, C06.0, C06.8, C08.0, C08.9, C09, C09.1, C10.1, C10.9, C13.2, C13.9, C14.0, C15.4, C16.1, C16.3, C17.1, C17.3, C18.1, C18.8, C18.9, C20, C21.2, C22.1, C22.2, C22.8, C24, C24.0, C24.8, C25.2, C26.0, C31.0, C31.9, C32.2, C34.02, C34.8, C34.9, C34.90, C34.92, C37, C38.2, C39, C39.0, C40.0, C40.92, C41.0, C43.3, C43.39, C43.51, C43.52, C43.72, C43.8, C45.1, C45.2, C46.0, C47.10, C47.2, C48.2, C49.2, C49.8, C50.019, C50.11, C50.221, C50.32, C50.321, C50.519, C50.529, C50.629, C50.81, C50.82, C50.822, C50.829, C51.2, C53, C54.0, C55, C57.21, C57.8, C62.11, C63.00, C63.10, C63.11, C64.1, C65.1, C65.2, C66.1, C67.3, C67.6, C67.7, C68.0, C68.1, C69.2, C69.52, C69.82, C69.91, C71.4, C71.8, C72.1, C72.20, C72.21, C72.4, C72.40, C73, C74.1, C74.90, C75.0, C75.2, C75.5, C75.8, C75.9, C76.40, C76.42, C81.22, C81.23, C81.31, C81.36, C81.44, C81.70, C81.94, C81.96, C81.97, C82.0, C82.03, C82.14, C82.15, C82.25, C82.33, C82.38, C82.40, C82.42, C82.46, C82.53, C82.6, C82.62, C82.65, C82.89, C82.98, C82.99, C83, C83.07, C83.09, C83.12, C83.32, C83.39, C83.53, C83.7, C83.70, C83.74, C83.79, C83.84, C83.85, C83.89, C83.96, C84.01, C84.03, C84.1, C84.11, C84.13, C84.14, C84.19, C84.61, C84.62, C84.71, C84.72, C84.77, C84.79, C84.97, C84.A1, C84.A7, C84.A9, C84.Z3, C84.Z6, C84.Z7,

© 2023 Lin KJ et al. *JAMA Network Open.*

	C85.1, C85.10, C85.12, C85.14, C85.15, C85.21, C85.25, C85.28, C85.85, C85.88, C85.91, C88.8, C88.9, C90.0, C94.32, C94.8, C94.80, C94.82, C95.90, C96.4, C96.A, C00, C02.8, C03.9, C04.0, C04.8, C06.80, C08, C11, C11.0, C11.2, C13, C14, C15.8, C17.2, C18.0, C18.3, C18.4, C18.6, C18.7, C21.1, C22.0, C24.9, C25.0, C25.1, C25.8, C26.1, C30.1, C31.2, C31.8, C32.0, C32.1, C32.3, C32.8, C33, C34.10, C34.31, C34.32, C38, C38.0, C40.00, C40.12, C40.20, C40.21, C40.30, C40.31, C40.82, C41, C41.9, C43.10, C43.5, C43.59, C43.6, C43.9, C45.9, C46.2, C47.21, C47.22, C47.5, C47.8, C48, C49, C49.0, C49.4, C50.0, C50.011, C50.021, C50.029, C50.111, C50.122, C50.211, C50.219, C50.22, C50.3, C50.311, C50.322, C50.329, C50.4, C50.419, C50.42, C50.5, C50.522, C50.612, C50.622, C50.819, C50.821, C50.9, C50.911, C50.922, C51, C51.1, C52, C54, C54.1, C56.1, C57, C57.12, C57.20, C57.7, C58, C60, C60.9, C62.1, C63.0, C63.01, C63.8, C64, C64.2, C64.9, C67.0, C67.5, C68.8, C68.9, C69, C69.0, C69.3, C69.41, C69.60, C69.80, C69.81, C69.92, C70.0, C71.1, C71.3, C71.7, C72.2, C72.42, C72.9, C74.10, C75.3, C76.41, C76.5, C76.52, C81.02, C81.04, C81.06, C81.18, C81.19, C81.27, C81.29, C81.40, C81.41, C81.43, C81.45, C81.72, C81.73, C81.74, C81.76, C81.91, C81.95, C81.98, C82.05, C82.09, C82.10, C82.19, C82.20, C82.26, C82.27, C82.36, C82.52, C82.54, C82.55, C82.56, C82.61, C82.66, C82.69, C82.8, C82.86, C82.87, C82.90, C83.0, C83.02, C83.08, C83.1, C83.11, C83.31, C83.81, C83.86, C83.88, C83.90, C83.97, C83.98, C84.04, C84.05, C84.12, C84.47, C84.65, C84.67, C84.78, C84.9, C84.A3, C84.A4, C84.A5, C84.A8, C84.Z0, C84.Z4, C84.Z9, C85.13, C85.17, C85.24, C85.81, C85.87, C85.94, C85.99, C88.2, C88.3, C94.3, C95.1, C95.10, C95.9, C95.92, D89, C02, C02.4, C03.0, C05.2, C05.9, C06.1, C06.2, C06.89, C06.9, C08.1, C11.1, C11.3, C11.9, C14.2, C15, C15.5, C15.9, C16.2, C16.8, C17, C18, C18.5, C21.0, C21.8, C22.3, C22.7, C23, C25.3, C25.9, C30, C31.1, C38.4, C40.1, C40.22, C40.8, C40.9, C41.1, C41.4, C43.1, C43.12, C43.20, C43.22, C43.31, C43.71, C45.0, C46.1, C46.3, C46.51, C47.0, C47.1, C47.12, C47.9, C49.21, C49.5, C49.9, C50.022, C50.21, C50.212, C50.222, C50.312, C50.429, C50.511, C50.52, C50.6, C50.61, C50.621, C50.92, C50.929, C51.0, C51.8, C53.1, C53.9, C57.10, C60.0, C60.1, C60.2, C62.90, C62.92, C63.02, C63.1, C63.2, C63.9, C65.9, C66, C67.1, C67.2, C67.4, C68, C69.01, C69.1, C69.21, C69.22, C69.61, C69.8, C71, C71.2, C72.30, C72.31, C72.50, C72.59, C74.02, C74.11, C75.1, C75.4, C76.1, C76.51, C81.00, C81.07, C81.11, C81.13, C81.14, C81.15, C81.16, C81.17, C81.2, C81.25, C81.38, C81.39, C81.42, C81.46, C81.48, C81.49, C81.7, C81.71, C81.75, C81.77, C81.99, C82, C82.04, C82.06, C82.13, C82.17, C82.22, C82.23, C82.30, C82.59, C82.64, C82.67, C82.81, C82.83, C82.85, C82.91, C82.92, C82.93, C82.96, C83.00, C83.01, C83.05, C83.15, C83.17, C83.3, C83.33, C83.36, C83.38,

© 2023 Lin KJ et al. *JAMA Network Open.*

	C83.5, C83.54, C83.71, C83.73, C83.77, C83.78, C83.87, C83.95, C83.99, C84, C84.00, C84.02, C84.17, C84.41, C84.44, C84.48, C84.6, C84.63, C84.66, C84.70, C84.91, C84.93, C84.95, C84.A, C84.A2, C85.18, C85.19, C85.22, C85.23, C85.8, C85.82, C85.84, C85.9, C85.92, C85.93, C85.96, C85.98, C94.30, C94.31, C95.00, C95.12, C00.0, C00.3, C00.8, C00.9, C01, C03.1, C04.1, C05.8, C09.8, C10.8, C12, C13.1, C13.8, C14.8, C16.6, C16.9, C17.0, C17.8, C18.2, C19, C21, C22, C22.4, C22.9, C24.1, C25, C25.4, C25.7, C30.0, C31, C31.3, C34, C34.01, C34.1, C34.30, C34.81, C39.9, C40, C40.01, C40.11, C40.2, C40.32, C40.80, C40.81, C40.90, C43.0, C43.2, C43.61, C43.7, C43.70, C46, C46.50, C46.7, C46.9, C47.11, C47.3, C47.4, C48.8, C49.22, C49.3, C49.6, C50.01, C50.1, C50.119, C50.2, C50.412, C50.421, C50.51, C50.512, C50.521, C50.619, C50.62, C50.8, C50.811, C50.812, C53.0, C53.8, C54.2, C54.3, C54.9, C56.2, C57.0, C57.02, C62, C62.0, C62.12, C62.9, C62.91, C63, C63.12, C65, C66.2, C67.8, C69.02, C69.10, C69.12, C69.20, C69.30, C69.42, C69.50, C69.6, C70, C70.1, C71.0, C71.5, C72, C72.32, C72.41, C72.5, C74, C74.00, C74.01, C74.12, C74.9, C74.92, C76.0, C76.4, C76.8, C81, C81.01, C81.03, C81.08, C81.09, C81.1, C81.24, C81.26, C81.3, C81.30, C81.32, C81.33, C81.34, C81.35, C81.37, C81.4, C81.47, C81.78, C81.79, C81.9, C81.90, C81.92, C82.1, C82.11, C82.12, C82.16, C82.18, C82.2, C82.24, C82.28, C82.34, C82.35, C82.37, C82.39, C82.4, C82.43, C82.49, C82.5, C82.50, C82.51, C82.57, C82.58, C82.63, C82.68, C82.82, C82.9, C82.94, C83.03, C83.06, C83.10, C83.14, C83.35, C83.52, C83.55, C83.72, C83.75, C83.76, C83.82, C83.83, C83.9, C83.91, C83.93, C83.94, C84.06, C84.07, C84.10, C84.15, C84.16, C84.18, C84.4, C84.40, C84.43, C84.45, C84.49, C84.60, C84.64, C84.68, C84.69, C84.7, C84.73, C84.90, C84.94, C84.98, C84.99, C84.A0, C84.A6, C84.Z, C84.Z1, C84.Z2, C84.Z5, C84.Z8, C85.11, C85.16, C85.2, C85.26, C85.29, C85.95, C88.0, C88.4, C94.81, C95.01, C95.11, C95.91, C96.0, C96.5, C96.6, Z85.46
Ischemic Heart Disease	ICD-9 Diagnosis Code (any position) is any of: 410.1, 410.10, 410.11, 410.20, 410.31, 410.7, 410.70, 410.71, 410.9, 410.92, 411.1, 411.81, 413.0, 414.0, 414.00, 414.01, 414.02, 414.03, 414.06, 410.21, 410.22, 410.41, 410.61, 410.72, 411.89, 412, 413.9, 414.12, 414.2, 410.0, 410.00, 410.3, 410.32, 410.4, 410.42, 410.5, 410.62, 410.80, 410.81, 410.82, 411.0, 414.11, 414.3, 414.8, 410.01, 410.02, 410.12, 410.2, 410.30, 410.51, 410.52, 410.90, 410.91, 411.8, 414.05, 414.07, 414.10, 414.19, 414.4, 414.9, 410.40, 410.50, 410.6, 410.60, 410.8, 413.1, 414.04, 414.1<br>ICD-10 Diagnosis Code (any position) is any of: I20.0, I21.02, I21.09, I21.19, I21.29, I22.2, I22.8, I24.9, I25.111, I25.119, I25.5, I25.708, I25.710, I25.738, I25.751, I25.84, I20.8, I21.11, I24.1, I25.2, I25.719, I25.721, I25.728, I25.731, I25.758, I25.760, I25.761, I25.791, I25.811, I25.82, I25.89, I25.9, I21.4, I22.9,

© 2023 Lin KJ et al. *JAMA Network Open.*

	I24.0, I24.8, I25.3, I25.42, I25.6, I25.750, I25.812, I25.83, I20.9, I21.3, I22.0, I22.1, I25.118, I25.701, I25.709, I25.718, I25.730, I25.768, I25.769, I25.790, I25.798, I25.799, I20.1, I21.01, I21.21, I25.10, I25.110, I25.41, I25.700, I25.711, I25.720, I25.729, I25.739, I25.759, I25.810
Peripheral Vascular Disease	HCPCS Procedure Code (any position) is any of: 35256, 35286, 35363, 35459, 35492, 35556, 35582, 35583, 35646, 35661, 35681, 37207, 37222, 37226, 37227, 37229, 37230, 35351, 35371, 35454, 35470, 35485, 35495, 35533, 35546, 35551, 35585, 35647, 35666, 35671, 35682, 37228, 35355, 35473, 35541, 35548, 35563, 35637, 35641, 35651, 35654, 35656, 37208, 37225, 37235, 35549, 35558, 35565, 35566, 35570, 35571, 35587, 35638, 35663, 35683, 37220, 37232, 37234, 35361, 35372, 35381, 35456, 35474, 35482, 35483, 35493, 35521, 35621, 35623, 35879, 37221, 37223, 37224, 37231, 37233<br>ICD-9 Procedure Code (any position) is any of: 38.18, 38.49, 39.50, 39.55, 39.59, 39.9, 39.91, 39.94, 39.97, 39.29, 39.51, 39.52, 39.53, 39.54, 39.58, 39.90, 39.92, 39.99, 38.08, 38.09, 38.38, 38.48, 39.5, 39.56, 39.93, 39.96, 38.39, 39.25, 39.57, 39.95, 39.98<br>ICD-10 Procedure Code (any position) is any of: 0410098, 041009B, 041009K, 04100A8, 04100AG, 04100J6, 04100JH, 04100JJ, 04100KB, 04100KJ, 04100Z7, 04100Z8, 04100ZC, 04100ZD, 04100ZJ, 0410497, 041049C, 041049D, 041049H, 041049J, 04104A7, 04104A8, 04104AR, 04104J6, 04104JB, 04104JK, 04104K8, 04104Z9, 04104ZD, 04104ZK, 041C0JH, 041C0KK, 041C49K, 041C4AH, 041C4KJ, 041D09J, 041D0AK, 041D0JJ, 041D0KJ, 041D49H, 041D4AK, 041E0KJ, 041E0ZH, 041E49K, 041E4JH, 041E4JJ, 041E4JK, 041E4ZK, 041F0AH, 041F0AK, 041F49H, 041F4AJ, 041F4JH, 041F4KH, 041F4ZJ, 041F4ZK, 041H09H, 041H0AJ, 041H0JK, 041H0ZK, 041H4AH, 041H4JJ, 041H4JK, 041H4ZH, 041J09H, 041J09J, 041J0AJ, 041J0ZJ, 041J4KK, 041K09H, 041K09J, 041K09M, 041K0AH, 041K0AK, 041K0AQ, 041K0JJ, 041K0JK, 041K0JN, 041K0KK, 041K0KM, 041K0KQ, 041K0ZH, 041K49H, 041K49N, 041K4AH, 041K4AN, 041K4AP, 041K4AQ, 041K4AS, 041K4KH, 041K4ZH, 041K4ZL, 041L0AH, 041L0JS, 041L0KH, 041L0KK, 041L0KP, 041L0ZM, 041L49K, 041L4AL, 041L4AM, 041L4AQ, 041L4JP, 041L4KK, 041L4KS, 041L4ZQ, 041M0ZS, 041M4AS, 041M4ZQ, 041M4ZS, 041N09S, 041N0AM, 041N0AS, 041N49P, 041N4AM, 041N4JM, 041N4KP, 041N4ZM, 0470046, 0470056, 0470066, 047007Z, 0470346, 04703GZ, 047044Z, 0470456, 04704F6, 04704FZ, 04704GZ, 047C05Z, 047C07Z, 047C376, 047C3E6, 047C3EZ, 047C3G6, 047C4D6, 047C4DZ, 047C4E6, 047C4EZ, 047C4GZ, 047C4Z6, 047C4ZZ, 047D0F6, 047D0G6, 047D3DZ, 047D3EZ, 047D4E6, 047D4G6, 047E07Z, 047E0EZ, 047E0F6, 047E0FZ, 047E0Z6, 047E346, 047E37Z, 047E3E6, 047E3EZ,

© 2023 Lin KJ et al. *JAMA Network Open.*

	047E3FZ, 047E44Z, 047E466, 047E4EZ, 047E4F6, 047E4GZ, 047E4ZZ, 047F066, 047F07Z, 047F0DZ, 047F0G6, 047F0Z6, 047F35Z, 047F376, 047F446, 047F476, 047F4Z6, 047F4ZZ, 047H046, 047H05Z, 047H06Z, 047H0Z6, 047H0ZZ, 047H3DZ, 047H3E6, 047H466, 047H4G6, 047H4Z6, 047J066, 047J06Z, 047J0DZ, 047J0FZ, 047J34Z, 047J3FZ, 047J3G6, 047J3GZ, 047J3ZZ, 047J466, 047K056, 047K06Z, 047K0E6, 047K0G6, 047K0Z1, 047K0Z6, 047K0ZZ, 047K341, 047K376, 047K3D6, 047K3E6, 047K3FZ, 047K3Z6, 047K45Z, 047K46Z, 047K476, 047K4D1, 047K4DZ, 047K4E6, 047K4EZ, 047K4F6, 047L041, 047L0D1, 047L0E6, 047L0GZ, 047L0Z6, 047L341, 047L34Z, 047L36Z, 047L3Z1, 047L446, 047L4D1, 047L4GZ, 047L4ZZ, 047M05Z, 047M0D1, 047M0EZ, 047M35Z, 047M3D6, 047M3Z1, 047M3Z6, 047M3ZZ, 047M466, 047M4EZ, 047M4G6, 047M4Z1, 047M4Z6, 047N056, 047N346, 047N36Z, 047N3E6, 047N3GZ, 047N3Z6, 047N45Z, 047N4DZ, 047N4E6, 047N4Z1, 047P04Z, 047P066, 047P076, 047P07Z, 047P36Z, 047P3DZ, 047P3E6, 047P3G6, 047P3GZ, 047P476, 047P4DZ, 047P4G6, 047P4ZZ, 047Q066, 047Q06Z, 047Q3FZ, 047Q47Z, 047Q4D6, 047Q4Z6, 047R066, 047R0EZ, 047R0F6, 047R0GZ, 047R376, 047R3D6, 047R3G6, 047R3GZ, 047R446, 047R456, 047R4ZZ, 047S076, 047S07Z, 047S0EZ, 047S0G6, 047S34Z, 047S356, 047S35Z, 047S37Z, 047S3D6, 047S3FZ, 047S476, 047S47Z, 047S4Z6, 047T046, 047T0D6, 047T0EZ, 047T0G6, 047T0Z6, 047T356, 047T3DZ, 047T456, 047T476, 047T4E6, 047U056, 047U0E6, 047U0FZ, 047U34Z, 047U376, 047U3D6, 047U3EZ, 047U47Z, 047U4FZ, 047V076, 047V07Z, 047V0F6, 047V0Z6, 047V34Z, 047V3E6, 047V3EZ, 047V44Z, 047V46Z, 047V476, 047V4FZ, 047W06Z, 047W0DZ, 047W0EZ, 047W0F6, 047W0GZ, 047W36Z, 047W3EZ, 047W4D6, 047W4GZ, 047Y046, 047Y06Z, 047Y356, 047Y37Z, 047Y3D6, 047Y3E6, 047Y476, 047Y47Z, 047Y4EZ, 04BM0ZZ, 04BP4ZZ, 04BQ0ZZ, 04BS4ZZ, 04BU4ZZ, 04BY4ZZ, 04CM0Z6, 04CN3Z6, 04CP4Z6, 04CQ0ZZ, 04CQ3Z6, 04CR3Z6, 04CR3ZZ, 04CS3Z6, 04CS3ZZ, 04CS4ZZ, 04CT0Z6, 04CT4ZZ, 04CU4ZZ, 04CV3ZZ, 04CV4Z6, 04CW3ZZ, 04CY4Z6, 04HC0DZ, 04HF0DZ, 04HH0DZ, 04HH4DZ, 04HK0DZ, 04HK3DZ, 04HK4DZ, 04HN3DZ, 04HQ4DZ, 04HT4DZ, 04HU3DZ, 04HY0DZ, 04ND3ZZ, 04NK0ZZ, 04NK4ZZ, 04NM0ZZ, 04NQ4ZZ, 04NR3ZZ, 04NR4ZZ, 04NS0ZZ, 04NU3ZZ, 04RK0JZ, 04RK0KZ, 04RL0KZ, 04RL4JZ, 04RL4KZ, 04RM47Z, 04RM4JZ, 04RN47Z, 04RN4KZ, 04RP47Z, 04RQ47Z, 04RQ4JZ, 04RR07Z, 04RR0JZ, 04RR4JZ, 04RS4KZ, 04RT4KZ, 04RU4KZ, 04RV0JZ, 04RW47Z, 04RW4JZ, 04RY0JZ, 04RY47Z, 04UD0KZ, 04UD3JZ, 04UE3JZ, 04UF0JZ, 04UF37Z, 04UH0KZ, 04UH37Z, 04UH47Z, 04UH4KZ, 04UJ4KZ, 04UK07Z, 04UL07Z, 04UL47Z, 04UL4JZ, 04UM37Z, 04UM3JZ, 04UN0JZ, 04UP3JZ, 04UP3KZ, 04UP47Z, 04UP4KZ, 04UQ47Z, 04UR07Z, 04UR3JZ, 04UR4JZ, 04UT0JZ, 04UT37Z,

© 2023 Lin KJ et al. *JAMA Network Open.*

	04UU4KZ, 04UV07Z, 04UW37Z, 04UW3JZ, 04UY4KZ, 04VC4CZ, 04VC4ZZ,<br>04VD3CZ, 04VD4CZ, 04VH0CZ, 04VH3CZ, 04VK0ZZ, 04VK4ZZ, 04VM4ZZ,<br>04VQ3CZ, 04VS0CZ, 04VT3CZ, 04VU4CZ, 04VV0ZZ, 04WY02Z, 04WY32Z,<br>04WY42Z, 04WY4DZ, 041009F, 041009J, 041009R, 04100JQ, 04100KD,<br>041049R, 04104A9, 04104AF, 04104AJ, 04104J8, 04104JG, 04104JH,<br>04104JR, 04104K6, 04104K7, 04104Z6, 041C0KH, 041C0ZK, 041C4AJ,<br>041C4JH, 041D09H, 041D0JK, 041D0ZJ, 041D0ZK, 041E4AK, 041E4KJ,<br>041F09K, 041F0JH, 041F0JJ, 041F0KK, 041F49J, 041H0KH, 041H4AJ,<br>041H4AK, 041H4KK, 041H4ZJ, 041J0AK, 041J0JH, 041J0JJ, 041J0KJ,<br>041J0ZK, 041J49K, 041J4AH, 041K09K, 041K09P, 041K09S, 041K0AJ,<br>041K0AN, 041K0JS, 041K0KL, 041K0KP, 041K0ZL, 041K0ZQ, 041K49J,<br>041K49P, 041K4KP, 041K4ZM, 041L09J, 041L09L, 041L09M, 041L09N,<br>041L09P, 041L0AL, 041L0AM, 041L0JH, 041L0ZK, 041L49H, 041L49S,<br>041L4AK, 041L4AS, 041L4JM, 041L4KH, 041L4ZL, 041L4ZP, 041M09L,<br>041M09P, 041M09Q, 041M0AL, 041M0AM, 041M0JM, 041M0ZM, 041M0ZQ,<br>041M4AM, 041M4AP, 041M4JP, 041M4JQ, 041M4ZP, 041N09L, 041N09M,<br>041N09P, 041N0AP, 041N0JP, 041N0JQ, 041N0KM, 041N4JL, 041N4KL,<br>041N4KM, 047004Z, 047035Z, 04703D6, 04703E6, 04703ZZ, 0470446,<br>0470466, 04704E6, 04704ZZ, 047C046, 047C04Z, 047C066, 047C076,<br>047C0ZZ, 047C356, 047C366, 047C3D6, 047C3F6, 047C3FZ, 047C3GZ,<br>047C3ZZ, 047C44Z, 047C47Z, 047D046, 047D0E6, 047D0FZ, 047D34Z,<br>047D3D6, 047D3GZ, 047D446, 047D466, 047D4D6, 047E0D6, 047E35Z,<br>047E3D6, 047E3Z6, 047E46Z, 047E476, 047E47Z, 047E4DZ, 047E4FZ,<br>047F0E6, 047F0F6, 047F34Z, 047F36Z, 047F3DZ, 047F3GZ, 047F3Z6,<br>047F3ZZ, 047F44Z, 047F456, 047F46Z, 047F4E6, 047H04Z, 047H07Z,<br>047H0DZ, 047H0E6, 047H3D6, 047H3Z6, 047H45Z, 047H4DZ, 047J046,<br>047J04Z, 047J076, 047J0D6, 047J0EZ, 047J0F6, 047J0GZ, 047J0ZZ,<br>047J35Z, 047J3EZ, 047J3F6, 047J44Z, 047J456, 047J476, 047J4FZ,<br>047J4GZ, 047J4ZZ, 047K076, 047K0D1, 047K36Z, 047K37Z, 047K3D1,<br>047K3G6, 047K456, 047K47Z, 047K4FZ, 047K4G6, 047K4GZ, 047K4Z1,<br>047K4Z6, 047L076, 047L0FZ, 047L0Z1, 047L37Z, 047L3FZ, 047L3GZ,<br>047L3ZZ, 047L4E6, 047L4EZ, 047L4Z1, 047M066, 047M076, 047M0Z1,<br>047M346, 047M356, 047M37Z, 047M3GZ, 047M44Z, 047M46Z, 047M476,<br>047M4DZ, 047M4E6, 047M4GZ, 047N041, 047N046, 047N04Z, 047N05Z,<br>047N066, 047N076, 047N0D1, 047N0EZ, 047N0F6, 047N0GZ, 047N0Z1,<br>047N341, 047N34Z, 047N366, 047N3D1, 047N3FZ, 047N3G6, 047N3Z1,<br>047N46Z, 047N47Z, 047N4D6, 047N4F6, 047N4G6, 047N4Z6, 047N4ZZ,<br>047P056, 047P05Z, 047P0D6, 047P0EZ, 047P0G6, 047P0ZZ, 047P35Z,<br>047P3EZ, 047P456, 047P45Z, 047P4D6, 047P4EZ, 047P4FZ, 047Q046,<br>047Q056, 047Q0FZ, 047Q0GZ, 047Q0ZZ, 047Q346, 047Q34Z, 047Q366,

© 2023 Lin KJ et al. *JAMA Network Open.*

	047Q466, 047Q4EZ, 047Q4F6, 047Q4FZ, 047R046, 047R056, 047R05Z, 047R06Z, 047R076, 047R0Z6, 047R0ZZ, 047R356, 047R36Z, 047R3ZZ, 047R476, 047R4E6, 047R4G6, 047R4GZ, 047S046, 047S06Z, 047S0D6, 047S0E6, 047S0FZ, 047S366, 047S3Z6, 047S3ZZ, 047S456, 047S466, 047S4DZ, 047S4E6, 047S4GZ, 047T04Z, 047T06Z, 047T076, 047T0DZ, 047T346, 047T366, 047T37Z, 047T3D6, 047T3EZ, 047T3FZ, 047T3G6, 047T466, 047T4D6, 047T4EZ, 047T4FZ, 047U066, 047U0D6, 047U346, 047U366, 047U3DZ, 047U3E6, 047U3F6, 047U44Z, 047U456, 047U4F6, 047V046, 047V04Z, 047V066, 047V0DZ, 047V356, 047V366, 047V3GZ, 047V456, 047V4EZ, 047V4F6, 047V4GZ, 047V4ZZ, 047W046, 047W066, 047W376, 047W37Z, 047W3D6, 047W3G6, 047W3Z6, 047W3ZZ, 047W44Z, 047W46Z, 047W47Z, 047W4G6, 047Y0DZ, 047Y346, 047Y3EZ, 047Y3F6, 047Y44Z, 047Y4D6, 047Y4F6, 04BK0ZZ, 04BV4ZZ, 04BW0ZZ, 04BY0ZZ, 04CK0ZZ, 04CK4ZZ, 04CL3ZZ, 04CM0ZZ, 04CM4ZZ, 04CP0ZZ, 04CP3ZZ, 04CT0ZZ, 04CU0ZZ, 04CU3Z6, 04CV4ZZ, 04CW0Z6, 04CW0ZZ, 04CW4Z6, 04HC3DZ, 04HF3DZ, 04HH3DZ, 04HJ4DZ, 04HN4DZ, 04HS3DZ, 04HT3DZ, 04HU4DZ, 04HV4DZ, 04HW0DZ, 04HY3DZ, 04HY42Z, 04ND0ZZ, 04NE3ZZ, 04NF4ZZ, 04NH3ZZ, 04NP0ZZ, 04NP3ZZ, 04NU0ZZ, 04NW4ZZ, 04QY3ZZ, 04RK07Z, 04RK4KZ, 04RL0JZ, 04RM0JZ, 04RN4JZ, 04RP4JZ, 04RP4KZ, 04RR0KZ, 04RR47Z, 04RR4KZ, 04RS07Z, 04RS0JZ, 04RS4JZ, 04RT07Z, 04RT4JZ, 04RU4JZ, 04RV07Z, 04RY07Z, 04RY4KZ, 04UC0KZ, 04UD3KZ, 04UD4JZ, 04UE3KZ, 04UE4JZ, 04UH3KZ, 04UJ3JZ, 04UJ47Z, 04UK37Z, 04UK3KZ, 04UK4KZ, 04UL3JZ, 04UL3KZ, 04UM07Z, 04UM4KZ, 04UN4JZ, 04UN4KZ, 04UP37Z, 04UP4JZ, 04UQ4JZ, 04UR0KZ, 04UR37Z, 04UR4KZ, 04US3JZ, 04UT3KZ, 04UT4KZ, 04UU07Z, 04UU3KZ, 04UV0JZ, 04UV3KZ, 04UV4KZ, 04UW0KZ, 04UY07Z, 04UY37Z, 04VC0CZ, 04VD0CZ, 04VE3CZ, 04VF0ZZ, 04VJ0ZZ, 04VK3CZ, 04VL3CZ, 04VM0CZ, 04VM3CZ, 04VN3CZ, 04VN4CZ, 04VP3CZ, 04VP4ZZ, 04VQ0ZZ, 04VQ4CZ, 04VR0CZ, 04VR4CZ, 04VR4ZZ, 04VS0ZZ, 04VS4CZ, 04VU0CZ, 04VU0ZZ, 04VU3CZ, 04VV4ZZ, 04VY3CZ, 04VY4CZ, 04WY00Z, 04WY3CZ, 04WY40Z, 04WY43Z, 04100A6, 04100AB, 04100AD, 04100AQ, 04100AR, 04100J7, 04100J8, 04100J9, 04100JF, 04100K6, 04100K7, 04100K8, 04100KQ, 04100Z9, 04100ZF, 04100ZK, 0410496, 0410499, 041049F, 041049Q, 04104AD, 04104AK, 04104J7, 04104JC, 04104JQ, 04104KH, 04104KK, 04104KR, 04104Z8, 04104ZJ, 04104ZR, 041C09J, 041C0AH, 041C4AK, 041C4JK, 041C4KH, 041C4KK, 041C4ZH, 041C4ZJ, 041D0AJ, 041D0JH, 041D0KK, 041D49J, 041D4AJ, 041D4KJ, 041E09H, 041E09J, 041E0JJ, 041E4AJ, 041E4KH, 041E4ZH, 041E4ZJ, 041F09J, 041F0KJ, 041F0ZH, 041F0ZK, 041F4KK, 041F4ZH, 041H0KJ, 041H0KK, 041H0ZJ, 041H49J, 041H4JH, 041H4KH, 041J0AH, 041J0JK, 041J49J, 041J4KH, 041J4KJ, 041K09Q, 041K0JM,

© 2023 Lin KJ et al. *JAMA Network Open.*

	041K0JP, 041K0JQ, 041K0KJ, 041K0KN, 041K49K, 041K49L, 041K49M, 041K4AM, 041K4JJ, 041K4JL, 041K4JN, 041K4ZJ, 041L09H, 041L09S, 041L0AJ, 041L0AS, 041L0JJ, 041L0JL, 041L0JM, 041L0JN, 041L0JP, 041L0KQ, 041L0KS, 041L0ZL, 041L0ZN, 041L49L, 041L49N, 041L4AJ, 041L4AN, 041L4AP, 041L4JJ, 041L4JK, 041L4JN, 041L4JQ, 041L4KL, 041L4KN, 041L4KP, 041L4KQ, 041L4ZM, 041L4ZN, 041M0AP, 041M0AS, 041M0JL, 041M0JP, 041M0KQ, 041M0ZL, 041M49M, 041M49Q, 041M49S, 041M4AQ, 041M4JL, 041M4KQ, 041M4ZL, 041N09Q, 041N0KP, 041N0KQ, 041N0KS, 041N4AL, 041N4AP, 041N4ZQ, 047006Z, 0470076, 04700D6, 04700DZ, 04700E6, 04700F6, 04700GZ, 04700ZZ, 0470366, 04703G6, 047046Z, 0470476, 04704D6, 04704DZ, 04704EZ, 04704G6, 047C06Z, 047C0GZ, 047C0Z6, 047C346, 047C34Z, 047C3DZ, 047C456, 047D05Z, 047D0D6, 047D346, 047D35Z, 047D3F6, 047D3FZ, 047D3G6, 047D3ZZ, 047D456, 047D47Z, 047D4DZ, 047D4EZ, 047D4Z6, 047E046, 047E05Z, 047E06Z, 047E076, 047E0E6, 047E0G6, 047E0GZ, 047E0ZZ, 047E376, 047E3G6, 047E3GZ, 047E3ZZ, 047E446, 047E456, 047E45Z, 047E4Z6, 047F056, 047F05Z, 047F0D6, 047F0EZ, 047F0FZ, 047F0GZ, 047F3D6, 047F3E6, 047F3EZ, 047F45Z, 047F47Z, 047F4D6, 047H076, 047H0EZ, 047H0F6, 047H35Z, 047H37Z, 047H3EZ, 047H446, 047H46Z, 047H47Z, 047H4D6, 047H4E6, 047H4FZ, 047J056, 047J05Z, 047J0G6, 047J376, 047J3DZ, 047J3E6, 047J3Z6, 047J47Z, 047J4D6, 047J4DZ, 047J4EZ, 047J4F6, 047J4Z6, 047K041, 047K05Z, 047K07Z, 047K366, 047K3EZ, 047K3ZZ, 047K441, 047K44Z, 047L05Z, 047L066, 047L06Z, 047L0DZ, 047L0EZ, 047L3D6, 047L44Z, 047L466, 047L46Z, 047L47Z, 047L4D6, 047L4Z6, 047M041, 047M04Z, 047M07Z, 047M0G6, 047M341, 047M36Z, 047M3D1, 047M3E6, 047M456, 047M45Z, 047M4ZZ, 047N0DZ, 047N0E6, 047N0FZ, 047N0G6, 047N356, 047N35Z, 047N376, 047N37Z, 047N3EZ, 047N44Z, 047N4FZ, 047P06Z, 047P0FZ, 047P0Z6, 047P346, 047P37Z, 047P3ZZ, 047P46Z, 047P4Z6, 047Q05Z, 047Q0D6, 047Q0DZ, 047Q3D6, 047Q3DZ, 047Q3E6, 047Q446, 047Q44Z, 047Q456, 047Q476, 047Q4DZ, 047Q4G6, 047R07Z, 047R0DZ, 047R0FZ, 047R35Z, 047R3F6, 047R3Z6, 047R44Z, 047R45Z, 047R4F6, 047R4Z6, 047S04Z, 047S056, 047S05Z, 047S066, 047S0GZ, 047S0Z6, 047S346, 047S446, 047S44Z, 047S46Z, 047S4D6, 047S4EZ, 047S4F6, 047S4G6, 047T0FZ, 047T0GZ, 047T34Z, 047T35Z, 047T3E6, 047T3GZ, 047T446, 047T4DZ, 047T4GZ, 047U04Z, 047U05Z, 047U076, 047U07Z, 047U0G6, 047U0GZ, 047U356, 047U36Z, 047U37Z, 047U3FZ, 047U476, 047U4GZ, 047V05Z, 047V06Z, 047V0E6, 047V36Z, 047V3D6, 047V3F6, 047V3FZ, 047V3ZZ, 047V446, 047V4D6, 047V4E6, 047V4Z6, 047W076, 047W0G6, 047W34Z, 047W3E6, 047W3FZ, 047W446, 047W456, 047W45Z, 047W4DZ, 047W4FZ, 047W4ZZ, 047Y066,

© 2023 Lin KJ et al. *JAMA Network Open.*

	047Y0D6, 047Y0EZ, 047Y35Z, 047Y3DZ, 047Y3ZZ, 047Y456, 047Y46Z, 047Y4Z6, 04BN0ZZ, 04BP0ZZ, 04BV0ZZ, 04BW4ZZ, 04CK4Z6, 04CL0ZZ, 04CM3ZZ, 04CN4ZZ, 04CQ4Z6, 04CR4ZZ, 04CT3Z6, 04CT3ZZ, 04CT4Z6, 04CU4Z6, 04CV3Z6, 04CW4ZZ, 04CY3Z6, 04CY3ZZ, 04CY4ZZ, 04HD3DZ, 04HD4DZ, 04HE3DZ, 04HJ0DZ, 04HL0DZ, 04HM3DZ, 04HP3DZ, 04HQ0DZ, 04HR3DZ, 04HR4DZ, 04HU0DZ, 04HV0DZ, 04HW3DZ, 04HY4DZ, 04ND4ZZ, 04NE4ZZ, 04NF3ZZ, 04NH0ZZ, 04NH4ZZ, 04NJ0ZZ, 04NK3ZZ, 04NL0ZZ, 04NL4ZZ, 04NM4ZZ, 04NS4ZZ, 04NT0ZZ, 04NT3ZZ, 04NU4ZZ, 04NV0ZZ, 04NV4ZZ, 04NY0ZZ, 04QY0ZZ, 04RS0KZ, 04RS47Z, 04RU0JZ, 04RU0KZ, 04RV47Z, 04RV4JZ, 04RV4KZ, 04RW0KZ, 04RW4KZ, 04RY4JZ, 04UC07Z, 04UC0JZ, 04UC37Z, 04UC3JZ, 04UC4JZ, 04UD07Z, 04UE37Z, 04UE4KZ, 04UF07Z, 04UF4JZ, 04UH0JZ, 04UH3JZ, 04UH4JZ, 04UJ0KZ, 04UJ37Z, 04UK0JZ, 04UK0KZ, 04UL37Z, 04UM0JZ, 04UM3KZ, 04UN37Z, 04UN3JZ, 04UP0JZ, 04US0JZ, 04US4JZ, 04UT07Z, 04UV0KZ, 04UV47Z, 04UW07Z, 04UW47Z, 04UW4JZ, 04UY0KZ, 04UY47Z, 04UY4JZ, 04VE4ZZ, 04VF0CZ, 04VF4ZZ, 04VH4ZZ, 04VJ3CZ, 04VJ4CZ, 04VL0CZ, 04VL4ZZ, 04VM4CZ, 04VN0CZ, 04VN0ZZ, 04VN4ZZ, 04VP4CZ, 04VR0ZZ, 04VR3CZ, 04VU4ZZ, 04VW4ZZ, 04WY03Z, 0410096, 041009D, 041009G, 041009Q, 04100A7, 04100A9, 04100AH, 04100AK, 04100JB, 04100JC, 04100JG, 04100JK, 04100JR, 04100K9, 04100KC, 04100ZG, 04100ZQ, 0410498, 041049G, 041049K, 04104A6, 04104AC, 04104AG, 04104AH, 04104AQ, 04104KD, 04104KF, 04104KG, 04104KJ, 04104KQ, 04104ZF, 04104ZG, 04104ZH, 041C0KJ, 041C4ZK, 041D09K, 041D4JH, 041D4JJ, 041D4KH, 041D4ZJ, 041D4ZK, 041E09K, 041E0AH, 041E0AJ, 041E0JH, 041E0KH, 041E0KK, 041E0ZK, 041E4AH, 041F09H, 041F0JK, 041F4AH, 041F4AK, 041F4KJ, 041H09J, 041H0JH, 041H0ZH, 041H49H, 041H4ZK, 041J09K, 041J0KH, 041J0KK, 041J0ZH, 041J4AK, 041J4JH, 041J4ZH, 041J4ZJ, 041J4ZK, 041K0AM, 041K0AS, 041K0KH, 041K0KS, 041K0ZJ, 041K0ZP, 041K49Q, 041K49S, 041K4AK, 041K4JH, 041K4JK, 041K4JM, 041K4JP, 041K4JS, 041K4KJ, 041K4KK, 041K4KL, 041K4KM, 041K4KS, 041K4ZK, 041K4ZN, 041K4ZP, 041K4ZS, 041L09Q, 041L0AP, 041L0AQ, 041L0JQ, 041L0KM, 041L0ZQ, 041L49P, 041L4JH, 041L4JL, 041L4KJ, 041L4ZH, 041L4ZJ, 041L4ZK, 041M09M, 041M0AQ, 041M0JQ, 041M0KL, 041M0KM, 041M0KP, 041M0KS, 041M0ZP, 041M49L, 041M49P, 041M4KS, 041N0AQ, 041N0KL, 041N0ZM, 041N0ZS, 041N4AQ, 041N4AS, 041N4JP, 041N4JQ, 041N4JS, 041N4KQ, 047005Z, 04700G6, 047037Z, 04703F6, 04703FZ, 047047Z, 047C0DZ, 047C0E6, 047C0F6, 047C0FZ, 047C0G6, 047C35Z, 047C36Z, 047C37Z, 047C3Z6, 047C466, 047C476, 047C4F6, 047C4G6, 047D056, 047D066, 047D06Z, 047D076, 047D07Z, 047D0DZ, 047D0EZ, 047D356, 047D366, 047D36Z, 047D37Z, 047D476, 047D4FZ, 047D4GZ, 047E04Z,

	047E056, 047E066, 047E0DZ, 047E3F6, 047E4D6, 047E4G6, 047F04Z, 047F076, 047F0ZZ, 047F346, 047F37Z, 047F3F6, 047F3G6, 047F466, 047F4DZ, 047F4FZ, 047F4GZ, 047H0D6, 047H0FZ, 047H0G6, 047H346, 047H34Z, 047H366, 047H3F6, 047H3ZZ, 047H44Z, 047H456, 047H476, 047H4F6, 047H4GZ, 047J0E6, 047J46Z, 047J4E6, 047J4G6, 047K046, 047K0D6, 047K0EZ, 047K0FZ, 047K34Z, 047K356, 047K3F6, 047K466, 047K4D6, 047L07Z, 047L0F6, 047L0G6, 047L0ZZ, 047L35Z, 047L366, 047L3D1, 047L3DZ, 047L3EZ, 047L3G6, 047L456, 047L4F6, 047L4G6, 047M056, 047M06Z, 047M0DZ, 047M0F6, 047M0ZZ, 047M3DZ, 047M3F6, 047M446, 047M47Z, 047M4D1, 047M4D6, 047M4FZ, 047N07Z, 047N0D6, 047N3D6, 047N3DZ, 047N456, 047N466, 047N4EZ, 047N4GZ, 047P046, 047P0E6, 047P0GZ, 047P34Z, 047P376, 047P3D6, 047P3F6, 047P446, 047P466, 047P47Z, 047P4E6, 047P4F6, 047Q04Z, 047Q07Z, 047Q0E6, 047Q0F6, 047Q376, 047Q3F6, 047Q3GZ, 047Q3Z6, 047Q3ZZ, 047Q45Z, 047Q46Z, 047Q4E6, 047R04Z, 047R0D6, 047R366, 047R37Z, 047R3DZ, 047R3EZ, 047R466, 047R46Z, 047R47Z, 047R4DZ, 047S0F6, 047S36Z, 047S3DZ, 047S3EZ, 047S3GZ, 047S4FZ, 047S4ZZ, 047T05Z, 047T0E6, 047T376, 047T3F6, 047T44Z, 047T45Z, 047T46Z, 047T47Z, 047T4F6, 047T4G6, 047T4ZZ, 047U06Z, 047U0DZ, 047U0EZ, 047U0F6, 047U0Z6, 047U446, 047U46Z, 047U4EZ, 047U4ZZ, 047V056, 047V0D6, 047V0EZ, 047V0FZ, 047V0ZZ, 047V346, 047V35Z, 047V37Z, 047V466, 047V47Z, 047V4DZ, 047W04Z, 047W056, 047W0D6, 047W0FZ, 047W0Z6, 047W0ZZ, 047W356, 047W35Z, 047W366, 047W3F6, 047W3GZ, 047W466, 047W4Z6, 047Y0E6, 047Y0F6, 047Y0FZ, 047Y0ZZ, 047Y36Z, 047Y376, 047Y3FZ, 047Y3GZ, 047Y446, 047Y45Z, 047Y466, 047Y4DZ, 047Y4E6, 047Y4GZ, 047Y4ZZ, 04BL4ZZ, 04BN4ZZ, 04BR0ZZ, 04BS0ZZ, 04BT4ZZ, 04CL0Z6, 04CL4Z6, 04CL4ZZ, 04CM4Z6, 04CN0Z6, 04CP3Z6, 04CP4ZZ, 04CQ0Z6, 04CQ4ZZ, 04CR0Z6, 04CS0ZZ, 04CV0ZZ, 04CY0ZZ, 04HC4DZ, 04HE4DZ, 04HF4DZ, 04HL3DZ, 04HN0DZ, 04HP0DZ, 04HQ3DZ, 04HS0DZ, 04HS4DZ, 04HT0DZ, 04HV3DZ, 04HW4DZ, 04HY02Z, 04NC0ZZ, 04NC3ZZ, 04NC4ZZ, 04NN0ZZ, 04NQ0ZZ, 04NR0ZZ, 04NS3ZZ, 04NT4ZZ, 04NV3ZZ, 04NW3ZZ, 04RK47Z, 04RK4JZ, 04RM07Z, 04RM0KZ, 04RM4KZ, 04RP0KZ, 04RQ0JZ, 04RQ0KZ, 04RT0JZ, 04RU07Z, 04RV0KZ, 04UC47Z, 04UC4KZ, 04UD0JZ, 04UD37Z, 04UD47Z, 04UE47Z, 04UF4KZ, 04UH07Z, 04UJ07Z, 04UJ0JZ, 04UJ3KZ, 04UK3JZ, 04UK4JZ, 04UL4KZ, 04UM4JZ, 04UN07Z, 04UN0KZ, 04UN47Z, 04UP07Z, 04UQ0JZ, 04UQ0KZ, 04UQ37Z, 04US07Z, 04US37Z, 04US4KZ, 04UT3JZ, 04UT4JZ, 04UU4JZ, 04UV37Z, 04UV3JZ, 04UW4KZ, 04UY0JZ, 04UY3JZ, 04UY3KZ, 04VC0ZZ, 04VC3CZ, 04VE0CZ, 04VF3CZ, 04VF4CZ, 04VH0ZZ, 04VH4CZ, 04VK0CZ, 04VL0ZZ, 04VL4CZ, 04VM0ZZ, 04VP0ZZ, 04VQ0CZ, 04VS4ZZ, 04VT0CZ, 04VT4ZZ, 04VV0CZ, 04VW0CZ,

© 2023 Lin KJ et al. *JAMA Network Open.*

	04VW3CZ, 04VW4CZ, 04VY0ZZ, 04VY4ZZ, 04WY0CZ, 04WY3DZ, 04WY4CZ, 0410097, 0410099, 041009C, 041009H, 04100AC, 04100AF, 04100AJ, 04100JD, 04100KF, 04100KG, 04100KH, 04100KK, 04100KR, 04100Z6, 04100ZB, 04100ZH, 04100ZR, 041049B, 04104AB, 04104J9, 04104JD, 04104JF, 04104JJ, 04104K9, 04104KB, 04104KC, 04104Z7, 04104ZB, 04104ZC, 04104ZQ, 041C09H, 041C09K, 041C0AJ, 041C0AK, 041C0JJ, 041C0JK, 041C0ZH, 041C0ZJ, 041C49H, 041C49J, 041C4JJ, 041D0AH, 041D0KH, 041D0ZH, 041D49K, 041D4AH, 041D4JK, 041D4KK, 041D4ZH, 041E0AK, 041E0JK, 041E0ZJ, 041E49H, 041E49J, 041E4KK, 041F0AJ, 041F0KH, 041F0ZJ, 041F49K, 041F4JJ, 041F4JK, 041H09K, 041H0AH, 041H0AK, 041H0JJ, 041H49K, 041H4KJ, 041J49H, 041J4AJ, 041J4JJ, 041J4JK, 041K09L, 041K09N, 041K0AL, 041K0AP, 041K0JH, 041K0JL, 041K0ZK, 041K0ZM, 041K0ZN, 041K0ZS, 041K4AJ, 041K4AL, 041K4JQ, 041K4KN, 041K4KQ, 041K4ZQ, 041L09K, 041L0AK, 041L0AN, 041L0JK, 041L0KJ, 041L0KL, 041L0KN, 041L0ZH, 041L0ZJ, 041L0ZP, 041L0ZS, 041L49J, 041L49M, 041L49Q, 041L4AH, 041L4JS, 041L4KM, 041L4ZS, 041M09S, 041M0JS, 041M4AL, 041M4JM, 041M4JS, 041M4KL, 041M4KM, 041M4KP, 041M4ZM, 041N0AL, 041N0JL, 041N0JM, 041N0JS, 041N0ZL, 041N0ZP, 041N0ZQ, 041N49L, 041N49M, 041N49Q, 041N49S, 041N4KS, 041N4ZL, 041N4ZP, 041N4ZS, 04700EZ, 04700FZ, 04700Z6, 047034Z, 0470356, 047036Z, 0470376, 04703DZ, 04703EZ, 04703Z6, 047045Z, 04704Z6, 047C056, 047C0D6, 047C0EZ, 047C446, 047C45Z, 047C46Z, 047C4FZ, 047D04Z, 047D0GZ, 047D0Z6, 047D0ZZ, 047D376, 047D3E6, 047D3Z6, 047D44Z, 047D45Z, 047D46Z, 047D4F6, 047D4ZZ, 047E34Z, 047E356, 047E366, 047E36Z, 047E3DZ, 047E4E6, 047F046, 047F06Z, 047F356, 047F366, 047F3FZ, 047F4EZ, 047F4F6, 047F4G6, 047H056, 047H066, 047H0GZ, 047H356, 047H36Z, 047H376, 047H3FZ, 047H3G6, 047H3GZ, 047H4EZ, 047H4ZZ, 047J07Z, 047J0Z6, 047J346, 047J356, 047J366, 047J36Z, 047J37Z, 047J3D6, 047J446, 047J45Z, 047K04Z, 047K066, 047K0DZ, 047K0F6, 047K0GZ, 047K346, 047K35Z, 047K3DZ, 047K3GZ, 047K3Z1, 047K446, 047K4ZZ, 047L046, 047L04Z, 047L056, 047L0D6, 047L346, 047L356, 047L376, 047L3E6, 047L3F6, 047L3Z6, 047L441, 047L45Z, 047L476, 047L4DZ, 047L4FZ, 047M046, 047M0D6, 047M0E6, 047M0FZ, 047M0GZ, 047M0Z6, 047M34Z, 047M366, 047M376, 047M3EZ, 047M3FZ, 047M3G6, 047M441, 047M4F6, 047N06Z, 047N0Z6, 047N0ZZ, 047N3F6, 047N3ZZ, 047N441, 047N446, 047N476, 047N4D1, 047P0DZ, 047P0F6, 047P356, 047P366, 047P3FZ, 047P3Z6, 047P44Z, 047P4GZ, 047Q076, 047Q0EZ, 047Q0G6, 047Q0Z6, 047Q356, 047Q35Z, 047Q36Z, 047Q37Z, 047Q3EZ, 047Q3G6, 047Q4GZ, 047Q4ZZ, 047R0E6, 047R0G6, 047R346, 047R34Z, 047R3E6, 047R3FZ, 047R4D6, 047R4EZ,

© 2023 Lin KJ et al. *JAMA Network Open.*

	047R4FZ, 047S0DZ, 047S0ZZ, 047S376, 047S3E6, 047S3F6, 047S3G6, 047S45Z, 047T056, 047T066, 047T07Z, 047T0F6, 047T0ZZ, 047T36Z, 047T3Z6, 047T3ZZ, 047T4Z6, 047U046, 047U0ZZ, 047U35Z, 047U3G6, 047U3GZ, 047U3Z6, 047U3ZZ, 047U45Z, 047U466, 047U4D6, 047U4DZ, 047U4E6, 047U4G6, 047U4Z6, 047V0G6, 047V0GZ, 047V376, 047V3DZ, 047V3G6, 047V3Z6, 047V45Z, 047V4G6, 047W05Z, 047W07Z, 047W0E6, 047W346, 047W3DZ, 047W476, 047W4E6, 047W4EZ, 047W4F6, 047Y04Z, 047Y056, 047Y05Z, 047Y076, 047Y07Z, 047Y0G6, 047Y0GZ, 047Y0Z6, 047Y34Z, 047Y366, 047Y3G6, 047Y3Z6, 047Y4FZ, 047Y4G6, 04BK4ZZ, 04BL0ZZ, 04BM4ZZ, 04BQ4ZZ, 04BR4ZZ, 04BT0ZZ, 04BU0ZZ, 04CK0Z6, 04CK3Z6, 04CK3ZZ, 04CL3Z6, 04CM3Z6, 04CN0ZZ, 04CN3ZZ, 04CN4Z6, 04CP0Z6, 04CQ3ZZ, 04CR0ZZ, 04CR4Z6, 04CS0Z6, 04CS4Z6, 04CU0Z6, 04CU3ZZ, 04CV0Z6, 04CW3Z6, 04CY0Z6, 04HD0DZ, 04HE0DZ, 04HJ3DZ, 04HL4DZ, 04HM0DZ, 04HM4DZ, 04HP4DZ, 04HR0DZ, 04NE0ZZ, 04NF0ZZ, 04NJ3ZZ, 04NJ4ZZ, 04NL3ZZ, 04NM3ZZ, 04NN3ZZ, 04NN4ZZ, 04NP4ZZ, 04NQ3ZZ, 04NW0ZZ, 04NY3ZZ, 04NY4ZZ, 04QY4ZZ, 04RL07Z, 04RL47Z, 04RN07Z, 04RN0JZ, 04RN0KZ, 04RP07Z, 04RP0JZ, 04RQ07Z, 04RQ4KZ, 04RT0KZ, 04RT47Z, 04RU47Z, 04RW07Z, 04RW0JZ, 04RY0KZ, 04UC3KZ, 04UD4KZ, 04UE07Z, 04UE0JZ, 04UE0KZ, 04UF0KZ, 04UF3JZ, 04UF3KZ, 04UF47Z, 04UJ4JZ, 04UK47Z, 04UL0JZ, 04UL0KZ, 04UM0KZ, 04UM47Z, 04UN3KZ, 04UP0KZ, 04UQ07Z, 04UQ3JZ, 04UQ3KZ, 04UQ4KZ, 04UR0JZ, 04UR3KZ, 04UR47Z, 04US0KZ, 04US3KZ, 04US47Z, 04UT0KZ, 04UT47Z, 04UU0JZ, 04UU0KZ, 04UU37Z, 04UU3JZ, 04UU47Z, 04UV4JZ, 04UW0JZ, 04UW3KZ, 04VD0ZZ, 04VD4ZZ, 04VE0ZZ, 04VE4CZ, 04VJ0CZ, 04VJ4ZZ, 04VK4CZ, 04VP0CZ, 04VQ4ZZ, 04VS3CZ, 04VT0ZZ, 04VT4CZ, 04VV3CZ, 04VV4CZ, 04VW0ZZ, 04VY0CZ, 04WY0DZ, 04WY30Z, 04WY33Z<br>ICD-9 Diagnosis Code (any position) is any of: 440.20, 440.23, 440.31, 440.32, 440.22, 440.30, 443.9, 440.29, 440.4, 440.24, 440.21<br>ICD-10 Diagnosis Code is any of: I70.219, I70.231, I70.232, I70.233, I70.242, I70.244, I70.25, I70.268, I70.269, I70.291, I70.299, I70.303, I70.311, I70.313, I70.343, I70.345, I70.398, I70.401, I70.444, I70.492, I70.499, I70.502, I70.508, I70.513, I70.522, I70.531, I70.532, I70.535, I70.542, I70.545, I70.55, I70.568, I70.592, I70.608, I70.621, I70.628, I70.631, I70.641, I70.642, I70.649, I70.663, I70.668, I70.735, I70.761, I70.762, I70.793, I73.9, I70.211, I70.212, I70.222, I70.223, I70.234, I70.262, I70.292, I70.298, I70.334, I70.338, I70.342, I70.362, I70.368, I70.403, I70.418, I70.432, I70.442, I70.445, I70.462, I70.469, I70.503, I70.511, I70.529, I70.538, I70.544, I70.548, I70.549, I70.591, I70.599, I70.633, I70.634, I70.639, I70.661, I70.691, I70.692, I70.699, I70.711, I70.712, I70.718, I70.719, I70.742, I70.743, I70.744, I70.748, I70.791, I70.203, I70.208, I70.213, I70.229, I70.239, I70.245, I70.261, I70.301, I70.302, I70.318, I70.321, I70.323,

© 2023 Lin KJ et al. *JAMA Network Open.*

	I70.328, I70.329, I70.332, I70.333, I70.341, I70.344, I70.348, I70.35, I70.361, I70.391, I70.393, I70.411, I70.412, I70.413, I70.419, I70.422, I70.433, I70.443, I70.461, I70.512, I70.518, I70.519, I70.521, I70.523, I70.528, I70.543, I70.561, I70.563, I70.598, I70.602, I70.611, I70.618, I70.619, I70.635, I70.644, I70.645, I70.648, I70.662, I70.669, I70.702, I70.708, I70.713, I70.728, I70.731, I70.733, I70.738, I70.739, I70.745, I70.749, I70.799, I70.201, I70.209, I70.218, I70.221, I70.235, I70.241, I70.243, I70.293, I70.308, I70.309, I70.312, I70.319, I70.322, I70.331, I70.339, I70.349, I70.363, I70.369, I70.408, I70.409, I70.421, I70.428, I70.434, I70.438, I70.441, I70.448, I70.449, I70.45, I70.491, I70.493, I70.509, I70.533, I70.534, I70.562, I70.593, I70.603, I70.612, I70.623, I70.643, I70.65, I70.693, I70.701, I70.721, I70.729, I70.741, I70.75, I70.769, I70.792, I70.92, I70.202, I70.228, I70.238, I70.248, I70.249, I70.263, I70.335, I70.392, I70.399, I70.402, I70.423, I70.429, I70.431, I70.435, I70.439, I70.463, I70.468, I70.498, I70.501, I70.539, I70.541, I70.569, I70.601, I70.609, I70.613, I70.622, I70.629, I70.632, I70.638, I70.698, I70.703, I70.709, I70.722, I70.723, I70.732, I70.734, I70.763, I70.768, I70.798
Smoking	ICD-9 Diagnosis Code (any position) is any of: 989.84, 305.1, 649.0, 649.00, 649.04, 649.01, 649.02, 649.03, V15.82<br>ICD-10 Diagnosis Code (any position) is any of: F17.200, O99.332, T65.221A, T65.292A, F17.221, F17.291, F17.201, O99.331, O99.333, O99.334, T65.212A, T65.213A, T65.214A, T65.222A, T65.223A, T65.293A, T65.294A, F17.210, F17.211, F17.220, F17.290, O99.335, T65.211A, T65.224A, T65.291A, Z87.891, O99.330<br>HCPCS Procedure Code is any of: 1034F, 4001F, 99406, G9016, G9458, S4995, S9453, 4004F, 99407, G9276, G0437, G0436, S9075<br>NDC Generic Name is any of: NICOTINE BITARTRATE, VARENICLINE TARTRATE, NICOTINE, NICOTINE POLACRILEX
Stroke	ICD-9 Diagnosis Code (any position) is any of: 433.00, 433.20, 433.30, 433.90, 434.1, 434.9, 434.91, 438.19, 438.30, 438.40, 438.41, 438.51, 438.7, 438.85, 433.01, 433.1, 433.11, 433.3, 434.00, 434.10, 438.11, 438.21, 438.50, 438.6, 438.89, 433.2, 433.8, 433.80, 433.9, 436, 437.1, 438.1, 438.12, 438.13, 438.22, 438.31, 438.32, 438.42, 438.53, 438.82, 438.83, 433.10, 433.31, 433.81, 434.90, 438.10, 438.2, 438.20, 438.5, 438.52, 438.8, 438.81, 438.84, 431, 433.0, 433.21, 433.91, 434.0, 434.01, 434.11, 438.0, 438.14, 438.3, 438.4, 438.9<br>ICD-10 Diagnosis Code (any position) is any of: G45.9, G46.6, I63.013, I63.031, I63.033, I63.232, I63.323, I63.331, I63.342, I63.349, I63.50, I63.513, I63.521, I63.522, I63.523, I63.529, I63.541, I63.8, I65.21, I65.23, I66.02, I67.1, I67.7, G46.2, I63.00, I63.012, I63.12, I63.211, I63.213, I63.219, I63.239, I63.29, I63.313, I63.319, I63.329, I63.341, I63.39, I63.411, I63.413, I63.419, I63.432,

© 2023 Lin KJ et al. *JAMA Network Open.*

	I63.433, I63.449, I63.511, I63.532, I63.543, I63.549, I63.59, I65.9, I66.03, I66.22, I66.29, I66.9, I67.4, I67.89, I67.9, I68.8, G45.4, G45.8, G46.7, I63.019, I63.032, I63.112, I63.113, I63.131, I63.20, I63.233, I63.322, I63.333, I63.339, I63.40, I63.421, I63.422, I63.429, I63.431, I63.442, I63.443, I63.533, I63.539, I63.6, I63.9, I65.01, I65.02, I65.03, I65.1, I65.29, I65.8, I66.09, I66.12, I66.23, I66.3, I66.8, I67.2, I67.5, I67.6, I67.848, G45.0, G45.1, G45.2, G46.4, G46.5, G46.8, I63.02, I63.111, I63.133, I63.139, I63.19, I63.212, I63.231, I63.30, I63.312, I63.332, I63.343, I63.49, I63.512, I63.531, I63.542, I66.19, I66.21, I67.81, I68.2, G46.0, G46.1, G46.3, I63.011, I63.039, I63.09, I63.10, I63.119, I63.132, I63.22, I63.311, I63.321, I63.412, I63.423, I63.439, I63.441, I63.519, I65.09, I65.22, I66.01, I66.11, I66.13, I67.82, I67.841, I68.0
Syncope	ICD-9 Diagnosis Code (any position) is any of: 780.2, 992.1<br>ICD-10 Diagnosis Code (any position) is any of: R55
ACE inhibitors	NDC Generic Name is any of: CAPTOPRIL, LISINOPRIL, RAMIPRIL, TRANDOLAPRIL
ARBs	NDC Generic Name is any of: IRBESARTAN, TELMISARTAN, VALSARTAN
Antiarrhythmic agents	NDC Generic Name is any of: AMIODARONE HCL, DOFETILIDE, DRONEDARONE HCL, FLECAINIDE ACETATE, IBUTILIDE FUMARATE, MEXILETINE HCL, MORICIZINE HCL, PROCAINAMIDE HCL, PROPAFENONE HCL, QUINIDINE HCL, TOCAINIDE HCL
Injectable Anticoagulants	NDC Generic Name is any of: DALTEPARIN SODIUM,PORCINE, ENOXAPARIN SODIUM, FONDAPARINUX SODIUM
Antiplatelet agents	NDC Generic Name is any of: ASPIRIN, TICAGRELOR, CLOPIDOGREL BISULFATE, ASPIRIN (CALCIUM CARB & MAGNESIUM BUFFERS)/PRAVASTATIN, ASPIRIN/ACETAMINOPHEN, ASPIRIN/ACETAMINOPHEN/CAFFEINE, ASPIRIN/ACETAMINOPHEN/CAFFEINE/CALCIUM, ASPIRIN/ACETAMINOPHEN/CAFFEINE/POTASSIUM, ASPIRIN/ACETAMINOPHEN/CALCIUM CARBONATE, ASPIRIN/ACETAMINOPHEN/MAGNESIUM/ALUMINUM HYDROXIDE/CAFFEINE, ASPIRIN/CAFFEINE, ASPIRIN/CALCIUM CARBONATE, ASPIRIN/CALCIUM CARBONATE/MAGNESIUM, ASPIRIN/CALCIUM CARBONATE/MAGNESIUM/ALUMINUM HYDROXIDE, ASPIRIN/CODEINE PHOSPHATE, ASPIRIN/DIPHENHYDRAMINE CITRATE, ASPIRIN/DIPHENHYDRAMINE HCL, ASPIRIN/DIPHENHYDRAMINE/SODIUM BICARBONATE/CITRIC ACID, ASPIRIN/DIPYRIDAMOLE, ASPIRIN/MAGNESIUM CARBONATE/DIHYDROXYALUMINUM AMINOACETATE, ASPIRIN/MAGNESIUM HYDROXIDE/ALUMINUM HYDROXIDE, ASPIRIN/MAGNESIUM HYDROXIDE/ALUMINUM HYDROXIDE/CAFFEINE, ASPIRIN/MEPROBAMATE,

© 2023 Lin KJ et al. *JAMA Network Open.*

	ASPIRIN/SALICYLAMIDE/ACETAMINOPHEN/CAFFEINE, ASPIRIN/SALICYLAMIDE/CAFFEINE, ASPIRIN/SODIUM BICARBONATE/CITRIC ACID, BUTALBITAL/ASPIRIN/CAFFEINE, CARISOPRODOL/ASPIRIN, CHLORPHENIRAMINE MAL/PHENYLEPHRINE/D-METHORPHAN HB/ASPIRIN, CHLORPHENIRAMINE MALEATE/PHENYLEPHRINE BITARTRATE/ASPIRIN, CINNAMEDRINE HCL/ASPIRIN/CAFFEINE, CODEINE PHOSPHATE/BUTALBITAL/ASPIRIN/CAFFEINE, CODEINE PHOSPHATE/CARISOPRODOL/ASPIRIN, CODEINE/ASPIRIN/SALICYLAMIDE/ACETAMINOPHEN/CAFFEINE, DIHYDROCODEINE BITARTRATE/ASPIRIN/CAFFEINE, DIHYDROCODEINE/ASPIRIN/CAFFEINE, EPHEDRINE/ASPIRIN/ACETANILIDE/CAFFEINE, HYDROCODONE BITARTRATE/ASPIRIN, METHOCARBAMOL/ASPIRIN, ORPHENADRINE CITRATE/ASPIRIN/CAFFEINE, OXYCODONE HCL/ASPIRIN, OXYCODONE HCL/OXYCODONE TEREPHTHALATE/ASPIRIN, OXYCODONE/ASPIRIN, PENTAZOCINE HCL/ASPIRIN, PHENYLEPHRINE HCL/ASPIRIN, PHENYLPROPANOLAMINE BITARTRATE/ASPIRIN, PHENYLPROPANOLAMINE BITARTRATE/ASPIRIN/CHLORPHENIRAMINE, PHENYLPROPANOLAMINE HCL/ASPIRIN, PHENYLPROPANOLAMINE HCL/ASPIRIN/CHLORPHENIRAMINE, PHENYLPROPANOLAMINE HCL/ASPIRIN/CHLORPHENIRAMINE/CAFFEINE, PHENYLPROPANOLAMINE HCL/ASPIRIN/DIPHENHYDRAMINE, PROPOXYPHENE HCL/ASPIRIN/CAFFEINE, PSEUDOEPHEDRINE HCL/ASPIRIN/CHLORPHENIRAMINE, PRASUGREL HCL
Beta-Blockers	NDC Generic Name is any of: ATENOLOL, CARVEDILOL, METOPROLOL SUCCINATE, METOPROLOL TARTRATE, NADOLOL, PINDOLOL
Calcium Channel Blockers	NDC Generic Name is any of: ISRADIPINE, NIFEDIPINE, NIMODIPINE, NISOLDIPINE<br>NDC Brand Name is any of: ADALAT, CALAN, CARDENE, CARDIZEM, CLEVIPREX, COVERA-HS, DILT-CD, DYNACIRC, ISRADIPINE, NIFEDIPINE, NIMODIPINE, NISOLDIPINE, NORVASC, NYMALIZE, PLENDIL, PROCARDIA, SULAR, TIAZAC, VASCOR, VERELAN
Diuretics	NDC Brand Name is any of: BUMETANIDE, BUMEX, DELONE, DEMADEX, DETUE, DIAQUA-2, EDECRIN, ETHACRYNIC ACID, FUMIDE, FUROBEN 40, FUROMIDE, FUROSEMIDE, FUROSEMIDE-0.9 % NACL, FUROSEMIDE-0.9% NACL, LASAJECT-10, LASAJECT-2, LASIMIDE, LASIX, LO-AQUA, ROSE-40, TORSEMIDE, AMILORIDE HCL, AMILORIDE HCL W/HCTZ, AMILORIDE HCL-HCTZ, AMILORIDE HYDROCHLORIDE, AMILORIDE-HYDROCHLOROTHIAZIDE, EPLERENONE, SPIRONOLACTONE,

© 2023 Lin KJ et al. *JAMA Network Open.*

	SPIRONOLACTONE W/HCTZ, SPIRONOLACTONE-HCTZ, TRIAMTERENE, TRIAMTERENE W/HCTZ, TRIAMTERENE-HCTZ, TRIAMTERENE-HYDROCHLOROTHIAZID, ATENOLOL W/CHLORTHALIDONE, ATENOLOL-CHLORTHALIDONE, BENAZEPRIL-HYDROCHLOROTHIAZIDE, BENDROFLUMETHIAZIDE, BISOPROLOL-HYDROCHLOROTHIAZIDE, CAPTOPRIL-HYDROCHLOROTHIAZIDE, CAPTOPRIL/HYDROCHLOROTHIAZIDE, CHLOROTHIAZIDE, CHLOROTHIAZIDE SODIUM, CHLOROTHIAZIDE W/RESERPINE, CHLOROTHIAZIDE/RESERPINE, CHLORTHALIDONE, CLONIDINE HCL W/CHLORTHALIDONE, CLONIDINE W/CHLORTHALIDONE, ENALAPRIL-HYDROCHLOROTHIAZIDE, FOSINOPRIL-HYDROCHLOROTHIAZIDE, HYDROCHLOROTHIAZIDE, HYDROCHLOROTHIAZIDE PLUS, HYDROCHLOROTHIAZIDE/RESERPINE, HYDROFLUMETHIAZIDE, HYDROFLUMETHIAZIDE W/RESERPINE, HYDROFLUMETHIAZIDE/RESERPINE, INDAPAMIDE, IRBESARTAN-HYDROCHLOROTHIAZIDE, LISINOPRIL-HYDROCHLOROTHIAZIDE, LOSARTAN-HYDROCHLOROTHIAZIDE, METHYCLOTHIAZIDE, METHYCLOTHIAZIDE W/DESERPIDINE, METHYLDOPA W/CHLOROTHIAZIDE, METHYLDOPA-HYDROCHLOROTHIAZIDE, METHYLDOPA/HYDROCHLOROTHIAZIDE, METOLAZONE, METOPROLOL-HYDROCHLOROTHIAZIDE, MOEXIPRIL-HYDROCHLOROTHIAZIDE, NADOLOL-BENDROFLUMETHIAZIDE, QUINAPRIL-HYDROCHLOROTHIAZIDE, RAUWOLFIA/BENDROFLUMETHIAZIDE, RESERPINE/HYDROCHLOROTHIAZIDE, SK-CHLOROTHIAZIDE, SK-HYDROCHLOROTHIAZIDE, VALSARTAN-HYDROCHLOROTHIAZIDE
Fibrates	NDC Generic Name is any of: FENOFIBRATE, FENOFIBRATE NANOCRYSTALLIZED, FENOFIBRATE,MICRONIZED, FENOFIBRIC ACID, FENOFIBRIC ACID (CHOLINE), GEMFIBROZIL
Statins	NDC Generic Name is any of: LOVASTATIN, SIMVASTATIN, AMLODIPINE BESYLATE/ATORVASTATIN CALCIUM, ASPIRIN (CALCIUM CARB & MAGNESIUM BUFFERS)/PRAVASTATIN, ATORVASTATIN CALCIUM, CERIVASTATIN SODIUM, EZETIMIBE/ATORVASTATIN CALCIUM, EZETIMIBE/SIMVASTATIN, FLUVASTATIN SODIUM, IMIPENEM/CILASTATIN SODIUM, NIACIN/LOVASTATIN, NIACIN/SIMVASTATIN, NYSTATIN, NYSTATIN/EMOLLIENT COMBINATION NO.54, NYSTATIN/TRIAMCINOLONE ACETONIDE, PENTOSTATIN, PITAVASTATIN CALCIUM, PRAVASTATIN SODIUM, ROSUVASTATIN CALCIUM, SITAGLIPTIN PHOSPHATE/SIMVASTATIN

© 2023 Lin KJ et al. *JAMA Network Open.*

Nitrates	NDC Generic Name is any of: ISOSORBIDE DINITRATE, ISOSORBIDE DINITRATE/HYDRALAZINE HCL, ISOSORBIDE MONONITRATE, NITROGLYCERIN, NITROGLYCERIN/DEXTROSE 5 % IN WATER
Insulin	NDC Generic Name is any of: DILUENT,INSULIN ASPART COMBINATION #1, INSULIN ASPART, INSULIN ASPART PROTAMINE HUMAN/INSULIN ASPART, INSULIN DETEMIR, INSULIN GLARGINE,HUMAN RECOMBINANT ANALOG, INSULIN GLULISINE, INSULIN LISPRO, INSULIN LISPRO PROTAMINE & INSULIN LISPRO
Metformin	NDC Generic Name is any of: METFORMIN HCL, METFORMIN/AMINO ACIDS COMB. #7/HERBAL COMB.#125/CHOLINE, METFORMIN/CAFFEINE/AMINO ACIDS#7/HERBAL COMB#125/CHOLINE BIT, ALOGLIPTIN BENZOATE/METFORMIN HCL, CANAGLIFLOZIN/METFORMIN HCL, DAPAGLIFLOZIN PROPANEDIOL/METFORMIN HCL, GLIPIZIDE/METFORMIN HCL, GLYBURIDE/METFORMIN HCL, LINAGLIPTIN/METFORMIN HCL, PIOGLITAZONE HCL/METFORMIN HCL, REPAGLINIDE/METFORMIN HCL, ROSIGLITAZONE MALEATE/METFORMIN HCL, SAXAGLIPTIN HCL/METFORMIN HCL, SITAGLIPTIN PHOSPHATE/METFORMIN HCL
Sulfonylurea	NDC Generic Name is any of: CHLORPROPAMIDE, GLIMEPIRIDE, GLIPIZIDE, GLIPIZIDE/METFORMIN HCL, GLYBURIDE, GLYBURIDE,MICRONIZED, GLYBURIDE/METFORMIN HCL, PIOGLITAZONE HCL/GLIMEPIRIDE, ROSIGLITAZONE MALEATE/GLIMEPIRIDE, TOLAZAMIDE, TOLBUTAMIDE
Other oral hypoglycemic agents	NDC Generic Name is any of: ACARBOSE, ALOGLIPTIN BENZOATE, ALOGLIPTIN BENZOATE/PIOGLITAZONE HCL, EMPAGLIFLOZIN/LINAGLIPTIN, LINAGLIPTIN, MIGLITOL, NATEGLINIDE, PIOGLITAZONE HCL, REPAGLINIDE, ROSIGLITAZONE MALEATE, ROSIGLITAZONE MALEATE/GLIMEPIRIDE, SAXAGLIPTIN HCL, SITAGLIPTIN PHOSPHATE, SITAGLIPTIN PHOSPHATE/SIMVASTATIN, TROGLITAZONE, PIOGLITAZONE HCL/GLIMEPIRIDE, PIOGLITAZONE HCL/METFORMIN HCL
Corticosteroids	NDC Generic Name is any of: ACETIC ACID/HYDROCORTISONE, ACYCLOVIR/HYDROCORTISONE, BENZOYL PEROXIDE/HYDROCORTISONE, BENZOYL PEROXIDE/HYDROCORTISONE/SKIN CLEANSER COMB NO.14, CHLORCYCLIZINE HCL/HYDROCORTISONE ACETATE, CHLOROXYLENOL/BENZOCAINE/HYDROCORTISONE ACETATE, CIPROFLOXACIN HCL/DEXAMETHASONE, CIPROFLOXACIN HCL/HYDROCORTISONE, CLIOQUINOL/HYDROCORTISONE, CLIOQUINOL/HYDROCORTISONE/EMOLLIENT COMBINATION NO.88,

© 2023 Lin KJ et al. *JAMA Network Open.*

	CLIOQUINOL/HYDROCORTISONE/PRAMOXINE, CORTISONE ACETATE, DEXAMETHASONE, DEXAMETHASONE ACETATE, DEXAMETHASONE ACETATE, MICRONIZED, DEXAMETHASONE ISONICOTINATE, DEXAMETHASONE PHOSPHATE, DEXAMETHASONE PHOSPHATE/LIDOCAINE HCL, DEXAMETHASONE SOD PHOSPHATE, DEXAMETHASONE SODIUM PHOSPHATE IN 0.9 % SODIUM CHLORIDE, DEXAMETHASONE SODIUM PHOSPHATE/PF, DEXAMETHASONE, MICRONIZED, DIPHENHYDRAMINE HCL/HYDROCORTISONE, FLUDROCORTISONE ACETATE, GENTAMICIN SULFATE/PREDNISOLONE ACETATE, HYDROCORTISONE, HYDROCORTISONE ACETATE, HYDROCORTISONE ACETATE/ALOE POLYSACCHARIDE/IODOQUINOL, HYDROCORTISONE ACETATE/ALOE VERA, HYDROCORTISONE ACETATE/IODOQUINOL/ALOE POLYSACCHARIDES #2, HYDROCORTISONE ACETATE/IODOQUINOL/ALOE VERA, HYDROCORTISONE ACETATE/LIDOCAINE HCL/ALOE VERA, HYDROCORTISONE ACETATE/LIDOCAINE HCL/SKIN CLEANSER NO.6, HYDROCORTISONE ACETATE/PRAMOXINE HCL, HYDROCORTISONE ACETATE/PRAMOXINE HCL/ALOE POLYSACCHARIDE, HYDROCORTISONE ACETATE/PRAMOXINE HCL/EMOLLIENT BASE, HYDROCORTISONE ACETATE/PRAMOXINE HCL/SKIN CLEANSER NO.16, HYDROCORTISONE ACETATE/PRAMOXINE/EMOLLIENT/PRAMOXINE COMB#1, HYDROCORTISONE ACETATE/UREA, HYDROCORTISONE BUTYRATE, HYDROCORTISONE BUTYRATE/EMOLLIENT BASE, HYDROCORTISONE CYPIONATE, HYDROCORTISONE HEMISUCCINATE, HYDROCORTISONE PROBUTATE, HYDROCORTISONE SOD PHOSPHATE, HYDROCORTISONE SOD SUCCINATE, HYDROCORTISONE SODIUM SUCCINATE/PF, HYDROCORTISONE VALERATE, HYDROCORTISONE/ALOE POLYSACCHARIDE/IODOQUINOL, HYDROCORTISONE/ALOE VERA, HYDROCORTISONE/ALOE VERA/VITAMIN E ACETATE/VITAMINS A AND D, HYDROCORTISONE/BENZOCAINE/CHLOROXYLENOL, HYDROCORTISONE/COLLOIDAL OATMEAL/ALOE/VITAMIN E, HYDROCORTISONE/EMOLLIENT COMBINATION NO.45, HYDROCORTISONE/IODOQUINOL, HYDROCORTISONE/MINERAL OIL/PETROLATUM,WHITE, HYDROCORTISONE/PRAMOXINE HCL/CHLOROXYLENOL, HYDROCORTISONE/PRAMOXINE HCL/CHLOROXYLENOL/WATER, HYDROCORTISONE/PRAMOXINE/CHLOROXYLENOL/BENZALKONIUM, HYDROCORTISONE/RESORCINOL/BISMUTH SUBGALLATE/ZINC OXIDE, HYDROCORTISONE/SALICYLIC ACID/SULFUR,

© 2023 Lin KJ et al. *JAMA Network Open.*

	HYDROCORTISONE/SALICYLIC ACID/SULFUR/SHAMPOO CMB 1, HYDROCORTISONE/SKIN CLEANSER COMBINATION NO.25, HYDROCORTISONE/SKIN CLEANSER COMBINATION NO.35, HYDROCORTISONE/YERBA SANTA, KETOCONAZOLE/HYDROCORTISONE, LIDOCAINE HCL/HYDROCORTISONE ACETATE, LIDOCAINE HCL/HYDROCORTISONE ACETATE/PSYLLIUM HUSK, METHYLPREDNISOLONE, METHYLPREDNISOLONE ACETATE, METHYLPREDNISOLONE ACETATE, MICRONIZED, METHYLPREDNISOLONE ACETATE/BUPIVACAINE HCL, METHYLPREDNISOLONE SODIUM SUCCINATE, METHYLPREDNISOLONE SODIUM SUCCINATE/PF, METHYLPREDNISOLONE, MICRONIZED, NEOMYCIN SULFATE/BACITRACIN ZINC/POLYMYXIN B/HYDROCORTISONE, NEOMYCIN SULFATE/COLISTIN SULFATE/HYDROCORTISONE, NEOMYCIN SULFATE/COLISTIN SULFATE/HYDROCORTISONE/THONZONIUM, NEOMYCIN SULFATE/DEXAMETHASONE SOD PHOSPHATE, NEOMYCIN SULFATE/HYDROCORTISONE, NEOMYCIN SULFATE/HYDROCORTISONE ACETATE, NEOMYCIN SULFATE/POLYMYXIN B SULFATE/BUFFERS/HYDROCORTISONE, NEOMYCIN SULFATE/POLYMYXIN B SULFATE/HYDROCORTISONE, NEOMYCIN SULFATE/POLYMYXIN B SULFATE/PREDNISOLONE, NEOMYCIN/BACITRACIN/POLYMYXIN B/HYDROCORTISONE, NEOMYCIN/POLYMYXIN B SULFATE/DEXAMETHASONE, OXYTETRACYCLINE HCL/HYDROCORTISONE ACETATE, POLYMYXIN B SULFATE/HYDROCORTISONE, PREDNISOLONE, PREDNISOLONE ACETATE, PREDNISOLONE ACETATE, MICRONIZED, PREDNISOLONE SOD PHOSPHATE, PREDNISOLONE SODIUM PHOSPHATE/PEAK FLOW METER, PREDNISOLONE, MICRONIZED, PREDNISONE, PREDNISONE MICRONIZED, SULFACETAMIDE SODIUM/PREDNISOLONE ACETATE, SULFACETAMIDE SODIUM/PREDNISOLONE SODIUM PHOSPHATE, TOBRAMYCIN/DEXAMETHASONE
Estrogen	NDC Generic Name is any of: 2-METHOXYESTRADIOL, DESOGESTREL-ETHINYL ESTRADIOL, DESOGESTREL-ETHINYL ESTRADIOL/ETHINYL ESTRADIOL, DROSPIRENONE/ESTRADIOL, DROSPIRENONE/ETHINYL ESTRADIOL/LEVOMEFOLATE CALCIUM, ESTRADIOL, ESTRADIOL ACETATE, ESTRADIOL BENZOATE, ESTRADIOL CYPIONATE, ESTRADIOL CYPIONATE/MEDROXYPROGESTERONE ACETATE, ESTRADIOL HEMIHYDRATE, MICRONIZED, ESTRADIOL MICRONIZED, ESTRADIOL VALERATE, ESTRADIOL VALERATE/DIENOGEST, ESTRADIOL/LEVONORGESTREL, ESTRADIOL/NORETHINDRONE

© 2023 Lin KJ et al. *JAMA Network Open.*

	ACETATE, ESTRADIOL/NORGESTIMATE, ESTROGENS, CONJUGATED, ESTROGENS, CONJUGATED/BAZEDOXIFENE ACETATE, ESTROGENS, CONJUGATED/MEDROXYPROGESTERONE ACETATE, ESTROGENS, CONJUGATED/MEPROBAMATE, ESTROGENS,CONJ.,SYNTHETIC A, ESTROGENS,CONJ.,SYNTHETIC B, ESTROGENS,ESTERIFIED, ESTROGENS,ESTERIFIED/METHYLTESTOSTERONE, ESTROPIPATE, ETHINYL ESTRADIOL, ETHINYL ESTRADIOL/DROSPIRENONE, ETHYNODIOL DIACETATE-ETHINYL ESTRADIOL, ETONOGESTREL/ETHINYL ESTRADIOL, LEVONORGESTREL-ETH ESTRA/ETHINYL ESTRADIOL, LEVONORGESTREL-ETHINYL ESTRADIOL, LEVONORGESTREL-ETHINYL ESTRADIOL/PREGNANCY TEST KIT, NORELGESTROMIN/ETHINYL ESTRADIOL, NORETHINDRONE ACETATE-ETHINYL ESTRADIOL, NORETHINDRONE ACETATE-ETHINYL ESTRADIOL/FERROUS FUMARATE, NORETHINDRONE-ETHINYL ESTRADIOL, NORETHINDRONE-ETHINYL ESTRADIOL/FERROUS FUMARATE, NORGESTIMATE-ETHINYL ESTRADIOL, NORGESTREL-ETHINYL ESTRADIOL, TESTOSTERONE CYPIONATE/ESTRADIOL CYPIONATE, TESTOSTERONE ENANTHATE/ESTRADIOL VALERATE
Thyroid hormone replacement	NDC Generic Name is any of: LEVOTHYROXINE SODIUM, LIOTHYRONINE SODIUM, LIOTHYRONINE SODIUM, MICRONIZED, LIOTRIX
H2 blockers	NDC Generic Name is any of: CIMETIDINE, CIMETIDINE HCL, CIMETIDINE HCL IN 0.9 % SODIUM CHLORIDE, FAMOTIDINE, FAMOTIDINE IN 0.9 % SODIUM CHLORIDE, FAMOTIDINE IN SODIUM CHLORIDE, ISO-OSMOTIC/PF, FAMOTIDINE/CALCIUM CARBONATE/MAGNESIUM HYDROXIDE, FAMOTIDINE/PF, IBUPROFEN/FAMOTIDINE, NIZATIDINE, RANITIDINE BISMUTH CITRATE, RANITIDINE HCL, RANITIDINE HCL IN 0.45 % SODIUM CHLORIDE, RANITIDINE HCL/DIETARY SUPPLEMENT,MISC COMB17, RANITIDINE HCL/DIETARY SUPPLEMENT,MISC.COMBO8
Proton-pump inhibitors	NDC Generic Name is any of: ESOMEPRAZOLE MAGNESIUM, ESOMEPRAZOLE SODIUM, ESOMEPRAZOLE STRONTIUM, LANSOPRAZOLE, LANSOPRAZOLE/AMOXICILLIN TRIHYDRATE/CLARITHROMYCIN, LANSOPRAZOLE/NAPROXEN, NAPROXEN/ESOMEPRAZOLE MAGNESIUM, OMEPRAZOLE, OMEPRAZOLE MAGNESIUM, OMEPRAZOLE/CLARITHROMYCIN/AMOXICILLIN TRIHYDRATE, OMEPRAZOLE/SODIUM BICARBONATE, PANTOPRAZOLE SODIUM, RABEPRAZOLE SODIUM
Other antacids: sucralfate	NDC Generic Name is any of: SUCRALFATE, SUCRALFATE MALATE, POLYMERIZED

© 2023 Lin KJ et al. *JAMA Network Open.*

Anticonvulsants	**NDC Generic Name** is any of: AMINOPHYLLINE/EPHEDRINE/POTASSIUM IODIDE/PHENOBARBITAL, BELLADONNA ALKALOIDS/PHENOBARBITAL, CARBAMAZEPINE, ERGOTAMINE TARTRATE/BELLADONNA ALKALOIDS/PHENOBARBITAL, ETHOSUXIMIDE, EZOGABINE, FELBAMATE, GABAPENTIN, GABAPENTIN ENACARBIL, GABAPENTIN/DIETARY SUPPLEMENT, MISC COMBO NO.11, GUAIFENESIN/DYPHYLLINE/EPHEDRINE/PHENOBARBITAL, HYOSCYAMINE SULFATE/PHENOBARBITAL, LACOSAMIDE, LAMOTRIGINE, LEVETIRACETAM, LEVETIRACETAM IN SODIUM CHLORIDE, ISO-OSMOTIC, OXCARBAZEPINE, PHENOBARBITAL, PHENOBARBITAL SODIUM, PHENOBARBITAL SODIUM IN 0.9 % SODIUM CHLORIDE, PHENOBARBITAL/HYOSCYAMINE SULF/ATROPINE SULF/SCOPOLAMINE HB, PHENTERMINE HCL/TOPIRAMATE, PHENYTOIN, PHENYTOIN SODIUM, PHENYTOIN SODIUM EXTENDED, PREGABALIN, PRIMIDONE, PROPANTHELINE BROMIDE/PHENOBARBITAL, RUFINAMIDE, THEOPHYLLINE/EPHEDRINE HCL/PHENOBARBITAL, THEOPHYLLINE/EPHEDRINE/POTASSIUM IODIDE/PHENOBARBITAL, TIAGABINE HCL, TOPIRAMATE, VALPROIC ACID (AS SODIUM SALT) (VALPROATE SODIUM), VIGABATRIN, ZONISAMIDE
Dementia drugs	**NDC Generic Name** is any of: DONEPEZIL HCL, MEMANTINE HCL/DONEPEZIL HCL, RIVASTIGMINE, RIVASTIGMINE TARTRATE, GALANTAMINE HBR, MEMANTINE HCL
Parkinsonism drugs	**NDC Generic Name** is any of: LEVODOPA, CARBIDOPA/LEVODOPA, CARBIDOPA/LEVODOPA/ENTACAPONE, ROPINIROLE HCL, PRAMIPEXOLE DI-HCL, ROTIGOTINE, AMANTADINE HCL, TRIHEXYPHENIDYL HCL, BENZTROPINE MESYLATE, SELEGILINE, SELEGILINE HCL, RASAGILINE MESYLATE, TOLCAPONE, ENTACAPONE, APOMORPHINE HCL
NSAIDs	**NDC Generic Name** is any of: CELECOXIB, DIFLUNISAL, ETODOLAC, FLURBIPROFEN, IBUPROFEN, INDOMETHACIN, KETOPROFEN, MELOXICAM, NABUMETONE, NAPROXEN, OXAPROZIN, PIROXICAM, ROFECOXIB, SULINDAC, VALDECOXIB
Opioids	**NDC Generic Name** is any of: ACETAMINOPHEN WITH CODEINE PHOSPHATE, AMMONIUM CHL/POTASSIUM GUAICO/CODEINE/DIPHEN/BROMODIPHENHYD, AMMONIUM CHLORIDE/PHENYLEPHRINE HCL/CODEINE/CHLORPHENIRAMINE, ASPIRIN/CODEINE PHOSPHATE, BROMPHENIRAMINE MALEATE/CODEINE PHOSPHATE, BROMPHENIRAMINE MALEATE/PHENYLEPHRINE HCL/CODEINE PHOSPHATE,

© 2023 Lin KJ et al. *JAMA Network Open.*

	BROMPHENIRAMINE MALEATE/PHENYLEPHRINE HCL/DIHYDROCODEINE BT, BROMPHENIRAMINE MALEATE/PSEUDOEPHEDRINE HCL/CODEINE, BROMPHENIRAMINE/PSEUDOEPHEDRINE HCL/DIHYDROCODEINE, BUTALBITAL/ACETAMINOPHEN/CAFFEINE/CODEINE PHOSPHATE, CHLORCYCLIZINE HCL/CODEINE PHOSPHATE, CHLORCYCLIZINE HCL/PHENYLEPHRINE HCL/CODEINE PHOSPHATE, CHLORCYCLIZINE HCL/PSEUDOEPHEDRINE HCL/CODEINE PHOSPHATE, CHLORPHENIRAMINE MALEATE/CODEINE PHOSPHATE, CHLORPHENIRAMINE MALEATE/CODEINE PHOSPHATE/ACETAMINOPHEN, CHLORPHENIRAMINE MALEATE/PHENYLEPHRINE HCL/CODEINE PHOSPHATE, CHLORPHENIRAMINE MALEATE/PSEUDOEPHEDRINE HCL/CODEINE, CODEINE PHOSPHATE, CODEINE PHOSPHATE/BROMODIPHENHYDRAMINE HCL, CODEINE PHOSPHATE/BUTALBITAL/ASPIRIN/CAFFEINE, CODEINE PHOSPHATE/CARISOPRODOL/ASPIRIN, CODEINE PHOSPHATE/PYRILAMINE MALEATE, CODEINE POLISTIREX/CHLORPHENIRAMINE POLISTIREX, CODEINE SULFATE, CODEINE/ASPIRIN/SALICYLAMIDE/ACETAMINOPHEN/CAFFEINE, CODEINE/CALCIUM IODIDE, DEXBROMPHENIRAMINE MALEATE/PSEUDOEPHEDRINE HCL/CODEINE PHOS, DEXCHLORPHENIRAMINE MALEATE/PHENYLEPHRINE HCL/CODEINE, DIHYDROCODEINE BITARTRATE, DIHYDROCODEINE BITARTRATE/ACETAMINOPHEN/CAFFEINE, DIHYDROCODEINE BITARTRATE/ASPIRIN/CAFFEINE, DIHYDROCODEINE BITARTRATE/GUAIFENESIN, DIHYDROCODEINE/ASPIRIN/CAFFEINE, DIPHENHYDRAMINE HCL/PHENYLEPHRINE HCL/CODEINE PHOSPHATE, FENTANYL, FENTANYL CITRATE, FENTANYL CITRATE IN 0.9 % SODIUM CHLORIDE/PF, FENTANYL CITRATE/BUPIVACAINE HCL IN 0.9 % SODIUM CHLORIDE/PF, FENTANYL CITRATE/DEXTROSE 5%-WATER/PF, FENTANYL CITRATE/DROPERIDOL, FENTANYL CITRATE/PF, FENTANYL CITRATE/ROPIVACAINE HCL/SODIUM CHLORIDE 0.9%/PF, FENTANYL HCL, GUAIFENESIN/CODEINE PHOSPHATE, GUAIFENESIN/HYDROCODONE BITARTRATE, GUAIFENESIN/HYDROCODONE BITARTRATE/BROMPHENIRAMINE, GUAIFENESIN/PHENYLEPHRINE HCL/HYDROCODONE, GUAIFENESIN/PHENYLEPHRINE HCL/PHENYLPROP/HYDROCODONE/PHENIR, GUAIFENESIN/PHENYLPROPANOLAMINE HCL/CODEINE, GUAIFENESIN/PHENYLPROPANOLAMINE

© 2023 Lin KJ et al. *JAMA Network Open.*

	HCL/HYDROCODONE/PYRIL/PHENIR, GUAIFENESIN/PSEUDOEPHEDRINE HCL/DIHYDROCODEINE, GUAIFENESIN/PSEUDOEPHEDRINE HCL/HYDROCODONE/CHLORPHENIRAMINE, HYDROCODONE BITARTRATE, HYDROCODONE BITARTRATE/ACETAMINOPHEN, HYDROCODONE BITARTRATE/ACETAMINOPHEN/DIETARY SUPPLEMENT #11, HYDROCODONE BITARTRATE/ASPIRIN, HYDROCODONE BITARTRATE/CHLORPHENIRAMINE MALEATE, HYDROCODONE BITARTRATE/HOMATROPINE, HYDROCODONE BITARTRATE/HOMATROPINE METHYLBROMIDE, HYDROCODONE BITARTRATE/PSEUDOEPHEDRINE HCL/GUAIFENESIN, HYDROCODONE POLISTIREX/CHLORPHENIRAMINE POLISTIREX, HYDROCODONE TANNATE/CHLORPHENIRAMINE TANNATE, HYDROCODONE/IBUPROFEN, HYDROMORPHONE HCL, HYDROMORPHONE HCL IN 0.9 % SODIUM CHLORIDE, HYDROMORPHONE HCL IN 0.9 % SODIUM CHLORIDE/PF, HYDROMORPHONE HCL IN DEXTROSE 5 %-WATER/PF, HYDROMORPHONE HCL/BUPIVACAINE HCL IN 0.9% SODIUM CHLORIDE/PF, HYDROMORPHONE HCL/GUAIFENESIN, HYDROMORPHONE HCL/PF, IBUPROFEN/OXYCODONE HCL, LEVORPHANOL TARTRATE, MEPERIDINE HCL, MEPERIDINE HCL IN 0.9 % SODIUM CHLORIDE, MEPERIDINE HCL/ATROPINE SULFATE, MEPERIDINE HCL/PF, MEPERIDINE HCL/PROMETHAZINE HCL, METHADONE HCL, METHADONE HYDROCHLORIDE IN 0.9 % SODIUM CHLORIDE, MORPHINE SULFATE, MORPHINE SULFATE IN 0.9 % SODIUM CHLORIDE, MORPHINE SULFATE IN 0.9 % SODIUM CHLORIDE/PF, MORPHINE SULFATE IN SODIUM CHLORIDE, ISO-OSMOTIC/PF, MORPHINE SULFATE LIPOSOMAL/PF, MORPHINE SULFATE/DEXTROSE 5 % IN WATER, MORPHINE SULFATE/DEXTROSE 5%-WATER/PF, MORPHINE SULFATE/NALTREXONE HCL, MORPHINE SULFATE/PF, OXYCODONE HCL, OXYCODONE HCL/ACETAMINOPHEN, OXYCODONE HCL/ASPIRIN, OXYCODONE HCL/OXYCODONE TEREPHTHALATE/ASPIRIN, OXYCODONE/ASPIRIN, OXYMORPHONE HCL, PENTAZOCINE HCL/ACETAMINOPHEN, PENTAZOCINE HCL/ASPIRIN, PENTAZOCINE HCL/NALOXONE HCL, PENTAZOCINE LACTATE, PHENYLEPHRINE HCL/CODEINE PHOSPHATE, PHENYLEPHRINE HCL/CODEINE PHOSPHATE/ACETAMINOPHEN/GUAIFEN, PHENYLEPHRINE HCL/CODEINE PHOSPHATE/GUAIFENESIN, PHENYLEPHRINE HCL/CODEINE PHOSPHATE/PHENIRAMINE/SODIUM CIT, PHENYLEPHRINE HCL/CODEINE/ACETAMINOPHEN/CHLORPHENIRAMINE, PHENYLEPHRINE HCL/CODEINE/CHLORPHENIRAMINE, PHENYLEPHRINE

© 2023 Lin KJ et al. *JAMA Network Open.*

	HCL/CODEINE/CHLORPHENIRAMINE/POTASSIUM IODIDE, PHENYLEPHRINE HCL/CODEINE/PYRILAMINE, PHENYLEPHRINE HCL/DIHYDROCODEINE BITARTRATE, PHENYLEPHRINE HCL/DIHYDROCODEINE BITARTRATE/CHLORPHENIRAMINE, PHENYLEPHRINE HCL/DIHYDROCODEINE BITARTRATE/GUAIFENESIN, PHENYLEPHRINE HCL/HYDROCODONE BITARTRATE, PHENYLEPHRINE HCL/HYDROCODONE BITARTRATE/BROMPHENIRAMINE, PHENYLEPHRINE HCL/HYDROCODONE BITARTRATE/CARBINOXAMINE, PHENYLEPHRINE HCL/HYDROCODONE BITARTRATE/CHLORPHENIRAMINE, PHENYLEPHRINE HCL/HYDROCODONE BITARTRATE/DEXBROMPHENIRAMINE, PHENYLEPHRINE HCL/HYDROCODONE BITARTRATE/DEXCHLORPHENIRAMINE, PHENYLEPHRINE HCL/HYDROCODONE BITARTRATE/DIPHENHYDRAMINE, PHENYLEPHRINE HCL/HYDROCODONE BITARTRATE/PYRIL/CHLORPHENIR, PHENYLEPHRINE HCL/HYDROCODONE BITARTRATE/PYRILAMINE, PHENYLEPHRINE HCL/PPA HCL/DIHYDROCODEINE/CHLORPHENIRAMINE, PHENYLEPHRINE TANNATE/HYDROCODONE TANNATE/DIPHENHYDRAMINE, PHENYLEPHRINE/HYDROCODONE BIT/ACETAMINOPHEN/CHLORPHENIRAMINE, PHENYLEPHRINE/HYDROCODONE/BROMPHENIRAMINE TANNATES, PHENYLEPHRINE/HYDROCODONE/DEXCHLORPHENIRAMINE TANNATES, PHENYLEPHRINE/PSEUDOEPHED/HYDROCODONE/PYRILAMINE/CHLORPHENIR, PHENYLPROPANOLAMINE HCL/CODEINE/BROMPHENIRAMINE, PHENYLPROPANOLAMINE HCL/HYDROCODONE BITARTRATE, POTASSIUM GUAIACOLSULFONATE/HYDROCODONE BITARTRATE, POTASSIUM GUAIACOLSULFONATE/PSEUDOEPHEDRINE HCL/HYDROCODONE, PROMETHAZINE HCL/CODEINE, PROMETHAZINE/PHENYLEPHRINE HCL/CODEINE, PROPOXYPHENE HCL, PROPOXYPHENE HCL/ACETAMINOPHEN, PROPOXYPHENE HCL/ASPIRIN/CAFFEINE, PROPOXYPHENE NAPSYLATE, PROPOXYPHENE NAPSYLATE/ACETAMINOPHEN, PSEUDOEPHEDRINE HCL/CODEINE PHOSPHATE, PSEUDOEPHEDRINE HCL/CODEINE PHOSPHATE/ACETAMINOPHEN/GUAIFEN, PSEUDOEPHEDRINE HCL/CODEINE PHOSPHATE/GUAIFENESIN, PSEUDOEPHEDRINE HCL/CODEINE PHOSPHATE/TRIPROLIDINE, PSEUDOEPHEDRINE HCL/CODEINE/CHLORPHENIRAMINE, PSEUDOEPHEDRINE HCL/DIHYDROCODEINE BITARTRATE/CHLORPHENIR,

© 2023 Lin KJ et al. *JAMA Network Open.*

	PSEUDOEPHEDRINE HCL/HYDROCODONE BITARTRATE, PSEUDOEPHEDRINE HCL/HYDROCODONE BITARTRATE/BROMPHENIRAMINE, PSEUDOEPHEDRINE HCL/HYDROCODONE BITARTRATE/CARBINOXAMINE, PSEUDOEPHEDRINE HCL/HYDROCODONE BITARTRATE/CHLORPHENIRAMINE, PSEUDOEPHEDRINE HCL/HYDROCODONE BITARTRATE/TRIPROLIDINE, PSEUDOEPHEDRINE TANNATE/HYDROCODONE TANNATE, PSEUDOEPHEDRINE/HYDROCODONE/BROMPHENIRAMINE TANNATES, PSEUDOEPHEDRINE/HYDROCODONE/CHLORPHENIRAMINE TANNATES, PYRILAMINE MALEATE/PHENYLEPHRINE HCL/CODEINE PHOSPHATE, PYRILAMINE MALEATE/PHENYLEPHRINE HCL/DIHYDROCODEINE BT, PYRILAMINE MALEATE/PSEUDOEPHEDRINE HCL/CODEINE PHOSPHATE, TAPENTADOL HCL, TRAMADOL HCL, TRAMADOL HCL/ACETAMINOPHEN, TRAMADOL HCL/DIETARY SUPPLEMENT,MISC. CB.11
Antipsychotic agents	NDC Generic Name is any of: ACEPROMAZINE MALEATE, ARIPIPRAZOLE, ASENAPINE MALEATE, CHLORPROMAZINE HCL, CLOZAPINE, FLUPHENAZINE DECANOATE, FLUPHENAZINE ENANTHATE, FLUPHENAZINE HCL, HALOPERIDOL, HALOPERIDOL DECANOATE, HALOPERIDOL LACTATE, ILOPERIDONE, LOXAPINE, LOXAPINE HCL, LOXAPINE SUCCINATE, LURASIDONE HCL, MESORIDAZINE BESYLATE, MOLINDONE HCL, OLANZAPINE, OLANZAPINE PAMOATE, OLANZAPINE/FLUOXETINE HCL, PALIPERIDONE, PALIPERIDONE PALMITATE, PERPHENAZINE, PERPHENAZINE/AMITRIPTYLINE HCL, PIMOZIDE, PROMAZINE HCL, QUETIAPINE FUMARATE, RISPERIDONE, RISPERIDONE MICROSPHERES, THIORIDAZINE HCL, THIOTHIXENE, THIOTHIXENE HCL, TRIFLUOPERAZINE HCL, TRIFLUPROMAZINE HCL, ZIPRASIDONE HCL, ZIPRASIDONE MESYLATE
Anxiolytics (except benzodiazepines)	**NDC Generic Name** is any of: BUSPIRONE HCL, MEPROBAMATE, ASPIRIN/MEPROBAMATE, ESTROGENS, CONJUGATED/MEPROBAMATE
Benzodiazepines	NDC Generic Name is any of: ALPRAZOLAM, ALPRAZOLAM/DIETARY SUPPLEMENT,MISC COMBO NO.17, AMITRIPTYLINE HCL/CHLORDIAZEPOXIDE, CHLORDIAZEPOXIDE, CHLORDIAZEPOXIDE HCL, CHLORDIAZEPOXIDE HCL/METHSCOPOLAMINE NITRATE, CHLORDIAZEPOXIDE/CLIDINIUM BROMIDE, CLOBAZAM, CLONAZEPAM, CLORAZEPATE DIPOTASSIUM, DIAZEPAM, DIAZEPAM/SOYBEAN OIL, ESTAZOLAM, FLURAZEPAM HCL, LORAZEPAM, LORAZEPAM IN 0.9 % SODIUM CHLORIDE, LORAZEPAM/DEXTROSE 5 % IN WATER, MIDAZOLAM, MIDAZOLAM HCL, MIDAZOLAM HCL IN 0.9 % SODIUM CHLORIDE, MIDAZOLAM HCL IN 0.9 % SODIUM CHLORIDE/PF,

© 2023 Lin KJ et al. *JAMA Network Open.*

	MIDAZOLAM HCL IN DEXTROSE 5% IN WATER, MIDAZOLAM HCL/DEXTROSE 5%-WATER/PF, MIDAZOLAM HCL/PF, OXAZEPAM, QUAZEPAM, TEMAZEPAM, TEMAZEPAM/DIETARY SUPPLEMENT,MISC.COMBO8, TRIAZOLAM
Hypnotics	NDC Generic Name is any of: ACETAMINOPHEN/DIPHENHYDRAMINE HCL, AMINOGLUTETHIMIDE, ASPIRIN/DIPHENHYDRAMINE HCL, ASPIRIN/DIPHENHYDRAMINE/SODIUM BICARBONATE/CITRIC ACID, ASPIRIN/MEPROBAMATE, BROMPHENIRAMINE MAL/DIPHENHYDRAMINE HCL/PHENYLEPHRINE HCL, BROMPHENIRAMINE MALEATE/DIPHENHYDRAMINE HCL, BUSPIRONE HCL, CARBETAPENTANE TANNATE/PHENYLEPHRINE TANNATE/DIPHENHYDRAMINE, CHLORAL HYDRATE, DEXTROMETHORPHAN HBR/ACETAMINOPHEN/DIPHENHYDRAMINE HCL, DIPHENHYDRAMINE HCL, DIPHENHYDRAMINE HCL IN 0.9 % SODIUM CHLORIDE, DIPHENHYDRAMINE HCL/ALLANTOIN, DIPHENHYDRAMINE HCL/BENZETHONIUM CHLORIDE/ZINC ACETATE, DIPHENHYDRAMINE HCL/CALAMINE, DIPHENHYDRAMINE HCL/CAMPHOR/CALAMINE, DIPHENHYDRAMINE HCL/HYDROCORTISONE, DIPHENHYDRAMINE HCL/MENTHOL, DIPHENHYDRAMINE HCL/PHENYLEPHRINE HCL/ACETAMINOPHEN, DIPHENHYDRAMINE HCL/PHENYLEPHRINE HCL/ACETAMINOPHEN/GUAIFEN, DIPHENHYDRAMINE HCL/PHENYLEPHRINE HCL/CODEINE PHOSPHATE, DIPHENHYDRAMINE HCL/PHENYLEPHRINE HCL/DEXTROMETHORPHAN HBR, DIPHENHYDRAMINE HCL/TRIPELENNAMINE HCL/MENTHOL, DIPHENHYDRAMINE HCL/ZINC ACETATE, DIPHENHYDRAMINE/PHENYLEPHRIN/DEXTROMETHORPH/ACETAMINOPHEN/GG, ESTROGENS, CONJUGATED/MEPROBAMATE, ESZOPICLONE, ETHCHLORVYNOL, HYDROXYZINE HCL, HYDROXYZINE PAMOATE, IBUPROFEN/DIPHENHYDRAMINE HCL, MAGNESIUM SALICYLATE/DIPHENHYDRAMINE HCL, MEPROBAMATE, NAPROXEN SODIUM/DIPHENHYDRAMINE HCL, PHENYLEPHRINE HCL/DIPHENHYDRAMINE HCL, PHENYLEPHRINE HCL/HYDROCODONE BITARTRATE/DIPHENHYDRAMINE, PHENYLEPHRINE TANNATE/HYDROCODONE TANNATE/DIPHENHYDRAMINE, PHENYLEPHRINE/ACETAMINOPHEN/DIPHENHYDRAMINE/CHLORPHENIRAMINE, PHENYLPROPANOLAMINE HCL/ASPIRIN/DIPHENHYDRAMINE, PRAMOXINE HCL/DIPHENHYDRAMINE HCL, PSEUDOEPHEDRINE HCL/ACETAMINOPHEN/DIPHENHYDRAMINE, PSEUDOEPHEDRINE HCL/DIPHENHYDRAMINE HCL,

© 2023 Lin KJ et al. *JAMA Network Open.*

	THEOPHYLLINE/EPHEDRINE/HYDROXYZINE, ZALEPLON, ZOLPIDEM TARTRATE
SSRI/SNRI antidepressants	NDC Generic Name is any of: CITALOPRAM HYDROBROMIDE, DULOXETINE HCL, ESCITALOPRAM OXALATE, FLUOXETINE, FLUOXETINE HCL, FLUOXETINE HCL/DIETARY SUPPLEMENT,MISC COMB17, FLUOXETINE HCL/DIETARY SUPPLEMENT,MISC.COMBO8, FLUVOXAMINE MALEATE, OLANZAPINE/FLUOXETINE HCL, PAROXETINE HCL, PAROXETINE MESYLATE, SERTRALINE HCL, DESVENLAFAXINE, DESVENLAFAXINE FUMARATE, DESVENLAFAXINE SUCCINATE, VENLAFAXINE HCL, LEVOMILNACIPRAN HYDROCHLORIDE
Tricyclic antidepressants	NDC Generic Name is any of: AMITRIPTYLINE HCL, AMITRIPTYLINE HCL/CHLORDIAZEPOXIDE, AMOXAPINE, DESIPRAMINE HCL, DOXEPIN HCL, IMIPRAMINE HCL, IMIPRAMINE PAMOATE, NORTRIPTYLINE HCL, PERPHENAZINE/AMITRIPTYLINE HCL, PROTRIPTYLINE HCL, TRIMIPRAMINE MALEATE, CLOMIPRAMINE HCL
Other antidepressants	NDC Generic Name is any of: BUPROPION HBR, BUPROPION HCL, BUPROPION HCL/DIETARY SUPPLEMENT,MISC COMB15, BUPROPION HCL/DIETARY SUPPLEMENT,MISC COMB16, ISOCARBOXAZID, MIRTAZAPINE, NALTREXONE HCL/BUPROPION HCL, NEFAZODONE HCL, PHENELZINE SULFATE, TRAZODONE HCL, TRAZODONE HCL/DIETARY SUPPLEMENT,MISC.COMBO8, VILAZODONE HYDROCHLORIDE, VORTIOXETINE HYDROBROMIDE, MAPROTILINE HCL
Bronchodilators	NDC Generic Name is any of: ALBUTEROL, ALBUTEROL SULFATE, IPRATROPIUM BROMIDE/ALBUTEROL SULFATE, LEVALBUTEROL HCL, LEVALBUTEROL TARTRATE, METAPROTERENOL SULFATE, PIRBUTEROL ACETATE, SALMETEROL XINAFOATE, FLUTICASONE PROPIONATE/SALMETEROL XINAFOATE, FORMOTEROL FUMARATE, ARFORMOTEROL TARTRATE, BUDESONIDE/FORMOTEROL FUMARATE, MOMETASONE FUROATE/FORMOTEROL FUMARATE
Corticosteroids, inhaled	NDC Generic Name is any of: AZELASTINE HCL/FLUTICASONE PROPIONATE, BECLOMETHASONE DIPROPIONATE, BUDESONIDE, BUDESONIDE, MICRONIZED, BUDESONIDE/FORMOTEROL FUMARATE, CICLESONIDE, FLUNISOLIDE, FLUNISOLIDE/MENTHOL, FLUTICASONE FUROATE, FLUTICASONE FUROATE/VILANTEROL TRIFENATATE, FLUTICASONE PROPIONATE, FLUTICASONE PROPIONATE/SALMETEROL XINAFOATE, MOMETASONE FUROATE, MOMETASONE FUROATE/AMMONIUM LACTATE, MOMETASONE FUROATE/FORMOTEROL FUMARATE

© 2023 Lin KJ et al. *JAMA Network Open.*

Endoscopy	ICD-9 Procedure Code (any position) is any of: 45.1, 45.11, 45.12, 45.13, 45.14, 45.15, 45.16, 45.19, 45.2, 45.21, 45.22, 45.23, 45.24, 45.25, 45.26, 45.27, 45.28, 45.29<br>ICD-10 Procedure Code (any position) is any of: 0D927ZX, 0D958ZX, 0D987ZX, 0D9A7ZX, 0D9A8ZX, 0D9B7ZX, 0DB28ZX, 0DB47ZX, 0DB67ZX, 0DJ08ZZ, 0D928ZX, 0D947ZX, 0D957ZX, 0D988ZX, 0D9B8ZX, 0DB27ZX, 0DB57ZX, 0DB77ZX, 0DJ68ZZ, 0D917ZX, 0D938ZX, 0D948ZX, 0D9C4ZX, 0DB17ZX, 0DB48ZX, 0D918ZX, 0D967ZX, 0D977ZX, 0D997ZX, 0D998ZX, 0D9C7ZX, 0D9C8ZX, 0DB18ZX, 0D937ZX, 0D968ZX, 0D978ZX, 0DB37ZX, 0DB38ZX, 0DB58ZX, 0DB68ZX, 0DB78ZX, 0DB97ZX, 0DB98ZX
Liver disease	ICD-9 Diagnosis Code (any position) is any of: 070.2, 070.21, 070.52, 070.70, 456.21, 573.0, 573.1, 573.2, 573.5, 573.9, 576.8, 782.4, 070.0, 070.1, 070.20, 070.22, 070.23, 070.3, 070.41, 070.5, 070.51, 070.53, 070.71, 070.30, 070.4, 070.54, 070.59, 070.7, 570, 573.3, 573.4, 573.8, 070.31, 070.32, 070.33, 070.44, 070.49, 070.6, 070.9, 456.1, 456.2, 070.42, 070.43, 185.0, 456.0, 789.51, 789.57, 789.58, 789.59, 185.05, 198.30, 456.20, 789.5, 571.0, 571.1, 571.3, 571.40, 571.49, 571.5, 571.6, 571.8, 572, 572.0, 572.3, 572.4, 571, 571.2, 571.4, 571.41, 571.42, 571.9, 572.1, 572.2, 572.8<br>ICD-10 Diagnosis Code (any position) is any of: B16.1, B17.11, B17.2, B17.9, B18.2, B18.8, B19.0, B19.9, I85.11, K87, B15.9, B16.0, B16.2, B16.9, B18.0, B18.9, B19.10, B19.11, K76.2, K83.8, R17, B15.0, B19.21, K72.00, B17.0, B17.10, B17.8, B18.1, B19.20, I85.10, K83.5, I85.0, K74.6, R18, R18.0, R18.8, I85.00, I85.01, K70.10, K70.11, K70.2, K70.31, K70.40, K70.41, K71.0, K71.10, K71.11, K71.2, K71.3, K71.51, K71.6, K72.01, K72.10, K72.90, K72.91, K73.2, K73.8, K74.1, K74.2, K74.69, K75.0, K75.1, K75.4, K75.81, K75.89, K76.0, K76.3, K76.4, K76.5, K76.6, K76.7, K76.9, K77, B25.1, K70.0, K70.30, K70.9, K71.4, K71.50, K71.7, K71.8, K71.9, K72.11, K73.0, K73.1, K73.9, K74.0, K74.3, K74.4, K74.5, K74.60, K75.2, K75.3, K75.9, K76.1, K76.81, K76.89<br>ICD-9 Procedure Code (any position) is any of: 39.1, 42.91<br>ICD-10 Procedure Code (any position) is any of: 06100Z5, 0610496, 06104K5, 06104Z5, 06104ZY, 061107B, 0611099, 06110AY, 06110J9, 0611479, 061147Y, 06114J9, 06120ZY, 06140AY, 061507Y, 061509Y, 061549Y, 06154AY, 061647Y, 061709Y, 061807Y, 061809B, 061809Y, 06180J9, 06180Z9, 0618479, 061849B, 06184AB, 06184JB, 06184ZB, 06190ZY, 061947Y, 061949Y, 061B0ZY, 061B4AY, 061B4KY, 061J09Y, 061J0KY, 06L30ZZ, 061007Y, 06100A5, 06100J5, 06100K6, 06110JB, 06110KB, 06114JY, 061207Y, 061407Y, 061409Y, 061449Y, 06144AY, 06150KY, 06154ZY, 061607Y, 06160ZY, 06164AY, 061749Y, 06174AY, 06174KY, 0618099, 06180K9, 06184J9, 06184JY, 06184Z9, 061909Y, 061J4JY, 06L34CZ, 0610075, 06100A6, 06100K5, 06100KY, 06100ZY, 061049Y,

© 2023 Lin KJ et al. *JAMA Network Open.*

	06104A5, 06104A6, 06104JY, 06104Z6, 061107Y, 06110KY, 06110ZB, 061149Y, 06114A9, 06114AB, 06114AY, 06114ZB, 06140KY, 06140ZY, 06144ZY, 06150AY, 06150ZY, 061609Y, 061649Y, 06164JY, 06164KY, 06164ZY, 06170KY, 061807B, 06180JB, 06180KY, 06180ZY, 061847Y, 06184DY, 06184KY, 06190AY, 06194JY, 061B07Y, 061B0AY, 061B4ZY, 061J07Y, 061J4ZY, 06L33DZ, 06L34DZ, 0610096, 061009Y, 06100Z6, 0610476, 06104AY, 06104J5, 06104J6, 06104KY, 06110JY, 06110K9, 06110ZY, 0611499, 06114JB, 06114K9, 06114KB, 06114KY, 06114Z9, 06114ZY, 06120KY, 061247Y, 061249Y, 06124AY, 06150JY, 06154JY, 06160KY, 061747Y, 06174ZY, 0618079, 06180AY, 06180JY, 06180KB, 06180ZB, 06183DY, 061847B, 0618499, 06184AY, 06184K9, 06184ZY, 061907Y, 06194ZY, 061B0JY, 061B0KY, 061B47Y, 061B4JY, 061J0AY, 061J0ZY, 061J47Y, 06L30CZ, 06L30DZ, 06L33CZ, 06L33ZZ, 0610076, 0610095, 06100AY, 06100J6, 06100JY, 0610475, 061047Y, 0610495, 06104K6, 0611079, 061109B, 061109Y, 06110A9, 06110AB, 06110Z9, 061147B, 061149B, 061209Y, 06120AY, 06120JY, 06124JY, 06124KY, 06124ZY, 06140JY, 061447Y, 06144JY, 06144KY, 061547Y, 06154KY, 06160AY, 06160JY, 061707Y, 06170AY, 06170JY, 06170ZY, 06174JY, 06180A9, 06180AB, 061849Y, 06184A9, 06184KB, 06190JY, 06190KY, 06194AY, 06194KY, 061B09Y, 061B49Y, 061J0JY, 061J49Y, 061J4AY, 061J4KY, 06L34ZZ
Peptic Ulcer	ICD-9 Diagnosis Code (any position) is any of: 533, 533.0, 533.00, 533.01, 533.11, 533.20, 533.3, 533.31, 533.40, 533.6, 533.60, 533.61, 533.7, 533.71, 533.90, V12.71, 533.1, 533.10, 533.2, 533.21, 533.30, 533.4, 533.41, 533.5, 533.50, 533.51, 533.70, 533.9, 533.91<br>ICD-10 Diagnosis Code (any position) is any of: K27, Z87.11, K25.4, K25.7, K25.9, K26.1, K26.4, K26.5, K26.6, K26.7, K27.2, K27.5, K27.6, K27.7, K28.0, K28.4, K28.5, K28.7, K56.699, K25.1, K25.5, K26.0, K26.2, K26.3, K26.9, K27.0, K27.1, K27.3, K27.4, K27.9, K28.1, K28.2, K28.3, K28.6, K28.9
Obesity	HCPCS Procedure Code (any position) is any of: 43659, 43999, 43842, S2082, 43644, 43645, 43770, 43843, 43845, 43846, 43847<br>ICD-9 Procedure Code (any position) is any of: 43.89, 44.69, 44.31, 44.39, 44.95, 45.51, 45.91, 43.82, 44.38, 44.68<br>ICD-10 Procedure Code (any position) is any of: 0D160JB, 0D160KL, 0D160Z9, 0D164JL, 0D1687L, 0D168JB, 0D168Z9, 0D168ZL, 0D190ZA, 0D194J9, 0D194K9, 0D194ZA, 0D198K9, 0D198ZA, 0D1A0JB, 0D1A0ZB, 0D1A87A, 0D1A8JB, 0D1A8KA, 0D1A8KB, 0D1B4JB, 0D1B4KB, 0D1B8JB, 0D760DZ, 0DB63Z3, 0DB67ZZ, 0DB90ZZ, 0DM64ZZ, 0DN67ZZ, 0DQ64ZZ, 0DV60CZ, 0DV63CZ, 0D16079, 0D1607A, 0D1607L, 0D160ZL, 0D164J9, 0D164KA, 0D164KL, 0D1687A, 0D168J9, 0D168ZA, 0D190K9, 0D190KB,

© 2023 Lin KJ et al. *JAMA Network Open.*

	0D19479, 0D1947B, 0D194JA, 0D194KB, 0D1987A, 0D198ZB, 0D1A07A, 0D1A0KB, 0D1A47A, 0D1A4KA, 0D1A4ZB, 0D1A8ZA, 0D1A8ZB, 0D1B0JB, 0D763DZ, 0D764ZZ, 0DB60Z3, 0DB67Z3, 0DB68Z3, 0DQ68ZZ, 0DU64KZ, 0DU677Z, 0DU67KZ, 0DU687Z, 0DU68JZ, 0DU68KZ, 0DV63ZZ, 0DV64CZ, 0DV64DZ, 0D164Z9, 0D168JL, 0D168KA, 0D168KB, 0D168ZB, 0D190Z9, 0D1947A, 0D194KA, 0D194ZB, 0D1987B, 0D1A07B, 0D1A0JA, 0D1A4JA, 0D1A4KB, 0D763ZZ, 0D764DZ, 0DB63ZZ, 0DBB0ZZ, 0DF67ZZ, 0DF68ZZ, 0DN60ZZ, 0DQ60ZZ, 0DU60KZ, 0DU647Z, 0DU64JZ, 0DV60ZZ, 0DV64ZZ, 0D1607B, 0D160JL, 0D160K9, 0D160KA, 0D164JB, 0D1687B, 0D1907B, 0D190J9, 0D190JB, 0D190ZB, 0D194Z9, 0D198J9, 0D198KA, 0D1A0ZA, 0D1A47B, 0D1A4JB, 0D1A4ZA, 0D1A8JA, 0D1B47B, 0D1B4ZB, 0D1B8KB, 0D1B8ZB, 0D760ZZ, 0DB64Z3, 0DM60ZZ, 0DN63ZZ, 0DN64ZZ, 0DN68ZZ, 0DU607Z, 0DU60JZ, 0DV60DZ, 0DV63DZ, 0D160J9, 0D160JA, 0D160KB, 0D160ZA, 0D160ZB, 0D16479, 0D1647A, 0D1647B, 0D1647L, 0D164JA, 0D164K9, 0D164KB, 0D164ZA, 0D164ZB, 0D164ZL, 0D16879, 0D168JA, 0D168K9, 0D168KL, 0D19079, 0D1907A, 0D190JA, 0D190KA, 0D194JB, 0D19879, 0D198JA, 0D198JB, 0D198KB, 0D198Z9, 0D1A0KA, 0D1A87B, 0D1A8ZH, 0D1B07B, 0D1B0KB, 0D1B0ZB, 0D1B87B, 0D1B8ZH, 0DB60ZZ, 0DB80ZZ, 0DF60ZZ, 0DF63ZZ, 0DF64ZZ, 0DQ63ZZ, 0DQ67ZZ, 0DU67JZ, 0DV67ZZ, 0DV68ZZ<br>ICD-9 Diagnosis Code (any position) is any of: 539.89, 649.1, V85.36, V85.41, V85.42, 649.10, 649.11, 649.20, 649.23, V85.37, V85.44, 278.01, 278.03, 539, 649.13, 649.14, 649.2, 649.22, 649.24, V85.31, V85.34, V85.35, V85.43, 278.0, 278.00, 539.09, 539.8, 539.81, 649.12, 649.21, V85.38, V85.39, 539.0, 539.01, V85.30, V85.32, V85.33, V85.45<br>ICD-10 Diagnosis Code (any position) is any of: Z68.41, O99.210, O99.214, O99.844, Z68.35, Z68.38, Z68.43, Z68.45, O99.845, Z68.32, Z68.33, Z68.36, O99.212, O99.841, O99.842, Z68.34, Z68.37, Z68.42, O99.211, O99.213, O99.840, O99.843, Z68.30, Z68.31, Z68.39, Z68.44, O99.215, E66.01, E66.2<br>NDC Generic Name is any of: DEXFENFLURAMINE HCL, NALTREXONE HCL/BUPROPION HCL, PHENTERMINE HCL/TOPIRAMATE, LORCASERIN HCL, ORLISTAT, SIBUTRAMINE HCL M-HYDRATE, BENZPHETAMINE HCL, FENFLURAMINE HCL, DIETHYLPROPION HCL, PHENDIMETRAZINE TARTRATE, PHENTERMINE HCL
Cerebrovascular disease	ICD-9 Diagnosis Code (any position) is any of: 430, 431, 432, 432.0, 432.1, 432.9, 433, 433.0, 433.00, 433.01, 433.1, 433.10, 433.11, 433.2, 433.20, 433.21, 433.3, 433.30, 433.31, 433.8, 433.80, 433.81, 433.9, 433.90, 433.91, 434, 434.0, 434.00, 434.01, 434.1, 434.10, 434.11, 434.9, 434.90, 434.91, 435, 435.0, 435.1, 435.2, 435.3, 435.8, 435.9, 436, 437, 437.0, 437.1, 437.2, 437.3, 437.4, 437.5, 437.6, 437.7, 437.8, 437.9, 438, 438.0, 438.1, 438.10, 438.11,

© 2023 Lin KJ et al. *JAMA Network Open.*

	438.12, 438.13, 438.14, 438.19, 438.2, 438.20, 438.21, 438.22, 438.3, 438.30, 438.31, 438.32, 438.4, 438.40, 438.41, 438.42, 438.5, 438.50, 438.51, 438.52, 438.53, 438.6, 438.7, 438.8, 438.81, 438.82, 438.83, 438.84, 438.85, 438.89, 438.9<br>ICD-10 Diagnosis Code (any position) is any of: I60.0, I60.01, I60.10, I60.11, I60.21, I60.22, I60.3, I60.31, I60.5, I60.51, I60.52, I60.6, I60.8, I61, I61.4, I61.8, I62.1, I63.011, I63.012, I63.031, I63.112, I63.12, I63.132, I63.139, I63.19, I63.2, I63.21, I63.212, I63.213, I63.219, I63.22, I63.231, I63.232, I63.233, I63.239, I63.29, I63.30, I63.31, I63.311, I63.32, I63.322, I63.329, I63.33, I63.339, I63.34, I63.341, I63.342, I63.349, I63.39, I63.40, I63.411, I63.419, I63.422, I63.429, I63.431, I63.49, I63.50, I63.51, I63.52, I63.523, I63.529, I63.531, I63.533, I63.541, I63.542, I63.543, I63.8, I63.9, I65.01, I65.09, I65.2, I65.22, I65.23, I65.29, I65.9, I66, I66.0, I66.02, I66.09, I66.11, I66.12, I66.13, I66.19, I66.2, I66.21, I66.22, I66.8, I67.3, I67.6, I67.7, I67.8, I67.81, I67.84, I67.841, I67.848, I67.9, I68, I68.0, I68.8, I69.01, I69.02, I69.021, I69.022, I69.03, I69.031, I69.032, I69.04, I69.041, I69.042, I69.043, I69.051, I69.052, I69.053, I69.059, I69.06, I69.061, I69.063, I69.064, I69.065, I69.069, I69.09, I69.090, I69.092, I69.098, I69.12, I69.121, I69.128, I69.132, I69.133, I69.141, I69.143, I69.15, I69.151, I69.153, I69.154, I69.159, I69.16, I69.163, I69.165, I69.19, I69.190, I69.192, I69.193, I69.2, I69.21, I69.222, I69.228, I69.233, I69.234, I69.239, I69.241, I69.242, I69.244, I69.252, I69.253, I69.259, I69.26, I69.262, I69.263, I69.264, I69.265, I69.29, I69.291, I69.292, I69.298, I69.31, I69.32, I69.320, I69.323, I69.331, I69.332, I69.333, I69.334, I69.34, I69.344, I69.349, I69.35, I69.353, I69.362, I69.363, I69.364, I69.365, I69.391, I69.392, I69.393, I69.8, I69.81, I69.820, I69.822, I69.823, I69.831, I69.833, I69.834, I69.84, I69.841, I69.843, I69.844, I69.849, I69.851, I69.852, I69.853, I69.861, I69.862, I69.864, I69.869, I69.89, I69.890, I69.898, I69.9, I69.91, I69.921, I69.922, I69.93, I69.931, I69.932, I69.934, I69.939, I69.94, I69.941, I69.942, I69.943, I69.949, I69.951, I69.952, I69.953, I69.959, I69.963, I69.964, I69.969, I69.991, I69.992, G45.0, G45.9, I60, I60.00, I60.02, I60.1, I60.12, I60.2, I60.20, I60.30, I60.32, I60.4, I60.50, I60.7, I60.9, I61.0, I61.1, I61.2, I61.3, I61.5, I61.6, I61.9, I62, I62.0, I62.00, I62.01, I62.02, I62.03, I62.9, I63, I63.0, I63.00, I63.01, I63.013, I63.019, I63.02, I63.03, I63.032, I63.033, I63.039, I63.09, I63.1, I63.10, I63.11, I63.111, I63.113, I63.119, I63.13, I63.131, I63.133, I63.20, I63.211, I63.23, I63.3, I63.312, I63.319, I63.321, I63.331, I63.332, I63.4, I63.41, I63.412, I63.42, I63.421, I63.43, I63.432, I63.439, I63.44, I63.441, I63.442, I63.449, I63.5, I63.511, I63.512, I63.513, I63.519, I63.521, I63.522, I63.53, I63.532, I63.539, I63.54, I63.549, I63.59, I63.6, I65, I65.0, I65.02, I65.03, I65.1, I65.21, I65.8, I66.01, I66.03, I66.1, I66.23, I66.29, I66.3, I66.9, I67, I67.0, I67.1, I67.2, I67.4, I67.5, I67.82, I67.83, I67.89, I68.2, I69, I69.0, I69.00, I69.020, I69.023, I69.028,

© 2023 Lin KJ et al. *JAMA Network Open.*

	I69.033, I69.034, I69.039, I69.044, I69.049, I69.05, I69.054, I69.062, I69.091, I69.093, I69.1, I69.10, I69.11, I69.120, I69.122, I69.123, I69.13, I69.131, I69.134, I69.139, I69.14, I69.142, I69.144, I69.149, I69.152, I69.161, I69.162, I69.164, I69.169, I69.191, I69.198, I69.20, I69.22, I69.220, I69.221, I69.223, I69.23, I69.231, I69.232, I69.24, I69.243, I69.249, I69.25, I69.251, I69.254, I69.261, I69.269, I69.290, I69.293, I69.3, I69.30, I69.321, I69.322, I69.328, I69.33, I69.339, I69.341, I69.342, I69.343, I69.351, I69.352, I69.354, I69.359, I69.36, I69.361, I69.369, I69.39, I69.390, I69.398, I69.80, I69.82, I69.821, I69.828, I69.83, I69.832, I69.839, I69.842, I69.85, I69.854, I69.859, I69.86, I69.863, I69.865, I69.891, I69.892, I69.893, I69.90, I69.92, I69.920, I69.923, I69.928, I69.933, I69.944, I69.95, I69.954, I69.96, I69.961, I69.962, I69.965, I69.99, I69.990, I69.993, I69.998
Dementia	ICD-9 Diagnosis Code (any position) is any of: 290.0, 290.1, 290.11, 290.13, 290.20, 290.21, 290.42, 290.43, 294.11, 294.21, 294.8, 331.7, 290.12, 290.3, 290.40, 290.41, 294.0, 294.10, 294.20, 331.0, 331.11, 331.19, 331.2, 797<br>ICD-10 Diagnosis Code (any position) is any of: F01.50, F01.51, F03.91, G30.1, G30.8, G30.9, G31.01, G31.09, F02.80, F02.81, F03.90, G30.0, F01.5, F02.8, F03.9, G31.0
ED Visit	HCPCS Procedure Code (any position) is any of: 99281, 99282, 99283, 99284, 99285, 99288
Home health day	The occurrence of **Home Health Agency** claim
Hospitalizations	The occurrence of **Inpatient** claim

Clinical Risk Scores

Claims-based Frailty Index: https://dataverse.harvard.edu/dataverse/cfi

Combined Comorbidity Score: https://www.drugepi.org/dope/software#Combined1

© 2023 Lin KJ et al. *JAMA Network Open.*

eTable 1. Characteristics of the Overall Population of Medicare Fee-for-Service Beneficiaries With Atrial Fibrillation

Characteristics	Before Propensity Score Weighting				After Propensity Score Weighting			
	Apixaban (n=288,204) No. (%)	Rivaroxaban (n=158,101) No. (%)	Warfarin (n=147,791) No. (%)	SMD[a]	Apixaban (n=45,950) No. (%)	Rivaroxaban (n=45,320) No. (%)	Warfarin (n=45,281) No. (%)	SMD[a]
**Demographic characteristics**								
Age, mean (SD)	78.0 (7.5)	76.9 (7.1)	78.0 (7.3)	0.11	77.6 (7.3)	77.6 (7.3)	77.6 (7.3)	0.00
Female	155,308 (53.9)	80,476 (50.9)	75,384 (51.0)	0.04	23,563.0 (51.3)	23,502.2 (51.9)	23,525.7 (52.0)	0.01
Race				0.04				0.01
Black	11,996 (4.2)	6,457 (4.1)	6,333 (4.3)		1,886.0 (4.1)	1,932.6 (4.3)	1,897.2 (4.2)	
White	261,828 (90.8)	142,985 (90.4)	135,418 (91.6)		41,927.8 (91.2)	41,241.1 (91.0)	41,302.2 (91.2)	
Other/missing	14,380 (5.0)	8,659 (5.5)	6,040 (4.1)		2,135.8 (4.6)	2,146.3 (4.7)	2,081.0 (4.6)	
**Clinical characteristics, mean (SD)**								
CHA_2DS_2-VASc score	78.0 (7.5)	76.9 (7.1)	78.0 (7.3)	0.11	4.2 (1.5)	4.3 (1.5)	4.3 (1.5)	0.02
HAS-BLED score	4.3 (1.5)	4.1 (1.4)	4.4 (1.5)	0.12	2.3 (0.7)	2.3 (0.7)	2.3 (0.7)	0.01
Combined comorbidity score	3.3 (2.8)	3.0 (2.6)	3.5 (2.8)	0.08	3.2 (2.7)	3.3 (2.7)	3.3 (2.7)	0.02
Claims-based frailty index	0.2 (0.1)	0.2 (0.1)	0.2 (0.1)	0.13	0.2 (0.1)	0.2 (0.1)	0.2 (0.1)	0.02
**CV comorbidities**								
Acute myocardial infarction	19,616 (6.8)	8,668 (5.5)	11,512 (7.8)	0.06	3,019.4 (6.6)	3,048.9 (6.7)	3,086.6 (6.8)	0.01
Cardio-ablation	1,650 (0.6)	930 (0.6)	640 (0.4)	0.02	227.9 (0.5)	219.6 (0.5)	229.4 (0.5)	0.00
Cardioversion	20,372 (7.1)	9,790 (6.2)	4,788 (3.2)	0.12	2,280.0 (5.0)	2,129.8 (4.7)	2,198.8 (4.9)	0.01
Cerebrovascular disease	67,526 (23.4)	35,318 (22.3)	40,933 (27.7)	0.08	11,150.1 (24.3)	11,225.2 (24.8)	11,272.0 (24.9)	0.01
CHF (inpatient)	61,808 (21.4)	28,051 (17.7)	33,870 (22.9)	0.09	9,185.3 (20.0)	9,480.6 (20.9)	9,446.3 (20.9)	0.02
CHF (outpatient)	81,589 (28.3)	43,137 (27.3)	51,125 (34.6)	0.11	13,828.6 (30.1)	13,805.8 (30.5)	13,953.1 (30.8)	0.01
Coronary revascularization	5,471 (1.9)	2,596 (1.6)	3,390 (2.3)	0.03	9,185.3 (20.0)	9,480.6 (20.9)	9,446.3 (20.9)	0.00
Hypertension	251,051 (87.1)	132,648 (83.9)	122,830 (83.1)	0.08	38,682.8 (84.2)	38,339.5 (84.6)	38,241.5 (84.5)	0.01
Ischemic heart disease	131,046 (45.5)	67,600 (42.8)	69,888 (47.3)	0.06	20,793.6 (45.3)	20,495.7 (45.2)	20,724.5 (45.8)	0.01
Peripheral revascularization	131,046 (45.5)	67,600 (42.8)	69,888 (47.3)	0.06	8,155.8 (17.7)	8,187.1 (18.1)	8,156.4 (18.0)	0.01
Stroke (inpatient)	21,668 (7.5)	9,352 (5.9)	13,593 (9.2)	0.08	3,257.1 (7.1)	3,430.2 (7.6)	3,393.8 (7.5)	0.01
Stroke (outpatient)	61,010 (21.2)	29,667 (18.8)	33,289 (22.5)	0.06	9,436.9 (20.5)	9,482.4 (20.9)	9,474.2 (20.9)	0.01
Transient ischemic attack	18,419 (6.4)	9,126 (5.8)	9,724 (6.6)	0.02	2,796.0 (6.1)	2,832.6 (6.3)	2,866.2 (6.3)	0.01
Syncope	18,419 (6.4)	9,126 (5.8)	9,724 (6.6)	0.02	4,623.3 (10.1)	4,628.5 (10.2)	4,656.0 (10.3)	0.00
**Non-CV comorbidities**								
Acute renal failure	45,722 (15.9)	17,882 (11.3)	23,902 (16.2)	0.09	6,416.3 (14.0)	6,611.5 (14.6)	6,496.0 (14.3)	0.01
Alcohol abuse or dependence	6,992 (2.4)	3,438 (2.2)	2,908 (2.0)	0.02	1,006.5 (2.2)	1,027.7 (2.3)	985.7 (2.2)	0.00
Anemia	81,376 (28.2)	40,257 (25.5)	45,077 (30.5)	0.08	12,773.8 (27.8)	12,840.7 (28.3)	12,891.7 (28.5)	0.01
Chronic kidney disease	58,166 (20.2)	23,878 (15.1)	32,637 (22.1)	0.12	8,765.2 (19.1)	8,774.9 (19.4)	8,771.5 (19.4)	0.01
COPD	80,337 (27.9)	41,565 (26.3)	43,071 (29.1)	0.04	12,643.6 (27.5)	12,867.7 (28.4)	12,821.6 (28.3)	0.01

© 2023 Lin KJ et al. *JAMA Network Open.*

Characteristics	Before Propensity Score Weighting				After Propensity Score Weighting			
	Apixaban (n=288,204) No. (%)	Rivaroxaban (n=158,101) No. (%)	Warfarin (n=147,791) No. (%)	SMD[a]	Apixaban (n=45,950) No. (%)	Rivaroxaban (n=45,320) No. (%)	Warfarin (n=45,281) No. (%)	SMD[a]
Dementia	27,218 (9.4)	13,017 (8.2)	14,335 (9.7)	0.03	4,032.5 (8.8)	4,270.4 (9.4)	4,159.0 (9.2)	0.01
Diabetes	106,538 (37.0)	57,934 (36.6)	60,175 (40.7)	0.06	17,725.7 (38.6)	17,595.6 (38.8)	17,599.8 (38.9)	0.00
Endoscopy	5,666 (2.0)	2,771 (1.8)	3,752 (2.5)	0.04	934.3 (2.0)	960.5 (2.1)	964.5 (2.1)	0.00
Falls	4,121 (1.4)	1,769 (1.1)	1,722 (1.2)	0.02	559.1 (1.2)	570.8 (1.3)	559.4 (1.2)	0.00
Fractures	4,588 (1.6)	3,032 (1.9)	3,363 (2.3)	0.03	844.4 (1.8)	918.3 (2.0)	878.8 (1.9)	0.01
Frailty	66,588 (23.1)	31,325 (19.8)	37,681 (25.5)	0.09	10,196 (22.2)	10,644 (23.5)	10,496 (23.2)	0.02
GI bleeding (inpatient)	11,092 (3.8)	4,735 (3.0)	6,681 (4.5)	0.05	1,698.5 (3.7)	1,740.5 (3.8)	1,727.8 (3.8)	0.01
GI bleeding (outpatient)	16,908 (5.9)	8,842 (5.6)	10,054 (6.8)	0.03	2,829.7 (6.2)	2,808.9 (6.2)	2,800.5 (6.2)	0.00
Home oxygen use	14,629 (5.1)	6,643 (4.2)	7,031 (4.8)	0.03	2,076.9 (4.5)	2,179.8 (4.8)	2,163.0 (4.8)	0.01
Liver disease	13,564 (4.7)	6,148 (3.9)	5,238 (3.5)	0.04	1,844.0 (4.0)	1,838.8 (4.1)	1,815.9 (4.0)	0.01
Malignancy	52,586 (18.2)	28,615 (18.1)	27,274 (18.5)	0.01	8,408.2 (18.3)	8,363.7 (18.5)	8,347.3 (18.4)	0.00
Obesity	55,477 (19.2)	28,783 (18.2)	27,524 (18.6)	0.02	8,603.0 (18.7)	8,616.1 (19.0)	8,591.9 (19.0)	0.00
Peptic Ulcer	5,375 (1.9)	2,606 (1.6)	2,826 (1.9)	0.01	807.8 (1.8)	829.6 (1.8)	822.9 (1.8)	0.00
Smoking	5,375 (1.9)	2,606 (1.6)	2,826 (1.9)	0.01	8,119.9 (17.7)	8,170.5 (18.0)	8,295.3 (18.3)	0.01
**CV medications**								
ACE inhibitors	77,941 (27.0)	42,913 (27.1)	45,326 (30.7)	0.05	13,065.4 (28.4)	12,980.0 (28.6)	13,000.6 (28.7)	0.00
Angiotensin II receptor blockers	84,105 (29.2)	44,885 (28.4)	35,485 (24.0)	0.08	12,427.5 (27.0)	12,147.9 (26.8)	12,183.6 (26.9)	0.00
Antiarrhythmic agents	62,101 (21.5)	34,152 (21.6)	26,573 (18.0)	0.06	9,264.7 (20.2)	8,834.2 (19.5)	9,217.2 (20.4)	0.01
Antiplatelet agent	46,531 (16.1)	23,471 (14.8)	21,915 (14.8)	0.02	7,078.6 (15.4)	6,931.0 (15.3)	7,079.1 (15.6)	0.01
Beta-blockers	198,946 (69.0)	105,308 (66.6)	101,039 (68.4)	0.04	31,263.3 (68.0)	30,792.7 (67.9)	30,909.9 (68.3)	0.00
Calcium channel blockers	6,481 (2.2)	3,309 (2.1)	3,361 (2.3)	0.01	996.5 (2.2)	1,009.6 (2.2)	1,008.7 (2.2)	0.00
Diuretics	157,074 (54.5)	83,648 (52.9)	86,141 (58.3)	0.07	25,567.7 (55.6)	25,300.5 (55.8)	25,387.8 (56.1)	0.01
Fibrates	11,877 (4.1)	6,807 (4.3)	6,886 (4.7)	0.02	2,060.8 (4.5)	2,051.4 (4.5)	2,048.1 (4.5)	0.00
Statins	182,165 (63.2)	97,073 (61.4)	91,471 (61.9)	0.03	28,711.7 (62.5)	28,211.0 (62.2)	28,247.5 (62.4)	0.00
Nitrates	34,663 (12.0)	17,502 (11.1)	20,742 (14.0)	0.06	5,767.1 (12.6)	5,670.8 (12.5)	5,813.7 (12.8)	0.01
**Non-CV medications**								
Anticonvulsants	52,556 (18.2)	26,003 (16.4)	25,134 (17.0)	0.03	7,966.6 (17.3)	7,978.3 (17.6)	8,025.7 (17.7)	0.01
Antidepressants - SSRI/SNRI	64,245 (22.3)	33,333 (21.1)	31,017 (21.0)	0.02	9,833.5 (21.4)	9,879.6 (21.8)	9,886.2 (21.8)	0.01
Antidepressants - Tricyclics	8,215 (2.9)	4,421 (2.8)	4,427 (3.0)	0.01	1,326.3 (2.9)	1,329.2 (2.9)	1,333.0 (2.9)	0.00
Antidepressants - Other	27,820 (9.7)	13,595 (8.6)	12,649 (8.6)	0.03	4,061.8 (8.8)	4,125.3 (9.1)	4,081.3 (9.0)	0.01
Antipsychotic agents	9,970 (3.5)	5,287 (3.3)	4,977 (3.4)	0.00	1,516.9 (3.3)	1,597.0 (3.5)	1,581.0 (3.5)	0.01
Anxiolytics - Benzodiazepines	52,940 (18.4)	28,819 (18.2)	24,276 (16.4)	0.03	8,190.1 (17.8)	8,074.3 (17.8)	8,176.4 (18.1)	0.00
Anxiolytics - Non-benzodiazepines	4,618 (1.6)	2,112 (1.3)	1,742 (1.2)	0.02	612.0 (1.3)	618.3 (1.4)	614.4 (1.4)	0.00
Bronchodilators	59,781 (20.7)	31,417 (19.9)	28,805 (19.5)	0.02	9,159.9 (19.9)	9,324.5 (20.6)	9,251.7 (20.4)	0.01

© 2023 Lin KJ et al. *JAMA Network Open.*

Characteristics	Before Propensity Score Weighting				After Propensity Score Weighting			
	Apixaban (n=288,204) No. (%)	Rivaroxaban (n=158,101) No. (%)	Warfarin (n=147,791) No. (%)	SMD[a]	Apixaban (n=45,950) No. (%)	Rivaroxaban (n=45,320) No. (%)	Warfarin (n=45,281) No. (%)	SMD[a]
Corticosteroids, inhaled	66,661 (23.1)	36,148 (22.9)	30,279 (20.5)	0.04	10,155.1 (22.1)	10,168.5 (22.4)	10,152.0 (22.4)	0.01
Corticosteroids, oral	100,783 (35.0)	52,829 (33.4)	45,813 (31.0)	0.06	15,040.9 (32.7)	14,961.6 (33.0)	14,969.8 (33.1)	0.00
Dementia drugs	16,031 (5.6)	8,155 (5.2)	7,629 (5.2)	0.01	2,394.1 (5.2)	2,474.0 (5.5)	2,445.7 (5.4)	0.01
Diabetes agents - Insulin	22,383 (7.8)	11,027 (7.0)	13,429 (9.1)	0.05	3,739.9 (8.1)	3,830.8 (8.5)	3,788.2 (8.4)	0.01
Diabetes agents - Metformin	48,655 (16.9)	26,932 (17.0)	25,257 (17.1)	0.00	7,942.2 (17.3)	7,896.6 (17.4)	7,866.2 (17.4)	0.00
Diabetes agents - Other	16,269 (5.6)	8,555 (5.4)	7,762 (5.3)	0.01	2,516.9 (5.5)	2,537.3 (5.6)	2,541.2 (5.6)	0.00
Diabetes agents - Sulfonylurea	26,232 (9.1)	14,415 (9.1)	16,789 (11.4)	0.05	4,619.1 (10.1)	4,576.2 (10.1)	4,607.9 (10.2)	0.00
Estrogen	9,406 (3.3)	5,266 (3.3)	3,705 (2.5)	0.03	1,335.1 (2.9)	1,326.7 (2.9)	1,339.4 (3.0)	0.00
GI - H2 blockers	25,755 (8.9)	12,441 (7.9)	11,947 (8.1)	0.03	3,744.5 (8.1)	3,783.1 (8.3)	3,756.8 (8.3)	0.00
GI - Proton-pump inhibitors	96,712 (33.6)	50,777 (32.1)	47,746 (32.3)	0.02	15,012.4 (32.7)	14,921.7 (32.9)	14,992.8 (33.1)	0.01
GI - Antacids	6,317 (2.2)	3,154 (2.0)	2,806 (1.9)	0.01	922.9 (2.0)	924.8 (2.0)	932.9 (2.1)	0.00
Hypnotics	23,752 (8.2)	14,072 (8.9)	11,567 (7.8)	0.03	3,751.7 (8.2)	3,726.6 (8.2)	3,776.0 (8.3)	0.00
NSAIDs	49,699 (17.2)	28,975 (18.3)	21,359 (14.5)	0.07	7,489.8 (16.3)	7,449.7 (16.4)	7,455.7 (16.5)	0.00
Opioids	106,530 (37.0)	60,481 (38.3)	58,601 (39.7)	0.04	17,712.6 (38.5)	17,649.3 (38.9)	17,768.1 (39.2)	0.01
Parkinsonism drugs	12,099 (4.2)	6,233 (3.9)	5,997 (4.1)	0.01	1,888.7 (4.1)	1,889.4 (4.2)	1,911.6 (4.2)	0.00
Thyroid hormone replacement	66,890 (23.2)	34,117 (21.6)	32,864 (22.2)	0.03	10,298.0 (22.4)	10,138.0 (22.4)	10,215.3 (22.6)	0.00
**Healthcare utilization, mean (SD)**								
Hospitalizations	0.9 (1.1)	0.8 (1.0)	0.9 (1.1)	0.08	0.8 (1.1)	0.9 (1.1)	0.8 (1.0)	0.02
Skilled nursing facility	0.4 (1.2)	0.3 (1.1)	0.5 (1.4)	0.09	0.4 (1.2)	0.4 (1.3)	0.4 (1.2)	0.02
ED visits	0.7 (1.3)	0.7 (1.2)	0.7 (1.2)	0.03	0.7 (1.3)	0.7 (1.3)	0.7 (1.2)	0.01
Home health day	0.3 (0.9)	0.3 (0.9)	0.3 (0.9)	0.03	0.3 (0.9)	0.3 (0.9)	0.3 (0.9)	0.01
**Healthcare costs, $, mean (SD)**								
Inpatient	10,170.9 (17,808.7)	8,400.6 (15,866.4)	11,437.5 (19,960.1)	0.11	9,339.3 (17,351.1)	9,717.2 (17,435.0)	10,665.8 (18,931.8)	0.05
Outpatient	9,513.3 (12,026.0)	8,748.7 (11,127.0)	8,710.2 (10,404.7)	0.05	8,941.6 (11,196.9)	9,030.7 (11,325.6)	8,943.8 (10,841.4)	0.01
Pharmacy	4,204.7 (11,487.3)	3,319.5 (9,741.3)	2,284.7 (7,103.6)	0.14	3,290.9 (9,521.3)	3,351.9 (9,882.9)	3,064.7 (8,692.2)	0.02
Skilled nursing facility	2,890.1 (10,144.4)	2,371.8 (9,374.0)	3,679.2 (11,492.1)	0.08	2,781.9 (9,986.4)	3,104.2 (10,673.9)	2,966.9 (10,382.6)	0.02
Home health	900.2 (2,674.1)	755.3 (2,482.0)	837.4 (2,550.0)	0.04	810.6 (2,540.3)	852.6 (2,634.0)	827.9 (2,550.4)	0.01
Durable medical equipment	400.7 (2,074.8)	379.2 (1,858.5)	461.4 (2,553.9)	0.03	411.0 (2,104.1)	415.9 (1,965.1)	435.7 (2,642.0)	0.01
**Co-payment, $, mean (SD)**								

© 2023 Lin KJ et al. *JAMA Network Open.*

Characteristics	Before Propensity Score Weighting				After Propensity Score Weighting			
	Apixaban (n=288,204) No. (%)	Rivaroxaban (n=158,101) No. (%)	Warfarin (n=147,791) No. (%)	SMD[a]	Apixaban (n=45,950) No. (%)	Rivaroxaban (n=45,320) No. (%)	Warfarin (n=45,281) No. (%)	SMD[a]
Inpatient	2,471.1 (2,525.0)	2,200.8 (2,348.2)	2,362.3 (2,443.0)	0.07	2,284.4 (2,427.0)	2,352.7 (2,489.8)	2,338.2 (2,363.6)	0.02
Outpatient	1,767.8 (2,303.4)	1,646.5 (2,105.9)	1,611.1 (1,987.1)	0.05	1,671.5 (2,142.0)	1,686.6 (2,169.0)	1,670.7 (2,066.8)	0.00
Pharmacy	649.1 (895.6)	535.0 (791.4)	403.9 (685.7)	0.21	522.4 (797.5)	534.1 (801.6)	523.4 (792.5)	0.01
Skilled nursing facility	472.4 (2,081.2)	400.5 (1,931.1)	616.2 (2,337.5)	0.07	461.9 (2,059.4)	527.4 (2,213.8)	4867.0 (2,085.04)	0.02
Durable medical equipment	88.5 (428.7)	83.8 (384.6)	101.5 (524.3)	0.03	90.7 (434.3)	91.8 (406.7)	95.8 (542.8)	0.01
**Year of the cohort entry**				0.68				0.04
2013	4,829 (1.7)	20,197 (12.8)	27,940 (18.9)		3,356.7 (7.3)	3,233.2 (7.1)	3,591.3 (7.9)	
2014	20,132 (7.0)	26,742 (16.9)	32,831 (22.2)		8,012.2 (17.4)	7,748.0 (17.1)	8,404.4 (18.6)	
2015	35,924 (12.5)	22,798 (14.4)	27,903 (18.9)		8,356.2 (18.2)	8,336.7 (18.4)	8,222.9 (18.2)	
2016	46,337 (16.1)	21,090 (13.3)	21,105 (14.3)		7,636.8 (16.6)	7,620.0 (16.8)	7,449.5 (16.5)	
2017	47,312 (16.4)	26,457 (16.7)	16,669 (11.3)		7,411.5 (16.1)	7,389.3 (16.3)	7,142.7 (15.8)	
2018	64,995 (22.6)	22,137 (14.0)	12,067 (8.2)		6,148.0 (13.4)	6,098.9 (13.5)	5,788.7 (12.8)	
2019	68,675 (23.8)	18,680 (11.8)	9,276 (6.3)		5,028.2 (10.9)	4,893.9 (10.8)	4,681.0 (10.3)	
**Geographic region**				0.17				0.01
Northeast	54,261 (18.8)	29,731 (18.8)	29,943 (20.3)		8,939.7 (19.5)	8,885.0 (19.6)	8,736.2 (19.3)	
Midwest	65,279 (22.7)	36,584 (23.1)	45,780 (31.0)		11,975.7 (26.1)	11,885.7 (26.2)	11,907.1 (26.3)	
South	119,955 (41.6)	61,399 (38.8)	45,951 (31.1)		16,595.0 (36.1)	16,269.3 (35.9)	16,513.8 (36.5)	
West	48,485 (16.8)	30,175 (19.1)	25,951 (17.6)		8,393.6 (18.3)	8,233.6 (18.2)	8,070.6 (17.8)	
Other/missing	224 (0.1)	212 (0.1)	166 (0.1)		45.6 (0.1)	46.4 (0.1)	52.8 (0.1)	

Abbreviations: ACE, angiotensin converting enzyme; CHF, congestive heart failure; COPD, chronic obstructive pulmonary disease; CV, cardiovascular; ED, emergency department; GI, gastrointestinal; NSAID, non-steroidal anti-inflammatory drug; SD, standard deviation; SMD, standardized mean difference; SNRI, serotonin-norepinephrine reuptake inhibitor; SSRI, selective serotonin reuptake inhibitor.
[a]Presented are the average of the absolute SMDs between all pair-wise contrast among the apixaban, rivaroxaban, and warfarin users.

© 2023 Lin KJ et al. *JAMA Network Open.*

eTable 2. Characteristics of the Non-Frail Population of Medicare Fee-for-Service Beneficiaries With Atrial Fibrillation

Characteristics	Before Propensity Score Weighting				After Propensity Score Weighting			
	**Apixaban (n=55,429) No. (%)**	**Rivaroxaban (n=36,001) No. (%)**	**Warfarin (n=25,949) No. (%)**	**SMD[a]**	**Apixaban (n=8,612) No. (%)**	**Rivaroxaban (n=8,381) No. (%)**	**Warfarin (n=8,441) No. (%)**	**SMD[a]**
**Demographic characteristics**								
Age, mean (SD)	76.1 (6.6)	75.4 (6.3)	77.0 (6.8)	0.16	76.3 (6.6)	76.2 (6.5)	76.3 (6.6)	0.01
Female	22,346 (40.3)	13,505 (37.5)	10,112 (39.0)	0.04	3,322.7 (38.6)	3,257.5 (38.9)	3,311.4 (39.2)	0.01
Race				0.05				0.01
Black	1,198 (2.2)	779 (2.2)	597 (2.3)		190.9 (2.2)	186.9 (2.2)	186.5 (2.2)	
White	51,512 (92.9)	33,303 (92.5)	24,330 (93.8)		8,036.1 (93.3)	7,814.3 (93.2)	7,888.0 (93.5)	
Other/missing	2,719 (4.9)	1,919 (5.3)	1,022 (3.9)		384.8 (4.5)	380.0 (4.5)	366.3 (4.3)	
**Clinical characteristics, mean (SD)**								
CHA_2DS_2-VASc score	3.2 (1.0)	3.1 (1.0)	3.2 (1.0)	0.08	3.2 (1.0)	3.2 (1.0)	3.2 (1.0)	0.00
HAS-BLED score	1.9 (0.6)	1.9 (0.6)	1.9 (0.6)	0.04	1.9 (0.6)	1.9 (0.6)	1.9 (0.6)	0.01
Combined comorbidity score	1.5 (1.6)	1.4 (1.6)	1.5 (1.6)	0.07	1.5 (1.6)	1.5 (1.6)	1.5 (1.6)	0.01
Claims-based frailty index	0.1 (0.0)	0.1 (0.0)	0.1 (0.0)	0.05	0.1 (0.0)	0.1 (0.0)	0.1 (0.0)	0.01
**CV comorbidities**								0.01
Acute myocardial infarction	795 (1.4)	419 (1.2)	490 (1.9)	0.04	130.5 (1.5)	128.3 (1.5)	134.4 (1.6)	0.00
Cardio-ablation	288 (0.5)	188 (0.5)	91 (0.4)	0.02	38.2 (0.4)	34.0 (0.4)	38.5 (0.5)	0.02
Cardioversion	4,026 (7.3)	2,379 (6.6)	736 (2.8)	0.14	390.5 (4.5)	357.1 (4.3)	381.4 (4.5)	0.01
Cerebrovascular disease	4,192 (7.6)	2,949 (8.2)	2,531 (9.8)	0.05	757.5 (8.8)	727.6 (8.7)	761.9 (9.0)	0.00
CHF (inpatient)	3,099 (5.6)	1,714 (4.8)	1,355 (5.2)	0.03	417.1 (4.8)	436.6 (5.2)	446.9 (5.3)	0.01
CHF (outpatient)	7,091 (12.8)	4,840 (13.4)	4,308 (16.6)	0.07	1,257.8 (14.6)	1,214.8 (14.5)	1,271.1 (15.1)	0.00
Coronary revascularization	324 (0.6)	153 (0.4)	180 (0.7)	0.02	47.8 (0.6)	49.7 (0.6)	49.5 (0.6)	0.00
Hypertension	39,373 (71.0)	24,240 (67.3)	15,771 (60.8)	0.15	5,635.6 (65.4)	5,503.1 (65.7)	5,440.7 (64.5)	0.01
Ischemic heart disease	9,521 (17.2)	6,099 (16.9)	4,794 (18.5)	0.03	1,540.8 (17.9)	1,493.5 (17.8)	1,542.6 (18.3)	0.00
Peripheral revascularization	3,831 (6.9)	2,390 (6.6)	1,844 (7.1)	0.01	607.0 (7.0)	595.2 (7.1)	600.2 (7.1)	0.01
Stroke (inpatient)	594 (1.1)	355 (1.0)	322 (1.2)	0.02	92.3 (1.1)	97.3 (1.2)	96.4 (1.1)	0.01
Stroke (outpatient)	4,088 (7.4)	2,512 (7.0)	1,920 (7.4)	0.01	630.9 (7.3)	611.9 (7.3)	619.2 (7.3)	0.01
Transient ischemic attack	1,445 (2.6)	877 (2.4)	597 (2.3)	0.01	207.9 (2.4)	204.7 (2.4)	206.0 (2.4)	0.01
Syncope	3,341 (6.0)	1,981 (5.5)	1,157 (4.5)	0.05	436.5 (5.1)	425.0 (5.1)	442.9 (5.2)	0.00
**Non-CV comorbidities**								
Acute renal failure	1,309 (2.4)	621 (1.7)	555 (2.1)	0.03	174.3 (2.0)	177.0 (2.1)	172.2 (2.0)	0.00
Alcohol abuse or dependence	410 (0.7)	251 (0.7)	142 (0.5)	0.02	53.4 (0.6)	56.2 (0.7)	52.0 (0.6)	0.00
Anemia	6,048 (10.9)	3,706 (10.3)	2,923 (11.3)	0.02	943.1 (11.0)	924.5 (11.0)	947.7 (11.2)	0.01
Chronic kidney disease	2,817 (5.1)	1,467 (4.1)	1,382 (5.3)	0.04	426.3 (5.0)	426.7 (5.1)	424.5 (5.0)	0.00
COPD	3,623 (6.5)	2,299 (6.4)	1,714 (6.6)	0.01	577.5 (6.7)	576.4 (6.9)	591.6 (7.0)	0.01

© 2023 Lin KJ et al. *JAMA Network Open.*

Characteristics	Before Propensity Score Weighting				After Propensity Score Weighting			
	Apixaban (n=55,429) No. (%)	Rivaroxaban (n=36,001) No. (%)	Warfarin (n=25,949) No. (%)	SMD[a]	Apixaban (n=8,612) No. (%)	Rivaroxaban (n=8,381) No. (%)	Warfarin (n=8,441) No. (%)	SMD[a]
Dementia	37 (0.1)	20 (0.1)	20 (0.1)	0.01	5.4 (0.1)	6.4 (0.1)	6.4 (0.1)	0.00
Diabetes	8,117 (14.6)	5,624 (15.6)	4,277 (16.5)	0.03	1,400.3 (16.3)	1,369.8 (16.3)	1,359.5 (16.1)	0.00
Endoscopy	266 (0.5)	139 (0.4)	147 (0.6)	0.02	45.0 (0.5)	43.5 (0.5)	46.3 (0.5)	0.00
Falls	109 (0.2)	46 (0.1)	41 (0.2)	0.01	13.1 (0.2)	12.9 (0.2)	13.0 (0.2)	0.00
Fractures	89 (0.2)	60 (0.2)	49 (0.2)	0.01	14.8 (0.2)	16.4 (0.2)	15.1 (0.2)	0.00
Frailty	0 (0)	0 (0)	0 (0)	0.00	0 (0)	0 (0)	0 (0)	0.00
GI bleeding (inpatient)	534 (1.0)	258 (0.7)	283 (1.1)	0.03	77.6 (0.9)	81.1 (1.0)	78.4 (0.9)	0.00
GI bleeding (outpatient)	2,302 (4.2)	1,468 (4.1)	1,218 (4.7)	0.02	373.1 (4.3)	375.3 (4.5)	358.9 (4.3)	0.01
Home oxygen use	96 (0.2)	52 (0.1)	41 (0.2)	0.01	12.7 (0.1)	15.2 (0.2)	12.7 (0.2)	0.01
Liver disease	1,526 (2.8)	814 (2.3)	529 (2.0)	0.03	198.5 (2.3)	197.1 (2.4)	182.5 (2.2)	0.01
Malignancy	8,920 (16.1)	6,134 (17.0)	4,456 (17.2)	0.02	1,460.7 (17.0)	1,449.4 (17.3)	1,486.4 (17.6)	0.01
Obesity	5,406 (9.8)	3,322 (9.2)	2,122 (8.2)	0.04	773.1 (9.0)	759.3 (9.1)	748.0 (8.9)	0.00
Peptic Ulcer	358 (0.6)	206 (0.6)	149 (0.6)	0.01	48.6 (0.6)	50.4 (0.6)	49.6 (0.6)	0.00
Smoking	3,108 (5.6)	2,786 (7.7)	2,108 (8.1)	0.07	637.2 (7.4)	631.5 (7.5)	680.0 (8.1)	0.02
**CV medications**								
ACE inhibitors	12,167 (22.0)	7,761 (21.6)	6,412 (24.7)	0.05	1,983.5 (23.0)	1,943.2 (23.2)	1,950.7 (23.1)	0.00
Angiotensin II receptor blockers	13,800 (24.9)	8,972 (24.9)	5,251 (20.2)	0.08	2,001.2 (23.2)	1,953.5 (23.3)	1,981.4 (23.5)	0.00
Antiarrhythmic agents	11,772 (21.2)	7,686 (21.3)	4,369 (16.8)	0.08	1,667.3 (19.4)	1,596.4 (19.0)	1,693.6 (20.1)	0.02
Antiplatelet agent	3,731 (6.7)	2,276 (6.3)	1,525 (5.9)	0.02	560.2 (6.5)	530.1 (6.3)	567.6 (6.7)	0.01
Beta-blockers	34,918 (63.0)	22,067 (61.3)	15,878 (61.2)	0.03	5,316.8 (61.7)	5,176.5 (61.8)	5,239.0 (62.1)	0.00
Calcium channel blockers	770 (1.4)	537 (1.5)	372 (1.4)	0.01	127.0 (1.5)	126.2 (1.5)	131.0 (1.6)	0.00
Diuretics	22,339 (40.3)	14,359 (39.9)	11,418 (44.0)	0.06	3,651.6 (42.4)	3,570.1 (42.6)	3,629.2 (43.0)	0.01
Fibrates	1,610 (2.9)	1,101 (3.1)	868 (3.3)	0.02	287.4 (3.3)	290.7 (3.5)	293.6 (3.5)	0.01
Statins	30,734 (55.4)	19,683 (54.7)	14,051 (54.1)	0.02	4,766.9 (55.4)	4,658.8 (55.6)	4,677.2 (55.4)	0.00
Nitrates	2,450 (4.4)	1,474 (4.1)	1,482 (5.7)	0.05	425.1 (4.9)	420.4 (5.0)	438.3 (5.2)	0.01
**Non-CV medications**								
Anticonvulsants	3,365 (6.1)	2,038 (5.7)	1,482 (5.7)	0.01	521.6 (6.1)	509.6 (6.1)	526.2 (6.2)	0.00
Antidepressants - SSRI/SNRI	4,718 (8.5)	3,120 (8.7)	2,079 (8.0)	0.02	745.8 (8.7)	725.8 (8.7)	742.8 (8.8)	0.00
Antidepressants - Tricyclics	703 (1.3)	435 (1.2)	331 (1.3)	0.00	114.5 (1.3)	110.7 (1.3)	114.4 (1.4)	0.00
Antidepressants - Other	1,913 (3.5)	1,185 (3.3)	754 (2.9)	0.02	273.1 (3.2)	276.3 (3.3)	273.4 (3.2)	0.00
Antipsychotic agents	204 (0.4)	157 (0.4)	75 (0.3)	0.02	29.7 (0.3)	30.2 (0.4)	30.6 (0.4)	0.00
Anxiolytics - Benzodiazepines	5,702 (10.3)	3,699 (10.3)	2,285 (8.8)	0.03	851.1 (9.9)	821.4 (9.8)	842.9 (10.0)	0.00
Anxiolytics - Non-benzodiazepines	232 (0.4)	155 (0.4)	79 (0.3)	0.01	28.7 (0.3)	31.7 (0.4)	30.3 (0.4)	0.01
Bronchodilators	4,697 (8.5)	2,832 (7.9)	1,822 (7.0)	0.04	694.7 (8.1)	684.1 (8.2)	700.7 (8.3)	0.01

© 2023 Lin KJ et al. *JAMA Network Open.*

Characteristics	Before Propensity Score Weighting				After Propensity Score Weighting			
	Apixaban (n=55,429) No. (%)	Rivaroxaban (n=36,001) No. (%)	Warfarin (n=25,949) No. (%)	SMD[a]	Apixaban (n=8,612) No. (%)	Rivaroxaban (n=8,381) No. (%)	Warfarin (n=8,441) No. (%)	SMD[a]
Corticosteroids, inhaled	8,268 (14.9)	5,421 (15.1)	3,240 (12.5)	0.05	1,237.5 (14.4)	1,212.8 (14.5)	1,264.5 (15.0)	0.01
Corticosteroids, oral	13,614 (24.6)	8,332 (23.1)	5,280 (20.3)	0.07	1,934.0 (22.5)	1,894.2 (22.6)	1,913.2 (22.7)	0.00
Dementia drugs	463 (0.8)	323 (0.9)	288 (1.1)	0.02	85.2 (1.0)	85.1 (1.0)	86.6 (1.0)	0.00
Diabetes agents - Insulin	552 (1.0)	333 (0.9)	291 (1.1)	0.01	99.7 (1.2)	102.8 (1.2)	100.2 (1.2)	0.00
Diabetes agents - Metformin	4,222 (7.6)	2,770 (7.7)	2,008 (7.7)	0.00	686.2 (8.0)	688.3 (8.2)	662.2 (7.8)	0.01
Diabetes agents - Other	920 (1.7)	639 (1.8)	399 (1.5)	0.01	155.3 (1.8)	157.3 (1.9)	154.1 (1.8)	0.00
Diabetes agents - Sulfonylurea	1,435 (2.6)	1,008 (2.8)	913 (3.5)	0.04	280.4 (3.3)	269.2 (3.2)	263.6 (3.1)	0.01
Estrogen	1,809 (3.3)	1,108 (3.1)	615 (2.4)	0.04	241.2 (2.8)	243.8 (2.9)	261.6 (3.1)	0.01
GI - H2 blockers	2,643 (4.8)	1,492 (4.1)	1,034 (4.0)	0.03	370.3 (4.3)	362.3 (4.3)	363.5 (4.3)	0.00
GI - Proton-pump inhibitors	12,206 (22.0)	7,677 (21.3)	5,140 (19.8)	0.04	1,856.0 (21.6)	1,809.4 (21.6)	1,836.3 (21.8)	0.00
GI - Antacids	575 (1.0)	368 (1.0)	216 (0.8)	0.01	84.9 (1.0)	78.7 (0.9)	83.4 (1.0)	0.00
Hypnotics	3,067 (5.5)	2,148 (6.0)	1,187 (4.6)	0.04	441.4 (5.1)	442.1 (5.3)	469.3 (5.6)	0.01
NSAIDs	7,025 (12.7)	4,708 (13.1)	2,496 (9.6)	0.07	979.4 (11.4)	963.2 (11.5)	980.7 (11.6)	0.01
Opioids	11,616 (21.0)	8,039 (22.3)	5,794 (22.3)	0.02	1,915.5 (22.2)	1,873.4 (22.4)	1,940.5 (23.0)	0.01
Parkinsonism drugs	444 (0.8)	267 (0.7)	179 (0.7)	0.01	68.2 (0.8)	65.7 (0.8)	67.4 (0.8)	0.00
Thyroid hormone replacement	9,056 (16.3)	5,481 (15.2)	4,175 (16.1)	0.02	1,395.9 (16.2)	1,366.4 (16.3)	1,394.9 (16.5)	0.01
**Healthcare utilization, mean (SD)**								
Hospitalizations	0.3 (0.5)	0.3 (0.5)	0.3 (0.5)	0.04	0.2 (0.5)	0.3 (0.5)	0.3 (0.5)	0.03
Skilled nursing facility	0.0 (0.1)	0.0 (0.1)	0.0 (0.1)	0.02	0.0 (0.1)	0.0 (0.1)	0.0 (0.1)	0.00
ED visits	0.3 (0.7)	0.3 (0.7)	0.3 (0.6)	0.08	0.3 (0.6)	0.3 (0.6)	0.3 (0.6)	0.02
Home health day	0.0 (0.2)	0.0 (0.2)	0.0 (0.2)	0.01	0.0 (0.2)	0.0 (0.2)	0.0 (0.2)	0.01
**Healthcare costs, $, mean (SD)**								
Inpatient	2,690.9 (7,093.2)	2,287.1 (6,277.0)	3,007.4 (8,382.2)	0.07	2,348.7 (6,628.6)	2,421.6 (6,647.3)	3,115.8 (8,325.1)	0.07
Outpatient	6,275.8 (9,310.3)	5,901.6 (8,796.7)	5,248.5 (8,232.9)	0.08	5,727.8 (8,399.7)	5,774.9 (8,768.6)	5,789.6 (9,167.5)	0.00
Pharmacy	2,637.5 (9,474.3)	2,150.5 (7,814.4)	1,189.9 (5,652.7)	0.13	1,982.0 (7,799.6)	2,003.7 (7,460.8)	1,939.5 (7,612.1)	0.01
Skilled nursing facility	2.0 (105.3)	1.5 (90.6)	1.9 (100.2)	0.00	27.4 (638.9)	29.0 (661.1)	34.1 (859.4)	0.01
Home health	48.4 (478.8)	40.1 (383.6)	41.6 (428.2)	0.01	40.4 (429.8)	41.7 (390.7)	47.0 (479.0)	0.01
Durable medical equipment	91.2 (766.4)	81.8 (511.3)	95.6 (1,043.8)	0.01	92.3 (721.9)	84.0 (519.3)	103.3 (1,259.9)	0.01

© 2023 Lin KJ et al. *JAMA Network Open.*

Characteristics	Before Propensity Score Weighting				After Propensity Score Weighting			
	Apixaban (n=55,429) No. (%)	Rivaroxaban (n=36,001) No. (%)	Warfarin (n=25,949) No. (%)	SMD[a]	Apixaban (n=8,612) No. (%)	Rivaroxaban (n=8,381) No. (%)	Warfarin (n=8,441) No. (%)	SMD[a]
**Co-payment, $, mean (SD)**								
Inpatient	1,279.2 (1,536.5)	1,183.4 (1,391.5)	1,120.0 (1,449.8)	0.07	1,149.3 (1,395.4)	1,169.4 (1,410.8)	1,221.8 (1,547.0)	0.03
Outpatient	1,243.1 (1,795.8)	1,180.2 (1,607.7)	1,067.3 (1,551.4)	0.07	1,149.1 (1,619.9)	1,161.0 (1,627.6)	1,162.5 (1,669.4)	0.01
Pharmacy	548.0 (742.9)	463.3 (671.1)	290.7 (547.5)	0.27	419.3 (655.6)	432.2 (652.0)	434.1 (691.6)	0.01
Skilled nursing facility	2.0 (105.3)	1.5 (90.6)	1.9 (100.2)	0.00	1.9 (99.5)	1.9 (103.6)	2.1 (113.8)	0.00
Durable medical equipment	20.7 (160.0)	18.7 (109.0)	21.6 (215.5)	0.01	21.1 (151.2)	19.3 (111.0)	23.2 (259.3)	0.01
**Year of the cohort entry**				0.60				0.10
2013	1,181 (2.1)	4,408 (12.2)	4,617 (17.8)		749.2 (8.7)	675.5 (8.1)	919.3 (10.9)	
2014	4,370 (7.9)	6,003 (16.7)	5,714 (22.0)		1,576.1 (18.3)	1,487.6 (17.7)	1,799.0 (21.3)	
2015	7,060 (12.7)	5,037 (14.0)	4,521 (17.4)		1,456.1 (16.9)	1,446.7 (17.3)	1,433.2 (17.0)	
2016	8,958 (16.2)	4,882 (13.6)	3,726 (14.4)		1,389.6 (16.1)	1,371.8 (16.4)	1,308.1 (15.5)	
2017	9,292 (16.8)	5,949 (16.5)	3,109 (12.0)		1,339.4 (15.6)	1,326.1 (15.8)	1,216.8 (14.4)	
2018	11,930 (21.5)	5,197 (14.4)	2,351 (9.1)		1,134.4 (13.2)	1,120.9 (13.4)	974.7 (11.5)	
2019	12,638 (22.8)	4,525 (12.6)	1,911 (7.4)		967.1 (11.2)	952.6 (11.4)	789.7 (9.4)	
**Geographic region**				0.17				0.01
Northeast	10,719 (19.3)	7,188 (20.0)	5,087 (19.6)		1,712.6 (19.9)	1,677.2 (20.0)	1,664.8 (19.7)	
Midwest	11,746 (21.2)	7,914 (22.0)	7,922 (30.5)		2,151.8 (25.0)	2,136.4 (25.5)	2,118.2 (25.1)	
South	22,404 (40.4)	13,495 (37.5)	7,940 (30.6)		3,035.9 (35.3)	2,920.2 (34.8)	2,989.9 (35.4)	
West	10,525 (19.0)	7,368 (20.5)	4,973 (19.2)		1,703.9 (19.8)	1,640.0 (19.6)	1,660.5 (19.7)	
Other/missing	35 (0.1)	36 (0.1)	27 (0.1)		7.6 (0.1)	7.5 (0.1)	7.4 (0.1)	

Abbreviations: ACE, angiotensin converting enzyme; CHF, congestive heart failure; COPD, chronic obstructive pulmonary disease; CV, cardiovascular; ED, emergency department; GI, gastrointestinal; NSAID, non-steroidal anti-inflammatory drug; SD, standard deviation; SMD, standardized mean difference; SNRI, serotonin-norepinephrine reuptake inhibitor; SSRI, selective serotonin reuptake inhibitor.

[a]Presented are the average of the absolute SMDs between all pair-wise contrast among the apixaban, rivaroxaban, and warfarin users.

© 2023 Lin KJ et al. *JAMA Network Open.*

eTable 3. Characteristics of the Pre-Frail Population of Medicare Fee-for-Service Beneficiaries With Atrial Fibrillation

Characteristics	Before Propensity Score Weighting				After Propensity Score Weighting			
	Apixaban (n=166,187) No. (%)	Rivaroxaban (n=90,775) No. (%)	Warfarin (n=84,161) No. (%)	SMD[a]	Apixaban (n=26,383) No. (%)	Rivaroxaban (n=26,020) No. (%)	Warfarin (n=25,980) No. (%)	SMD[a]
**Demographic characteristics**								
Age, mean (SD)	77.9 (7.4)	76.8 (7.1)	77.8 (7.3)	0.10	77.5 (7.3)	77.4 (7.2)	77.5 (7.2)	0.00
Female	90,128 (54.2)	47,201 (52.0)	42,487 (50.5)	0.05	13,617.8 (51.6)	13,513.1 (51.9)	13,529.5 (52.1)	0.01
Race				0.05				0.01
Black	6,726 (4.0)	3,712 (4.1)	3,280 (3.9)		1,044.0 (4.0)	1,061.6 (4.1)	1,029.8 (4.0)	
White	151,166 (91.0)	82,072 (90.4)	77,358 (91.9)		24,099.1 (91.3)	23,714.4 (91.1)	23,745.6 (91.4)	
Other/missing	8,295 (5.0)	4,991 (5.5)	3,523 (4.2)		1,239.9 (4.7)	1,244.2 (4.8)	1,204.1 (4.6)	
**Clinical characteristics, mean (SD)**								
CHA_2DS_2-VASc score	4.2 (1.3)	4.1 (1.3)	4.3 (1.4)	0.08	4.2 (1.3)	4.2 (1.3)	4.2 (1.3)	0.01
HAS-BLED score	2.3 (0.6)	2.3 (0.6)	2.1 (0.7)	0.04	2.3 (0.6)	2.3 (0.6)	2.3 (0.6)	0.00
Combined comorbidity score	3.0 (2.3)	2.7 (2.2)	2.5 (2.2)	0.10	2.9 (2.3)	2.9 (2.3)	2.9 (2.3)	0.01
Claims-based frailty index	0.2 (0.0)	0.2 (0.0)	0.2 (0.0)	0.07	0.2 (0.0)	0.2 (0.0)	0.2 (0.0)	0.01
**CV comorbidities**								
Acute myocardial infarction	10,861 (6.5)	5,021 (5.5)	6,187 (7.4)	0.05	1,691.4 (6.4)	1,689.0 (6.5)	1,706.9 (6.6)	0.00
Cardio-ablation	968 (0.6)	546 (0.6)	361 (0.4)	0.02	131.4 (0.5)	130.8 (0.5)	133.0 (0.5)	0.00
Cardioversion	12,893 (7.8)	6,138 (6.8)	3,016 (3.6)	0.12	1,420.3 (5.4)	1,350.0 (5.2)	1,377.4 (5.3)	0.01
Cerebrovascular disease	34,960 (21.0)	19,149 (21.1)	20,374 (24.2)	0.05	5,847.4 (22.2)	5,780.0 (22.2)	5,825.5 (22.4)	0.01
CHF (inpatient)	30,661 (18.4)	14,157 (15.6)	16,057 (19.1)	0.06	4,503.3 (17.1)	4,615.4 (17.7)	4,605.4 (17.7)	0.01
CHF (outpatient)	43,222 (26.0)	23,264 (25.6)	26,549 (31.5)	0.09	7,368.7 (27.9)	7,272.0 (27.9)	7,378.6 (28.4)	0.00
Coronary revascularization	3,599 (2.2)	1,766 (1.9)	2,175 (2.6)	0.03	589.1 (2.2)	589.2 (2.3)	584.1 (2.2)	0.00
Hypertension	147,683 (88.9)	78,584 (86.6)	71,190 (84.6)	0.08	22,785.2 (86.4)	22,506.0 (86.5)	22,448.7 (86.4)	0.01
Ischemic heart disease	77,890 (46.9)	41,413 (45.6)	40,505 (48.1)	0.03	12,466.8 (47.3)	12,146.8 (46.7)	12,299.3 (47.3)	0.00
Peripheral revascularization	26,538 (16.0)	13,748 (15.1)	13,828 (16.4)	0.02	4,187.0 (15.9)	4,136.9 (15.9)	4,144.8 (16.0)	0.01
Stroke (inpatient)	9,658 (5.8)	4,320 (4.8)	5,499 (6.5)	0.05	1,419.4 (5.4)	1,473.8 (5.7)	1,458.6 (5.6)	0.00
Stroke (outpatient)	32,260 (19.4)	16,349 (18.0)	16,757 (19.9)	0.03	5,026.1 (19.1)	4,966.8 (19.1)	4,962.3 (19.1)	0.01
Transient ischemic attack	9,607 (5.8)	4,934 (5.4)	4,842 (5.8)	0.01	1,460.6 (5.5)	1,460.8 (5.6)	1,486.1 (5.7)	0.00
Syncope	17,111 (10.3)	8,868 (9.8)	7,448 (8.8)	0.03	2,490.1 (9.4)	2,469.8 (9.5)	2,497.6 (9.6)	0.00
**Non-CV comorbidities**								
Acute renal failure	21,089 (12.7)	8,298 (9.1)	10,459 (12.4)	0.08	2,911.1 (11.0)	2,969.2 (11.4)	2,900.4 (11.2)	0.01
Alcohol abuse or dependence	3,478 (2.1)	1,788 (2.0)	1,351 (1.6)	0.02	492.6 (1.9)	498.9 (1.9)	474.5 (1.8)	0.00
Anemia	41,752 (25.1)	21,001 (23.1)	22,463 (26.7)	0.06	6,579.7 (24.9)	6,509.3 (25.0)	6,548.1 (25.2)	0.00
Chronic kidney disease	30,583 (18.4)	12,755 (14.1)	16,915 (20.1)	0.11	4,659.5 (17.7)	4,608.1 (17.7)	4,592.0 (17.7)	0.00
COPD	42,514 (25.6)	22,979 (25.3)	21,898 (26.0)	0.01	6,720.8 (25.5)	6,763.6 (26.0)	6,756.7 (26.0)	0.01

© 2023 Lin KJ et al. *JAMA Network Open.*

Characteristics	Before Propensity Score Weighting				After Propensity Score Weighting			
	Apixaban (n=166,187) No. (%)	Rivaroxaban (n=90,775) No. (%)	Warfarin (n=84,161) No. (%)	SMD[a]	Apixaban (n=26,383) No. (%)	Rivaroxaban (n=26,020) No. (%)	Warfarin (n=25,980) No. (%)	SMD[a]
Dementia	4,728 (2.8)	2,500 (2.8)	2,438 (2.9)	0.01	734.1 (2.8)	767.5 (2.9)	730.1 (2.8)	0.01
Diabetes	60,973 (36.7)	34,328 (37.8)	33,537 (39.8)	0.04	10,248.1 (38.8)	10,035.8 (38.6)	10,069.4 (38.8)	0.00
Endoscopy	2,622 (1.6)	1,254 (1.4)	1,675 (2.0)	0.03	424.2 (1.6)	431.2 (1.7)	434.7 (1.7)	0.00
Falls	1,483 (0.9)	648 (0.7)	595 (0.7)	0.01	203.4 (0.8)	199.3 (0.8)	199.1 (0.8)	0.00
Fractures	1,521 (0.9)	977 (1.1)	1,071 (1.3)	0.02	274.2 (1.0)	299.4 (1.2)	283.2 (1.1)	0.01
Frailty	0 (0)	0 (0)	0 (0)	0.00	0 (0)	0 (0)	0 (0)	0.00
GI bleeding (inpatient)	5,120 (3.1)	2,183 (2.4)	2,933 (3.5)	0.04	775.8 (2.9)	785.4 (3.0)	779.1 (3.0)	0.00
GI bleeding (outpatient)	9,452 (5.7)	4,962 (5.5)	5,635 (6.7)	0.03	1,592.1 (6.0)	1,572.8 (6.0)	1,569.7 (6.0)	0.00
Home oxygen use	4,484 (2.7)	2,156 (2.4)	1,956 (2.3)	0.02	624.7 (2.4)	652.6 (2.5)	651.1 (2.5)	0.01
Liver disease	7,600 (4.6)	3,560 (3.9)	2,840 (3.4)	0.04	1,039.3 (3.9)	1,018.8 (3.9)	1,022.4 (3.9)	0.00
Malignancy	31,311 (18.8)	16,904 (18.6)	15,803 (18.8)	0.00	4,941.1 (18.7)	4,928.9 (18.9)	4,900.8 (18.9)	0.00
Obesity	30,800 (18.5)	16,197 (17.8)	14,469 (17.2)	0.02	4,724.0 (17.9)	4,675.8 (18.0)	4,670.3 (18.0)	0.00
Peptic Ulcer	2,832 (1.7)	1,379 (1.5)	1,415 (1.7)	0.01	419.1 (1.6)	422.4 (1.6)	424.0 (1.6)	0.00
Smoking	22,504 (13.5)	16,168 (17.8)	16,950 (20.1)	0.12	4,588.6 (17.4)	4,585.0 (17.6)	4,689.5 (18.1)	0.01
**CV medications**								
ACE inhibitors	45,299 (27.3)	24,989 (27.5)	25,847 (30.7)	0.05	7,562.9 (28.7)	7,482.9 (28.8)	7,487.3 (28.8)	0.00
Angiotensin II receptor blockers	50,748 (30.5)	27,067 (29.8)	21,084 (25.1)	0.08	7,486.5 (28.4)	7,302.5 (28.1)	7,304.9 (28.1)	0.00
Antiarrhythmic agents	36,566 (22.0)	20,031 (22.1)	15,702 (18.7)	0.06	5,476.7 (20.8)	5,219.5 (20.1)	5,426.5 (20.9)	0.01
Antiplatelet agent	27,130 (16.3)	14,041 (15.5)	12,283 (14.6)	0.03	4,139.4 (15.7)	4,016.8 (15.4)	4,090.7 (15.7)	0.01
Beta-blockers	115,928 (69.8)	61,182 (67.4)	58,239 (69.2)	0.03	18,181.8 (68.9)	17,868.8 (68.7)	17,932.7 (69.0)	0.01
Calcium channel blockers	3,770 (2.3)	1,955 (2.2)	1,950 (2.3)	0.01	585.9 (2.2)	589.8 (2.3)	591.2 (2.3)	0.00
Diuretics	90,788 (54.6)	48,622 (53.6)	48,889 (58.1)	0.06	14,777.1 (56.0)	14,529.1 (55.8)	14,572.0 (56.1)	0.00
Fibrates	7,054 (4.2)	4,125 (4.5)	3,975 (4.7)	0.02	1,236.0 (4.7)	1,216.4 (4.7)	1,222.6 (4.7)	0.00
Statins	106,249 (63.9)	56,819 (62.6)	52,555 (62.4)	0.02	16,743.9 (63.5)	16,386.4 (63.0)	16,403.5 (63.1)	0.01
Nitrates	20,019 (12.0)	10,397 (11.5)	11,743 (14.0)	0.05	3,373.3 (12.8)	3,265.1 (12.5)	3,343.2 (12.9)	0.01
**Non-CV medications**								
Anticonvulsants	26,659 (16.0)	13,767 (15.2)	12,071 (14.3)	0.03	4,065.7 (15.4)	4,006.5 (15.4)	4,039.2 (15.5)	0.00
Antidepressants - SSRI/SNRI	31,751 (19.1)	17,329 (19.1)	14,606 (17.4)	0.03	4,904.7 (18.6)	4,851.3 (18.6)	4,847.9 (18.7)	0.00
Antidepressants - Tricyclics	4,338 (2.6)	2,466 (2.7)	2,328 (2.8)	0.01	721.1 (2.7)	712.1 (2.7)	718.5 (2.8)	0.00
Antidepressants - Other	12,738 (7.7)	6,413 (7.1)	5,381 (6.4)	0.03	1,847.0 (7.0)	1,844.2 (7.1)	1,827.0 (7.0)	0.00
Antipsychotic agents	2,772 (1.7)	1,558 (1.7)	1,278 (1.5)	0.01	428.3 (1.6)	437.7 (1.7)	437.3 (1.7)	0.00
Anxiolytics - Benzodiazepines	28,902 (17.4)	16,029 (17.7)	12,525 (14.9)	0.05	4,437.4 (16.8)	4,315.9 (16.6)	4,375.4 (16.8)	0.00
Anxiolytics - Non-benzodiazepines	2,026 (1.2)	980 (1.1)	729 (0.9)	0.02	270.5 (1.0)	268.4 (1.0)	262.7 (1.0)	0.00
Bronchodilators	32,718 (19.7)	17,785 (19.6)	14,985 (17.8)	0.03	5,003.3 (19.0)	5,045.8 (19.4)	5,012.1 (19.3)	0.01

© 2023 Lin KJ et al. *JAMA Network Open.*

Characteristics	Before Propensity Score Weighting				After Propensity Score Weighting			
	Apixaban (n=166,187) No. (%)	Rivaroxaban (n=90,775) No. (%)	Warfarin (n=84,161) No. (%)	SMD[a]	Apixaban (n=26,383) No. (%)	Rivaroxaban (n=26,020) No. (%)	Warfarin (n=25,980) No. (%)	SMD[a]
Corticosteroids, inhaled	37,738 (22.7)	20,894 (23.0)	16,581 (19.7)	0.05	5,752.1 (21.8)	5,724.5 (22.0)	5,717.1 (22.0)	0.00
Corticosteroids, oral	58,840 (35.4)	31,415 (34.6)	26,117 (31.0)	0.06	8,790.9 (33.3)	8,708.9 (33.5)	8,704.3 (33.5)	0.00
Dementia drugs	4,681 (2.8)	2,516 (2.8)	2,197 (2.6)	0.01	729.0 (2.8)	735.2 (2.8)	722.8 (2.8)	0.00
Diabetes agents - Insulin	10,258 (6.2)	5,366 (5.9)	5,923 (7.0)	0.03	1,768.1 (6.7)	1,764.9 (6.8)	1,760.8 (6.8)	0.00
Diabetes agents - Metformin	29,464 (17.7)	16,737 (18.4)	15,010 (17.8)	0.01	4,848.3 (18.4)	4,762.3 (18.3)	4,755.5 (18.3)	0.00
Diabetes agents - Other	9,047 (5.4)	5,015 (5.5)	4,228 (5.0)	0.02	1,430.9 (5.4)	1,422.7 (5.5)	1,432.3 (5.5)	0.00
Diabetes agents - Sulfonylurea	15,431 (9.3)	8,693 (9.6)	9,525 (11.3)	0.05	2,715.3 (10.3)	2,673.5 (10.3)	2,681.0 (10.3)	0.00
Estrogen	5,664 (3.4)	3,260 (3.6)	2,198 (2.6)	0.04	807.2 (3.1)	804.8 (3.1)	813.2 (3.1)	0.00
GI - H2 blockers	13,749 (8.3)	6,777 (7.5)	6,183 (7.3)	0.02	2,007.7 (7.6)	2,007.2 (7.7)	1,995.1 (7.7)	0.00
GI - Proton-pump inhibitors	54,134 (32.6)	28,685 (31.6)	26,050 (31.0)	0.02	8,417.2 (31.9)	8,288.0 (31.9)	8,312.2 (32.0)	0.00
GI - Antacids	3,326 (2.0)	1,705 (1.9)	1,440 (1.7)	0.01	491.4 (1.9)	488.5 (1.9)	488.3 (1.9)	0.00
Hypnotics	12,886 (7.8)	7,776 (8.6)	5,991 (7.1)	0.04	2,028.7 (7.7)	1,992.3 (7.7)	2,009.6 (7.7)	0.00
NSAIDs	29,820 (17.9)	17,621 (19.4)	12,576 (14.9)	0.08	4,498.7 (17.1)	4,447.3 (17.1)	4,460.8 (17.2)	0.00
Opioids	60,790 (36.6)	35,111 (38.7)	32,520 (38.6)	0.03	10,114.5 (38.3)	10,009.2 (38.5)	10,069.6 (38.8)	0.01
Parkinsonism drugs	4,960 (3.0)	2,737 (3.0)	2,340 (2.8)	0.01	783.3 (3.0)	763.8 (2.9)	779.9 (3.0)	0.00
Thyroid hormone replacement	38,413 (23.1)	19,872 (21.9)	18,481 (22.0)	0.02	5,918.0 (22.4)	5,797.4 (22.3)	5,812.9 (22.4)	0.00
**Healthcare utilization, mean (SD)**								
Hospitalizations	0.7 (0.8)	0.7 (0.8)	0.7 (0.8)	0.06	0.7 (0.8)	0.7 (0.8)	0.7 (0.8)	0.02
Skilled nursing facility	0.1 (0.6)	0.1 (0.5)	0.2 (0.7)	0.08	0.1 (0.6)	0.1 (0.6)	0.1 (0.6)	0.01
ED visits	0.7 (1.1)	0.6 (1.0)	0.6 (1.0)	0.03	0.6 (1.0)	0.7 (1.0)	0.6 (1.0)	0.01
Home health day	0.2 (0.6)	0.2 (0.6)	0.2 (0.6)	0.02	0.2 (0.6)	0.2 (0.6)	0.2 (0.6)	0.01
**Healthcare costs, $, mean (SD)**								
Inpatient	7,958.9 (13,137.0)	6,757.2 (11,755.2)	8,720.1 (14,899.2)	0.10	7,373.2 (12,824.7)	7,602.1 (12,760.9)	8,180.4 (14,329.4)	0.04
Outpatient	9,088.8 (11,906.1)	8,565.5 (11,101.5)	8,159.9 (10,148.7)	0.06	8,578.6 (11,126.8)	8,607.9 (11,127.1)	8,511.4 (10,468.6)	0.01
Pharmacy	4,014.8 (11,556.1)	3,232.8 (9,691.2)	2,097.6 (7,088.2)	0.14	3,145.6 (9,537.6)	3,146.3 (9,600.0)	2,922.6 (8,702.6)	0.02
Skilled nursing facility	950.7 (5,192.1)	750.4 (4,641.6)	1,308.1 (6,214.7)	0.07	918.4 (5,125.7)	1,016.4 (5,436.2)	966.1 (5,301.3)	0.01
Home health	471.3 (1,706.4)	405.1 (1,607.4)	426.5 (1,618.3)	0.03	418.0 (1,582.5)	433.2 (1,637.0)	436.4 (1,642.1)	0.04
Durable medical equipment	313.3 (1,879.7)	292.4 (1,296.4)	344.8 (2,135.3)	0.02	325.8 (2,029.5)	305.5 (1,351.3)	329.2 (1,874.0)	0.01
**Co-payment, $, mean (SD)**								

© 2023 Lin KJ et al. *JAMA Network Open.*

Characteristics	Before Propensity Score Weighting				After Propensity Score Weighting			
	Apixaban (n=166,187) No. (%)	Rivaroxaban (n=90,775) No. (%)	Warfarin (n=84,161) No. (%)	SMD[a]	Apixaban (n=26,383) No. (%)	Rivaroxaban (n=26,020) No. (%)	Warfarin (n=25,980) No. (%)	SMD[a]
Inpatient	2,229.7 (2,049.7)	2,043.0 (1,943.3)	2,060.3 (1,880.3)	0.06	2,055.63 (1,957.88)	2,104.41 (2,004.30)	2,091.44 (1,895.33)	0.02
Outpatient	1,723.4 (2,268.1)	1,644.6 (2,144.7)	1,552.4 (1,947.5)	0.05	1,638.3 (2,126.1)	1,646.5 (2,187.4)	1,627.7 (2,012.8)	0.01
Pharmacy	667.0 (906.8)	556.1 (802.4)	412.4 (683.7)	0.21	542.1 (814.4)	551.6 (806.8)	539.3 (793.5)	0.01
Skilled nursing facility	119.1 (961.3)	95.6 (851.7)	171.0 (1,145.7)	0.05	118.2 (963.1)	131.0 (1,004.2)	120.8 (951.7)	0.01
Durable medical equipment	69.8 (388.2)	65.5 (271.1)	76.6 (437.6)	0.02	72.5 (418.0)	68.4 (282.5)	73.2 (386.4)	0.02
**Year of the cohort entry**				0.67				0.04
2013	2,822 (1.7)	11,484 (12.7)	15,554 (18.5)		1,931.6 (7.3)	1,856.4 (7.1)	2,081.0 (8.0)	
2014	11,642 (7.0)	15,225 (16.8)	18,491 (22.0)		4,568.1 (17.3)	4,394.8 (16.9)	4,809.9 (18.5)	
2015	20,711 (12.5)	13,069 (14.4)	15,823 (18.8)		4,767.6 (18.1)	4,748.4 (18.2)	4,705.3 (18.1)	
2016	26,722 (16.1)	12,025 (13.2)	12,126 (14.4)		4,356.9 (16.5)	4,349.5 (16.7)	4,263.7 (16.4)	
2017	27,085 (16.3)	15,434 (17.0)	9,576 (11.4)		4,253.7 (16.1)	4,246.5 (16.3)	4,089.6 (15.7)	
2018	37,545 (22.6)	12,745 (14.0)	7,052 (8.4)		3,555.6 (13.5)	3,538.3 (13.6)	3,313.2 (12.8)	
2019	39,660 (23.9)	10,793 (11.9)	5,539 (6.6)		2,949.4 (11.2)	2,886.3 (11.1)	2,716.8 (10.5)	
**Geographic region**				0.17				0.01
Northeast	31,005 (18.7)	16,992 (18.7)	16,675 (19.8)		5,051.0 (19.1)	5,034.7 (19.3)	4,948.2 (19.0)	
Midwest	37,888 (22.8)	21,198 (23.4)	26,250 (31.2)		6,931.0 (26.3)	6,875.4 (26.4)	6,878.6 (26.5)	
South	68,937 (41.5)	34,987 (38.5)	25,948 (30.8)		9,492.7 (36.0)	9,267.1 (35.6)	9,428.4 (36.3)	
West	28,203 (17.0)	17,460 (19.2)	15,171 (18.0)		4,878.3 (18.5)	4,810.2 (18.5)	4,689.6 (18.1)	
Other/missing	154 (0.1)	138 (0.2)	117 (0.1)		29.9 (0.1)	32.7 (0.1)	34.6 (0.1)	

Abbreviations: ACE, angiotensin converting enzyme; CHF, congestive heart failure; COPD, chronic obstructive pulmonary disease; CV, cardiovascular; ED, emergency department; GI, gastrointestinal; NSAID, non-steroidal anti-inflammatory drug; SD, standard deviation; SMD, standardized mean difference; SNRI, serotonin-norepinephrine reuptake inhibitor; SSRI, selective serotonin reuptake inhibitor.

[a]Presented are the average of the absolute SMDs between all pair-wise contrast among the apixaban, rivaroxaban, and warfarin users.

© 2023 Lin KJ et al. *JAMA Network Open.*

eTable 4. Characteristics of the Frail Population of Medicare Fee-for-Service Beneficiaries With Atrial Fibrillation

Characteristics	Before Propensity Score Weighting				After Propensity Score Weighting			
	Apixaban (n=66,588) No. (%)	Rivaroxaban (n=31,325) No. (%)	Warfarin (n=37,681) No. (%)	SMD[a]	Apixaban (n=10,248) No. (%)	Rivaroxaban (n=10,193) No. (%)	Warfarin (n=10,176) No. (%)	SMD[a]
**Demographic characteristics**								
Age, mean (SD)	79.9 (7.8)	78.8 (7.7)	79.2 (7.7)	0.10	79.2 (7.8)	79.1 (7.7)	79.1 (7.7)	0.01
Female	42,834 (64.3)	19,770 (63.1)	22,785 (60.5)	0.05	6,339.4 (61.9)	6,336.0 (62.2)	6,333.1 (62.2)	0.01
Race				0.05				0.01
Black	4,072 (6.1)	1,966 (6.3)	2,456 (6.5)		631.7 (6.2)	646.7 (6.3)	644.0 (6.3)	
White	59,150 (88.8)	27,610 (88.1)	33,730 (89.5)		9,147.4 (89.3)	9,067.3 (89.0)	9,073.3 (89.2)	
Other/missing	3,366 (5.1)	1,749 (5.6)	1,495 (4.0)		468.4 (4.6)	478.8 (4.7)	458.3 (4.5)	
**Clinical characteristics, mean (SD)**								
CHA_2DS_2-VASc score	5.5 (1.5)	5.3 (1.5)	5.5 (1.5)	0.07	5.4 (1.5)	5.4 (1.5)	5.4 (1.5)	0.01
HAS-BLED score	2.8 (0.7)	2.7 (0.7)	2.8 (0.7)	0.06	2.8 (0.7)	2.8 (0.7)	2.8 (0.7)	0.00
Combined comorbidity score	5.9 (2.8)	5.5 (2.8)	5.8 (2.8)	0.09	5.7 (2.8)	5.8 (2.8)	5.7 (2.8)	0.01
Claims-based frailty index	0.3 (0.1)	0.3 (0.1)	0.3 (0.1)	0.02	0.3 (0.1)	0.3 (0.1)	0.3 (0.1)	0.01
**CV comorbidities**								
Acute myocardial infarction	7,960 (12.0)	3,228 (10.3)	4,835 (12.8)	0.05	1,191.9 (11.6)	1,179.6 (11.6)	1,189.3 (11.7)	0.00
Cardio-ablation	394 (0.6)	196 (0.6)	188 (0.5)	0.01	55.3 (0.5)	53.9 (0.5)	56.6 (0.6)	0.00
Cardioversion	3,453 (5.2)	1,273 (4.1)	1,036 (2.7)	0.08	409.8 (4.0)	383.4 (3.8)	399.4 (3.9)	0.01
Cerebrovascular disease	28,374 (42.6)	13,220 (42.2)	18,028 (47.8)	0.08	4,496.7 (43.9)	4,510.5 (44.3)	4,498.1 (44.2)	0.01
CHF (inpatient)	28,048 (42.1)	12,180 (38.9)	16,458 (43.7)	0.07	4,224.9 (41.2)	4,243.2 (41.6)	4,226.0 (41.5)	0.00
CHF (outpatient)	31,276 (47.0)	15,033 (48.0)	20,268 (53.8)	0.09	5,103.5 (49.8)	5,066.1 (49.7)	5,062.4 (49.8)	0.00
Coronary revascularization	1,548 (2.3)	677 (2.2)	1,035 (2.7)	0.03	253.1 (2.5)	245.7 (2.4)	245.7 (2.4)	0.01
Hypertension	63,995 (96.1)	29,824 (95.2)	35,869 (95.2)	0.03	9,774.1 (95.4)	9,738.1 (95.5)	9,721.7 (95.5)	0.01
Ischemic heart disease	43,635 (65.5)	20,088 (64.1)	24,589 (65.3)	0.02	6,685.6 (65.2)	6,559.0 (64.4)	6,613.4 (65.0)	0.00
Peripheral revascularization	21,656 (32.5)	9,840 (31.4)	12,431 (33.0)	0.02	3,300.9 (32.2)	3,288.2 (32.3)	3,261.0 (32.0)	0.01
Stroke (inpatient)	11,416 (17.1)	4,677 (14.9)	7,772 (20.6)	0.10	1,736.6 (16.9)	1,782.1 (17.5)	1,765.0 (17.3)	0.00
Stroke (outpatient)	24,662 (37.0)	10,806 (34.5)	14,612 (38.8)	0.06	3,732.6 (36.4)	3,734.2 (36.6)	3,729.3 (36.6)	0.00
Transient ischemic attack	7,367 (11.1)	3,315 (10.6)	4,285 (11.4)	0.02	1,111.3 (10.8)	1,117.8 (11.0)	1,125.9 (11.1)	0.00
Syncope	11,445 (17.2)	5,205 (16.6)	5,658 (15.0)	0.04	1,663.0 (16.2)	1,644.9 (16.1)	1,653.4 (16.2)	0.00
**Non-CV comorbidities**								
Acute renal failure	23,324 (35.0)	8,963 (28.6)	12,888 (34.2)	0.09	3,315.7 (32.4)	3,334.2 (32.7)	3,300.9 (32.4)	0.01
Alcohol abuse or dependence	3,104 (4.7)	1,399 (4.5)	1,415 (3.8)	0.03	453.4 (4.4)	464.2 (4.6)	450.3 (4.4)	0.00
Anemia	33,576 (50.4)	15,550 (49.6)	19,691 (52.3)	0.04	5,171.2 (50.5)	5,163.4 (50.7)	5,147.2 (50.6)	0.00
Chronic kidney disease	24,766 (37.2)	9,656 (30.8)	14,340 (38.1)	0.10	3,642.3 (35.5)	3,595.4 (35.3)	3,624.2 (35.6)	0.00
COPD	34,200 (51.4)	16,287 (52.0)	19,459 (51.6)	0.01	5,303.5 (51.8)	5,303.6 (52.0)	5,265.6 (51.7)	0.00

© 2023 Lin KJ et al. *JAMA Network Open.*

Characteristics	Before Propensity Score Weighting				After Propensity Score Weighting			
	Apixaban (n=66,588) No. (%)	Rivaroxaban (n=31,325) No. (%)	Warfarin (n=37,681) No. (%)	SMD[a]	Apixaban (n=10,248) No. (%)	Rivaroxaban (n=10,193) No. (%)	Warfarin (n=10,176) No. (%)	SMD[a]
Dementia	22,453 (33.7)	10,497 (33.5)	11,877 (31.5)	0.03	3,295.7 (32.2)	3,356.8 (32.9)	3,288.6 (32.3)	0.01
Diabetes	37,448 (56.2)	17,982 (57.4)	22,361 (59.3)	0.04	5,968.8 (58.2)	5,911.0 (58.0)	5,911.2 (58.1)	0.00
Endoscopy	2,778 (4.2)	1,378 (4.4)	1,930 (5.1)	0.03	468.9 (4.6)	464.6 (4.6)	467.4 (4.6)	0.00
Falls	2,529 (3.8)	1,075 (3.4)	1,086 (2.9)	0.03	342.9 (3.3)	348.0 (3.4)	339.0 (3.3)	0.00
Fractures	2,978 (4.5)	1,995 (6.4)	2,243 (6.0)	0.06	551.8 (5.4)	579.3 (5.7)	558.6 (5.5)	0.01
Frailty	66,588 (100)	31,325 (100)	37,681 (100)	0.00	10,247.5 (100)	10,192.7 (100)	10,175.6 (100)	0.00
GI bleeding (inpatient)	5,438 (8.2)	2,294 (7.3)	3,465 (9.2)	0.05	847.8 (8.3)	843.8 (8.3)	842.7 (8.3)	0.00
GI bleeding (outpatient)	5,154 (7.7)	2,412 (7.7)	3,201 (8.5)	0.02	842.2 (8.2)	819.6 (8.0)	829.2 (8.1)	0.00
Home oxygen use	10,049 (15.1)	4,435 (14.2)	5,034 (13.4)	0.03	1,437.3 (14.0)	1,445.9 (14.2)	1,437.6 (14.1)	0.00
Liver disease	4,438 (6.7)	1,774 (5.7)	1,869 (5.0)	0.05	591.0 (5.8)	595.2 (5.8)	582.8 (5.7)	0.00
Malignancy	12,355 (18.6)	5,577 (17.8)	7,015 (18.6)	0.01	1,883.9 (18.4)	1,880.6 (18.5)	1,878.7 (18.5)	0.00
Obesity	19,271 (28.9)	9,264 (29.6)	10,933 (29.0)	0.01	3,038.7 (29.7)	3,016.1 (29.6)	3,014.1 (29.6)	0.00
Peptic Ulcer	2,185 (3.3)	1,021 (3.3)	1,262 (3.3)	0.00	336.4 (3.3)	337.4 (3.3)	332.5 (3.3)	0.00
Smoking	14,410 (21.6)	8,792 (28.1)	11,856 (31.5)	0.15	2,840.5 (27.7)	2,819.9 (27.7)	2,808.7 (27.6)	0.00
**CV medications**								
ACE inhibitors	20,475 (30.7)	10,163 (32.4)	13,067 (34.7)	0.06	3,359.7 (32.8)	3,359.1 (33.0)	3,362.3 (33.0)	0.00
Angiotensin II receptor blockers	19,557 (29.4)	8,846 (28.2)	9,150 (24.3)	0.08	2,797.0 (27.3)	2,731.0 (26.8)	2,754.3 (27.1)	0.01
Antiarrhythmic agents	13,763 (20.7)	6,435 (20.5)	6,502 (17.3)	0.06	1,995.6 (19.5)	1,910.6 (18.7)	1,988.2 (19.5)	0.01
Antiplatelet agent	15,670 (23.5)	7,154 (22.8)	8,107 (21.5)	0.03	2,341.9 (22.9)	2,267.6 (22.2)	2,331.4 (22.9)	0.01
Beta-blockers	48,100 (72.2)	22,059 (70.4)	26,922 (71.4)	0.03	7,327.5 (71.5)	7,266.8 (71.3)	7,278.9 (71.5)	0.00
Calcium channel blockers	1,941 (2.9)	817 (2.6)	1,039 (2.8)	0.01	276.9 (2.7)	280.3 (2.8)	274.4 (2.7)	0.00
Diuretics	43,947 (66.0)	20,667 (66.0)	25,834 (68.6)	0.04	6,873.3 (67.1)	6,830.6 (67.0)	6,842.1 (67.2)	0.00
Fibrates	3,213 (4.8)	1,581 (5.0)	2,043 (5.4)	0.02	528.3 (5.2)	527.1 (5.2)	520.1 (5.1)	0.00
Statins	45,182 (67.9)	20,571 (65.7)	24,865 (66.0)	0.03	6,852.5 (66.9)	6,786.0 (66.6)	6,797.6 (66.8)	0.00
Nitrates	12,194 (18.3)	5,631 (18.0)	7,517 (19.9)	0.03	1,948.2 (19.0)	1,888.3 (18.5)	1,935.2 (19.0)	0.01
**Non-CV medications**								
Anticonvulsants	22,532 (33.8)	10,198 (32.6)	11,581 (30.7)	0.04	3,347.1 (32.7)	3,315.7 (32.5)	3,333.4 (32.8)	0.00
Antidepressants - SSRI/SNRI	27,776 (41.7)	12,884 (41.1)	14,332 (38.0)	0.05	4,122.2 (40.2)	4,112.5 (40.3)	4,121.5 (40.5)	0.00
Antidepressants - Tricyclics	3,174 (4.8)	1,520 (4.9)	1,768 (4.7)	0.01	483.0 (4.7)	484.8 (4.8)	479.0 (4.7)	0.00
Antidepressants - Other	13,169 (19.8)	5,997 (19.1)	6,514 (17.3)	0.04	1,914.9 (18.7)	1,916.7 (18.8)	1,899.5 (18.7)	0.00
Antipsychotic agents	6,994 (10.5)	3,572 (11.4)	3,624 (9.6)	0.04	1,046.2 (10.2)	1,074.7 (10.5)	1,058.2 (10.4)	0.01
Anxiolytics - Benzodiazepines	18,336 (27.5)	9,091 (29.0)	9,466 (25.1)	0.06	2,826.9 (27.6)	2,790.9 (27.4)	2,828.0 (27.8)	0.01
Anxiolytics - Non-benzodiazepines	2,360 (3.5)	977 (3.1)	934 (2.5)	0.04	306.8 (3.0)	303.8 (3.0)	305.2 (3.0)	0.00
Bronchodilators	22,366 (33.6)	10,800 (34.5)	11,998 (31.8)	0.04	3,407.2 (33.2)	3,421.3 (33.6)	3,393.0 (33.3)	0.00

© 2023 Lin KJ et al. *JAMA Network Open.*

Characteristics	Before Propensity Score Weighting				After Propensity Score Weighting			
	Apixaban (n=66,588) No. (%)	Rivaroxaban (n=31,325) No. (%)	Warfarin (n=37,681) No. (%)	SMD[a]	Apixaban (n=10,248) No. (%)	Rivaroxaban (n=10,193) No. (%)	Warfarin (n=10,176) No. (%)	SMD[a]
Corticosteroids, inhaled	20,655 (31.0)	9,833 (31.4)	10,458 (27.8)	0.05	3,075.6 (30.0)	3,069.9 (30.1)	3,035.0 (29.8)	0.00
Corticosteroids, oral	28,329 (42.5)	13,082 (41.8)	14,416 (38.3)	0.06	4,179.5 (40.8)	4,141.1 (40.6)	4,158.1 (40.9)	0.00
Dementia drugs	10,887 (16.3)	5,316 (17.0)	5,144 (13.7)	0.06	1,574.3 (15.4)	1,580.4 (15.5)	1,566.3 (15.4)	0.00
Diabetes agents - Insulin	11,573 (17.4)	5,328 (17.0)	7,215 (19.1)	0.04	1,859.1 (18.1)	1,867.1 (18.3)	1,841.4 (18.1)	0.00
Diabetes agents - Metformin	14,969 (22.5)	7,425 (23.7)	8,239 (21.9)	0.03	2,357.4 (23.0)	2,340.7 (23.0)	2,345.9 (23.1)	0.00
Diabetes agents - Other	6,302 (9.5)	2,901 (9.3)	3,135 (8.3)	0.03	917.6 (9.0)	912.9 (9.0)	908.4 (8.9)	0.00
Diabetes agents - Sulfonylurea	9,366 (14.1)	4,714 (15.0)	6,351 (16.9)	0.05	1,612.2 (15.7)	1,564.8 (15.4)	1,595.6 (15.7)	0.01
Estrogen	1,933 (2.9)	898 (2.9)	892 (2.4)	0.02	274.7 (2.7)	263.1 (2.6)	265.2 (2.6)	0.00
GI - H2 blockers	9,363 (14.1)	4,172 (13.3)	4,730 (12.6)	0.03	1,340.5 (13.1)	1,348.2 (13.2)	1,331.2 (13.1)	0.00
GI - Proton-pump inhibitors	30,372 (45.6)	14,415 (46.0)	16,556 (43.9)	0.03	4,614.4 (45.0)	4,584.9 (45.0)	4,625.7 (45.5)	0.01
GI - Antacids	2,416 (3.6)	1,081 (3.5)	1,150 (3.1)	0.02	339.0 (3.3)	341.7 (3.4)	343.7 (3.4)	0.00
Hypnotics	7,799 (11.7)	4,148 (13.2)	4,389 (11.6)	0.03	1,232.2 (12.0)	1,214.1 (11.9)	1,230.5 (12.1)	0.00
NSAIDs	12,854 (19.3)	6,646 (21.2)	6,287 (16.7)	0.08	1,920.1 (18.7)	1,914.5 (18.8)	1,911.0 (18.8)	0.00
Opioids	34,124 (51.2)	17,331 (55.3)	20,287 (53.8)	0.06	5,541.2 (54.1)	5,488.7 (53.8)	5,517.2 (54.2)	0.00
Parkinsonism drugs	6,695 (10.1)	3,229 (10.3)	3,478 (9.2)	0.02	1,029.4 (10.0)	1,009.6 (9.9)	1,023.0 (10.1)	0.00
Thyroid hormone replacement	19,421 (29.2)	8,764 (28.0)	10,208 (27.1)	0.03	2,914.1 (28.4)	2,842.4 (27.9)	2,887.2 (28.4)	0.01
**Healthcare utilization, mean (SD)**								
Hospitalizations	1.7 (1.4)	1.7 (1.4)	1.7 (1.4)	0.03	1.7 (1.4)	1.7 (1.4)	1.7 (1.4)	0.03
Skilled nursing facility	1.3 (2.1)	1.3 (2.1)	1.5 (2.2)	0.07	1.3 (2.1)	1.4 (2.2)	1.3 (2.1)	0.02
ED visits	1.2 (1.8)	1.2 (1.9)	1.2 (1.8)	0.02	1.2 (1.8)	1.2 (1.9)	1.2 (1.8)	0.01
Home health day	0.9 (1.4)	0.9 (1.5)	0.8 (1.4)	0.05	0.9 (1.4)	0.9 (1.4)	0.9 (1.4)	0.01
**Healthcare costs, $, mean (SD)**								
Inpatient	21,918.1 (26,519.7)	20,188.8 (25,201.7)	23,312.4 (28,495.6)	0.08	21,240.2 (26,435.6)	21,679.9 (26,409.7)	21,949.8 (27,234.9)	0.02
Outpatient	13,267.6 (13,294.7)	12,551.5 (12,447.6)	12,323.0 (11,221.1)	0.05	12,699.7 (12,210.7)	12,747.7 (12,535.3)	12,664.2 (11,964.9)	0.00
Pharmacy	5,983.1 (12,563.2)	4,914.3 (11,509.2)	3,456.4 (7,837.1)	0.16	4,794.1 (10,703.1)	4,951.0 (12,109.7)	4,496.2 (9,561.7)	0.00
Skilled nursing facility	10,113.1 (17,589.4)	9,771.9 (17,662.6)	11,480.2 (18,672.2)	0.06	10,032.8 (17,549.9)	10,732.8 (18,381.2)	10,255.5 (17,886.7)	0.03
Home health	2,679.8 (4,388.9)	2,592.0 (4,372.6)	2,303.3 (4,070.1)	0.06	2,507.2 (4,260.7)	2,516.4 (4,313.2)	2,493.0 (4,264.7)	0.02
Durable medical equipment	876.4 (2,999.8)	972.7 (3,433.2)	973.7 (3,776.6)	0.02	925.3 (2,972.8)	958.4 (3,416.7)	959.7 (4,244.0)	0.00

© 2023 Lin KJ et al. *JAMA Network Open.*

Characteristics	Before Propensity Score Weighting				After Propensity Score Weighting			
	Apixaban (n=66,588) No. (%)	Rivaroxaban (n=31,325) No. (%)	Warfarin (n=37,681) No. (%)	SMD[a]	Apixaban (n=10,248) No. (%)	Rivaroxaban (n=10,193) No. (%)	Warfarin (n=10,176) No. (%)	SMD[a]
**Co-payment, $, mean (SD)**								
Inpatient	4,065.5 (3,357.9)	3,827.2 (3,279.3)	3,892.4 (3,233.8)	0.05	3,890.9 (3,250.6)	3,939.2 (3,391.0)	3,873.1 (3,072.3)	0.01
Outpatient	2,315.4 (2,627.8)	2,188.1 (2,353.8)	2,116.5 (2,211.9)	0.06	2,210.4 (2,393.6)	2,212.4 (2,372.6)	2,210.8 (2,374.0)	0.00
Pharmacy	688.4 (974.0)	556.3 (877.4)	462.8 (762.5)	0.17	572.0 (876.9)	582.1 (902.6)	576.3 (872.2)	0.01
Skilled nursing facility	1,745.6 (3,783.4)	1,742.4 (3,802.3)	2,033.7 (3,972.4)	0.05	1,754.7 (3,781.5)	1,920.3 (3,972.4)	1,776.9 (3,762.3)	0.03
Durable medical equipment	191.5 (618.7)	211.5 (705.6)	211.9 (775.0)	0.02	201.8 (613.1)	208.4 (702.5)	208.9 (870.2)	0.01
**Year of the cohort entry**				0.78				0.01
2013	826 (1.2)	4,305 (13.7)	7,769 (20.6)		625.6 (6.1)	617.0 (6.1)	629.4 (6.2)	
2014	4,120 (6.2)	5,514 (17.6)	8,626 (22.9)		1,765.4 (17.2)	1,740.7 (17.1)	1,777.1 (17.5)	
2015	8,153 (12.2)	4,692 (15.0)	7,559 (20.1)		1,992.7 (19.4)	1,993.9 (19.6)	1,985.4 (19.5)	
2016	10,657 (16.0)	4,183 (13.4)	5,253 (13.9)		1,771.7 (17.3)	1,770.2 (17.4)	1,754.4 (17.2)	
2017	10,935 (16.4)	5,074 (16.2)	3,984 (10.6)		1,708.9 (16.7)	1,700.4 (16.7)	1,695.9 (16.7)	
2018	15,520 (23.3)	4,195 (13.4)	2,664 (7.1)		1,360.2 (13.3)	1,360.3 (13.3)	1,332.8 (13.1)	
2019	16,377 (24.6)	3,362 (10.7)	1,826 (4.8)		1,023.1 (10.0)	1,010.2 (9.9)	1,000.6 (9.8)	
**Geographic region**				0.18				0.01
Northeast	12,537 (18.8)	5,551 (17.7)	8,181 (21.7)		2,000.1 (19.5)	2,001.4 (19.6)	1,969.0 (19.3)	
Midwest	15,645 (23.5)	7,472 (23.9)	11,608 (30.8)		2,732.5 (26.7)	2,709.2 (26.6)	2,743.6 (27.0)	
South	28,614 (43.0)	12,917 (41.2)	12,063 (32.0)		3,854.9 (37.6)	3,834.9 (37.6)	3,872.7 (38.1)	
West	9,757 (14.7)	5,347 (17.1)	5,807 (15.4)		1,653.2 (16.1)	1,641.2 (16.1)	1,581.6 (15.5)	
Other/missing	35 (0.1)	38 (0.1)	22 (0.1)		6.8 (0.1)	6.1 (0.1)	8.7 (0.1)	

Abbreviations: ACE, angiotensin converting enzyme; CHF, congestive heart failure; COPD, chronic obstructive pulmonary disease; CV, cardiovascular; ED, emergency department; GI, gastrointestinal; NSAID, non-steroidal anti-inflammatory drug; SD, standard deviation; SMD, standardized mean difference; SNRI, serotonin-norepinephrine reuptake inhibitor; SSRI, selective serotonin reuptake inhibitor.

[a]Presented are the average of the absolute SMDs between all pair-wise contrast among the apixaban, rivaroxaban, and warfarin users.

© 2023 Lin KJ et al. *JAMA Network Open.*

eFigure 1. Frailty and Association of Apixaban, Rivaroxaban, and Warfarin With Home Time Lost and Clinical Events in Medicare Fee-for-Service Beneficiaries With Atrial Fibrillation

	Apixaban		Rivaroxaban		Warfarin		Rivaroxaban vs Apixaban	Warfarin vs Apixaban
	Sample Size	Events (Risk or Rate)*	Sample Size	Events (Risk or Rate)*	Sample Size	Events (Risk or Rate)*	Estimate (95% CI)	Estimate (95% CI)
**Home time lost > 14 days in 12 months**								
Total population	45,950	9,233 (20.1)	45,320	9,923 (21.9)	45,280	10,546 (23.3)	1.8 (1.5, 2.1)	3.2 (2.9, 3.5)
Non-frail	8,612	521 (6.0)	8,381	578 (6.9)	8,441	654 (7.7)	0.8 (0.5, 1.2)	1.7 (1.2, 2.2)
Pre-frail	26,383	4,223 (16.0)	26,020	4,448 (17.1)	25,979	4,901 (18.9)	1.1 (0.7, 1.4)	2.9 (2.5, 3.2)
Frail	10,247	4,444 (43.4)	10,193	4,694 (46.0)	10,176	4,777 (46.9)	2.7 (1.9, 3.4)	3.6 (2.8, 4.3)
							$P_{Heterogeneity} < 0.001$	$P_{Heterogeneity} < 0.001$
**Composite endpoint**								
Total population	45,950	5,130 (119.5)	45,320	5,884 (140.8)	45,280	6,192 (148.9)	21.3 (16.4, 26.2)	29.4 (24.5, 34.3)
Non-frail	8,612	312 (37.0)	8,381	396 (48.7)	8,441	427 (52.2)	11.6 (5.3, 17.9)	15.1 (8.7, 21.5)
Pre-frail	26,383	2,343 (93.7)	26,020	2,729 (111.9)	25,979	2,896 (119.3)	18.2 (12.5, 23.8)	25.6 (19.8, 31.3)
Frail	10,247	2,443 (277.3)	10,193	2,653 (308.9)	10,176	2,749 (322.9)	31.6 (15.5, 47.7)	45.5 (29.2, 61.9)
							$P_{Heterogeneity} = 0.050$	$P_{Heterogeneity} = 0.001$
**Ischemic stroke or systemic embolism**								
Total population	45,950	445 (10.3)	45,320	565 (13.3)	45,280	583 (13.8)	3.0 (1.6, 4.5)	3.5 (2.1, 5.0)
Non-frail	8,612	49 (5.8)	8,381	63 (7.7)	8,441	61 (7.5)	1.8 (-0.7, 4.3)	1.6 (-0.9, 4.1)
Pre-frail	26,383	247 (9.8)	26,020	315 (12.7)	25,979	332 (13.5)	2.9 (1.0, 4.7)	3.7 (1.8, 5.6)
Frail	10,247	145 (16.2)	10,193	181 (20.6)	10,176	177 (20.3)	4.4 (0.4, 8.4)	4.1 (0.1, 8.1)
							$P_{Heterogeneity} = 0.558$	$P_{Heterogeneity} = 0.375$
**Major bleeding**								
Total population	45,950	919 (21.3)	45,320	1,431 (34.1)	45,280	1,430 (34.2)	12.8 (10.5, 15.0)	12.9 (10.7, 15.1)
Non-frail	8,612	80 (9.5)	8,381	131 (16.1)	8,441	147 (17.9)	6.6 (3.1, 10.0)	8.4 (4.8, 12.0)
Pre-frail	26,383	499 (19.9)	26,020	787 (32.1)	25,979	753 (30.9)	12.3 (9.4, 15.1)	11.0 (8.2, 13.8)
Frail	10,247	333 (37.6)	10,193	489 (56.6)	10,176	510 (59.5)	19.0 (12.6, 25.5)	21.9 (15.4, 28.5)
							$P_{Heterogeneity} = 0.002$	$P_{Heterogeneity} = 0.002$
**Death**								
Total population	45,950	4,139 (95.1)	45,320	4,382 (102.6)	45,280	4,750 (111.9)	7.5 (3.3, 11.7)	16.7 (12.4, 21.0)
Non-frail	8,612	208 (24.6)	8,381	236 (28.7)	8,441	264 (31.9)	4.1 (-0.8, 9.1)	7.3 (2.2, 12.4)
Pre-frail	26,383	1,781 (70.3)	26,020	1,854 (74.4)	25,979	2,082 (84.1)	4.1 (-0.6, 8.8)	13.7 (8.8, 18.6)
Frail	10,247	2,124 (235.7)	10,193	2,206 (248.4)	10,176	2,306 (261.9)	12.7 (-1.8, 27.1)	26.2 (11.5, 40.8)
							$P_{Heterogeneity} = 0.527$	$P_{Heterogeneity} = 0.026$

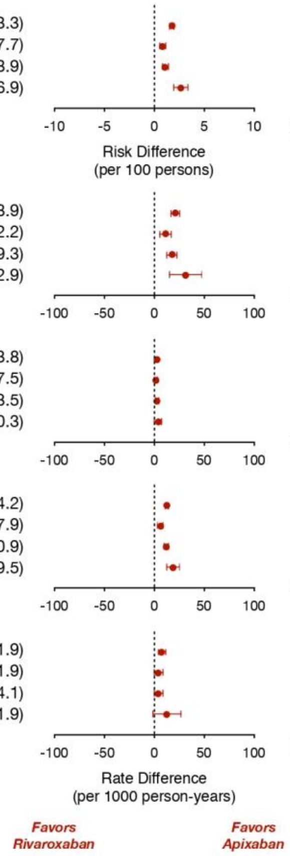

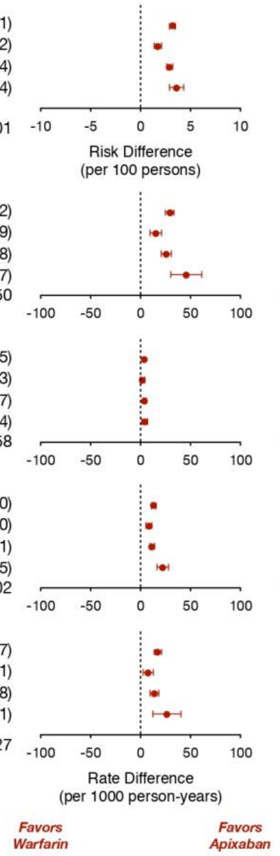

Abbreviation: CI, confidence interval.

The figure displays 1) the risk (per 100 persons) of home time lost >14 days and risk differences (95% confidence interval) and 2) the rates (per 1,000 person-years) of the composite and individual endpoint of ischemic stroke, systemic embolism, major bleeding, or death and rate differences (95% confidence interval) in 12 months (365 days) after initiating an oral anticoagulant in the propensity score overlap-weighted populations. p-for-heterogeneity tests whether the frailty level-specific estimates are different from each other.

© 2023 Lin KJ et al. *JAMA Network Open.*

eFigure 2. Frailty and Association of Apixaban, Rivaroxaban, and Warfarin With Health Care Cost in Medicare Fee-for-Service Beneficiaries With Atrial Fibrillation

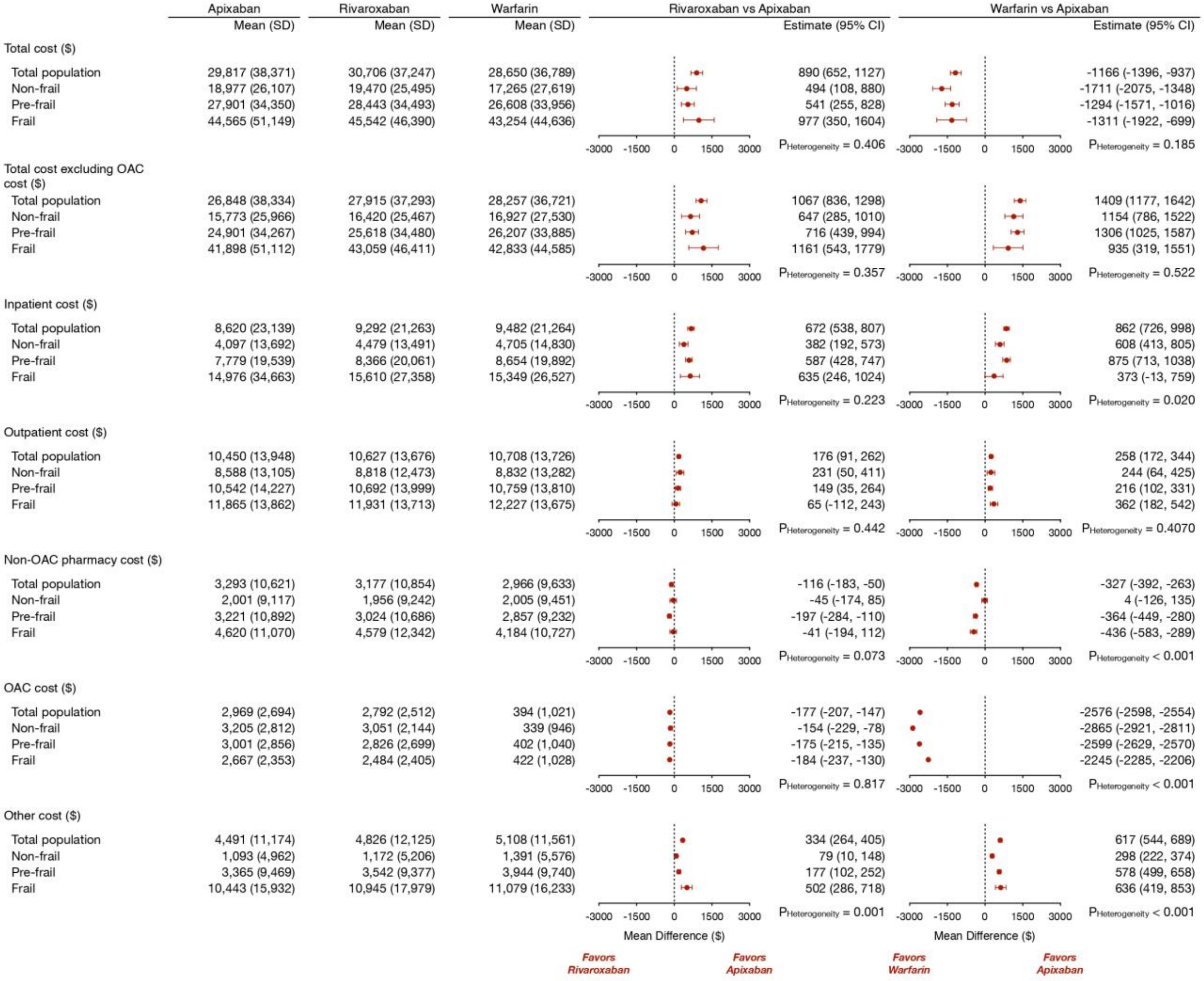

	Apixaban Mean (SD)	Rivaroxaban Mean (SD)	Warfarin Mean (SD)	Rivaroxaban vs Apixaban Estimate (95% CI)	Warfarin vs Apixaban Estimate (95% CI)
**Total cost ($)**					
Total population	29,817 (38,371)	30,706 (37,247)	28,650 (36,789)	890 (652, 1127)	-1166 (-1396, -937)
Non-frail	18,977 (26,107)	19,470 (25,495)	17,265 (27,619)	494 (108, 880)	-1711 (-2075, -1348)
Pre-frail	27,901 (34,350)	28,443 (34,493)	26,608 (33,956)	541 (255, 828)	-1294 (-1571, -1016)
Frail	44,565 (51,149)	45,542 (46,390)	43,254 (44,636)	977 (350, 1604)	-1311 (-1922, -699)
				$P_{Heterogeneity}$ = 0.406	$P_{Heterogeneity}$ = 0.185
**Total cost excluding OAC cost ($)**					
Total population	26,848 (38,334)	27,915 (37,293)	28,257 (36,721)	1067 (836, 1298)	1409 (1177, 1642)
Non-frail	15,773 (25,966)	16,420 (25,467)	16,927 (27,530)	647 (285, 1010)	1154 (786, 1522)
Pre-frail	24,901 (34,267)	25,618 (34,480)	26,207 (33,885)	716 (439, 994)	1306 (1025, 1587)
Frail	41,898 (51,112)	43,059 (46,411)	42,833 (44,585)	1161 (543, 1779)	935 (319, 1551)
				$P_{Heterogeneity}$ = 0.357	$P_{Heterogeneity}$ = 0.522
**Inpatient cost ($)**					
Total population	8,620 (23,139)	9,292 (21,263)	9,482 (21,264)	672 (538, 807)	862 (726, 998)
Non-frail	4,097 (13,692)	4,479 (13,491)	4,705 (14,830)	382 (192, 573)	608 (413, 805)
Pre-frail	7,779 (19,539)	8,366 (20,061)	8,654 (19,892)	587 (428, 747)	875 (713, 1038)
Frail	14,976 (34,663)	15,610 (27,358)	15,349 (26,527)	635 (246, 1024)	373 (-13, 759)
				$P_{Heterogeneity}$ = 0.223	$P_{Heterogeneity}$ = 0.020
**Outpatient cost ($)**					
Total population	10,450 (13,948)	10,627 (13,676)	10,708 (13,726)	176 (91, 262)	258 (172, 344)
Non-frail	8,588 (13,105)	8,818 (12,473)	8,832 (13,282)	231 (50, 411)	244 (64, 425)
Pre-frail	10,542 (14,227)	10,692 (13,999)	10,759 (13,810)	149 (35, 264)	216 (102, 331)
Frail	11,865 (13,862)	11,931 (13,713)	12,227 (13,675)	65 (-112, 243)	362 (182, 542)
				$P_{Heterogeneity}$ = 0.442	$P_{Heterogeneity}$ = 0.4070
**Non-OAC pharmacy cost ($)**					
Total population	3,293 (10,621)	3,177 (10,854)	2,966 (9,633)	-116 (-183, -50)	-327 (-392, -263)
Non-frail	2,001 (9,117)	1,956 (9,242)	2,005 (9,451)	-45 (-174, 85)	4 (-126, 135)
Pre-frail	3,221 (10,892)	3,024 (10,686)	2,857 (9,232)	-197 (-284, -110)	-364 (-449, -280)
Frail	4,620 (11,070)	4,579 (12,342)	4,184 (10,727)	-41 (-194, 112)	-436 (-583, -289)
				$P_{Heterogeneity}$ = 0.073	$P_{Heterogeneity}$ < 0.001
**OAC cost ($)**					
Total population	2,969 (2,694)	2,792 (2,512)	394 (1,021)	-177 (-207, -147)	-2576 (-2598, -2554)
Non-frail	3,205 (2,812)	3,051 (2,144)	339 (946)	-154 (-229, -78)	-2865 (-2921, -2811)
Pre-frail	3,001 (2,856)	2,826 (2,699)	402 (1,040)	-175 (-215, -135)	-2599 (-2629, -2570)
Frail	2,667 (2,353)	2,484 (2,405)	422 (1,028)	-184 (-237, -130)	-2245 (-2285, -2206)
				$P_{Heterogeneity}$ = 0.817	$P_{Heterogeneity}$ < 0.001
**Other cost ($)**					
Total population	4,491 (11,174)	4,826 (12,125)	5,108 (11,561)	334 (264, 405)	617 (544, 689)
Non-frail	1,093 (4,962)	1,172 (5,206)	1,391 (5,576)	79 (10, 148)	298 (222, 374)
Pre-frail	3,365 (9,469)	3,542 (9,377)	3,944 (9,740)	177 (102, 252)	578 (499, 658)
Frail	10,443 (15,932)	10,945 (17,979)	11,079 (16,233)	502 (286, 718)	636 (419, 853)
				$P_{Heterogeneity}$ = 0.001	$P_{Heterogeneity}$ < 0.001

Abbreviations: CI, confidence interval; OAC, oral anticoagulant.

The figure displays the mean cost (standard deviation) in 12 months (365 days) after initiating an oral anticoagulant in the propensity score overlap-weighted populations. p-for-heterogeneity tests whether the frailty level-specific estimates are different from each other.

© 2023 Lin KJ et al. *JAMA Network Open.*

eFigure 3. Frailty and Association of Apixaban, Rivaroxaban, and Warfarin With Home Time Loss and Clinical Events in Medicare Fee-for-Service Beneficiaries With Atrial Fibrillation – Sensitivity Analysis 1 to Exclude Ischemic Stroke and Major Bleeding in the 60 Days Prior to the Index Date

	Apixaban		Rivaroxaban		Warfarin		Rivaroxaban vs Apixaban	Warfarin vs Apixaban
	Sample Size	Events (Risk or Rate)*	Sample Size	Events (Risk or Rate)*	Sample Size	Events (Risk or Rate)*	Estimate (95% CI)	Estimate (95% CI)
Home time lost > 14 days in 12 months								
Total population	44,908	8,877 (19.77)	44,294	9,538 (21.53)	44,244	10,148 (22.94)	1.8 (1.5, 2.1)	3.2 (2.9, 3.5)
Non-frail	8,586	519 (6.04)	8,358	576 (6.89)	8,419	651 (7.74)	0.8 (0.5, 1.2)	1.7 (1.2, 2.1)
Pre-frail	25,946	4,125 (15.90)	25,583	4,352 (17.01)	25,552	4,803 (18.80)	1.1 (0.8, 1.5)	2.9 (2.5, 3.3)
Frail	9,673	4,189 (43.31)	9,625	4,413 (45.85)	9,608	4,491 (46.74)	2.5 (1.8, 3.3)	3.4 (2.6, 4.2)
							$P_{Heterogeneity}$ = 0.001	$P_{Heterogeneity}$ < 0.001
Composite endpoint								
Total population	44,908	4,920 (117.11)	44,294	5,648 (138.10)	44,244	5,955 (146.33)	21.0 (16.1, 25.9)	29.2 (24.3, 34.2)
Non-frail	9,673	2,292 (37.00)	9,625	2,483 (48.79)	9,608	2,589 (51.97)	11.8 (5.5, 18.1)	15.0 (8.5, 21.4)
Pre-frail	25,946	2,287 (92.97)	25,583	2,667 (111.16)	25,552	2,830 (118.46)	18.2 (12.5, 23.9)	25.5 (19.7, 31.3)
Frail	9,673	2,292 (275.24)	9,625	2,483 (305.57)	9,608	2,589 (321.93)	30.3 (13.9, 46.8)	46.7 (29.9, 63.4)
							$P_{Heterogeneity}$ = 0.076	$P_{Heterogeneity}$ = 0.001
Ischemic stroke or systemic embolism								
Total population	44,908	416 (9.82)	44,294	524 (12.60)	44,244	544 (13.16)	2.8 (1.3, 4.2)	3.3 (1.9, 4.8)
Non-frail	9,673	126 (5.81)	9,625	157 (7.75)	9,608	156 (7.32)	1.9 (-0.6, 4.4)	1.5 (-1.0, 4.0)
Pre-frail	25,946	237 (9.55)	25,583	298 (12.20)	25,552	315 (13.00)	2.7 (0.8, 4.5)	3.4 (1.6, 5.3)
Frail	9,673	126 (14.95)	9,625	157 (18.77)	9,608	156 (18.89)	3.8 (-0.1, 7.7)	3.9 (-0.0, 7.9)
							$P_{Heterogeneity}$ = 0.726	$P_{Heterogeneity}$ = 0.402
Major bleeding								
Total population	44,908	880 (20.86)	44,294	1,379 (33.57)	44,244	1,375 (33.63)	12.7 (10.5, 15.0)	12.8 (10.5, 15.0)
Non-frail	9,673	309 (9.52)	9,625	453 (16.15)	9,608	475 (17.93)	6.6 (3.2, 10.1)	8.4 (4.8, 12.0)
Pre-frail	25,946	484 (19.60)	25,583	772 (32.03)	25,552	735 (30.64)	12.4 (9.6, 15.3)	11.0 (8.2, 13.9)
Frail	9,673	309 (36.88)	9,625	453 (55.45)	9,608	475 (58.77)	18.6 (12.0, 25.1)	21.9 (15.2, 28.6)
							$P_{Heterogeneity}$ = 0.002	$P_{Heterogeneity}$ = 0.002
Death								
Total population	44,908	3,972 (93.33)	44,294	4,209 (100.73)	44,244	4,570 (110.03)	7.4 (3.2, 11.6)	16.7 (12.4, 21.0)
Non-frail	9,673	1,997 (24.55)	9,625	2,074 (28.73)	9,608	2,178 (31.74)	4.2 (-0.8, 9.1)	7.2 (2.1, 12.3)
Pre-frail	25,946	1,744 (70.00)	25,583	1,817 (74.16)	25,552	2,038 (83.67)	4.2 (-0.6, 8.9)	13.7 (8.8, 18.6)
Frail	9,673	1,997 (234.70)	9,625	2,074 (247.14)	9,608	2,178 (262.12)	12.4 (-2.4, 27.2)	27.4 (12.3, 42.5)
							$P_{Heterogeneity}$ = 0.566	$P_{Heterogeneity}$ = 0.020

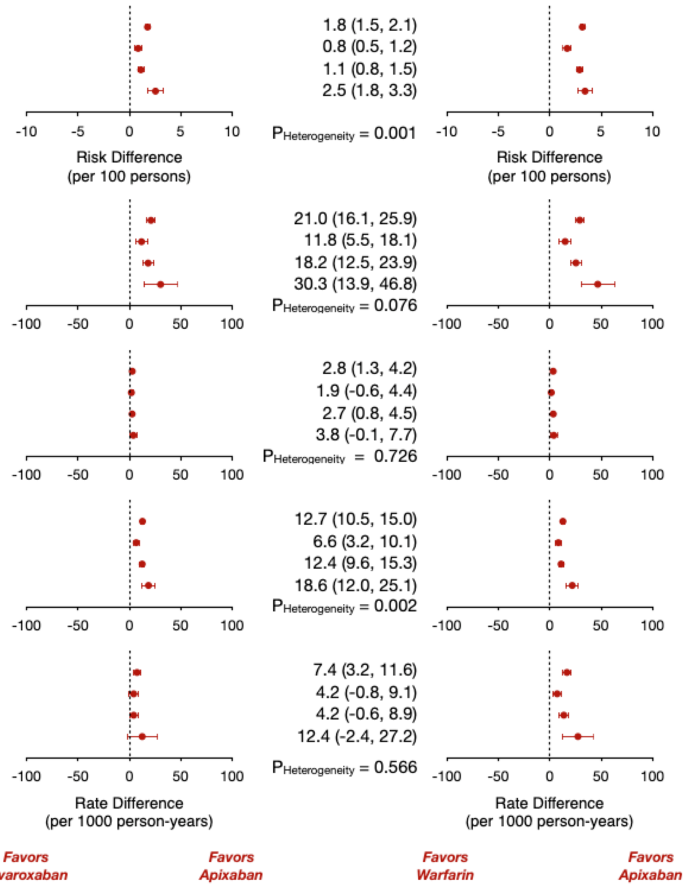

Abbreviation: CI, confidence interval.
The figure displays 1) the risk (per 100 persons) of home time loss >14 days and risk differences (95% confidence interval) and 2) the rates (per 1,000 person-years) of the composite and individual endpoint of ischemic stroke, systemic embolism, major bleeding, or death and rate differences (95% confidence interval) in 12 months (365 days) after initiating an oral anticoagulant in the propensity score overlap-weighted populations. *p*-for-heterogeneity tests whether the frailty level-specific estimates are different from each other.

© 2023 Lin KJ et al. *JAMA Network Open.*

eFigure 4. Frailty and Association of Apixaban, Rivaroxaban, and Warfarin With Health Care Cost in Medicare Fee-for-Service Beneficiaries With Atrial Fibrillation – Sensitivity Analysis 1 to Exclude Ischemic Stroke and Major Bleeding in the 60 Days Prior to the Index Date

	Apixaban	Rivaroxaban	Warfarin	Rivaroxaban vs Apixaban	Warfarin vs Apixaban
	Mean (SD)	Mean (SD)	Mean (SD)	Estimate (95% CI)	Estimate (95% CI)
Total cost ($)					
Total population	29,637 (38,289)	30,509 (36,955)	28,446 (36,718)	872 (632, 1112)	-1191 (-1422, -959)
Non-frail	18,973 (26,117)	19,454 (25,488)	17,255 (27,619)	481 (95, 868)	-1718 (-2082, -1354)
Pre-frail	27,844 (34,317)	28,398 (34,480)	26,563 (33,965)	553 (264, 843)	-1281 (-1562, -1001)
Frail	44,621 (51,466)	45,502 (45,778)	43,236 (44,859)	881 (234, 1528)	-1385 (-2016, -754)
				$P_{Heterogeneity}$ = 0.576	$P_{Heterogeneity}$ = 0.174
Total cost excluding OAC cost ($)					
Total population	26,675 (38,248)	27,723 (36,997)	28,057 (36,652)	1048 (815, 1281)	1383 (1148, 1617)
Non-frail	15,770 (25,974)	16,405 (25,460)	16,917 (27,530)	634 (271, 998)	1147 (779, 1516)
Pre-frail	24,853 (34,231)	25,581 (34,463)	26,166 (33,894)	728 (448, 1008)	1313 (1029, 1597)
Frail	41,982 (51,419)	43,046 (45,792)	42,825 (44,810)	1064 (427, 1701)	843 (208, 1479)
				$P_{Heterogeneity}$ = 0.517	$P_{Heterogeneity}$ = 0.388
Inpatient cost ($)					
Total population	8,561 (23,112)	9,223 (21,192)	9,414 (21,262)	663 (526, 799)	853 (716, 991)
Non-frail	4,095 (13,685)	4,476 (13,494)	4,708 (14,843)	381 (190, 572)	613 (417, 809)
Pre-frail	7,765 (19,541)	8,359 (20,063)	8,645 (19,919)	593 (432, 755)	879 (715, 1044)
Frail	15,058 (34,979)	15,630 (27,356)	15,379 (26,742)	572 (169, 976)	321 (-80, 722)
				$P_{Heterogeneity}$ = 0.238	$P_{Heterogeneity}$ = 0.014
Outpatient cost ($)					
Total population	10,445 (13,951)	10,626 (13,631)	10,700 (13,760)	181 (94, 267)	255 (168, 342)
Non-frail	8,584 (13,109)	8,809 (12,456)	8,828 (13,284)	225 (44, 406)	244 (64, 425)
Pre-frail	10,547 (14,233)	10,698 (13,992)	10,758 (13,813)	151 (35, 267)	212 (95, 328)
Frail	11,912 (13,862)	11,985 (13,554)	12,282 (13,812)	73 (-110, 256)	370 (185, 556)
				$P_{Heterogeneity}$ = 0.515	$P_{Heterogeneity}$ = 0.363
Non-OAC pharmacy cost ($)					
Total population	3,293 (10,640)	3,172 (10,642)	2,964 (9,641)	-121 (-188, -54)	-329 (-394, -265)
Non-frail	2,004 (9,136)	1,955 (9,261)	1,999 (9,427)	-49 (-179, 81)	-5 (-136, 126)
Pre-frail	3,227 (10,895)	3,031 (10,730)	2,869 (9,281)	-196 (-284, -108)	-359 (-444, -273)
Frail	4,669 (11,147)	4,601 (11,289)	4,228 (10,812)	-68 (-222, 86)	-441 (-589, -294)
				$P_{Heterogeneity}$ = 0.117	$P_{Heterogeneity} < 0.001$
OAC cost ($)					
Total population	2,963 (2,709)	2,787 (2,524)	389 (1,013)	-176 (-207, -146)	-2573 (-2596, -2551)
Non-frail	3,204 (2,818)	3,050 (2,144)	339 (944)	-153 (-229, -77)	-2865 (-2921, -2811)
Pre-frail	2,993 (2,864)	2,818 (2,690)	398 (1,034)	-175 (-215, -134)	-2594 (-2624, -2565)
Frail	2,640 (2,380)	2,457 (2,411)	412 (1,013)	-183 (-239, -128)	-2228 (-2269, -2187)
				$P_{Heterogeneity}$ = 0.821	$P_{Heterogeneity} < 0.001$
Other cost ($)					
Total population	4,382 (11,034)	4,708 (11,936)	4,986 (11,416)	326 (256, 396)	604 (531, 676)
Non-frail	1,093 (4,966)	1,171 (5,206)	1,388 (5,573)	78 (9, 147)	295 (220, 372)
Pre-frail	3,320 (9,380)	3,499 (9,333)	3,900 (9,674)	180 (105, 255)	581 (501, 661)
Frail	10,349 (15,898)	10,835 (17,770)	10,942 (16,160)	487 (266, 708)	593 (371, 816)
				$P_{Heterogeneity}$ = 0.001	$P_{Heterogeneity} < 0.001$

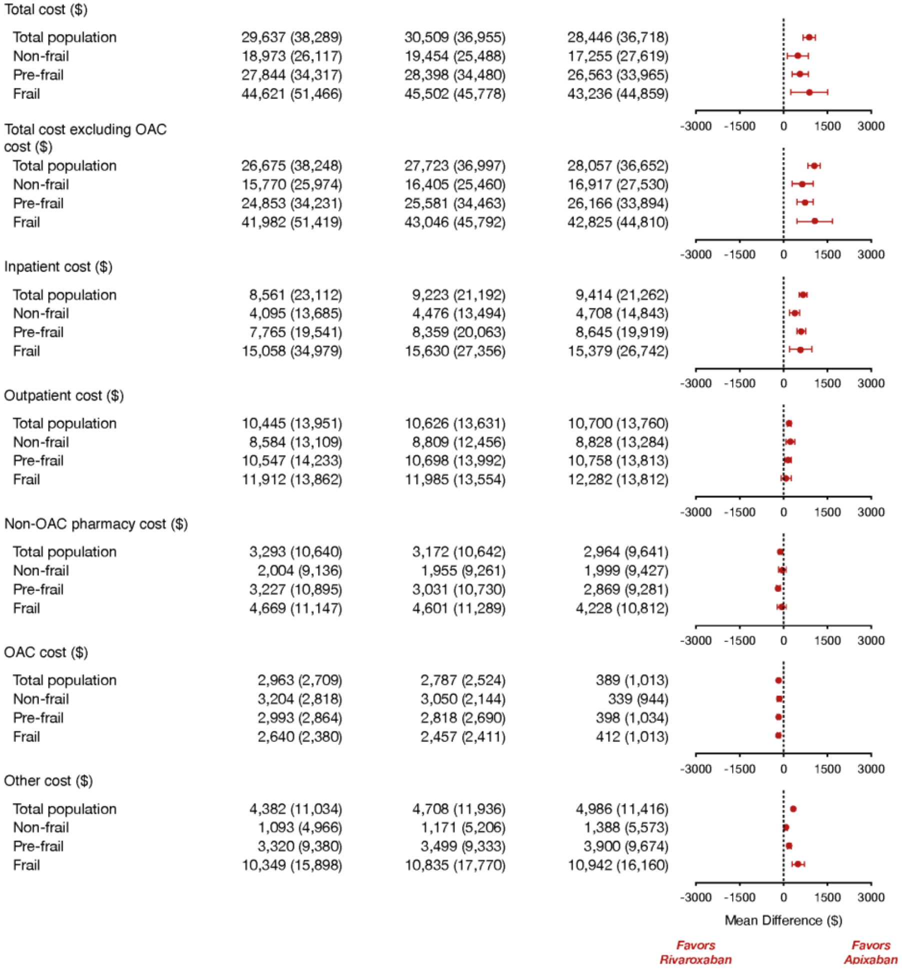

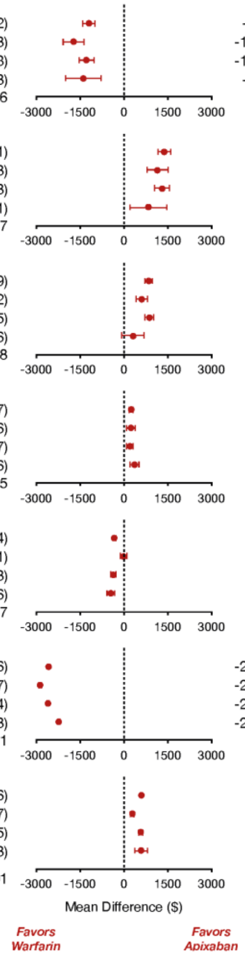

Abbreviations: CI, confidence interval; OAC, oral anticoagulant.
The figure displays the mean cost (standard deviation) in 12 months (365 days) after initiating an oral anticoagulant in the propensity score overlap-weighted populations. *p*-for-heterogeneity tests whether the frailty level-specific estimates are different from each other.

© 2023 Lin KJ et al. *JAMA Network Open.*

eFigure 5. Frailty and Association of Apixaban, Rivaroxaban, and Warfarin With Home Time Loss and Clinical Events in Medicare Fee-for-Service Beneficiaries With Atrial Fibrillation – Sensitivity Analysis 2 to Exclude Beneficiaries with Skilled Nursing Facility Stay in the 365 Days Prior to the Index Date

	Apixaban		Rivaroxaban		Warfarin		Rivaroxaban vs Apixaban	Warfarin vs Apixaban
	Sample Size	Events (Risk or Rate)*	Sample Size	Events (Risk or Rate)*	Sample Size	Events (Risk or Rate)*	Estimate (95% CI)	Estimate (95% CI)
**Home time lost > 14 days in 12 months**								
Total population	40,220	6,497 (16.15)	39,622	6,987 (17.64)	39,592	7,580 (19.15)	1.5 (1.2, 1.8)	3.0 (2.7, 3.3)
Non-frail	8,561	512 (5.98)	8,335	569 (6.83)	8,395	647 (7.70)	0.8 (0.5, 1.2)	1.7 (1.3, 2.2)
Pre-frail	24,711	3,646 (14.75)	24,358	3,870 (15.89)	24,337	4,297 (17.66)	1.1 (0.8, 1.5)	2.9 (2.5, 3.3)
Frail	6,398	2,317 (36.21)	6,360	2,459 (38.65)	6,344	2,525 (39.80)	2.4 (1.5, 3.4)	3.6 (2.6, 4.5)
							$P_{Heterogeneity}$ < 0.001	$P_{Heterogeneity}$ < 0.001
**Composite endpoint**								
Total population	40,220	3,632 (95.34)	39,622	4,262 (114.92)	39,592	4,528 (122.67)	19.6 (14.9, 24.2)	27.3 (22.6, 32.1)
Non-frail	8,561	307 (36.61)	8,335	392 (48.42)	8,395	420 (51.66)	11.8 (5.5, 18.1)	15.0 (8.6, 21.5)
Pre-frail	24,711	2,041 (86.80)	24,358	2,412 (105.24)	24,337	2,570 (112.56)	18.4 (12.8, 24.1)	25.8 (20.0, 31.5)
Frail	6,398	1,267 (223.65)	6,360	1,409 (254.92)	6,344	1,467 (268.54)	31.3 (13.1, 49.4)	44.9 (26.4, 63.3)
							$P_{Heterogeneity}$ = 0.050	$P_{Heterogeneity}$ = 0.001
**Ischemic stroke or systemic embolism**								
Total population	40,220	353 (9.19)	39,622	457 (12.14)	39,592	489 (13.04)	2.9 (1.5, 4.4)	3.8 (2.3, 5.3)
Non-frail	8,561	48 (5.75)	8,335	63 (7.75)	8,395	60 (7.34)	2.0 (-0.5, 4.5)	1.6 (-0.9, 4.1)
Pre-frail	24,711	220 (9.29)	24,358	281 (12.07)	24,337	303 (13.09)	2.8 (0.9, 4.6)	3.8 (1.9, 5.7)
Frail	6,398	84 (14.50)	6,360	111 (19.47)	6,344	114 (20.38)	5.0 (0.2, 9.8)	5.9 (1.0, 10.7)
							$P_{Heterogeneity}$ = 0.558	$P_{Heterogeneity}$ = 0.375
**Major bleeding**								
Total population	40,220	744 (19.45)	39,622	1,167 (31.33)	39,592	1,185 (31.96)	11.9 (9.6, 14.2)	12.5 (10.2, 14.8)
Non-frail	8,561	79 (9.44)	8,335	131 (16.11)	8,395	146 (17.92)	6.7 (3.2, 10.1)	8.5 (4.9, 12.1)
Pre-frail	24,711	450 (19.05)	24,358	720 (31.28)	24,337	700 (30.52)	12.2 (9.3, 15.1)	11.5 (8.6, 14.3)
Frail	6,398	210 (36.86)	6,360	300 (53.85)	6,344	329 (59.82)	17.0 (9.1, 24.9)	23.0 (14.8, 31.1)
							$P_{Heterogeneity}$ = 0.002	$P_{Heterogeneity}$ = 0.002
**Death**								
Total population	40,220	2,809 (72.82)	39,622	2,999 (79.23)	39,592	3,281 (87.16)	6.4 (2.5, 10.3)	14.3 (10.3, 18.4)
Non-frail	8,561	205 (24.28)	8,335	233 (28.41)	8,395	258 (31.39)	4.1 (-0.8, 9.1)	7.1 (2.0, 12.2)
Pre-frail	24,711	1,532 (64.37)	24,358	1,613 (68.93)	24,337	1,806 (77.57)	4.6 (-0.1, 9.2)	13.2 (8.4, 18.0)
Frail	6,398	1,058 (182.63)	6,360	1,121 (196.12)	6,344	1,162 (205.39)	13.5 (-2.4, 29.4)	22.8 (6.6, 38.9)
							$P_{Heterogeneity}$ = 0.527	$P_{Heterogeneity}$ = 0.026

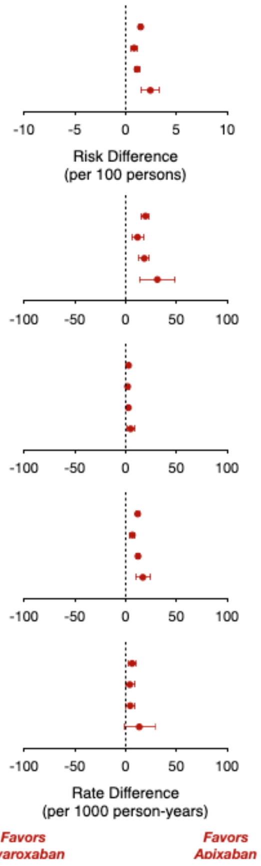

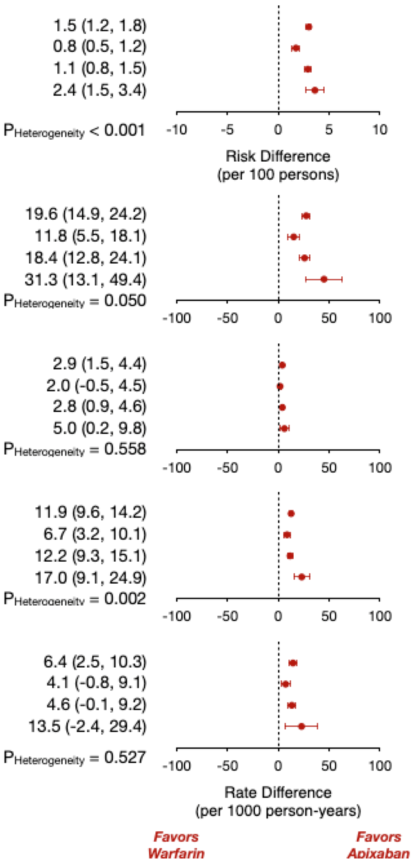

Abbreviation: CI, confidence interval.

The figure displays 1) the risk (per 100 persons) of home time loss >14 days and risk differences (95% confidence interval) and 2) the rates (per 1,000 person-years) of the composite and individual endpoint of ischemic stroke, systemic embolism, major bleeding, or death and rate differences (95% confidence interval) in 12 months (365 days) after initiating an oral anticoagulant in the propensity score overlap-weighted populations. P-for-heterogeneity tests whether the frailty level-specific estimates are different from each other.

© 2023 Lin KJ et al. *JAMA Network Open.*

eFigure 6. Frailty and Association of Apixaban, Rivaroxaban, and Warfarin With Health Care Cost in Medicare Fee-for-Service Beneficiaries With Atrial Fibrillation – Sensitivity Analysis 2 to Exclude Beneficiaries with Skilled Nursing Facility Stay in the 365 Days Prior to the Index Date

	Apixaban Mean (SD)	Rivaroxaban Mean (SD)	Warfarin Mean (SD)	Rivaroxaban vs Apixaban Estimate (95% CI)	Warfarin vs Apixaban Estimate (95% CI)
**Total cost ($)**					
Total population	28,142 (37,284)	28,971 (35,707)	27,038 (35,619)	829 (583, 1075)	-1104 (-1342, -866)
Non-frail	18,928 (26,067)	19,436 (25,486)	17,259 (27,630)	508 (122, 895)	-1669 (-2033, -1305)
Pre-frail	27,483 (34,146)	28,059 (34,327)	26,284 (33,899)	576 (280, 871)	-1199 (-1486, -913)
Frail	43,795 (52,971)	44,861 (45,343)	42,931 (44,406)	1065 (263, 1868)	-864 (-1650, -79)
				$P_{Heterogeneity} = 0.406$	$P_{Heterogeneity} = 0.185$
**Total cost excluding OAC cost ($)**					
Total population	25,153 (37,219)	26,156 (35,739)	26,656 (35,551)	1003 (764, 1241)	1503 (1262, 1744)
Non-frail	15,723 (25,923)	16,383 (25,458)	16,922 (27,541)	660 (297, 1023)	1198 (830, 1568)
Pre-frail	24,487 (34,051)	25,238 (34,308)	25,889 (33,827)	751 (465, 1037)	1402 (1113, 1692)
Frail	41,172 (52,888)	42,418 (45,360)	42,531 (44,361)	1246 (455, 2037)	1358 (566, 2151)
				$P_{Heterogeneity} = 0.357$	$P_{Heterogeneity} = 0.522$
**Inpatient cost ($)**					
Total population	8,010 (22,685)	8,686 (20,499)	8,942 (20,723)	675 (535, 815)	932 (790, 1074)
Non-frail	4,083 (13,674)	4,458 (13,433)	4,694 (14,769)	375 (185, 566)	612 (416, 808)
Pre-frail	7,657 (19,521)	8,285 (20,149)	8,580 (19,941)	628 (463, 793)	924 (755, 1092)
Frail	15,100 (36,939)	15,812 (26,763)	15,746 (26,833)	713 (204, 1223)	646 (138, 1156)
				$P_{Heterogeneity} = 0.223$	$P_{Heterogeneity} = 0.020$
**Outpatient cost ($)**					
Total population	10,411 (14,170)	10,621 (13,846)	10,690 (13,914)	210 (117, 302)	278 (186, 371)
Non-frail	8,571 (13,068)	8,808 (12,471)	8,841 (13,312)	236 (55, 417)	270 (89, 451)
Pre-frail	10,573 (14,323)	10,748 (14,093)	10,807 (13,908)	175 (55, 294)	233 (114, 353)
Frail	12,365 (14,766)	12,476 (14,471)	12,807 (14,392)	111 (-128, 351)	442 (199, 685)
				$P_{Heterogeneity} = 0.442$	$P_{Heterogeneity} = 0.407$
**Non-OAC pharmacy cost ($)**					
Total population	3,207 (10,817)	3,081 (10,804)	2,896 (9,895)	-127 (-199, -55)	-312 (-382, -242)
Non-frail	1,998 (9,129)	1,961 (9,303)	2,009 (9,512)	-37 (-167, 93)	11 (-121, 142)
Pre-frail	3,224 (10,946)	3,011 (10,775)	2,876 (9,420)	-213 (-304, -123)	-349 (-438, -260)
Frail	4,793 (12,144)	4,794 (12,533)	4,336 (11,858)	0 (-209, 210)	-457 (-658, -258)
				$P_{Heterogeneity} = 0.073$	$P_{Heterogeneity} < 0.001$
**OAC cost ($)**					
Total population	2,990 (2,817)	2,817 (2,541)	384 (1,010)	-174 (-206, -141)	-2607 (-2631, -2583)
Non-frail	3,205 (2,823)	3,054 (2,144)	338 (943)	-151 (-228, -75)	-2867 (-2923, -2812)
Pre-frail	2,997 (2,888)	2,822 (2,711)	395 (1,031)	-175 (-217, -133)	-2602 (-2633, -2571)
Frail	2,624 (2,438)	2,444 (2,291)	401 (1,011)	-180 (-250, -111)	-2223 (-2274, -2172)
				$P_{Heterogeneity} = 0.817$	$P_{Heterogeneity} < 0.001$
**Other cost ($)**					
Total population	3,530 (9,690)	3,774 (10,684)	4,134 (10,221)	244 (179, 310)	604 (535, 673)
Non-frail	1,077 (4,925)	1,162 (5,186)	1,384 (5,574)	85 (16, 154)	306 (231, 383)
Pre-frail	3,039 (8,923)	3,201 (8,834)	3,633 (9,295)	162 (89, 235)	594 (516, 673)
Frail	8,920 (14,483)	9,342 (17,166)	9,648 (15,008)	421 (167, 676)	727 (468, 987)
				$P_{Heterogeneity} = 0.001$	$P_{Heterogeneity} < 0.001$

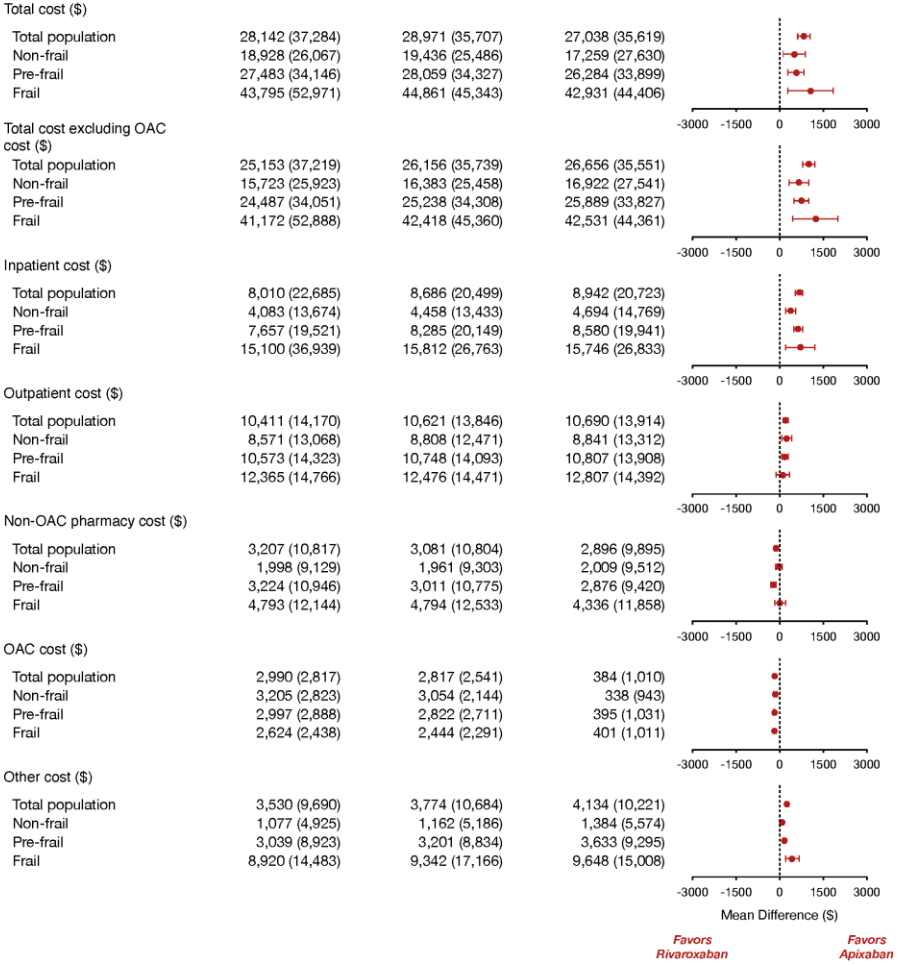

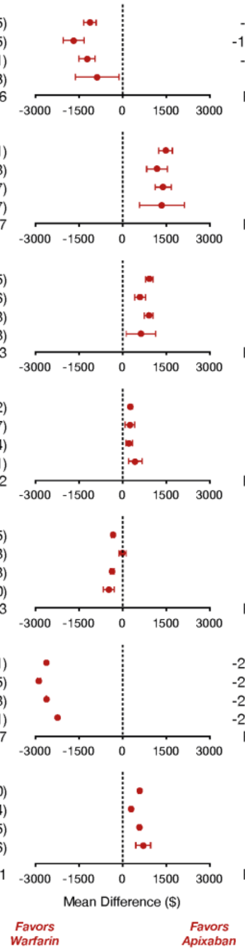

Abbreviations: CI, confidence interval; OAC, oral anticoagulant.
The figure displays the mean cost (standard deviation) in 12 months (365 days) after initiating an oral anticoagulant in the propensity score overlap-weighted populations. P-for-heterogeneity tests whether the frailty level-specific estimates are different from each other.

© 2023 Lin KJ et al. *JAMA Network Open.*

eFigure 7. Frailty and Association of Apixaban, Rivaroxaban, and Warfarin With Home Time Loss and Clinical Events in Medicare Fee-for-Service Beneficiaries With Atrial Fibrillation – Sensitivity Analysis 2 to Exclude Data from 2020

	Apixaban		Rivaroxaban		Warfarin		Rivaroxaban vs Apixaban	Warfarin vs Apixaban
	Sample Size	Events (Risk or Rate)*	Sample Size	Events (Risk or Rate)*	Sample Size	Events (Risk or Rate)*	Estimate (95% CI)	Estimate (95% CI)
**Home time lost > 14 days in 12 months**								
Total population	36,005	5,825 (16.18)	35,611	6,298 (17.69)	35,552	6,867 (19.32)	1.5 (1.2, 1.8)	3.1 (2.8, 3.5)
Non-frail	7,851	463 (5.89)	7,728	528 (6.84)	7,749	598 (7.72)	0.9 (0.5, 1.4)	1.8 (1.4, 2.3)
Pre-frail	22,104	3,264 (14.77)	21,859	3,483 (15.93)	21,822	3,875 (17.76)	1.2 (0.8, 1.5)	3.0 (2.6, 3.4)
Frail	5,772	2,088 (36.18)	5,742	2,224 (38.73)	5,720	2,308 (40.34)	2.6 (1.6, 3.5)	4.2 (3.2, 5.2)
							$P_{Heterogeneity} = 0.013$	$P_{Heterogeneity} < 0.001$
**Composite endpoint**								
Total population	36,005	3,236 (94.86)	35,611	3,829 (114.88)	35,552	4,100 (123.79)	20.0 (15.1, 24.9)	28.9 (23.9, 33.9)
Non-frail	7,851	276 (35.92)	7,728	365 (48.62)	7,749	390 (51.90)	12.7 (6.1, 19.2)	16.0 (9.3, 22.7)
Pre-frail	22,104	1,813 (86.18)	21,859	2,156 (104.79)	21,822	2,329 (113.86)	18.6 (12.7, 24.6)	27.7 (21.6, 33.8)
Frail	5,772	1,137 (222.27)	5,742	1,276 (255.76)	5,720	1,336 (271.59)	33.5 (14.4, 52.6)	49.3 (29.8, 68.8)
							$P_{Heterogeneity} = 0.090$	$P_{Heterogeneity} = 0.001$
**Ischemic stroke or systemic embolism**								
Total population	36,005	314 (9.13)	35,611	410 (12.11)	35,552	449 (13.35)	3.0 (1.4, 4.5)	4.2 (2.6, 5.8)
Non-frail	7,851	43 (5.55)	7,728	58 (7.62)	7,749	56 (7.36)	2.1 (-0.5, 4.6)	1.8 (-0.7, 4.4)
Pre-frail	22,104	197 (9.30)	21,859	251 (12.01)	21,822	282 (13.58)	2.7 (0.7, 4.7)	4.3 (2.2, 6.3)
Frail	5,772	74 (14.14)	5,742	100 (19.44)	5,720	107 (21.23)	5.3 (0.3, 10.3)	7.1 (1.9, 12.2)
							$P_{Heterogeneity} = 0.532$	$P_{Heterogeneity} = 0.130$
**Major bleeding**								
Total population	36,005	672 (19.64)	35,611	1,051 (31.39)	35,552	1,083 (32.53)	11.8 (9.3, 14.2)	12.9 (10.5, 15.3)
Non-frail	7,851	72 (9.38)	7,728	128 (16.97)	7,749	135 (17.93)	7.6 (3.9, 11.2)	8.6 (4.8, 12.3)
Pre-frail	22,104	405 (19.18)	21,859	644 (31.14)	21,822	638 (31.04)	12.0 (8.9, 15.0)	11.9 (8.8, 14.9)
Frail	5,772	193 (37.45)	5,742	272 (54.19)	5,720	301 (60.86)	16.7 (8.4, 25.1)	23.4 (14.7, 32.1)
							$P_{Heterogeneity} = 0.065$	$P_{Heterogeneity} = 0.008$
**Death**								
Total population	36,005	2,492 (72.14)	35,611	2,690 (79.05)	35,552	2,956 (87.48)	6.9 (2.8, 11.0)	15.3 (11.1, 19.6)
Non-frail	7,851	184 (23.82)	7,728	211 (27.80)	7,749	240 (31.54)	4.0 (-1.1, 9.1)	7.7 (2.5, 13.0)
Pre-frail	22,104	1,355 (63.59)	21,859	1,444 (68.73)	21,822	1,628 (78.00)	5.1 (0.2, 10.0)	14.4 (9.3, 19.5)
Frail	5,772	946 (180.78)	5,742	1,009 (195.58)	5,720	1,053 (206.64)	14.8 (-1.9, 31.5)	25.9 (8.9, 42.8)
							$P_{Heterogeneity} = 0.477$	$P_{Heterogeneity} = 0.051$

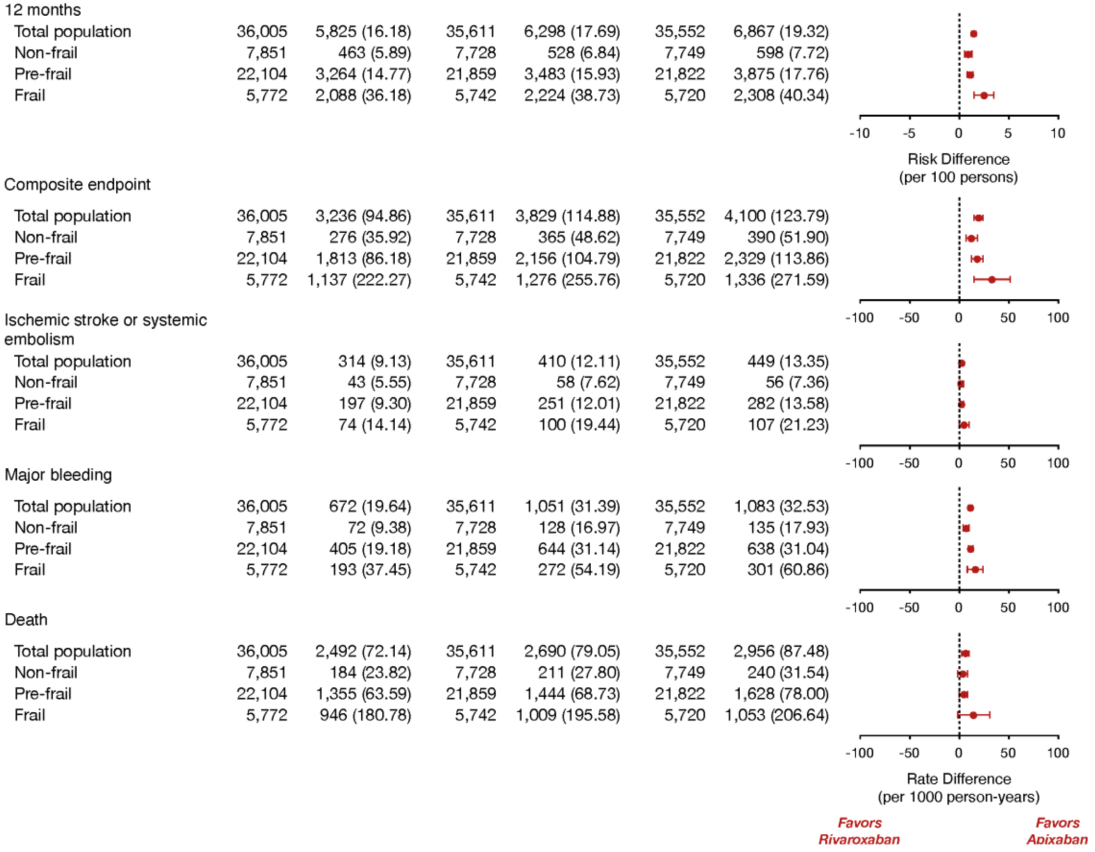

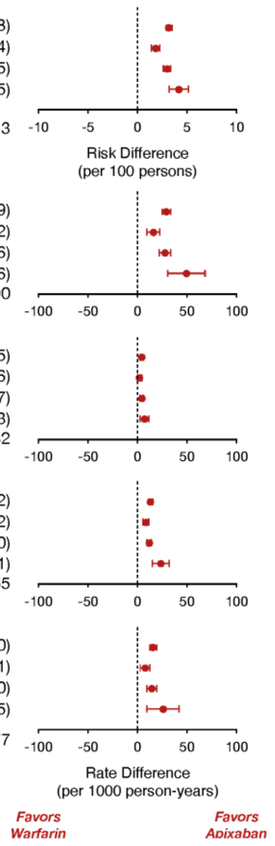

Abbreviation: CI, confidence interval.

The figure displays 1) the risk (per 100 persons) of home time loss >14 days and risk differences (95% confidence interval) and 2) the rates (per 1,000 person-years) of the composite and individual endpoint of ischemic stroke, systemic embolism, major bleeding, or death and rate differences (95% confidence interval) in 12 months (365 days) after initiating an oral anticoagulant in the propensity score overlap-weighted populations. P-for-heterogeneity tests whether the frailty level-specific estimates are different from each other.

© 2023 Lin KJ et al. *JAMA Network Open.*

eFigure 8. Frailty and Association of Apixaban, Rivaroxaban, and Warfarin With Health Care Cost in Medicare Fee-for-Service Beneficiaries With Atrial Fibrillation – Sensitivity Analysis 2 to Exclude Data From 2020

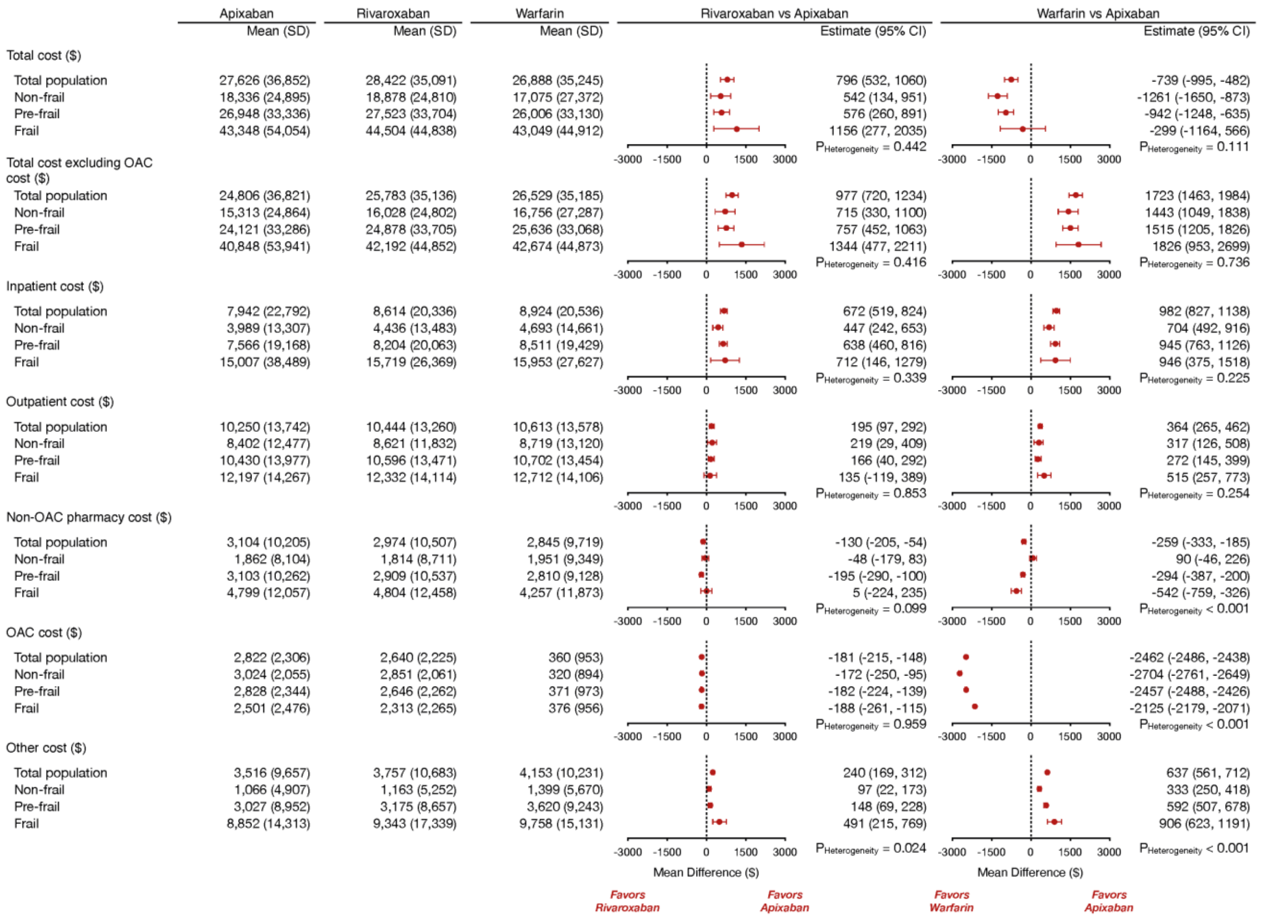

Abbreviations: CI, confidence interval; OAC, oral anticoagulant.
The figure displays the mean cost (standard deviation) in 12 months (365 days) after initiating an oral anticoagulant in the propensity score overlap-weighted populations. *p*-for-heterogeneity tests whether the frailty level-specific estimates are different from each other.

© 2023 Joshua K et al. *JAMA Network Open.*
