# Supplementary material for: Frailty, Home Time, and Health Care Costs in Older Adults With Atrial Fibrillation Receiving Oral Anticoagulants
Source: JAMA Netw Open. 2023 Nov 9;6(11):e2342264. doi: 10.1001/jamanetworkopen.2023.42264 (PMC10636636; doi:10.1001/jamanetworkopen.2023.42264)
Supplement: Supplement 2. — Data Sharing Statement [file jamanetwopen-e2342264-s002.pdf]

Data Sharing Statement

Lin. Frailty, Home Time, and Health Care Costs in Older Adults With Atrial Fibrillation Receiving Oral Anticoagulants. *JAMA Netw Open*. Published November 09, 2023. doi:10.1001/jamanetworkopen.2023.42264

Data
Data available: No
